# Diagnosis of Persistent Fever in the Tropics: Set of Standard Operating Procedures Used in the NIDIAG Febrile Syndrome Study

**DOI:** 10.1371/journal.pntd.0004749

**Published:** 2016-11-03

**Authors:** Emilie Alirol, Ninon Seiko Horie, Barbara Barbé, Veerle Lejon, Kristien Verdonck, Philippe Gillet, Jan Jacobs, Philippe Büscher, Basudha Kanal, Narayan Raj Bhattarai, Sayda El Safi, Thong Phe, Kruy Lim, Long Leng, Pascal Lutumba, Deby Mukendi, Emmanuel Bottieau, Marleen Boelaert, Suman Rijal, François Chappuis

**Affiliations:** 1 Clinical Research Centre, Geneva University Hospitals, Geneva, Switzerland; 2 Division of Tropical and Humanitarian Medicine, Geneva University Hospitals, Geneva Switzerland; 3 Institute of Tropical Medicine, Antwerp, Belgium; 4 Institut de Recherche pour le Développement (IRD), Unité Mixte de Recherche IRD-CIRAD 177, Montpellier, France; 5 Department of Microbiology and Immunology, KU Leuven, Leuven, Belgium; 6 B.P. Koirala Institute of Health Science, Dharan, Nepal; 7 Faculty of Medicine, University of Khartoum, Khartoum, Sudan; 8 Sihanouk Hospital Center of HOPE, Phnom Penh, Cambodia; 9 Institut National de Recherche Biomédicale, Kinshasa, Democratic Republic of the Congo; 10 Département de Médecine Tropicale, Université de Kinshasa, Kinshasa, Democratic Republic of the Congo; Common Heritage Foundation, NIGERIA

## Abstract

In resource-limited settings, the scarcity of skilled personnel and adequate laboratory facilities makes the differential diagnosis of fevers complex [1–5]. Febrile illnesses are diagnosed clinically in most rural centers, and both Rapid Diagnostic Tests (RDTs) and clinical algorithms can be valuable aids to health workers and facilitate therapeutic decisions [6,7]. The persistent fever syndrome targeted by NIDIAG is defined as presence of fever for at least one week. The NIDIAG clinical research consortium focused on potentially severe and treatable infections and therefore targeted the following conditions as differential diagnosis of persistent fever: visceral leishmaniasis (VL), human African trypanosomiasis (HAT), enteric (typhoid and paratyphoid) fever, brucellosis, melioidosis, leptospirosis, malaria, tuberculosis, amoebic liver abscess, relapsing fever, HIV/AIDS, rickettsiosis, and other infectious diseases (e.g., pneumonia). From January 2013 to October 2014, a prospective clinical phase III diagnostic accuracy study was conducted in one site in Cambodia, two sites in Nepal, two sites in Democratic Republic of the Congo (DRC), and one site in Sudan (clinicaltrials.gov no. NCT01766830). The study objectives were to (1) determine the prevalence of the target diseases in patients presenting with persistent fever, (2) assess the predictive value of clinical and first-line laboratory features, and (3) assess the diagnostic accuracy of several RDTs for the diagnosis of the different target conditions.

In order to ensure consistency across study sites and compliance with Good Clinical (Laboratory) Practices (GC[L]P) and other applicable regulatory requirements, a robust quality assurance system was developed and implemented prior to recruitment start. This symposium describes the different components of the quality assurance system, introduces the set of standard operating procedures (SOPs) specifically developed for the NIDIAG persistent fever syndrome study, and identifies key challenges encountered by study sites in conducting the study in compliance with this quality framework.

## What is a study quality assurance system and why was it required in the NIDIAG persistent fever syndrome study?

The quality assurance system of a clinical study usually includes the development and implementation of SOPs along with staff training and internal and external quality control (QC) activities [8]. SOPs are powerful tools to enhance the quality of clinical research activities through harmonization and standardization. By providing detailed instructions and guidance to operators performing a given task, SOPs enable researchers to achieve uniformity in the performance of this task across different sites and for the total duration of the study. SOPs also help ensure that the study is implemented according to what is set forth in the protocol and that measures to control for bias are in place. This in turn ensures that robust, accurate, and reproducible study data are generated, and that reliable conclusions are drawn from studies. Well-designed SOPs not only warrant quality and integrity of study data, but they also leave a permanent record of the methods employed and help provide public assurance that ethical standards are met and patients’ safety is safeguarded. Last but not least, SOPs help ensure compliance with regulations and international standards. According to GCP guidelines, sponsors are responsible for implementing and maintaining appropriate quality assurance and QC systems, including SOPs for all research-related activities. Many clinical studies develop their own set of SOPs, and some research groups have made them public [9–11].

Training is another essential pillar of any study quality assurance system. Training of staff involved in clinical research is not only an ethical mandate, but it is also key to a successful study. Although often seen as a one-time activity that takes place before study start, training is actually an ongoing pursuit. Staff turnover, protocol amendments, changes in disease diagnosis and management recommendations, or detection of protocol deviations all prompt additional or refresh trainings. As far as possible, training should be tailored to the specific objectives of the study and should be articulated around the study SOPs. Whenever possible, dummy runs should be organized as part of the initial study training to verify that everything works in order before the first patient is included.

QC encompasses a set of internal and external activities that are crucial to verify that the research is being conducted in accordance with initial plans and with applicable standards and regulatory requirements. Internal QC is continuous and concurrent with the conduct of the clinical study and is carried out by the research staff. Internal QC includes, for example, the definition of reference standards and calibrators for laboratory measurements and the evaluation of day-to-day conformity with these standards. External QC, on the other hand, is conducted by independent organizations and is often linked to accreditation processes. It usually includes monitoring, which takes place at regular, predefined intervals, and external audits.

The six study sites participating in the NIDIAG persistent fever syndrome study showed a high heterogeneity with regards to epidemiological context (i.e., prevalence of target conditions), diagnostic-treatment algorithms in use, existing disease control activities, laboratory capacity and health staff knowledge, and experience in clinical research. Hence, harmonizing study activities throughout study sites was paramount. In addition, the high number of laboratory procedures, some of which were introduced for the purpose of the study in local laboratories, necessitated detailed guidance, extensive training and coaching of laboratory staff, and a robust QC framework.

## How was the NIDIAG persistent fever syndrome study conducted: how were patients recruited and followed-up, and what diagnostic work-up was performed?

The NIDIAG persistent fever syndrome study targeted all patients presenting with more than one week of fever at one of the study sites during the study period. In all sites, patients aged five years and above were included, except in Cambodia, where only adults were included. Patients with an existing laboratory-confirmed diagnosis (including diseases not targeted by NIDIAG) and patients in need of immediate intensive care due to shock or respiratory distress were excluded from the study. Study investigators assessed inclusion and exclusion criteria through history taking, review of existing medical documents, and rapid clinical assessment.

After providing informed consent, patients underwent a full clinical assessment as well as blood and urine sampling, on which a standard, country-specific panel of ancillary, RDT, and reference laboratory testing was performed (see Table 1). The health care provider remained free to order other optional diagnostic procedures (e.g., chest X-ray, abdominal ultrasound). Patients were followed-up daily during their hospital stay or at each outpatient visit for those who were not hospitalized. A single systematic follow-up visit was performed at four weeks post discharge to assess clinical response to treatment and to collect additional serum samples. Additional follow-up visits were scheduled at the discretion of the care provider.

**Table 1:** Diagnostic plan for the NIDIAG persistent fever syndrome study.

	Sudan	DRC	Cambodia	Nepal
Screening for entry criteria	x	x	x	x
Medical history	x	x	x	x
Clinical examination	x	x	x	x
Ancillary blood testing (on-site unless stated otherwise)				
Hemoglobin concentration determination	x	x	x	x
WBC cell count	x	x	x	x
Blood chemistry: ASAT, ALAT, Bilirubin, urea, creatinine	x	x	x	x
RDT on whole blood/serum (on-site)				
HAT-RDTs		x		
CATT whole blood and dilution		x		
rK28 RDT	x			x^2^
rK39 IT-LEISH	x			x^2^
Malaria-RDT (SD)	x	x	x	x
Malaria-RDT (CareStart)		x		
HIV RDTs (2–3 per site, see text)	x	x	x	x
Typhidot Rapid IgM (Reszon Diagnostic Int.)	x	x	x	x
S. Typhi IgM/IgG (SD)	x	x	x	x
Test-it Typhoid IgM (Life Assay)	x	x	x	x
Test-it Leptospirosis IgM (Life Assay)	x	x	x	x
Leptospira IgG/IgM (SD)	x	x	x	x
Urinalaysis (on-site)				
Dipstick including • Leucocytes and leukocyte esterase • Nitrite • Urobilinogen • Proteins • pH • Erythrocytes/Hemoglobin • Ketones • Specific gravity • Bilirubin • Glucose	x	x	x	x
Reference blood testing				
Search for trypanosomes, with concentration methods (on-site)		x		
Direct Agglutination Test (on-site) for Leishmaniasis	x			x^1^^,^^2^
Microscopic examination of thick/thin films (on-site)	x	x	x	x
Blood cultures for *Salmonella* sp. (on-site unless stated otherwise)	x^1^	x^1^	x	x^1^
Blood cultures for *Brucella* sp. (on-site unless stated otherwise)	x^1^	x^1^	x^1^	
Blood cultures for *Burkholderia pseudomallei*			x	
Serologies for *Brucella* sp. (international reference lab)	x	x	x	x
Serologies for *Entamoeba histolytica* (international reference lab)	(x)^3^	(x)	(x)	(x)
PCR for *Borrelia*, *Rickettsia*, and *Leptospira* sp. (international reference lab)	x	x	x	x
Serologies for *Rickettsia* sp. (international reference lab)	x	x	x	x
Serologies for *Leptospira* sp. (MAT) (international reference lab)	x	x	x	x
Other tests				
Microscopic search for *Leishmania donovani* on bone marrow/lymph node aspirate (on-site)	x			x^1^^,^^2^
Culture of *L*. *donovani* from bone marrow/lymph node aspirate (on-site)	x			x^1^^,^^2^
CSF examination^4^	(x)	(x)	(x)	(x)
Urine culture (if leukocyte esterase positive on urine dipstick)			(x)	(x)
Urine: PCR for *Leptospira* sp. (international reference lab)	x	x	x	x
Sputum (or other biological fluid) microscopic examination for acid fast bacilli	(x)	(x)	(x)	(x)
Sputum (or other biological fluid) culture for *Mycobacterium tuberculosis* (on-site unless stated otherwise)	(x)^1^		(x)	(x)^1^
PCR (GeneXpert or GenoType MTBDRplus Assay from Hain Lifesciences) for *M*. *tuberculosis* (on-site unless stated otherwise)	(x)^1^	(x)^1^	(x)^1^	(x)^1^
Bone Marrow Culture (search for *Salmonella Typhi/Paratyphi*)	(x)^1^		(x)^1^	(x)^1^
Culture of *B*. *pseudomallei* (various sites: e.g., throat, liver abscess, etc.)			(x)	
Chest X-ray^5^ (on-site unless stated otherwise)	(x)^1^	(x)	(x)	(x)
Abdominal ultrasound^5^ (on-site unless stated otherwise)	(x)^1^	(x)	(x)	(x)^1^
Microscopic examination of abscess material (search for *Entamoeba histolytica*)	(x)^1^	(x)	(x)	(x)^1^
PCR examination on abscess material (search for *E*. *histolytica*) (international reference lab)	(x)	(x)	(x)	(x)
Therapeutic responses^6^				
Response to anti-trypanosomal drugs		(x)		
Response to anti-leishmanial drugs	(x)			(x)
Response to anti-malarials	(x)	(x)	(x)	(x)
Response to empirical or targeted antibiotics	(x)	(x)	(x)	(x)
Response to anti-TB drugs	(x)	(x)	(x)	(x)

ASAT: Aspartate aminotransferase; ALAT: Alanine transaminase; CATT: Card Agglutination Test for Trypanosomiasis; CSF: Cerebro-Spinal Fluid; MAT: Microscopic Agglutination Test; PCR: Polymerase Chain Reaction; SD: Standard Diagnostics; TB: Tuberculosis; WBC: Whole Blood Cell.

^1^ Test performed in reference laboratories or referral hospital.

^2^ Test not performed in Dhankuta Hospital, as VL is not endemic in the area.

^3^ “(x)” meant that these tests were not performed for all study patients but only for a subset of them according to specific/presumptive diagnosis and clinical evolution.

^4^ Lumbar puncture and CSF examination were only performed in patients with proven or suspected HAT (DRC) and in patients with clinical suspicion of meningoencephalitis (all sites).

^5^ Chest X-ray and abdominal ultrasound were performed in all patients with focal symptoms or signs indicative of thoracic (e.g., suspicion of pulmonary TB) or abdominal (e.g., suspicion of intra-abdominal abscess) condition and in all patients with no proven diagnosis after completion of the initial laboratory work-up.

^6^ Therapeutic response to various treatments was assessed at patient’s discharge and at week four post discharge by history taking, clinical examination, and, at the discretion of the physician in charge, biological markers.

All new or persistent clinical symptoms and signs were recorded, together with new therapies and treatment modifications. After completing the first-line diagnostic work-up, patients for whom no confirmed or presumptive diagnosis could be established underwent further testing, including chest X-ray, abdominal ultrasound, sputum collection, lymph node puncture, bone marrow aspiration, and lumbar puncture (if indicated by the appearance of a new clinical feature or nonresponse to initial treatment). Results of this second-line diagnostic work-up were also recorded.

## What SOPs were developed for the NIDIAG persistent fever syndrome study?

A set of 46 SOPs were developed specifically for the persistent fever syndrome study (see S1 Table and S1 Text). Those included clinical SOPs (4), laboratory SOPs (29), data management SOPs (2), and QC SOPs (11). Besides these, a few additional SOPs had to be developed for and adapted to local circumstances (e.g., SOP on study samples transport or SOP on inoculating and growing bacterial cultures). Those SOPs were meant to be used by different target groups: clinicians and health care workers, laboratory staff, data entry clerks and data managers, and staff involved in QC activities. SOPs were made available in both French and English, depending on whether they were meant for French- or English-speaking staff. All SOPs used in the persistent fever syndrome study can be found in annex, except site-specific SOPs and SOPs in French, which are available upon request.

All NIDIAG SOPs were developed based on a template and following a SOP for developing SOP to ensure consistency in among all different SOPs developed. A specific numbering system was used, and each SOP received a unique identification number. The tasks for SOP development were shared in the research group: principal investigators, laboratory referents, and QC experts contributed as first authors for specific SOPs. Each draft SOP was then reviewed by a second author who was familiar with the procedure described. Input from study site teams was sought through specific training workshops and piloting of the SOPs. Finally, all SOPs and their annexes were reviewed and approved by the GC(L)P expert who was responsible for ensuring consistency with other documentations and maintaining version control. All approved SOPs were published as PDF files on the NIDIAG website and were made available for study sites to download. Study personnel were informed of SOPs revisions, and additional training was organized when necessary.

## What challenges did study sites meet in conducting the study and complying with the SOPs?

Between January 2013 and October 2014, a total of 3,409 patients were screened at the 6 study sites (427 in Cambodia, 856 in Sudan, 646 in Nepal, and 1,480 in DRC), of whom 1,927 were included in the NIDIAG persistent fever syndrome study (378 in Cambodia, 670 in Sudan, 578 in Nepal, and 301 in DRC). Eighty-six (4.5%) patients died, and 454 (23.5%) were lost to follow-up (LFU) during the study. After the last patient’s last visit, a short survey was conducted among research staff about the challenges faced during the study. The outcomes of this survey are shown in Tables 2 and 3. Most challenges highlighted by the research teams pertained to logistical and organizational aspects; however, compliance with quality standards and adherence to SOPs was also listed by laboratory staff. This perceived difficulty could be related to the high number of SOPs implemented or to the relative inexperience of some teams in conducting GC(L)P-compliant studies. Recruitment and retention of study participants was also among the main challenges faced by study sites. This is in line with published research, which shows that recruitment and retention of research participants is among the top challenges of clinical research today. According to a recent report, 89% of 150 clinical studies assessed met the recruitment target, but time for doing so was generally double what was forecasted in the study protocol [12].

**Table 2:** Challenges encountered by the laboratory staff during the NIDIAG persistent fever syndrome study.

	Sudan	DRC	Cambodia	Nepal
High number of tests to be performed daily due to the high recruitment rate	x	x		
Identification of adequate national laboratory capacity to perform reference tests		x	x	
Timely transport of biological samples to national reference laboratories	x	x		x
Coordination between different national reference laboratories	x			
Ensuring strict compliance with GCLP at peripheral level		x		x
Ensuring strict compliance with laboratory SOPs	x		x	
Delays in laboratory materials and RDTs supply, resulting in short time window for use		x	x	x
Complexity of data collection forms			x	
Ensuring uninterrupted power supply at peripheral level		x		x
Disappointing quality of certain index tests, requiring adaptation of manufacturer’s instruction		x		x
Culture contamination	x	x		

**Table 3:** Challenges encountered by the clinical staff during the NIDIAG persistent fever syndrome study.

	Sudan	DRC	Cambodia	Nepal
Ensuring only eligible patients are included	x	x		
Achieving target sample size within study timeframe and budget	x	x^1^	x	
Recruiting enough participants during the rainy season	x			
Obtaining consent in view of the amount of blood to be collected	x			x
Obtaining consent in view of the length of follow-up and number of follow-up visits foreseen				x
Identifying suitable witness for consent of illiterate patients in view of the low literacy rate		x		
Several patients in critical state complicating the clinical management		x	x	
Organizing referral of study participants to higher-level hospitals	x	x		
Minimizing dropouts		x		x
Ensuring continuity in study quality with high staff turnover			x	x
Complexity of data collection forms			x	
Simultaneous conduct of the NIDIAG Neurological Syndrome Study		x		

^1^ In DRC, in order not to miss any of the eligible patients, all patients presenting with fever were screened by the local investigators. Besides, in order to reach recruitment target, a second site had to be opened during the study to boost recruitment. This resulted in increased costs and increased workload for research staff.

QC activities conducted during the NIDIAG persistent fever syndrome study did not identify significant deviations from the protocol and SOPs, and compliance was found very good overall, suggesting that most end users found the SOPs valuable and convenient to use. In conclusion, although labor-intensive, the development of SOPs for the febrile syndrome study proved extremely useful to ensure consistency between the different study sites and to warrant the quality of the data generated.

## Supporting Information

S1 Table Overview of the standard operating procedures (SOPs) used in the NIDIAG febrile syndrome study. (DOCX)

S1 Text Set of SOPs used in the NIDIAG persistent fever syndrome study. (PDF)
