# Supplementary material for: Diagnosis of Persistent Fever in the Tropics: Set of Standard Operating Procedures Used in the NIDIAG Febrile Syndrome Study
Source: PLoS Negl Trop Dis. 2016 Nov 3;10(11):e0004749. doi: 10.1371/journal.pntd.0004749 (PMC5094701; doi:10.1371/journal.pntd.0004749)
Supplement: S1 Table — (DOCX) [file pntd.0004749.s001.docx]

**S1 table:** Overview of the standard operating procedures (SOPs) used in the NIDIAG febrile syndrome study.

|  | **Type of SOP** | **Purpose of SOP** | **End User** | **Languages** |
| --- | --- | --- | --- | --- |
| 1 | Clinical | Assessing inclusion / exclusion criteria | study investigators | English and French |
| 2 | Clinical | Clinical examination at baseline and during follow-up | study investigators | English and French |
| 3 | Clinical | Performing lumbar puncture^1^ | study investigators | French |
| 4 | Clinical | Performing bone marrow aspirate | study investigators | English |
| 5 | Laboratory | Urine sampling^1^ | site investigator/nurse | English and French |
| 6 | Laboratory | Blood sampling^1^ | site investigator/nurse | English |
| 7 | Laboratory | Collection of Lymph Node (LN) aspirates | Site investigator/nurse | English |
| 8 | Laboratory | Preparation of thick blood film, Giemsa staining and microscopic examination^1^ | lab technicians | English and French |
| 9 | Laboratory | Storage of bacterial isolates^1^ | Lab technicians | English |
| 10 | Laboratory | Use of urine test strips Multistix X-SG^1^ | lab technicians | French |
| 11 | Laboratory | Fresh trypanosome examination in LN aspirates^1^ | Lab technicians | French |
| 12 | Laboratory | Mini anion exchange centrifugation technique (mAECT)^1^ | Lab technicians | French |
| 13 | Laboratory | Modified Single Centrifugation (MSC)^1^ | lab technicians | French |
| 14 | Laboratory | Capillary Tube Centrifugation (CTC)^1^ | Lab technicians | French |
| 15 | Laboratory | Card Agglutination Test for *T.b. gambiense*^1^ | Lab technicians | French |
| 16 | Laboratory | Performing the SD Bioline HAT test (Standard Diagnostics)^1^ | Lab technicians | French |
| 17 | Laboratory | Performing the Gambiense sero-K-Set test (Coris bioconcept)^1^ | Lab technicians | French |
| 18 | Laboratory | Blood: RDT malaria Carestart PDLH^1^ | lab technicians | English |
| 19 | Laboratory | Blood: RDT malaria SD Bioline 60^1^ | lab technicians | English |
| 20 | Laboratory | Performing the DAT | lab technicians | English |
| 21 | Laboratory | Inoculating and growing cultures of *L. donovani* from body fluids | lab technicians | English |
| 22 | Laboratory | Detecting *L. donovani* in body fluids and tissue aspirates | lab technicians | English |
| 23 | Laboratory | Performing the rK28 from EASE-Medtrend | lab technicians | English |
| 24 | Laboratory | Performing the rK39 IT LEISH (Bio-Rad) | lab technicians | English |
| 25 |  |  |  |  |
| 26 | Laboratory | Performing the Typhidot Rapid IgM (Reszon Diagnostics) | lab technicians | English and French |
| 27 | Laboratory | Performing the S. Typhi IgM/IgG (SD Bioline) | lab technicians | English and French |
| 28 | Laboratory | Performing the Test-it Typhoid IgM (Life Assay) | lab technicians | English and French |
| 29 | Laboratory | Performing the Test-it Leptospirosis IgM (Life Assay) | lab technicians | English and French |
| 30 | Laboratory | Performing the Leptospira IgG/IgM (SD Bioline) | lab technicians | English and French |
| 31 | Laboratory | Blood : RDT HIV Determine^1^ | lab technicians | English and French |
| 32 | Laboratory | Use of the Reflotron plus (Roche) biochemical analyser^1^ | lab technicians | French |
| 33 | Laboratory | Sputum collection, smear and Ziehl Neelsen staining for detection of mycobacteria^1^ | lab technicians | French |
| 34 | Data Management | Completing CRFs^1^ | Site investigator | English and French |
| 35 | Data Management | Procedure for data management (data entry)^1^ | clinical/lab staff, data entry clerks | English |
| 36 | Quality Control | Obtaining informed consent ^1^ | Site investigator | English and French |
| 37 | Quality Control | Patient & sample numbering and labelling^1^ | site investigators / lab technician | English and French |
| 38 | Quality Control | Management of study documents^1^ | PI/Site investigator/nurse/lab technicians | English and French |
| 39 | Quality Control | SOP on SOP^1^ | SOP author | English and French |
| 40 | Quality Control | Monitoring study centre^1^ | PI/Site investigator | English |
| 41 | Quality Control | Internal Quality Control Activities^1^ | Quality Managers | English and French |
| 42 | Quality Control | Lab Supervision visit^1^ | Lab Expert | English and French |
| 43 | Quality Control | Storing and Handling RDTs^1^ | Lab Technicians | English and French |
| 44 | Quality Control | How to install and use the Min/max thermometer^1^ | Lab Technicians/quality manager | English and French |
| 45 | Quality Control | Stock Management^1^ | Lab Technicians/quality manager | English |
| 46 | Quality Control | Handling of expired & disqualified products^1^ | Study Investigators and Lab technicians | English and French |

^1^ these SOPs were also used in other NIDIAG syndromes.
