# Supplementary material for: Diagnosis of Persistent Fever in the Tropics: Set of Standard Operating Procedures Used in the NIDIAG Febrile Syndrome Study
Source: PLoS Negl Trop Dis. 2016 Nov 3;10(11):e0004749. doi: 10.1371/journal.pntd.0004749 (PMC5094701; doi:10.1371/journal.pntd.0004749)
Supplement: S1 Text — (PDF) [file pntd.0004749.s002.pdf]

SOP Manual

- Febrile Syndrome -

Table of Content

Clinical SOPs		Page #
Assessing inclusion/exclusion criteria	SOP-WP2-CLIN-05-V1-09Oct2012	4
Clinical examination at baseline and during follow-up	SOP-WP2-CLIN-06-V1-15Nov2012	6
Performing Bone marrow aspirate	SOP-WP2-CLIN-09-V1-08Nov2012	8
**Laboratory SOPs**		
Urine sampling	SOP-WP2-LAB-36-v1.1-22Aug2012	11
Blood sampling	SOP-WP2-LAB-44-v1.1-27Dec2012	13
Collection of lymph node (LN) aspirates	SOP-WP2-LAB-48-V1- 07Dec2012	17
Preparation of thick blood film, Giemsa staining and microscopic examination	SOP-WP2-LAB-20-V1.1-22Aug2012	20
Storage of bacterial isolates	SOP-WP6-LAB-01-V1-15Apr2013	26
Blood : RDT malaria SD Bioline 60	SOP-WP2-LAB-35-V2.1-11Apr2014	32
Performing the DAT<br>*+ 2 annexes*	SOP-WP2-LAB-17-V1-10Sep2013<br>*SOP-WP2-LAB-17-V1-10Sep2013-Annex1/2*	36
Inoculating and growing cultures of *Leishmania donovani* from tissue aspirates (Nepal)<br>*+ 4 annexes*	SOP-WP2-LAB-16-V2.0 Nepal-09Oct2013<br>*SOP-WP2-LAB-16-V01-09Oct2013-Annex1/2/3/4*	46
Inoculating and growing cultures of *Leishmania donovani* from tissue aspirates (Sudan)<br>*+ 1 annex*	*SOP-WP2-LAB-16-V2.1-10Sep2013*<br>*SOP-WP2-LAB-16-V2.1-10Sep2013-Annex1*	53
Detecting *L. donovani* in body fluids and tissue aspirates	SOP-WP2-LAB-15-V1-18Oct2012	58
Performing the rK28 from EASE-Medtrend	SOP-WP2-LAB-06-V4-20Aug2013	62
Performing the rK39 IT LEISH (Bio-Rad)	SOP-WP2-LAB-05-V3-11Feb2013	67
Performing the Typhidot Rapid IgM (Reszon Diagnostics)	SOP-WP2-LAB-10-V3-11Feb2013	72
Performing the *S. Typhi* IgM/IgG (SD Bioline)	SOP-WP2-LAB-11-V3-11Feb2013	77
Performing the Test-it Typhoid IgM (Life Assay)	SOP-WP2-LAB-12-V4-08Jan2014	82
Performing the Test-it Leptospirosis IgM (Life Assay)	SOP-WP2-LAB-13-V3-11Feb2013	86
Performing the Leptospira IgG/IgM (SD Bioline)	SOP-WP2-LAB-14-V3-11Feb2013	90
Blood : RDT HIV Determine	SOP-WP2-LAB-32-V1.1-03Dec2012	95
**Data Management SOPs**		
Completing CRFs<br>*+ 6 annexes (2 per country)*	SOP-WP6-DATA-01-V4-09Oct2013<br>*SOP-WP6-DATA-01-V3-30Jul2013_Annex1/2*	99
Procedures for data management (data entry)<br>*+ 6 annexes*	SOP-WP6-DATA-02-V2-04Mar2014<br>*SOP-WP6-DATA-02-V2-04Mar2014-Appendix1/2/3/4/5/6*	112
**Quality Control SOPs**		
Obtaining informed consent	SOP-WP6-DOC-01-V1-01Feb2012	133
Patient & sample numbering and labelling<br>*+ 2 annexes*	SOP-WP6-DOC-02-V2-21December2012<br>*SOP-WP6-DOC-02-V1-25June2012-annex1/2*	137
Management of study documents	SOP-WP6-DOC-03-V1-24May2012	146

SOP on SOP	SOP-WP6-QUAL-01-V1-02Nov2011	152
Monitoring study centre<br>*+ 1 annex*	SOP-WP6-QUAL-02-V2-05Dec2012<br>*SOP-WP6-QUAL-02-annex1*	156
Internal quality control activities<br>*+ 1 annex*	SOP-WP6-QUAL-03-V4-23Jun2014<br>*SOP-WP6-QUAL-03-V2-01Feb2013-annex1*	170
Laboratory supervision visit	SOP-WP6-QUAL-04-V01-23Feb2014	179
Storing and handling RDTs<br>*+ 2 annexes*	SOP-WP6-QUAL-05-V2-20Aug2013<br>*SOP-WP6-QUAL-05-V1-08Feb2013-annex1*<br>*SOP-WP6-QUAL-05-V2-20Aug2013-annex2*	187
How to install and use the « min-max » thermometer<br>*+ 4 annexes*	SOP-WP6-QUAL-06-V1.1-04Feb2013<br>*SOP-WP6-QUAL-06-V1.1-04Feb2013-annex1/2/3/4*	196
Stock Management<br>*+ 2 annexes*	SOP-WP6-QUAL-07-V1.1-04Feb2013<br>*SOP-WP6-QUAL-07-V1.1-04Feb2013-annex1/2*	203
Handling of expired and disqualified products<br>*+ 1 annex*	SOP-WP6-QUAL-08-V1-05Mar2014<br>*SOP-WP6-QUAL-08-V1-05Mar2014-annex1*	208

	**SOP Title:** Assessing Inclusion and Exclusion Criteria
	**Project/study:** Evaluation of Rapid Diagnostic Tests (RDT) in association with clinical and laboratory predictors for the diagnosis of Neglected Tropical Diseases (NTD) in patients presenting with persistent fever (≥1 week) in Cambodia, Nepal, Democratic Republic of the Congo and Sudan

1. Scope and application

This SOP applies to all the patients referred to the study investigator/research officer by the care provider in all study sites involved in the persistent fever component of the NIDIAG study (WP2). It describes the procedure for checking inclusion and exclusion criteria.

2. Responsibilities

Function	Activities
Study Investigator/Research Officer	Perform detailed history taking and perform physical examination of the patient.

3. Procedures

1. When a patient is referred or contacted by the care provider for consideration for enrollment the investigator will visit the patient in the respective area along with the CRF and consent forms.

2. Age of the patient, duration of the fever and whether the patient will be able to come to the hospital for follow up will be ascertained. Any person less than 5 years old or with fever for < 7 days or unable to come for the planned follow up in the hospital will not be enrolled.

3. A quick clinical assessment to assess respiratory distress and blood pressure (BP) will be measured. Those in respiratory distress or with low BP (Systolic BP < 90 mm in adults and < 60 mm Hg in children) will not be included in the study and the investigator will communicate this to the care provider.

4. The clinical notes and any laboratory reports of the patient during this illness will be studied. Patients who already have a confirmed diagnosis or whose fever has resolved will be excluded. However, if the patient is receiving (or has received) treatment with antimicrobials but the fever has not responded, he/she will be considered for inclusion.

5. A written informed consent will be obtained from the patient (or in the case of children, from a parent) before enrollment in the study (see procedure in SOP SOP-WP6-DOC-01-V1-01Feb2012); patients who are not willing or physically or mentally not able to give consent will not be included in the study.

   - If the patient is an adult (>18 yrs) she/he can give consent directly.
   - If the patient is an adolescent (between 12 to 18 years) take the consent from their parents/guardian. Also take the assent from the adolescent.
   - If the patient is < 12 years, take the consent from guardian/parent.

6. Take consent for investigation on HIV/AIDS.
7. Complete the *screening checklist for patient inclusion* that includes the following table:

**INCLUSION/EXCLUSION CRITERIA (to be filled by study physicians)**			
**INCLUSION CRITERIA:**		YES	NO
1	Fever ≥1 week	☐	☐
2	Age from 5 years onwards	☐	☐
**EXCLUSION CRITERIA**		YES	NO
1	Patients with an existing diagnosis established on the study criteria	☐	☐
2	Patients in need of immediate intensive care due to shock or respiratory distress	☐	☐
3	Patient unwilling or unable to comply with study requirements	☐	☐
4	Patient unwilling or unable to sign/thumb print the informed consent	☐	☐
**SELECTION FOR THE STUDY**		YES*	NO**
The patient meets all inclusion criteria and none of the exclusion criteria		☐	☐

4. Definitions

CRF= Case Report Form

5. Records and archives

**Appendices & Forms for completion**	
**Number**	**Title**
NA	

**Name and function**	**Date**	**Signature**
Author		
Suman Rijal	24/08/2012	
Reviewed by		
Tine Verdonck	19/09/2012	
Approved by		
François Chappuis	19/09/2012	

	**SOP Title:** Clinical examination at baseline and during follow-up
	**Project/study:** Evaluation of Rapid Diagnostic Tests (RDT) in association with clinical and laboratory predictors for the diagnosis of Neglected Tropical Diseases (NTD) in patients presenting with persistent fever (≥1 week) in Cambodia, Nepal, Democratic Republic of the Congo and Sudan

1. Scope and application

This SOP describes the baseline and follow-up clinical assessments to be applied to all patients who have fulfilled the inclusion and exclusion criteria and signed the informed consent for the persistent fever study (NIDIAG project, WP2).

2. Responsibilities

Function	Activities
Study Investigator/Research Officer	Taking history, physical examination of the patient, ordering the planned laboratory tests, recording information in the CRF, scheduling follow up visit, assessing patient during the follow up.
Lab. Technician or Nurse	Collecting biological samples.

3. Procedures

A. Day of admission or first outpatient consultation (Day 0)

1. Ensure that the criteria for enrollment are fulfilled (see SOP-WP2-CLIN-05) and informed consent has been obtained (see SOP-WP6-DOC-01).
2. Allocate a unique patient ID to the patient and write it in each page of the CRF.
   Patient Number ☐☐☐☐☐-☐☐ (see SOP-WP6-DOC-02 and SOP-WP6-DATA-01)
3. Take a detailed history, proceed with clinical examination and record this information directly in the CRF, in the “history taking day 0” and “physical examination day 0” sections.
4. Have all biological samples collected and sent to the laboratory for processing or storage (see SOP-WP2-LAB-02 and SOP-WP2-CLIN-07).

B. During hospitalisation or outpatient visits

5. Follow all patients daily during their hospital stay, or on each outpatient visit (timing decided by the care provider) if the patient is followed on an outpatient basis.
6. Record vital signs, **new** symptoms or signs and assess clinical evolution compared to baseline (improving, stable, deteriorating, death, left hospital against medical advice) in the “subsequent clinical assessment” section of the CRF.
7. Upon patient discharge (or at time of last follow-up visit), record vital signs, remaining symptoms and signs, and assess clinical evolution compared to baseline (resolved, resolved with sequelae,

not resolved-improving, not resolved-stable, not resolved-deteriorating, death, left hospital against medical advice) in the "study site discharge assessment" section of the CRF.

C. One month after study inclusion

8. Conduct a systematic follow-up assessment **one month** after the patient was included in the study. This follow-up visit will generally - but not necessarily (e.g. prolonged hospitalisation or treatment) - take place after the patient has been discharged from study site or after the last OPD visit.
9. Record vital signs, presence or absence of fever, new symptoms or signs since last clinical assessment, investigations done or treatment taken since last assessment, and clinical outcome in the "follow-up assessment (one-month post-inclusion)" section of the CRF.
10. Have blood collected in all patients to assess seroconversion. This sample must be stored for shipment to international reference laboratories (see SOP-WP2-LAB-02).

Additional notes

- Maintain a log book for the appointments and provide a patient card to all patients with the appointment dates, at day 0 and at each visit for outpatients and at time of discharge for inpatients.
- During initial, subsequent, discharge and one-month post-inclusion follow-up assessments, record all treatment administered in the "medication forms" at the end of the CRF.

4. Definitions

5. Records and archives

Appendices & Forms for completion	
**Document**	**Sections**
CRF	History taking day 0<br>Physical examination day 0<br>Subsequent clinical assessment<br>Study site discharge assessment<br>Follow-up assessment (one-month post inclusion)<br>Medication forms

Name and function	Date	Signature
Author		
Suman Rijal<br>Task 2.d. co-leader	12/11/2012	
Reviewed by		
Tine Verdonck	15/11/2012	
Approved by		
François Chappuis<br>WP2 leader	15/11/2012	

	**SOP Title:** Performing bone marrow aspirate
	**Project/study:** Evaluation of Rapid Diagnostic Tests (RDT) in association with clinical and laboratory predictors for the diagnosis of Neglected Tropical Diseases (NTD) in patients presenting with persistent fever (≥1 week) in Cambodia, Nepal, Democratic Republic of the Congo and Sudan

1. Scope and application

This SOP applies for parasitological diagnosis and bone marrow culture in patients suspected to have Visceral LeishmaniasisinTabarak Allah (Sudan), and BPKIHS and Koshi Zonal Hospital (Nepal). It describes the puncture and aspiration of bone marrow.

2. Responsibilities

Function	Activities
Study Investigator/Research Officer	Take informed consent, record vital parameters, perform the bone marrow aspiration, send the sample to the laboratory

3. Procedures

3.1 Precautions

Before the procedure it is important to know of any medical conditions that may complicate the procedure like disease or recent surgery involving the pelvic bone, active bleeding, severe cardiac or pulmonary disease, unusual sensitivity to pain, allergies to iodine or lidocaine.

3.2 Required materials

Isopropyl alcohol swabs
Betadine (povidone iodine 10%) solution
Sponge holder
Sterile gauge sponges 3 x 4 in.
Latex gloves, non-sterile
Latex gloves, sterile
LidocaineHCl, injection, 1%,
Needles: 22 gauge and 26 gauge
Disposable plastic sterile syringe: 10 ml (2)
Sterile dressing sheet, with 1 1/2" x 2" fenestration
Bone marrow aspiration needle, 15 gauge, 4"
Bandage scissors
Adhesive tape
Microscope slides, 1" x 3", frosted

3.3 Procedure

Preparation

1. Check that the indication for performing bone marrow aspiration is present, according to the national guidelines and the study protocol:
    - Fever for ≥14 days
    - Palpable splenomegaly
    - Negative lymph node aspirate for LeishmanDonovan bodies (in Sudan)
2. Check for contraindications for performing bone marrow aspiration: local site infection
3. Reassure the patient, describe the procedure in brief and take the informed consent.
4. Record pre-procedure blood pressure and pulse.
5. Check that all the materials are available for performing the bone marrow aspiration.

Puncture

1. Place the patient in prone position.
2. Remove clothes from the site of puncture.
3. Wash hands and wear non-sterilegloves.
4. Select the site at posterior superior iliac spine/most prominent bony part at the back (fig). If required make a mark with a ball point pen.

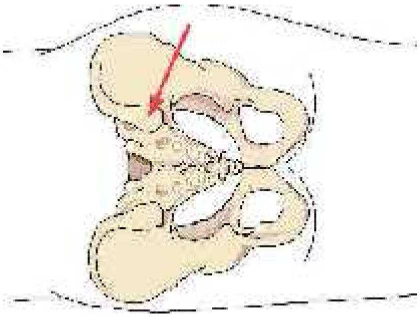

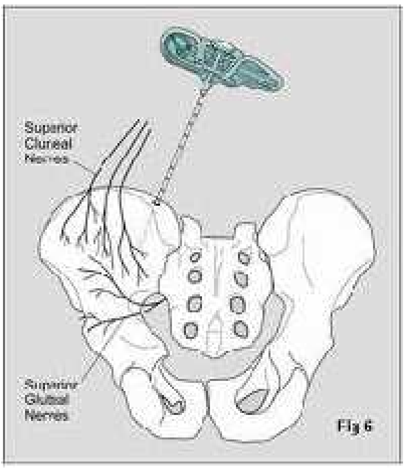

5. Wear sterile gloves and drape the area with sterile draping sheet
6. First swipe the area with isopropyl alcohol and let dry (minimal 30 seconds), next wipe with Betadine and wait for 2 minutes. Clean starting from center to periphery.
7. Take 10 ml of local anaesthesia (1% lidocaine) in a 10 ml syringe. Infiltrate the skin using 26 gauge needle. After a few minutes infiltrate the subcutaneous tissue into the periosteum.
8. Ensure that the periosteum is numb using the needle.
9. Push the bone marrow aspiration needle through the skin and subcutaneous tissue uptothe periosteum. Adjust the guard of needle so that only a further 5 mm can be advanced into the bone marrow.
10. Hold the needle at a right angle to the bone.
11. Exert firm pressure while turning the needle clockwise and counter clockwise: you will push through the outer cortex. When you feel a sensation of decreased resistance, you have entered the marrow cavity: do not push further.

Aspiration

1. Remove the stylet; attach the 10 ml syringe to the needle. With asharp suction, aspirateup to 1 ml of marrow into the syringe. Avoid mixing with peripheral blood. During aspiration, patient experiences an excruciating suction pain indicating that the needle is in the marrow.If no marrow is aspirated, rotate the needle or replace the stylet and cautiously advance or retract the needle advanced or retracted. If marrow is still unobtainable, chose another site for puncture.
2. Withdraw the syringe and give it to the assistant to prepare 3 slides immediately before coagulation. Then from the remaining amount use half for inoculation for Brucella/Salmonella culture and the other half for Leishmania culture. If less than 1 ml bone marrow is aspirated then omit the inoculation for Brucella/Salmonella.
3. Label the slide and the vials.
4. Withdraw the needle, pressthe puncture site and fix with sterile gauze.Apply adhesive tape over the gauze.
5. Discard the instrument and needle in an appropriate container.
6. Advise the patient to lie for 30 mins at site of aspiration.
7. Send the slides and the inoculated culture media to the Laboratory for staining and microscopy and incubation in the appropriate temperature respectively.
8. Record post procedure blood pressure and pulse.

4. Records and archives

**Appendices & Forms for completion**	
**Number**	**Title**
NA	LAB REPORT FORM

**Name and function**	**Date**	**Signature**
Author		
Suman Rijal, co-task leader	26/10/2012	
Reviewed by		
Tine Verdonck	19/09/2012	
Approved by		
François Chappuis, WP2 leader	05/11/2012	

	**SOP title :** Urine sampling
	Project/Study : NIDIAG

1. Scope and application

This document contains instructions on how to obtain a midstream sample of urine

2. Responsibilities

Function	Activities
Laboratory technician	• Obtains urine samples<br>• Follows the procedures

3. Procedures

3.1 Precautions

- All urine samples are potentially contagious. Respect the universal precautions. USE SINLGE-USE GLOVES DURING THE ENTIRE PROCEDURE!

3.2 Required material

- Single-use, plastic receptacle for urine
- Single-use, non-sterile gloves
- Marker

3.3 Procedure

- Put on single-use gloves
- Write the patient number, the date and the hour on the receptacle
- Give the receptacle to the patient
- Explain to the patient how to obtain a midstream urine specimen
- Ask the patient to produce a urine sample in a local rest room:
  - o Minimum 50 ml
  - o Midstream
- Use the urine within 4 hours after obtaining the sample

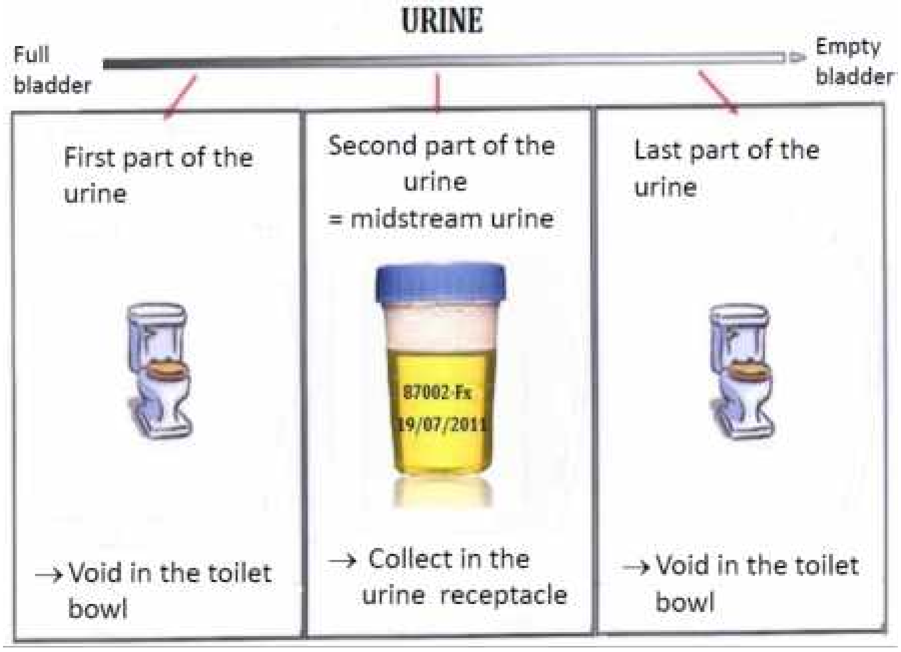

4. Records and archives

Appendices & forms to fill out	
**Number**	**Title**
	Not applicable

5. Document History

Revision	
SOP-WP2-LAB-36-V01-13Aug2012	Initial version
SOP-WP2-LAB-36-V01.1-13Aug2012	Translation from French to English

Name and function	Date	Signature
Author (Translation from French to English)		
Tine Verdonck	22/08/2012	
Reviewed by		
Approved by		
Veerle Lejon	22/08/2012	

	**SOP Title:** Blood sampling<br>(blood cultures, serum, whole blood on heparin and EDTA)
	Project/Study : NIDIAG

1. Scope and application

This procedure describes the blood sampling for blood cultures and by means of blood collection tubes and it explains the pre-treatment of the blood samples.

2. Responsibilities

Function	Activities
Laboratory technicians<br>Nurses	• Complies with this procedure<br>• Performs the blood collections<br>• Pre-treats the blood samples<br>• Labels the blood samples
Clinicians	• Requests the blood samples<br>• Supervises the blood collections

3. Procedures

3.1 Materials and consumables

1. Blood culture bottle (BactAlert) :
    - Adult (≥14 years) : 2 blood culture bottles with a green cap
    - Child (5 – 13 years): 1 blood culture bottle with a yellow cap
2. Examination gloves (single use – non sterile)
3. Tourniquet
4. Gauze (non sterile)
5. 70% Ethanol
6. 10% Povidone iodine
7. Butterfly needles (23G for adults – 21G for children) with Vacutainer adapter
8. 2 Vacutainer adapter caps for blood culture bottles (pre-cleaned with 70% ethanol)
9. Vacutainer tube holder (pre-cleaned with 70% ethanol)
10. Vacutainer serum tube
11. Vacutainer heparin tube
12. Vacutainer EDTA tube
13. Dry bandage
14. Sharps container

3.2 Procedure

3.2.1 General comments (see annex 1)

- Collect the blood for the blood cultures BEFORE any other sampling
- Collect the blood ALWAYS before antibiotics administration
    ➔ *For patients who are already under antibiotics treatment, perform the blood sampling before the next administration of antibiotics.*
- Blood collection:

<u>Adults</u> (≥ 14 years)

- 2 different sampling sites
- 2 blood culture bottles with green cap yellow cap
- 1 serum tube (9 ml)
- 1 heparin tube (4 ml)
- 1 EDTA tube (6 ml)

<u>Children</u> (5 – 13 years)

- 1 sampling site
- 1 blood culture bottle with
- 1 serum tube (5 ml)
- 1 heparin tube (4 ml)
- 1 EDTA tube (6 ml)

- **NEVER RECAP NEEDLES !**

3.2.2 Blood collection procedure

1. Prepare all the necessary materials and inform the patient.
2. Clean your hands with alcohol gel or wash them with water and soap.
3. Disinfect the skin at the blood collection site with 70% ethanol. Begin by disinfecting the selected puncture site and then continue in a circular motion (outwards).
4. Let dry for 1 minute.
5. Disinfect the skin at the blood collection site with 10% povidone iodine in the same manner.
6. Let dry for 2 minutes. In the meantime, continue with steps 7-13.
   DO NOT touch the puncture site from this moment onwards!!
7. Label the blood culture bottle(s) and the blood collection tube(s).
8. Put the blood culture bottle(s) in a rack and remove the cap(s).
   Child: Blood culture bottle with yellow cap (1x)
   Adult: Blood culture bottle with green cap (2x)
9. Disinfect the rubber stopper of the blood culture bottle with 10% povidone idone.
10. Let dry for 1 minute.
11. Put on gloves.
12. Connect the butterfly needle to the Vacutainer adaptor cap for blood culture bottles. Screw until tightened.
13. Place the rack with the blood culture bottle(s) on a support (table) close to the blood collection site.
14. Apply the tourniquet.
15. Puncture the selected vein with the blood collection device.
16. Place the adaptor cap on the blood culture bottle, push on the adaptor cap to introduce the needle in the rubber stopper. **Remove the tourniquet once the blood starts to flow.**
17. Let the blood enter the bottle up to a volume of 1 line of 4 ml (for children) or 2 lines of 5 ml (for adults).

Child (5 – 13 years)	4 ml (1 line)
Adult (≥ 14 years)	10 ml (2 lines)

If you do not succeed in collecting the blood, repeat the procedure with a new blood collection device.

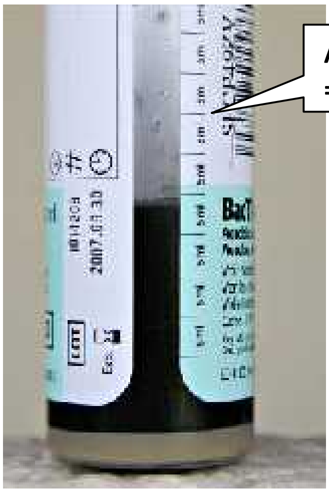

Adult : Blood: 10 ml
= 2 lines of 5 ml

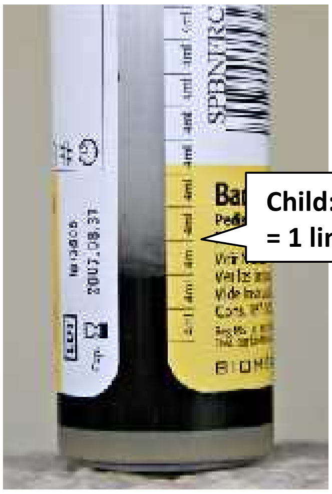

Child: Blood: 4 ml
= 1 line of 4 ml

18. Remove the blood culture bottle and place the Vacutainer tube holder.
19. Insert the Vacutainer serum tube in the tube holder and collect the blood for the serum tube.

Only for children (5 – 13 years):

20. Remove the serum tube and replace it with a heparin tube (4 ml tube).
21. Remove the heparin tube and replace it with an EDTA tube (6 ml tube).
22. Remove the EDTA tube.

For all patients :

23. Remove the butterfly needle from the vein.
24. Disconnect the butterfly needle from the Vacutainer adapter cap.
25. Dispose the butterfly needle and tube in a sharps container.
26. Put a dry bandage on the puncture site.
27. Peel off the barcode of the blood culture bottle and paste it on the blood culture collection form.

Only for adults (≥ 14 years) :

Repeat the same procedure on the second arm (steps 3-18). *Use a new blood collection system (butterfly needle, Vacutainer adapter cap and Vacutainer tube holder), then:*

1. Insert the Vacutainer heparin tube in the tube holder and collect the blood for the heparin tube.
2. Remove the heparin tube and replace it with an EDTA tube.
3. Remove the EDTA tube.
4. Remove the butterfly needle from the vein.
5. Disconnect the butterfly needle from the Vacutainer adapter cap.
6. Dispose the butterfly needle and tube in a sharps container.
7. Put a dry bandage on the puncture site.
8. Peel off the barcode of the blood culture bottle and paste it on the blood culture collection form.
9. Disinfect the adaptor cap with 70% ethanol.

3.2.3 Pre-treatment of blood samples

See document SOP-WP2-LAB-37 (list of tests).

- Store the blood culture bottles in an incubator at 37°C until shipment.
- The heparin and EDTA tubes are ready to be analyzed.
  If tests cannot be performed immediately, whole blood can generally be stored for 3 days at 2-8°C. Check specific test recommendations on sample storage and handling.
- The serum tube needs to be centrifuged before analysis:
  1. Let stand the Vacutainer serum tube for 10 minutes.
  2. Centrifuge the tube for 10 minutes at 3000 rpm.

  Check specific test recommendations on sample storage and handling.

4. Records and archives

Appendices & forms for completion	
**Number**	**Title**
1	CRF laboratory

5. Document History

Revision	
SOP-WP2-LAB-44-V01-18Sep2012	Initial version
SOP-WP2-LAB-44-V1.1-27Dec2012	Translation in English

Name and function	Date	Signature
Author		
Barbara Barbé	27/12/2012	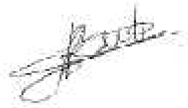
Reviewed by		
Philippe Gillet	28/12/2012	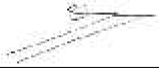
Approved by		
Emilie Alirol	02/01/2013	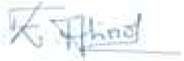

	**SOP Title:** Collection of lymph node (LN) aspirates
	**Study title:** Evaluation of Rapid Diagnostic Tests (RDT) in association with clinical and laboratory predictors for the diagnosis of Neglected Tropical Diseases (NTD) in patients presenting with persistent fever (≥1 week) in Cambodia, Nepal, Democratic Republic of the Congo and Sudan

1. Scope and application

Lymph node aspiration (LNA) is a useful tool in establishing parasitologic diagnosis of visceral leishmaniasis (VL) in Sudan where the high prevalence of generalized lymphadenopathy is a characteristic feature of the disease. The procedure is least invasive, simple and is performed in < 1 minute without local anesthesia. LN aspirates can then be used for the detection of *Leishmania donovani* amastigotes (LD bodies) in stained smears and for invitro culture for the detection of promastigotes (SOP-WP2-LAB-15). This SOP describes how to perform LNA. It is applicable to the parasitological diagnosis of visceral leishmaniasis in the patients enrolled in the persistent fever syndrome of NIDIAG study in Sudan.

2. Responsibilities

Function	Activities
Laboratory Technician	1. Perform the lymph node aspiration.<br>2. Prepare, stain and examine by microscopy smears of LN aspirate<br>3. Report results in the laboratory register and in the Lab Report Form of the CRF

3. Procedures

3.1 Aspiration sites

The palpable inguinal and epitrochlear lymph nodes are most convenient for the procedure.

3.2 Materials required

- 21G needle
- 5ml syringe
- Lead or diamond pencil,
- Clean (new) microscope slides
- Clean piece of gauze
- Cotton wool
- Disinfectant (iodine or 70% alcohol)
- Bed for the patient (screened off area for privacy with good lighting)
- Waste containers

3.3 Aspiration procedures

- Label a slide with the patients' lab number (use lead or diamond pencil)
- Clean the slide with gauze
- Rest the patient on the back with the legs stretched out
- Inform the patient the the procedure is slightly painful and that he/she should keep still
- Ask another person to hold the legs if the patient is restless (If it is a small child, the mother can hold him/her on her lap)
- Disinfect the skin over the gland with gloved hands using cotton wool soaked in 70% alcohol or iodine and leave to dry
- Hold the gland between the thumb and index finger of your left hand (see picture):

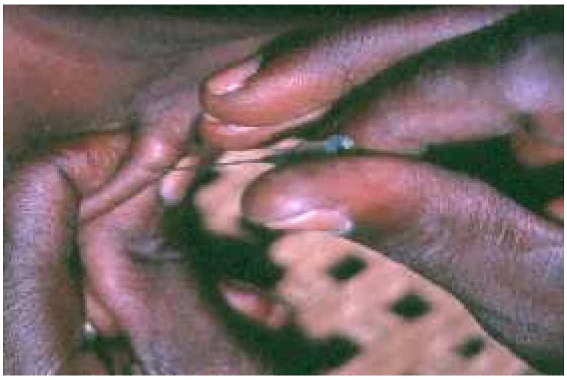

- Insert a sterile needle (*21G*) into the centre of the gland at right angles to the skin
- Avoid adjacent blood vessels
- Gently squeeze the gland with the left hand while rotating the needle in the right hand. Glandular fluid will come up the bore of the needle
- Place the index finger over the end of the needle and withdraw it rapidly
- Apply a swab with disinfectant over the site
- Draw back the piston of a new 5ml syringe
- Attach the needle to the syringe and discharge the fluid/aspirated material onto the slide by pushing the syringe piston
- Spread the fluid with the needle to make a thin smear on the slide with one quick motion

3.4 Fixation, staining, microscopy and grading of parasite load (refer to SOP-WP2-LAB-15)

4. Records and archives

Appendices and forms to complete	
**Number**	**Title**
NA	NA

5. References

Kirk R, Sati MH, 1940. Studies in leishmaniasis in the Anglo-Egyptian Sudan II. The skin and lymph glands in kala-azar. *Trans R Soc Trop Med Hyg 33:* 501–506.

Zijlstra EE, El-Hassan AM, 2001. Leishmaniasis in Sudan: visceral leishmaniasis. *Trans R Soc Trop Med Hyg 95 (Suppl 1):* S27–S58.

Babiker ZO, Davidson R, Mazinda C, Kipngetich S and Ritmeijer. Utility of lymph node aspiration in the diagnosis of visceral leishmaniasis in Sudan. *Am. J. Trop. Med. Hyg.,* 76(4), 2007, pp. 689–693.

6. Document History

**Revision**	
	Initial version

**Name and function**	**Date**	**Signature**
Author		
Sayda El Safi	19/11/2012	
Review by		
François Chappuis	26/11/2012	
Approved by		
Emilie Alirol	07/12/2012	

	**SOP Title:** Preparation of thick blood film, Giemsa staining and microscopic examination
	**Project/Study**: NIDIAG

1. Scope and application

This document contains instructions on how to prepare a thick smear, make a Giemsa stain and do the microscopic examination.
In this study, the thick smear is used for the detection of malaria (Plasmodium spp.), recurrent fever (Borrelia spp.) and sleeping sickness (Trypanosoma spp.).

2. Responsibilities

Function	Activities
Laboratory technician	• Takes the blood<br>• Does the test<br>• Prepares the dilution of Giemsa and checks the pH.<br>• Correctly stores concentrated and diluted Giemsa<br>• Interprets the result<br>• Records the results<br>• Maintains the microscope

3. Procedures

3.1 Precautions

- All blood samples are potentially contagious. Respect the universal precautions. USE SINGLE-USE GLOVES DURING THE ENTIRE PROCEDURE!
- Giemsa is inflammable; manipulate this product far from any flame.

3.2 Material and samples

3.2.1 Required material

- Single-use, non-sterile gloves
- New microscopic glass slides
- Concentrated Giemsa (Merck N° 1.09204)
- pH 7.2 tablets (Merck N° 9468)
- pH-meter and calibration solution
- Bottle of 1000 ml
- Clean water or tap water
- Dropper bottle
- Pasteur pipettes
- 10 ml graduated cylinder
- 200 ml beaker
- Support for staining

- Rack for slides
- Timer
- Microscope (objective 100 x)
- Immersion oil
- 2 touch counters
- Storage box for slides
- Tissue

3.2.2 Sample to be examined

- Anti-coagulated blood taken on EDTA : ± 10 µl
- Stability : maximum one hour at room temperature (to avoid morphological deformations of Plasmodium parasites)

3.3 Operating procedures

3.3.1 Preparation of the thick smear

1. Submerge the new slides in denatured alcohol for 30 minutes and dry them with a clean tissue.
2. Put on the single-use, non-sterile gloves.
3. Write the patient code on the glass slide.
4. Put a small drop of blood (±10 µl) on the middle of the slide using a Pasteur pipette.
5. Mix the blood immediately during 20 – 30 seconds with the corner of another slide.
6. Smear the blood on an area of 1 to 2 cm diameter.
   Good thickness of smear = when it is possible to read the letters of a text through the thick smear.
7. Leave the slide to dry on a flat surface WITHOUT HEATING and WITHOUT EXPOSURE TO THE SUN to avoid fixation of red blood cells which would prevent haemolysis.

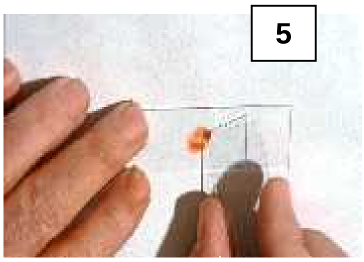

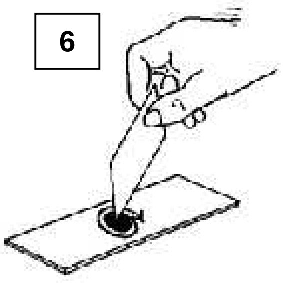

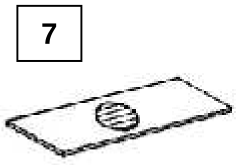

3.3.2 Staining of the thick smear with Giemsa 3,5 % stain

3.3.2.1 Preparation of the buffer solution

- Dissolve a tablet of pH 7,2 in 1 litre of clean water.
- Check the pH of the prepared solution (see Procedure pH-meter). The pH should be between 7,0 and 7,4.
- Write « Buffer, the preparation date, and the expiry date » on the bottle.

➔ *Stability*: one month at room temperature. Replace the solution if it is slightly turbid after agitation.

3.3.2.2 Preparation of Giemsa diluted at 3,5 % (prepare just before the staining)

- Pour a quantity of concentrated Giemsa that is sufficient for approximately one week of work in a small dropper bottle. Use this bottle to make fresh dilutions of Giemsa at 3,5% before each staining.

- Prepare a quantity of diluted Giemsa that is sufficient for the number of slides that have to be stained. Per slide:
    - Pour 4 ml of the buffer solution in a graduated cylinder of 10 ml
    - Add 5 drops of concentrated Giemsa
    - Mix together well
    - ➔ *Stability:* one hour at room temperature.

3.3.2.3 Staining of the thick smear with Giemsa 3,5 % stain

1. Wait until the thick smear is completely dry before staining (± 20 minutes).
2. Put the slide on a staining support. The slides must not be touched. Note: Do not use a basin for staining to avoid contamination between slides.
3. Dilute the necessary quantity of Giemsa (see « 3.3.2.2 Preparation of Giemsa diluted at 3,5% »).
4. Cover each slide completely with Giemsa diluted at 3,5%.
5. Let the stain act for 20 minutes.
6. Let the stain drain from the slide.
7. Gently rinse each slide in a beaker containing clean water.
8. Let the thick smear dry on a slide rack.

3.3.3 Microscopic examination of the thick smear

3.3.3.1 Detection of parasites/bacteria in blood

- Take off the gloves to do the microscopic examination (objective 100x, with immersion oil).
- Pass through the slide field by field and count the number of examined fields
- After 100 fields without detecting one parasite or one bacterium → report as negative examination.
- If a parasite or bacterium is found:
    - Identify the species (See « 3.3.3.2 Species identification ») and record the species in the laboratory notebook.
    - In the case of *Plasmodium spp.*, report also the stages of the parasites present in the thick smear (trophozoites, schizonts, gametocytes).
    - In the case of *Plasmodium spp.* → determine parasite density (See « 3.3.3.3 Parasite density »).

Blood element	Colour after Giemsa stain
Red blood cells	Haemolysed (not visible)
White blood cells	Magenta (pink-purple)
Platelets	Pale pink
Cytoplasm of *Plasmodium spp.*	Blue – mauve
Chromatin of *Plasmodium spp.*	Dark red – purple
Schüffner's stippling	Purple – red
Malaria pigment (hemozoin)	Yellow/brown or black
Borrelia spp.	Blue
Cytoplasm of *Trypanosoma spp.*	Blue – mauve
Nucleus and kinetoplast of *Trypanosoma spp.*	Red – purple
Undulating membrane and flagellum of *Trypanosoma spp.*	Bluish

3.3.3.2 Species identification

- <u>*Plasmodium spp.*</u>

The most important characteristics of each *Plasmodium* species:

P. falciparum		***P. vivax***	
– Uniform image<br>– Trophozoites: thin, ring-shaped, 1-2 grains of chromatin<br>– No schizonts (with the exception of severe cases)<br>– Gametocytes : shape of banana	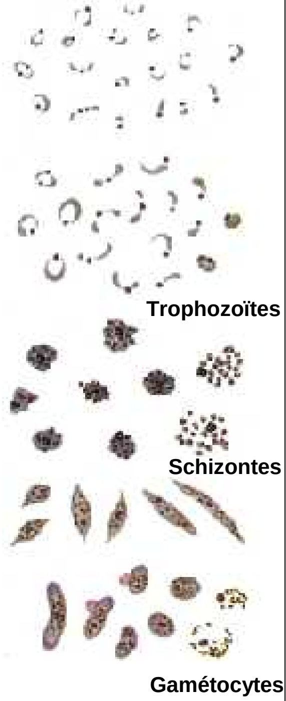	– All stages<br>– Amoeboid form (old trophozoites)<br>– Mature schizonts ≥16 nuclei<br>– Shadows of red blood cells (fine Schüffner dots)	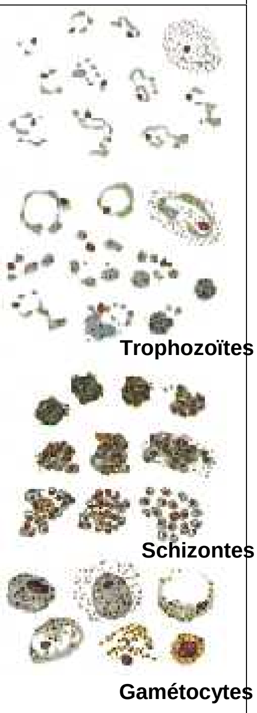

P. ovale		***P. malariae***	
– All stages<br>– 1 prominent chromatin dot<br>– Compact parasites<br>– Mature schizonts with 8 nuclei<br>– Shadows of red blood cells (Schüffner's dots)	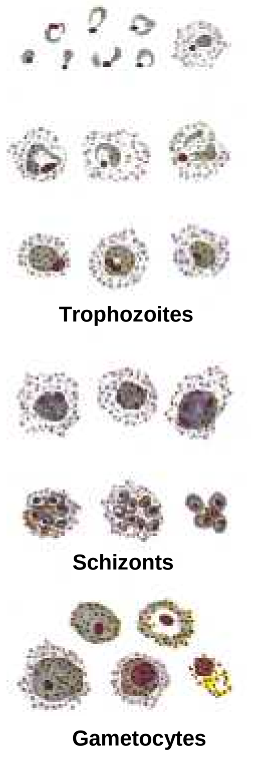	– All stages<br>– Small, compact, dark parasites<br>– Mature schizonts with 6-12 nuclei (form of a daisy)<br>– Early malaria pigment even in young trophozoites	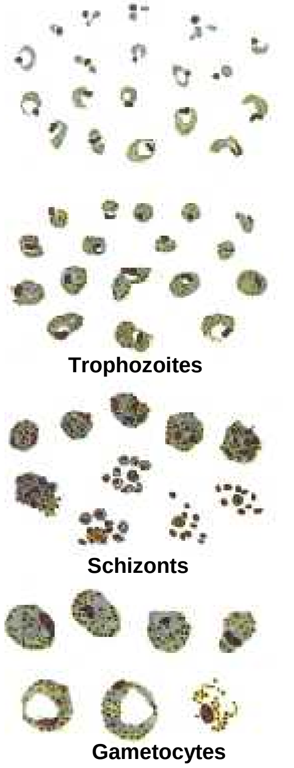

➔ Consult the WHO bench aid plates for the diagnosis of malaria!

➔ Record the species and the stages present in the thick smear in the laboratory notebook.

The features of severe malaria (*P. falciparum*):

- Hyperparasitaemia: >250.000 parasites/µl blood (endemic area)
- Presence of schizonts in circulating blood (and hence in thick smear)
- Predominance of mature trophozoites (malaria pigment present in >20% of trophozoites)
- Phagocytised hemozoin in white blood cells

➔ Record the features of severe malaria on the laboratory CRF and immediately notify the attending physician.

Borrelia spp. in thick smear

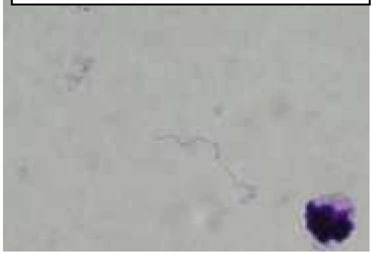

- <u>*Borrelia spp.*</u>
  - Gram-negative bacterium (spirochete)
  - Fine and long : 10-20 µm x 0,5 µm
  - Helicoid shape
  - Blue colour (with Giemsa)

- <u>*Trypanosoma spp.*</u>
  - *Trypanosoma brucei gambiense*: West and Central Africa
    Trypanosoma brucei rhodesiense: East Africa
  - 4 out of the 5 following characteristics must be present:
    - Size: between 15 and 25 µm
    - Cytoplasm : fusiform, bluish
    - Nucleus: rather big, reddish
    - Kinetoplast: small and usually central
      Reddish colour
    - Undulating membrane: of blue colour,
      Starts from the kinetoplast (sub-terminal) and extends up to the flagellum
  - Dimorphic: long forms (multiplying) and short forms (infective for the vector)
  - No morphological differences between the two species (geographic difference)

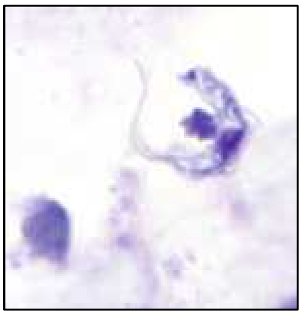

Trypanosoma spp. in thick smear

3.3.3.3 Parasite density *(Plasmodium spp.)*

- Take 2 touch counters (one in each hand)
- Pass through the slide field by field (objective 100x)
- Count ± 200 white blood cells and count at the same time the asexual *Plasmodium* parasites. Do not count the *P. falciparum* gametocytes (=sexual parasites).
  - If you do not find one parasite in 100 fields → the result is negative.
  - If you find > 100 parasites per field → count 5 fields and calculate parasite density
  - If you have counted 200 white blood cells and you have found
    ≥ 100 parasites → calculate parasite density
    < 100 parasites → continue counting up to 500 white blood cells and calculate the parasite density
- Use the following formula to calculate parasite density:

$$\frac{\text{Count of asexual parasites}}{\text{White blood cell count}} \times \text{concentration of white blood cells} = \text{Number of parasites/µl blood}$$

➔ If the white blood cell count has not been done, use 8000 white blood cells/µl blood.

- Note: 50.000 parasites per µl blood correspond with 1% of parasitized red blood cells.

3.4 Storage and conservation of slides for rereading

- Gently put the slides on a piece of toilet paper to eliminate the immersion oil.
- Store the clean slides in slide storage boxes and label the boxes clearly (study name, box number, type of sample and stain, number of first and last patients).

4. Definitions and abbreviations

- EDTA: ethylene diamine tetra-acetic acid
- spp.: species

5. Records and archives

**Appendices & forms to fill out**	
**Number**	**Title**
1	CRF "laboratory"

6. Document history

**Revision**	
SOP-WP2-LAB-20-V01-13Aug2012	Initial version
SOP-WP2-LAB-20-V01.1-22Aug2012	Translation from French to English

**Name and function**	**Date**	**Signature**
Author (Translation from French to English)		
Tine Verdonck	22/08/2012	
Reviewed by		
Approved by		
Veerle Lejon	22/08/2012	

	**SOP Title:** Storage of bacterial isolates
	Project/Study: Evaluation of Rapid Diagnostic Tests (RDT) in association with clinical and laboratory predictors for the diagnosis of Neglected Tropical Diseases (NTD) in patients presenting with persistent fever (≥1 week) in Cambodia, Nepal, Democratic Republic of the Congo and Sudan

1. Scope and application

This SOP describes the storage of bacterial isolates obtained from different types of specimens on Tryptic Soy Agar (TSA) or in the Microbank (Pro-Lab Diagnostics) in order to maintain them viable and free of contamination. This SOP also describes the frequency of shipment of these isolates to the Institute of Tropical Medicine in Antwerp, Belgium.

2. Responsibilities

Function	Activities
Laboratory technician	- safe handling of samples once arrived in the laboratory<br>- complies with the SOPs on isolation and identification<br>- documentation of all results obtained<br>- safe storage of all isolates by complying with this SOP<br>- correct disposal of any waste materials derived from the procedure
Laboratory technician trained in shipment of infectious substances	- proper shipment of the bacterial strains (Infectious substances, category B) according to WHO guidelines.

3. Safety

- Practice standard safety precautions for handling and disposing of infectious materials.

4. Precautions

- Do not store strains longer than 1 year on Tryptic Soy Agar (TSA).
  If longer storage is needed, re-culture the strain and store in fresh TSA medium.
- Ensure that the strain you will store is pure and fresh (< 24 hours)!
  - Your isolate is not pure if you observe any of the following,:
    - differences in hemolytic behavior
    - differences in size
    - differences in color
  - If this is the case, subculture each type of colony onto a separate blood agar plate (purity plate) and perform identification of each isolate.
  - If you observe mixed growth, identify all different colonies before storage.
- Sterile working is essential throughout the procedures!
  - Disinfect your hands with alcohol gel (70% ethanol) before starting the work.
  - Sterilize the stab needle / loop before each manipulation with the flame of a Bunsen burner.
  - Use sterile swabs for the Microbank (Pro-Lab Diagnostics) procedure.

- Sterilize the opening of the cryotube/vial whenever necessary with the flame of a Bunsen burner.
- In contrast to the manufacturer's user manual we do not recommend to remove the cryopreservative from the Microbank (Pro-Lab Diagnostics) vials before freezing.

5. Procedures

Prepare **2 TSA cryotubes** and **2 Microbank vials** for **EACH bacterial isolate**, that is isolated during the Nidiag study!

5.1 Storage of bacterial isolates on TSA

5.1.1 Materials

- Dehydrated TSA powder (for example OXOID, ref. CM0131 or any other reliable company)
- Distilled water
- 2ml cryotubes, with screw cap, autoclavable.
- Box that can contain tubes, autoclavable
- Pipette or dispenser to deliver approximately 1.5ml
- *Staphylococcus aureus* ATCC 25923 (control strain)
- Pure colony on agar
- Sterile stab needle or small inoculation loop
- Incubator, 35-37°C, aerobic
- Study specimens log (SOP-WP6-DOC-02-annex2)
- Site specific QC result form
- Site specific freezer inventory form

5.1.2 Procedures

5.1.2.1 Preparation of TSA tubes

1. Ensure that none of the used material is expired.
2. Follow manufacturers' instructions for preparation of Tryptic Soy Agar (TSA) as indicated on the bottle.
    a. Weigh correct amount of TSA powder as stated by the manufacturer.
    b. Dissolve powder in distilled water. Use a magnetic stirrer if available.
    c. Bring to boil.
    d. Cool down in water bath, ensure that media is still liquid.
    e. Unscrew a cryotube.
    f. Add approximately 1.5ml of the prepared TSA to the tube with a pipette or dispenser.
    Comment: When preparing large quantities of TSA, continue stirring the TSA during the filling process.
    g. Close the cryotube by screwing on the cap.
    h. Loosen the cap for autoclaving by turning it half a tour back.
    i. Repeat step e to h until the desired number of cryotubes are prepared.
3. Place all filled TSA cryotubes into a box
4. Autoclave for 15 minutes at 121°C
5. Let cool down inside the autoclave
6. When cooled down, close the cap of each tube firmly, to prevent drying out of the TSA medium.
7. Store in the dark at cool temperatures (2-8°C)
8. Perform QC (quality control) **for each batch of TSA cryotubes prepared** (see section 5.1.2.2).
9. **Do NOT use** the TSA cryotubes if the QC result is not good!

5.1.2.2 Quality control of TSA cryotubes

1. Select 2 TSA cryotubes that were prepared on the same day.
   - Label TSA cryotube 1 with 'QC positive' and the date.
   - Label TSA cryotube 2 with 'QC negative' and the date.
2. Inoculate the control strain *Staphylococcus aureus* ATCC 25923 to the tube labeled 'QC positive' (see section 5.1.2.3). This is your positive control.
3. Do not open the tube labeled 'QC negative', this is your negative control
4. Loosen the caps of both cryotubes a little bit.
5. Incubate both TSA cryotubes at 35°C ±2°C, aerobic, overnight.
6. After incubation, tightly tighten the screw cap.
7. Store the TSA cryotubes at room temperature at the assigned position.
8. 5 days after inoculation streak out an aliquot of the positive and negative control on a non-selective medium:
   a. Add an aliquot of TSA to the first quadrant of the non-selective agar plate
   b. Flame sterilize a loop
   c. Spread the well evenly on the first quadrant of the plate, as shown in step 1 (Figure 1)
   d. Flame sterilize the loop
   e. Spread the inoculum as shown in step 2 (Figure 2)
   f. Repeat procedure for step 3 and 4 (Figure 1)
   g. Heat sterilize loop between each step
   h. Ensure that the loop has cooled down before touching any colonies

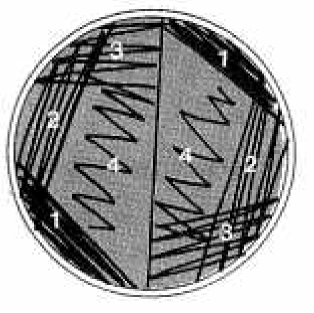

Figure 1:
Plating two isolates on one plate

9. Incubate at 35°C ±2°C, aerobic
10. Check for growth after 12-18h
11. Based on the result do the following:

	QC positive	QC negative	Action
**Result**	Growth	No growth	You can use the prepared TSA
	No growth	No growth	The prepared TSA does not support growth of bacterial isolates, you can't use the prepared media for strain storage
	Growth	Growth	Check with your supervisor:<br>• The non-selective media may not be sterile<br>• You may have introduced contamination during plating

12. Record on the QC result form

5.1.2.3 Storage of bacterial isolate on TSA

1. Label **2 TSA cryotubes per bacterial isolate** with the study specimen number (SOP-WP6-DOC-02) and the date of inoculation.
2. Fill in the study specimens log, **record the identity of the bacterial isolate and its position in the freezer** under the column "comments".
3. Select one pure colony on agar with a sterilized stab needle / loop.
4. Open the first TSA cryotube and flame the opening of the cryotube.
5. Stab the TSA media with the stab needle / loop carrying the colony and retract the needle.

6. Flame the opening of the TSA cryotube and close with the screw cap. Close the cryotube loosely with the screw cap.
7. Repeat step 3 – 6 for the second TSA cryotube for the same bacterial isolate.
8. Incubate the TSA cryotubes at 35ºC ±2ºC, aerobic, overnight.
9. After incubation, tightly tighten the screw cap.
10. Store the TSA cryotubes at room temperature at the assigned position.
    Comment: The TSA cryotubes can be stored for 1 year. If longer storage is needed, re-culture the strain and store in fresh TSA medium.

5.2 Storage of bacterial isolates in the Microbank (Pro-Lab Diagnostics)

5.2.1 Materials

- Pen, with freeze-resistant ink
- Sterile cotton swabs
- Microbank vials containing cryopreservative and cryobeads
  (Pro-Lab Diagnostics: ref. PLB170B, Microbank - 80 Blue colour coded beads)
- Pure colonies on agar
- Freezer inventory/study specimens log (SOP-WP6-DOC-02-annex2)
- Sterile loop / sterile forceps
- Enrichment media or agar
- Incubator, 35-37°C, aerobic

5.2.2 Procedures

5.2.2.1 Freezing of Microbank vials

1. Clearly label **2 Microbank vials per bacterial isolate** with the study specimen number (SOP-WP6-DOC-02) and date of inoculation. Use a pen with freezer-resistant ink.
   Comment: If you use a paper label, cover the entire label with scotch tape after labeling.
2. Record the storage position and identity of the isolated strain on the freezer inventory.
3. Collect as much growth from the agar plate as possible by using a sterile cotton swab.
4. Insert the swab into the Microbank vial: insert the swab into the cryopreservative and gently penetrate the cryobeads until you have reached the bottom of the vial.
5. Swirl a couple of times up and down with the swab.
6. Gently retract the swab.
7. Remove all remaining fluid from the swab by gently pressing it against the vial wall above the fluid surface and rotate it around the lumen of the vial.
   Comment: You should now have a heavy bacterial suspension of a 3-4 McFarland standard.
8. Close the lid of the vial sufficiently to contain the content without leakage. Do NOT close it with force.
   Comment: Ensure that the rubber ring of the lid is free of liquid, to prevent damage during freezing.
9. Invert the vial 4-5 times. Do NOT vortex the vial!
10. Let the inoculated vial stand for at least two minutes at room temperature.
11. Repeat step 2 – 10 for the second Microbank vial.
12. Store the Microbank vial at the assigned position at -70ºC Celsius (see freezer inventory log).
    Comment: While not recommended, you can store strains for a limited time (6 months) at -20ºC. However, storage at -20ºC is not recommended for *Streptococcus pneumoniae* and *Haemophilus influenza.*
13. Fill in the study specimen log, **record the identity of the bacterial isolate and its position in the freezer** under the column "comments".

5.2.2.2 Recovery of frozen bacterial isolates from Microbank vials

1. Wear protective gear when handling material from the -70°C freezer. Extreme cold can cause severe and painful burns.

2. Select the isolate you want to recover, and search the correct position of its Microbank vial in the freezer inventory.
3. Remove the respective box from the -70°C freezer.
   Comment: Limit the time of the box outside the freezer to an absolute minimum to avoid defrosting the entire collection.
4. Keep the Microbank vial in an isolation box.
5. Open the Microbank vial aseptically.
   6. Remove an aliquot of the cryopreservative with a sterile loop and close the vial.
   7. Streak the aliquot onto non-selective agar (see 5.1.2.2, Figure 1) or insert into enrichment media.

   OR

   6. Remove a single cryobead from the vial with a sterile forceps or needle loop and close the vial.
   7. Streak the cryobead onto non-selective agar (see 5.1.2.2, Figure 1) or insert into enrichment media.

   Comment: Do not re-insert any material that may have fallen out of the tube.
8. Refreeze the Microbank vial.
9. Incubate agar / enrichment media at 35°C ±2°C, aerobic, overnight.

5.3 Shipment of bacterial isolates

Responsibility of the lab technician that is trained in the shipment of infectious substances:

- Ship the bacterial isolates, stored on TSA or in the Microbank to ITM, Antwerp, once **a year**:
  - Ship TSA tubes at room temperature.
  - Ship Microbank vials (in boxes) on dry ice or in liquid nitrogen.
- Select a courier company that can ship the isolates in the correct conditions.
- Follow the packaging requirements of a category B infectious substance (WHO, Guidance on regulations for the transport of infectious substances, 2009-2010): triple packaging, UN number 3373, proper shipper and receiver name, etc.
- Record the type of samples, the date of shipment and the receiver on the study specimen log.
- Add a packaging list with the content of the box (= overview of all bacterial isolates to be shipped).

6. References

- J. Vandepitte et al. Basic Laboratory Procedures in Clinical Bacteriology. Second Edition World Health Organization 2003.
- Pro-Lab Diagnostics, Revision: 2005 06, Microbank, Product information.
- WHO, Guidance on regulations for the transport of infectious substances, 2009-2010 *http://whqlibdoc.who.int/hq/2008/WHO_HSE_EPR_2008.10.pdf*

7. Records and archives

Appendices and forms to complete	
**Number**	**Title**
SOP-WP6-DOC-02-annex2	Study specimens log
	Site specific QC result form
	Site specific Freezer inventory

8. Document History

Revision	
SOP-WP6-LAB-03-14Mar2013	Initial version

Name and function	Date	Signature
Author		
Barbara Barbé	29/03/2013	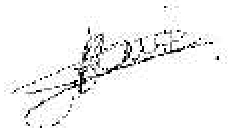
Reviewed by		
Jan Jacobs	02/04/2013	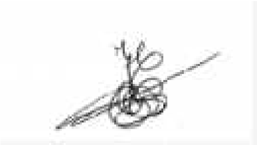
Approved by		
Emilie Alirol	15/04/2013	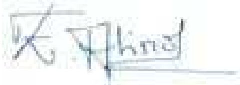

	**Titre de la SOP :** Blood : RDT malaria SD Bioline 60
	Project/Study: Evaluation of Rapid Diagnostic Tests (RDT) in association with clinical and laboratory predictors for the diagnosis of Neglected Tropical Diseases (NTD) in patients presenting with persistent fever (≥1 week) in Cambodia, Nepal, Democratic Republic of the Congo and Sudan.

1. Scope and application

This document provides the instructions to execute the Malaria rapid diagnostic test (RDT) SD 05FK60. This RDT detects antigens specific to *Plasmodium falciparum* HRP-II (Protéine II, histidine-rich) and to all the *Plasmodium* species (Pan-pLDH: Parasite Lactate DeHydrogenase): *P. falciparum, P. vivax, P. ovale* and *P. malariae*. This test constitutes a valuable tool to diagnose malaria. Be careful : This test does not always allow differentiation between infection with *Plasmodium falciparum* and a mixed infection (*P. falciparum* associated with one or more other Plasmodium species).

2. Responsibilities

**Function**	**Activities**
Laboratory technician	• Blood collection<br>• Performing the test<br>• Interpretation of the test result<br>• Registration of the test result

3. Procedures

3.1 Precautions

- All blood samples are potentially infectious. Please respect universal precautions. USE DISPOSABLE GLOVES DURINGTHE WHOLE PROCEDURE!
- Do not use the kit beyond expiry date.
- If the package is damaged, use another test device.
- Use the test device immediately after it is opened.
- Do not reuse the test device.

3.2 Material and samples

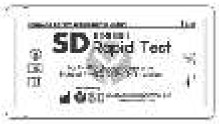

3.2.1. Material provided in the kit and storage

- The cassette SD BIOLINE Ag Pf / Pan :
  One strip of tests contains:
  - The conjugate : Monoclonal mice antibodies specific to P.f. HRP-2 – colloide gold
    Polyclonal mice antibodies specific to pLDH - colloide gold
  - Test line P.f. : Monoclonal mice antibodies specific to P.f. HRP-II
  - Test line Pan : Polyclonal mice antibodies specific to pan-pLDH
  - Control test line : Goat antibodies; mice anti-antibodies

Storage: room temperature (1 - 40°C). DO NOT FREEZE.
The cassette is sensitive to humidity and heat. Use the tests immediately after opening the package. DO NOT USE the test if the package is damaged.

- Buffer :
  One bottle contains:
  - Bovine albumine
  - Triton-X 100

  Storage: room temperature (1 - 40°C). DO NOT FREEZE.
- Note: All tests included in the kit should only be performed using the buffer included in the same kit.
- Explanatory brochure

3.2.2. Required supplementary material

- Non sterile gloves, disposable
- Marker
- Micropipette of 5-20 µl
- Pipet tips
- Timer
- « Bio-hazard » container

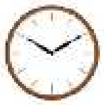
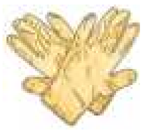
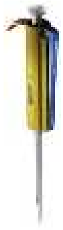
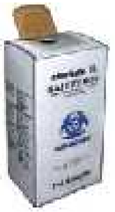
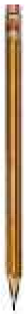

3.2.3. Sample to be examined

- Whole blood (collected with EDTA): 5 µl
- Storage: Execute the test immediately or store at 2-8°C up to maximum 3 days. For longer periods, freeze the sample at -20°C.

Note : Let the samples adjust to room temperature before performing the test.

3.3 Test execution

3.3.1 Internal quality control

A control line « C » is included in the system to validate the test. The result is invalid if the control line does not appear by the end of the test.

3.3.2 Internal quality control

1. Let the sample adjust to room temperature

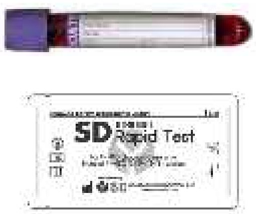

2.Check the expiry date on the package
(DO NOT USE after expiry date!)

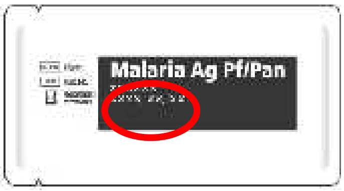

3. Put gloves

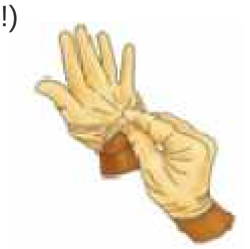

4. Open the package
a) Take the cassette

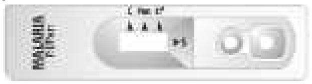

b) Check the colour of the silica gel

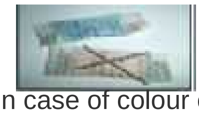

In case of colour change,
(blue turning to pink) use another test !

5. Write the date & patient number on the cassette

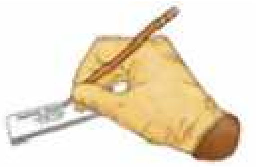

6. Aspirate 5µl of EDTA blood with a micropipette Use a new tip for each sample

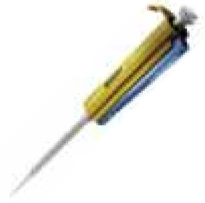

7. Add 5 µl of blood to the round well

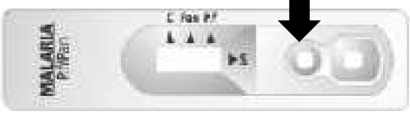

8. Add 4 drops of buffer to the squared well (hold the vial vertical)

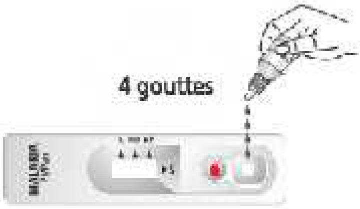

9. Start the timer. Read the result after 15min. If negative, repeat the reading at 30min (DO NOT read the result after 30min)

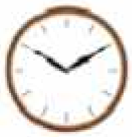

3.4 Interpretation of the test

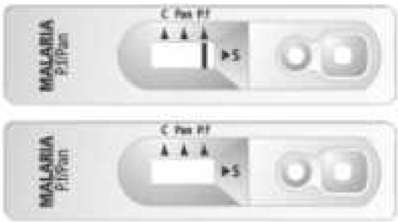

Control line « C » absent = invalid result. Repeat the test.

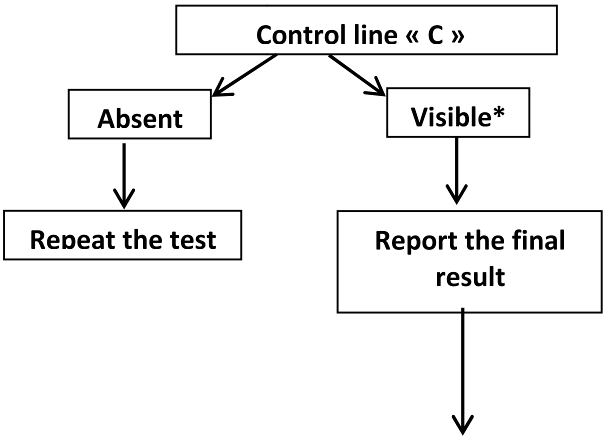

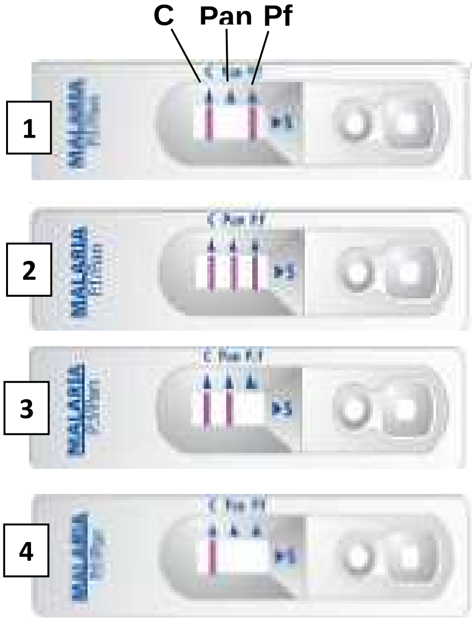

	Control line « C » visible	Pan line visible	Pf line visible	Final result to report
1	Yes	No	Yes	Infection *with P. falciparum*
2	Yes	Yes	Yes	*Infection with P. falciparum* infection; mixed infection possible (*P. falciparum* with *Plasmodium* non-*falciparum*)
3	Yes	Yes	No	Infection with *Plasmodium* non-*falciparum*
4	Yes	No	No	Negative

*** Irrespective of band intensity**

Intensity of the reaction

If the reaction is POSITIVE, use the following scaling system to grade the intensity of the test lines:

Faint	Test line visible, but very faint
Weak	Test line visible, but weaker than the control line
Medium	Test line intensity equals control line intensity
Strong	Test line more intense than control line

Do this for the two test lines (Pf and Pan).

3.5 Result recording in the CRF

- Record in the CRF if the test was done or not, and provide a reason if it was not done.
  Record if the control line is present (= valid result): “Yes” or “No”.
  If “No”, a repeat test was done.
- If the control line of the repeat test is not present, record the result as “Invalid”.
- Record the result of the test: “Negative”,” Positive” or “Invalid”.
- If the test is positive, record the intensity of the test lines (P.f and Pan): “Faint, weak, medium or strong”.

3.6 Waste management, cleaning

- Discard the used tests in a « bio-hazard » container.
- Discard the left-over of the buffer (after having used all the tests in the kit) in a « bio-hazard » container.

4. Records and archives

Appendixes & forms to be completed	
**Number**	**Title**
1	Laboratory CRF

5. References

- Instructions of manufacturer, version 05FK60-EN-14 2013.04

6. Document History

Revision	
SOP-WP2-LAB-35-V1.0-12Jul2012	Initial version
SOP-WP2-LAB-35-V1.1-03Dec2012	▪ Translation in English<br>▪ Addition of “Intensity of the reaction” and “3.5 Result recording in the CRF”
SOP-WP2-LAB-35-V2.1-11Apr2014	▪ Adaptation of time of reading

Name and function	Date	Signature
Auteur		
Barbara Barbé	11/04/2014	
Revised by		
Approved by		
Ninon Horié	11/04/2014	

SOP-WP2-LAB-17-V1-10Sep2013

	**SOP Title:** Performing the DAT
	**Study title:** Evaluation of Rapid Diagnostic Tests (RDT) in association with clinical and laboratory predictors for the diagnosis of Neglected Tropical Diseases (NTD) in patients presenting with persistent fever (≥1 week) in Cambodia, Nepal, Democratic Republic of the Congo and Sudan

1. Scope and application

The Direct Agglutination Test is used for the quantitative detection of antibodies against *Leishmania donovani* or *Leishmania infantum* in human serum or blood in patients with visceral leishmaniasis.DAT/VL antigen consists of a freeze-dried suspension of trypsin-treated, fixed and stained promastigotes of *L.donovani*. The test is performed inmicrotitre plates**.** This SOP describes how to perform the DAT which should be performed on all patients enrolled in the persistent fever syndrome of NIDIAG study in Nepal and Sudan.

2. Responsibilities

**Function**	**Activities**
Laboratory Technician 1	- Performance of the Direct Agglutination Test<br>- Interpretation of the DAT results (blinded to the results of the index tests rK39 and rK28)<br>- Reporting of the results in the laboratory register
Laboratory Technician 2	- Second reading and interpretation of the DAT
Laboratory Technician 3	- Final interpretation of the DAT in case of discrepancy between reader 1 and 2
Site investigator	- Copy the DAT results in the Fever CRF v2.0

3. Procedures

3.1 Specimen

- Do not freeze the DAT buffer and DAT diluents.
- Do not freeze the reconstituted antigen suspension.
- Take care to avoid any contamination between the test, pipettes and other materials.
- 2 MercaptoEthanol (2-ME) and sodium azide are toxic so handle cautiously.

3.2 Safety

- Handle all samples as potentially infectious.
- Practice safety precautions for handling and disposal of the infectious materials.

3.3 Storage

- Store the DAT-antigens at +2 to + 8°C.
- Store the DAT-buffer and DAT-diluents in the refrigerator at +2to + 8°C up to 6-12 months.
- Store the positive and negative controls at-20°C.
- Store 2 MercaptoEthanol at +2 to + 8°C.
- Keep the reagents away from direct sunlight and dust.

3.4 Materials and samples

3.4.1 Reagents

a) DAT-ANTIGEN (2.5 ml/vial)(ITM, Antwerp)

- Freeze dried suspension of purified, trypsin-treated, fixed and stained promastigotes of *L. Donovani* strain 1-S.
- Preservative: sodium azide (0.1 %)

b) DAT-BUFFER

- Phosphate Buffered Saline (PBS – pH 7.2) supplemented with protein.
- Used for reconstitution of DAT-antigen, positive and negative controls.
- Preservative: sodium azide (0.1%)

c) POSITIVE CONTROL

The positive control is prepared with VL-positive serum of DAT titre of 1:102 400.

d) NEGATIVE CONTROL

The negative control is prepared with VL-negative serum of DAT titre below 1: 200.

e) DAT-DILUENT

õ Phosphate Buffered Saline (PBS – pH 7.2) supplemented with protein and added 2-ME.
õ Used to prepare the sample dilutions.
õ Preservative: sodium azide (0.1%)

f) 2-MERCAPTO-ETHANOL (1 ml/vial)

õ Commercial 2-ME (Merck Ref.805740)

3.4.2 Additional material required

õ Tube
õ Timer
õ Latex examination gloves
õ Discarding jar
õ V-Shape microtitre plates + re-usable covers or plate sealers
õ Pipette (5.0 ml) for reconstituting the antigen
õ Automatic pipette + tips (range needed: 600 µl for reconstitution of positive and negative control sera and 240 µl for adding 2-ME to the DAT/VL diluent)
õ Automatic pipette 10-200 µl + tips
õ Multichannel pipette with 12 channels - pipetting volume needed 50 µl
õ Plastic stirring rods or equivalent
õ 2ml microcentrifuge tube with cap
õ Pre-printed reading sheets *(see annex)*

3.4.3 Samples

Serum (1 µl)

3.5 Procedure

3.5.1 Preparation prior to testing

Reconstitution of the DAT-ANTIGEN

- Add 2.5 ml of DAT-BUFFER to a vial of freeze dried DAT-antigen.
- Immediately gently shake the vial for a few seconds so as to obtain a homogeneous suspension.
- The antigen is ready for use.
  Note: Before each use, gently shake the vial for a few seconds.
- **Reconstitution of the controls**Using an automatic pipette, add 600 µl of DAT-BUFFER to a vial of the positive control and to a vial of the negative control.

Preparation of DAT –BUFFER *(refer to Annex 1)*

Preparation of DAT-DILUENT

õ Using an automatic pipette add 240µl of 2-Mercapto-Ethanol to the vial (30 ml) of DAT-DILUENT.

õ Use this solution to prepare the sample dilutions.

Preparation of V-shaped microtitre plate

Note that the first dilution starts at 1: 200 and the last at 1:12800

õ Prepare the pre-printed reading sheets.

õ Write an identification number on each microtitre plate.

õ With a marker draw a line onto the microtitre plate before the antigen control (= in between row 11 and row 12) in order to avoid titrating one step further.

õ On the pre-printed reading sheet *(see example in Annex 2)*:
- Mark date and batch numbers on top of the sheet
- Mark the ID number of the microplate
- Mark the sample's ID next to the dilution row on the sheet. Use the sample ID to identify which sample will go in which row.

3.5.2 Test procedure

1. Add 100 µl positive control in A1 and 100 µl of negative control in B1position of 96-well microtitre plate.
2. Add 100 µl of DAT diluent into the remaining wells of column 1 (C-H).
3. Using a multichannel pipette dispense 50µl of DAT diluent into the wells of column 2-8 (A-H).
4. Add 1 µl of patient's serum in column 1 (C-H). DO NOT ADD PATIENT SERUM IN A1 AND B1.
5. Mix the serum in column 1 (C-H) thoroughly with the multichannel pipette up and down.
6. Transfer 50 µl from column 1 (A-H) to column 2. Avoid touching the tips into the wells.
7. Mix thoroughly with multichannel pipette up and down and transfer from column 2 to column 3.
8. Repeat the steps 5 and 6 until column7 and discard the last 50 µl into the discarding jar.
9. Add 50 µl of antigen solution into each well [row 1- 8, column A-H] of the microtitre plate.

10. Seal the plate with an adhesive plate sealer.
11. Gently shake the plate by quickly "rubbing" it back and forth over the table top or shake in plate shaker for 1 minute. Avoid moistening the underside of the plate sealer.
12. Incubate overnight at ambient temperature in a horizontal position and do not touch the plate.
13. Read the results next day.

3.5.3 Reading, interpretation and recording

3.5.3.1 Reading of DAT

- Read the test by placing the plate onto a white background.
- Read the antigen wells (column 8) first.
- Then read row A (positive control) and row B(negative control).
- Take the reading of subsequent rows.

3.5.3.2 Interpretation of results

- Negative result

Dark blue dot of the size identical to the size of the antigen control dot is considered negative *(See Figure 1)*.

- Positive result

Film formation in the microwell and/or the appearance of a dot larger than the antigen dot is considered as agglutination and thus indicates a positive result.

3.5.3.3 Recording of the result

- Take the last well in which the agglutination is seen as end point titre and note down the corresponding dilution in the lab register / reading paper.
- Two independent readers should read the test, each one filling in a reading paper independently.
- Compare the two reading papers, and in case of discrepancy, both readers should return to the microplate and come to a common final result.
- If no consensus can be reached call a third reader (i.eTechnician 3) as a referee.
- Write the comments concerning difficulties on fixing a titre on the back of the reading sheets/ lab register.

Figure 1 Interpretation and reading of DAT microtitre plates.

Positive: Titre of ≥ 3200 is considered positive for visceral leishmaniasis.

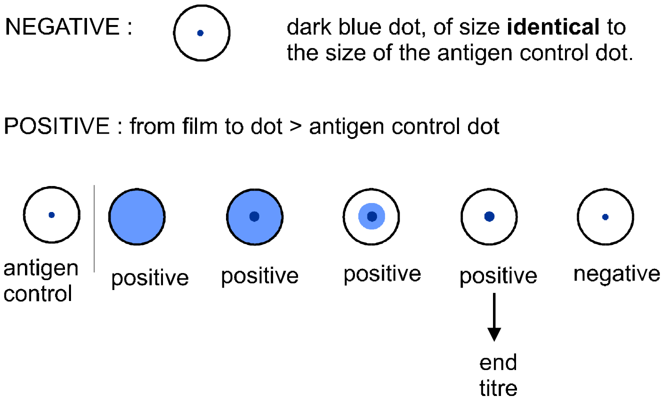

3.6 Waste management

õ Discard the used test and the plastic pipette in a biohazard waste container.

3.7 Documentation of result

õ Record the result in the laboratory register.
õ The site investigator will copy the lab results in the CRF.

4. Records and archives

Appendices and forms to complete	
**Number**	**Title**
SOP-WP2-LAB17-V01-10Sep2013-Annex 1	Preparation of DAT Buffer
SOP-WP2-LAB17-V01-10Sep2013-Annex 2	DAT reading sheet

5. References

1. JacquetD,BoelaertM,SeamanJ,RijalS,SunderS,MentenJ,MagnusE.Comparative evaluation of freeze dried and liquid antigens in the direct agglutination test for serodiagnosis of visceral leishmaniasis (ITMA-DAT/VL)*Trop Med Int Health,*2006;11(12):1777-84.
2. El HarithA, KolkAHJ, Kager PA et al.A simple and economical direct agglutination test for serodiagnosis and seroepidemiological studies of visceral leishmaniasis.*TransRSocTropMed Hyg*,1986;80:583-586
3. El HarithA, KolkAH, Leewenberg J et al .Improvement of a direct agglutination test for field studies of visceral leishmaniasis.*JClinMicrobial,1988;*26:1321-1325.
4. DAT/VL Direct agglutination test for visceral leishmaniasis, product leaflet, with freeze dried antigen on blood impregnated filter papers. Applied technology and production unit, Institute of Tropical Medicine, Antwerp, Belgium

6. Document History

Revision	
SOP-WP2-LAB17-v01-10SEP2013	Initial version

Name and function	Date	Signature
Author		
Basudha Khanal	26/10/2012	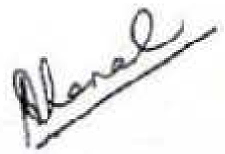
Reviewed by		
Sayda El Safi	28/10/2013	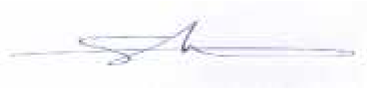
Barbara Barbé	28/10/2013	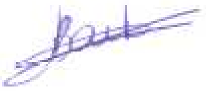
Approved by		
Ninon Horié	28/10/2013	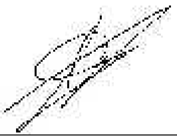

SOP-WP2-LAB-17-V1-10Sep2013-Annex1

Preparation of DAT-Buffer

Use

- DAT- buffer is used in the DAT-test for the reconstitution of the freeze dried reagents and for the elution of filter papers.

Safety

- Sodium azide is toxic, so handle it cautiously.

Reagents

- Distilled water (1020 ml)
- Ingredients for Phosphate Buffer Saline (PBS) (see below)
- Sodium azide (NaN_3) (1.00g)
- UltroSer G (US-G)

Materials

- Balance (0.01g accuracy)
- Magnetic stirring device
- pH meter

Methods

1. Preparation of PBS + Sodiumazide

- Wear gloves
- Weigh the following ingredients:

Ingredients	Weight
NaCl	7.20g
$Na_2HPO_4.2H_2O$	1.86g
KH_2PO_4	0.43g
NaN_3	1.00g

- Add all the reagents to 1000 ml of distilled water.
- Stir on a magnetic stirring device until the ingredients are completely dissolved.
- Measure the pH at room temperature. Adjust to pH 7.2.
- The final pH should be between 7.1 and 7.3.

2. Reconstitution of US-G

- The lyophilised powder is solubilised in 20 ml of distilled water.
- Open the flask by removing the seal and the rubber stopper and introduce 20 ml distilled water by means of a pipette.
- Allow the lyophilisate to swell for 10-15 minutes. Shake the flask slightly or draw up and down a pipette to obtain the quick solubilisation.
- Wait untill the reconstituted solution is clear and pink-colored.
- Aliquot the solution by the volume that will be used in one single time for the preparation of the DAT/VL buffer and then freeze immediately at -20^0C. It cannot be refrozen.

3. Preparation of DAT- Buffer

- Add US-G to the PBS-Azide in the proportion of 1 ml US-G to 500 ml PBS-Azide.

4. Production sheet for DAT-buffer

- Fill out the production sheet (see below) for each batch of DAT-buffer prepared.
- Store the production sheet in the lab file.

5. Storage

- Store all solutions between 2-8^{0}C
- Store the DAT-buffer at 2-8^{0}C for 6-12 months.

6. Quality control

1. Internal quality control

Test all DAT reagents by performing DAT on a well-known positive and negative sample, either serum or filter paper.

2. External quality control

Of each batch prepared, send 50 ml should to Antwerp for the external quality control.

Production sheet: DAT–Buffer

DAT- Buffer: Batch.................

Date..........

	Batch	volume
Distilled water		1000ml
NaCl		7.20 g
$Na_2HPO_4.2H_2O$		1.86 g
KH_2PO_4		0.43 g
Na-azide		1.00 g
pH		
Ultroser G		2 ml
Quality control	Date	☐ OK
		☐ Not OK

DAT- Buffer: Batch.................
Date..........

	Batch	volume
Distilled water		1000ml
NaCl		7.20 g
$Na_2HPO_4.2H_2O$		1.86 g
KH_2PO_4		0.43 g
Na-azide		1.00 g
pH		
Ultroser G		2 ml
Quality control	Date	☐ OK
		☐ Not OK

Design of microtitre plate and NIDIAG LAB DAT reading sheet

NIDIAG Lab DAT reading sheet

Date: ……………………………… Name of the performer: …………………………………… Name of the reader: ……………………………………

Date: DAT/VL Antigen batch: ……………………………… Positive control: …………………………… Negative control: ……………………………

DAT/VL Buffer batch: …………………………………… DAT/VL Diluent batch: ………………

Column	Sample code	Positive titre
12		
11		
10		
9		
8		
7		
6		
5		
4		
3		
2		
1		

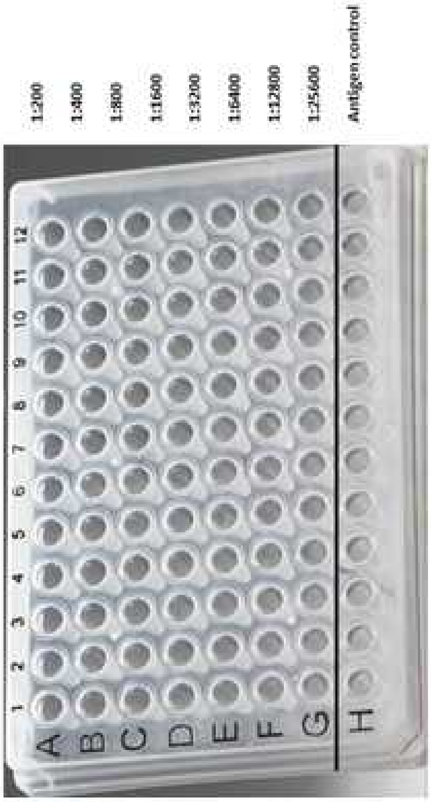

	**SOP Title:** Inoculating and growing cultures of *L. donovani* from body fluids
	**Project/study**: Evaluation of Rapid Diagnostic Tests (RDT) in association with clinical and laboratory predictors for the diagnosis of Neglected Tropical Diseases (NTD) in patients presenting with persistent fever (≥1 week) in **Nepal**

1. Scope and application

This SOP describes the method for *in vitro* cultivation of *Leishmania donovani* from body fluids and aims at all medical and laboratory workers who will be involved in *in vitro* cultivation of *L. donovani* throughout NIDIAG fever study (WP2).

In vitro cultivation is the process whereby leishmanial organisms are removed from their host and transferred into culture media so that multiplication can occur. *In vitro* cultivation of *L. donovani* improves sensitivity of VL diagnosis. Specimens for culturing the organism within NIDIAG include bone marrow (BM) or lymph node (LN) aspirates.

This SOP is applicable in the Nidiag fever site in **Nepal** only.

2. Responsibilities

Function	Activities
Study investigator	Bone marrow aspiration.
Laboratory Technician	Bone marrow collection, inoculation in the culture medium, subcultures, examination of culture and reporting of results.
Central laboratory assistant	Preparation of culture media.

3. Procedures

3.1. Specimen

- Collect BM or LN aspirates according to SOP-WP2-CLIN-09 and SOP-WP2-LAB-48, respectively.

3.2. Precautions

- Use strict aseptic precautions to inoculate the specimen into the tubes of medium.
- Do not inoculate material into large volumes of culture medium.

3.3. Materials

- Equipment:

  Binocular light microscope

  Water bath at 56°C

  Incubator at 26°C

  Refrigerator at 2-8°C

  Autoclave

Safety cabinet

pH meter

Gas or spirit burner

- Solutions *(see annexes 1-4)*

3.4. Procedure

3.4.1 Inoculation of culture medium and incubation

1) Label the culture tubes with the patient's study number.
2) Restrict the volume of the liquid phase to 0.5 ml or less.
3) Introduce the inoculum into the liquid portion of the biphasic Tobies medium (modified NNN), NOT into the blood agar.
4) Inoculate at least two tubes if sufficient material is available, as it increases the chances of obtaining growth.
5) Incubate the culture tubes as soon as possible (within 15 minutes after aspiration) in cooled incubator between 22°C - 25°C (remember that more *Leishmania* cultures are killed by heat than by cold).
6) Maintain cultures and transport them from the field to the central laboratory between 22°C- 25°C.

3.4.2 Routine examination and reporting of results

1) Examine the cultures regularly, using aseptic precautions every 3 days.

2) Remove a small drop of medium with a sterile Pasteur pipette or similar and place on a microscope slide under a coverslip.

3) Examine under low power objectives (x10 or x20). Transfer 2 or 3 drops into fresh medium as soon as motile promastigotes are seen.

4) Add small volumes (approx. 0.5 ml) of fresh medium to the culture tubes when the organisms increase in number.

5) Sub-inoculate immediately into fresh culture medium containing higher concentrations of different antibacterial or antifungal agents if contamination is observed.

6) Keep the negative tubes up to 8 weeks; discard them if still negative after this time.

7) Report as positive for *Leishmania* spp. if organisms are found prior to or at the end of 8 weeks.

8) Discard the tubes and report as negative for *Leishmania spp.* if no organisms are seen after 8 weeks of incubation.

3.4.3 Quality control

- Check all reagents and media at least once a week. The media should be free of any signs of precipitation and bacterial and/or fungal infections.
- Each medium is identified with a batch number and the date it was made. The medium should be used within 2-3 weeks.
- For each batch, a sterility check is performed by incubating at 37°C for 48 hours.
- The performance of each batch is verified with known standard Leishmania culture strain at 26°C for four days.
- Check and record refrigerator temperature in QC record daily (see SOP-WP6-QUAL06).
- Check and record incubator temperature in QC record daily (see SOP-WP6-QUAL06).

4. Definitions

NA

5. Records and archives

Number	Title
WP2-LAB-16-Annex 1	Proline Balanced Salts Solution (PBSS)
WP2-LAB-16-Annex 2	Evans Modified Tobie's Medium
WP2-LAB-16-Annex 3	Tobies Blood Agar
WP2-LAB-16-Annex 4	Locke Solution

6. Document History

**Revision**	
SOP-WP2-LAB-16-V01-10Feb2013	Initial version
SOP-WP2-LAB-16-V2.0-09Oct2013	Site specific version of Nepal
SOP-WP2-LAB-16-V2.1-10Sep2013	Site specific version of Sudan

**Name and function**	**Date**	**Signature**
Author		
Barbara Barbé	09/10/2013	
Sayda El Safi	28/10/2013	
Reviewed by		
Basudha Khanal	26/10/2013	
Approved by		
Ninon Horié	28/10/2013	

WP2-LAB-16-Annex 1

Proline balanced salts solution (PBSS)

(A useful general-purpose salts solution and liquid phase for biphasic culture media for *Leishmania*).

KCL	0.4 g
Na_2HPO_4 $2H_20$	0.06 g
KH_2PO_4	0.06 g
$CaCl_2.2H_2O$	0.185 g
$MgSO_4.7H_2O$	0.1 g
$MgCl_2.6H_2O$	0.1 g
NaCl	8.0 g
L-Proline	1.0 g
*Phenol red	0.001 g
Distilled Water 1000 ml	

Dissolve the ingredients, one at a time, in approx. 750 ml of distilled water. Adjust the pH to 7.2 with solid Tris (Tris(hydroxymethyl)aminomethane), make up the volume to 1000 ml, dispense into convenient screw-cap containers and autoclave at 121°C for 15 min. Store preferably at 4°C, but will withstand several months at room temperature.

Evans Modified Tobie's Medium

(A rich biphasic medium which has been used successfully for the isolation of a great variety of leishmaniasis from both Old and New World sources)

<u>Solid phase :</u>

Beef extract (Oxoid Lab-Lemco L29)	0.3 g
Bacteriological peptone (Oxoid L37)	0.5 g
NaCl	0.8 g
Agar (Oxoid purified)	0.2 g
Distilled water	100 ml

<u>Method :</u>

Mix and heat the ingredients in a flask as for 3N medium. Transfer the molten agar into culture tubes and autoclave at 121°C for 15 min. Cool the sterilized agar to about 55°C, then add defibrinated rabbit blood (inactivated by heating at 56°C for 30 min) to give a final concentration of approximately 15%. Mix and slope as for 3N medium.

<u>Liquid phase</u> :

A liquid phase other than the simple water of condensation is used, and this is proline-containing balanced salts solution (PBSS) [see SOP-WP2-LAB16 Annex1].

Add 0.2 - 0.3 ml of the liquid phase to the agar slope immediately before inoculation.

Note on the use of blood other than rabbit blood in biphasic medium: Quite often rabbit blood is not easily available for inclusion in biphasic media such as 3N or USAMRU. In such cases mammalian blood other than rabbit may be used. Sheep, horse, and human blood have all been used, but it is worth experimenting with whatever bloods are easily available. With bloods other than rabbit use them either defibrinated or with an anticoagulant, but always heat- inactivate them (56°C: 30 min) and increase the concentration of agar-agar in the medium to 2%.

<u>Sterility checking of blood agar :</u>

Incubate freshly made blood agar medium at 37°C for 24 hours and examine the surface of the blood agar for signs of bacterial growth. Discard immediately any medium showing signs of bacteria.

<u>Storage :</u>

Store at 4°C, and if a separate liquid phase is to be added, do not add this until the medium is to be used. These media are best used within one week of making up. Discard after 3 weeks at 4°C.

Tobies Blood Agar (medium used in Nepal)

Tobie EJ, 1949

Ingredients

Bidistilled water	1000 ml
Bacto-tryptose (Difco)	15.0 g
NaCl	4.0 g
Na2PO4.12H2O	5.0 g
KCl	0.4 g
Bacto-Agar (Difco)	15.0 g

pH 7.6 adjusted with HCl or NaOH1N

Method of preparation

- Mix the ingredients in a flask and dissolve by boiling onto heating plate with magnetic stirring.
- Dispense per 80ml in 250 ml screw –capped bottles
- Autoclave at 121°C for 20 min
- Store the media at 4°C
- Before use, take out the media from refrigerator and melt in boiling water bath, then cool down at 56°C for 30min in a water bath.
- Add 20 ml rabbit blood obtained by aseptic heart puncture using 5% polyanethol sulphonic acid (PAS)
- Mix gel and blood, dispense in tubes (about 1ml in 10x100mm test tubes), slant
- Incubate at 37°C for 24 hours for sterility control
- Add between 0.5 ml Locke or Locke –Krebs per tube just before use

WP2-LAB-16-Annex 4

Preparation of Locke solution (for overlaying Tobie's medium)

Ingredients

Bidistilled water	1000 ml
NaCl	8.00 g
KCl	0.20 g
KH_2PO_4	0.30 g
$MgSO_4.7H_2O$	0.1 g
$NaHCO_3$	1.00 g
Glucose	2.50 g
Penicillin	200.000 IU
Streptomycin	200.000 µg

pH adjusted to 7.4 with HCl or NaOH 1N

	**SOP Title:** Inoculating and growing cultures of *Leishmania donovani* from tissue aspirates
	**Project/study**: Evaluation of Rapid Diagnostic Tests (RDT) in association with clinical and laboratory predictors for the diagnosis of Neglected Tropical Diseases (NTD) in patients presenting with persistent fever (≥1 week) in **Sudan**

1. Scope and application

This SOP describes the method for *in vitro* cultivation of *Leishmania donovani* from body fluids and aims at all medical and laboratory workers who will be involved in *in vitro* cultivation of *L. donovani* throughout NIDIAG fever study (WP2).

In vitro cultivation is the process whereby leishmanial organisms are removed from their host and transferred into culture media so that multiplication can occur. *In vitro* cultivation of *L. donovani* improves sensitivity of VL diagnosis. Specimens for culturing the organism within NIDIAG include bone marrow (BM) or lymph node (LN) aspirates.

This SOP is applicable in the Nidiag fever site in **Sudan** only.

2. Responsibilities

Function	Activities
Study investigator	Bone marrow aspiration
Field technician	Lymph node aspiration, specimen (LN and BM) collection, handling, labeling at study site, inoculation of culture medium and incubation until transportation to the central laboratory (Soba University Hospital)
Central laboratory technician	Laboratory manipulations and labeling in central (reference) laboratory<br>Quality Control
Central laboratory chief technician	Backup for central lab technician when he is not available
Central laboratory assistant	Preparation of culture media

3. Procedures

3.1. Specimen

- BM or LN aspirates: 1-2 drop(s)

  Refer to **SOP-WP2-CLIN-09** and **SOP-WP2-LAB-48**, respectively.
- If the LN sample is not sufficient, repeat aspiration from a LN on the other side of the body.

3.2. Precautions

- Use strict aseptic precautions to inoculate the specimen into the tubes of medium.
- Do not inoculate material into larger volumes of culture medium than the ones specified in this SOP (see Annex 1).
- Work under a safety cabinet when doing routine examinations at the central laboratory.

3.3. Materials

- Equipment:
  - Binocular light microscope
  - Microscopy slides
  - Covers slips
  - Pasteur pipettes (sterile)
  - Incubators at 25°C and at 37°C (at central lab)
  - Refrigerator at 2-8°C
  - Autoclave
  - Safety cabinet
  - pH meter
  - Gas or spirit burner

- Solutions and media *(for preparation see annexe 1)*:
  - Proline Balanced Salts Solution (PBSS)
  - NNN medium
  - Sloppy Evan's Medium

3.4. Procedure

3.4.1 Inoculation of culture medium and incubation – at field site

1) Label the culture tubes with the patient's study number.
2) Restrict the volume of the liquid phase of the NNN medium to 0.5 ml or less.
3) Introduce the inoculum (1-2 drops) aseptically into the liquid portion of the biphasic NNN medium (NOT into the blood agar), and in the semi-solid Sloppy Evan's medium, with the same needle used for aspiration.
   Note: Inoculate at least two tubes of both NNN and Sloppy Evan's medium, if sufficient material is available, as it increases the chances of obtaining growth
4) Incubate the culture tubes as soon as possible (within 15 minutes after aspiration) between 22°C - 25°C (remember that more *Leishmania* cultures are killed by heat than by cold).
5) Maintain cultures at 22-25°C and transport them once a week from the field to the central laboratory.
   Note: At the field site, the cultures are inoculated in the pharmacy, where temperature is constant and is kept below 25°C.

3.4.2 Routine examination and reporting of results – at central laboratory

6) Incubate the cultures at 22-25°C in an incubator.
7) Examine the cultures regularly, using aseptic precautions:
   - Perform the first check within 4 to 9 days after inoculation (depending on the date of inoculation and the transport from the field (every Saturday)).
   - Perform the subsequent checks every 3 days (up to 8 weeks after inoculation).
8) Remove a small drop of medium with a sterile Pasteur pipette and place on a microscope slide under a coverslip.
9) Examine under low power objectives (x10 or x20).
10) Transfer 2 or 3 drops into fresh medium as soon as motile promastigotes are seen.
11) Add small volumes (approx. 0.5 ml) of fresh medium to the culture tubes when the organisms increase in number.
12) Sub-inoculate immediately into fresh culture medium containing higher concentrations of the antibacterial or antifungal agents if contamination is observed.
13) Keep the negative tubes up to 8 weeks. Report as negative for *Leishmania spp*. if no organisms are seen after 8 weeks of incubation.
14) Report as positive for *Leishmania spp.* if organisms are found prior to or at the end of 8

weeks.

15) Discard the tubes.

3.4.3 Quality control

- Check all reagents and media at least once a week. The media should be free of any signs of precipitation and bacterial and/or fungal infections.
- Each medium is identified with a batch number and the date it was made.
- For each batch of medium, a sterility check is performed by incubating at 37°C for 48 hours, and checking for signs of bacterial growth.
- The performance of each batch of medium is verified by inoculating with a standard *Leishmania* culture strain and incubation at 25°C for four days.
- Check and record refrigerator temperature in QC record daily (see SOP-WP6-QUAL06). Check and record incubator temperature in QC record daily (see SOP-WP6-QUAL06).

4. Definitions and abbreviations

BM: Bone marrow

LN: Lymph node

NNN medium: Novy-MacNeal-Nicolle medium

PBSS: Proline Balanced Salts Solution

QC: Quality control

5. Records and archives

Number	Title
WP2-LAB-16-V02-10Sep2013-Annex1	Proline Balanced Salts Solution (PBSS), NNN and Sloppy Evan's media

6. Document History

**Revision**	
SOP-WP2-LAB-16-V01-10Feb2013	Initial version
SOP-WP2-LAB-16-V2.0-09Oct2013	Site specific version of Nepal
SOP-WP2-LAB-16-V2.1-10Sep2013	Site specific version of Sudan

**Name and function**	**Date**	**Signature**
Author		
Sayda El Safi	04/09/2013	
Reviewed by		
Barbara Barbé	17/10/2013	
Approved by		
Ninon Horié	21/10/2013	

Annex 1: Preparation of solutions and media required for *Leishmania* culture

A. Proline Balanced Salts Solution (PBSS)

(A useful general-purpose salts solution and liquid phase for biphasic culture media for *Leishmania*).

KCL	0.4 g
Na_2HPO_4 $2H_20$	0.06 g
KH_2PO_4	0.06 g
$CaCl_2.2H_2O$	0.185 g
$MgSO_4.7H_2O$	0.1 g
$MgCl_2.6H_2O$	0.1 g
NaCl	8.0 g
L-Proline	1.0 g
Phenol red	0.001 g
Distilled Water	1000 ml

1. Dissolve the ingredients, one at a time, in approximately 750 ml of distilled water.
2. Adjust the pH to 7.2 with solid Tris (Tris(hydroxymethyl)aminomethane)
3. Make up the total volume to 1000 ml by adding distilled water
4. Dispense the solution into convenient screw-cap containers and autoclave at 121°C for 15 min.
5. Label the containers as PBSS with the preparation date and the expiration date (1 month from the date of preparation).
6. Store at 4°C.

B. NNN medium

1. Preparation

a. Solid phase

Bacto Agar	1.4 g
Sodium chloride (NaCl)	0.6 g
Double-distilled water<br>Gentamycin (200µg/ml)<br>Defibrinated rabbit heart blood	90 ml<br>4 ml<br>10 ml

b. Liquid phase

Sterile normal saline 0.5 ml per tube

2. Method

1. Mix the NaCl and Bacto agar with the double-distilled water in a 500 ml flask.
2. Heat the mixture until the agar melts.
3. Autoclave at 121°C for 15 minutes.

4. Cool to about 50°C.
5. Add in a sterile way 10 ml of aseptically collected defibrinated rabbit heart blood and mix well
6. Add 2 ml of 200 µg/ml Gentamycin.
7. Dispense 4 ml into sterile screw-caped culture tubes.
8. Place the tubes at 10° angle until the agar sets.
9. Label the tubes as NNN medium with the preparation date and an expiration date of 3 weeks from the date of preparation.
10. Store at 4°C.
11. Add 0.5 ml of sterile normal solution as an overlay prior to inoculation in each tube

C. Sloppy Evan's Medium

1. Preparation

Proline Balanced Salts Solution (PBSS)	80 ml
Bacteriological peptone (Oxoid L37)	0.1 g
Beef extract (Oxoid Lab-Lemco L29)	0.03 g
Agar (Oxoid purified)<br>Gentamycin 200µg/ml<br>Defibrinated rabbit heart blood	0.3 g<br>4 ml<br>20 ml

2. Method

1. Mix all the ingredients in 80 ml PBSS in a flask using a magnetic stirrer.
2. Heat the mixture until the agar melts.
3. Autoclave at 121°C for 15 minutes.
4. Cool to about 50°C.
5. Add in a sterile way 20 ml of aseptically collected defibrinated rabbit heart blood and mix well
6. Add 2 ml of 200 µg/ml Gentamycin.
7. Dispense 4 ml into sterile screw-caped culture tubes.
8. Place the tubes at 10° angle until the agar semi sets.
9. Label the tubes as Sloppy Evans medium with the preparation date and an expiration date of 3 weeks from the date of preparation.
10. Store at 4°C.

D. Sterility check of media

Incubate freshly made NNN and Sloppy Evan's medium at 37°C for 48 hours and examine the surface of the agars for signs of bacterial growth. Discard the batch immediately any medium showing signs of bacteria.

E. Quality control

Verify the performance of each batch of medium by inoculating with standard *Leishmania* culture strain and incubating the media at 26°C for four days.

	**SOP Title:** Detecting *L. donovani* in body fluids and tissue aspirates
	**Study title:** Persistent fever syndrome of the NIDIAG study

1. Scope and application

This procedure is used for the detection of *Leishmania donovani* amastigotes in stained smears of aspirates obtained from tissues such as bone marrow, spleen and lymph node. In a microscopic examination of Giemsa stained smear, amastigote forms of *L.donovani* appear as round or oval structures inside or outside phagocytic cells (macrophages). Those structures are called Leishman Donovan (LD) bodies. This SOP is applicable to the parasitological diagnosis of visceral leishmaniasis in the patients enrolled in the persistent fever syndrome of NIDIAG study.

2. Responsibilities

Function	Activities
Laboratory Technician	Preparation, staining and microscopic examination of smears of tissue aspirates and reporting the results in the laboratory register and in the Lab Report Form of the CRF

3. Procedures

3.1 Materials and samples

3.1.1 Materials & equipments

- Glass slide: 7.5cm X 2.5cm
- Giemsa powder
- Methanol
- Glycerol
- Dropper
- Staining rack
- Timer
- Slide stand/tray
- Microscope
- Immersion oil
- Tray
- Diamond pencil
- Distilled water

3.1.2 Preparation of Giemsa stain

Stock solution

Giemsa powder: 0.75g

Methanol (CH_3OH): 65ml

Glycerol ($C_3H_8O_3$) : 35ml

Put the ingredients in a bottle containing glass beads and shake.Shake the bottle three times a day for 4 consecutive days .Filter into a staining bottle.Label the bottle ‘Giemsa Stain’and write date.

3.1.3 Samples

- Bone marrow aspirate
- Lymph node aspirate

3.2 Procedure

3.2.1 Precautions

- Handle the samples as potentially infectious.
- Practice safety precautions for handling and disposal of the infectious materials.

3.2.2 Preparation of smears

1. Prepare thin smears of the aspirate on at least two glass microscopic slides immediately after sample collection.
2. Deliver a drop of sample on to slide about 1 cm from one end.
3. In a bone marrow smear, suck off most of the blood with a fine Pasteur pipette applied to the edge of drop.
4. Take a smooth edged glass spreader to spread the drop along the slide to make a thin film of 3-5cm length. Ensure that the film has a good ‘tail’ and does not reach the edges of the slide laterally.
5. Allow the smears to air dry.
6. Fix with 100% methanol for one minute.
7. Dry and label the slide on one edge with the patient’s ID with the help of a diamond pencil.

3.2.3 Giemsa staining and microscopic examination

1. Dilute the stock Giemsa (1 volume of stain to 19 volumes of phosphate buffered water pH 6.8) and mix well
2. Overlay Giemsa stain (working solution) and leave for 25-30 minutes.

3. Wash the slides thoroughly under slow running tap water.
4. Wipe off the excess stain from under surface of the slide and air dry in a vertical position.
5. Examine the stained smears using a 100X oil immersion objective.

3.2.4 Interpretation of the results

- *Leishmania* amastigotes (LD bodies) are very small round or oval organisms about 3µm X 5µm found inside or outside the macrophages. Each LD body contains a red nucleus, smaller deep red kinetoplast and pale blue cytoplasm.
- Examine at least 1000 fields at 100X objective.
- If LD bodies are not seen, report the smear as LD bodies negative.
- If LD bodies are seen, report as LD bodies positive and grade as follows,

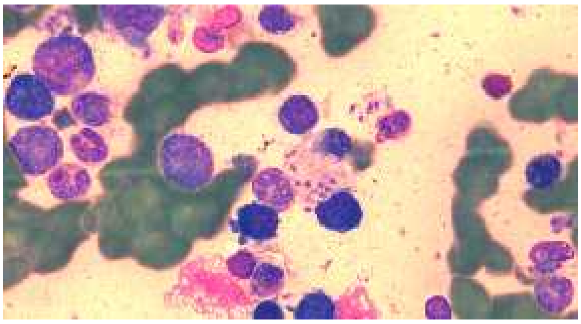

3.2.5 Grading scale

Average Amastigote density	Grade
>100 /field	6+
10-100/field	5+
1-10/field	4+
1-10/10 field	3+
1-10/100 field	2+
1-10/1000 field	1+

3.3 Documentation of results

Record the results in the laboratory register and the case-report form.

4. Records and archives

Appendices and forms to complete	
**Number**	**Title**
NA	NA

5. References

1. Manual on visceral leishmaniasis and control. WHO/LEISH/96.40

2. WHO manual of basic techniques for a health laboratory.2nd edition 2003

6. Document History

**Revision**	
SOP-WP2-LAB-15-V01-18Oct2012	Initial version

**Name and function**	**Date**	**Signature**
Author		
Basudha Khanal	26/05/2012	
Review by		
Sayda El Safi	01/09/2012	
Approved by		
Veerle Lejon	18/10/2012	

SOP-WP2-LAB-06-V4-20Aug2013

	**SOP Title:** Performing the rK28 from EASE-Medtrend
	**Study title:** Evaluation of Rapid Diagnostic Tests (RDT) in association with clinical and laboratory predictors for the diagnosis of Neglected Tropical Diseases (NTD) in patients presenting with persistent fever (≥1 week) in Cambodia, Nepal, Democratic Republic of the Congo and Sudan

1. Scope and application

rK28 Dynamic Flow (EASE-Medtrend) is a rapid test used for the detection of antibodies against *Leishmania*. It is an immunoblot assay in which the recombinant *Leishmania* antigens are coated in the test line of the device. Liquid conjugate applied to the reagent port (R) facilitates the migration of sample applied through the sample port (S). In case of the presence of *Leishmania* antibodies in the sample, antibodies captured by the conjugate react with specific coated *Leishmania* antigens. The reactions are demonstrated by the appearance of magenta bands on the test line and control line.

This SOP is applicable for diagnosis of visceral leishmaniasis in patients enrolled in the persistent fever syndrome of NIDIAG study in Sudan and Nepal.

2. Responsibilities

Function	Activities
Laboratory technician	Performing the rK28 (EASE Medtrend) blinded to the results of reference tests<br>Reporting the results in the laboratory register and in the Lab Report Form of the CRF

3. Procedures

3.1 Precautions

- Do not use the kit beyond expiry date.
- If the package is damaged, use another test device.
- Use the test device immediately after it is opened.
- Do not reuse the test device.

3.2 Safety

- Handle all samples as potentially infectious. Wear gloves during the procedure.
- Practice safety precautions for handling and disposal of the infectious materials.

3.3 Storage

- Store the test cards at 2 - 30°C.
- Store the whole blood treatment solution at 2 - 30°C.
- Store the <u>unopened</u> conjugate reagent at 2 - 30°C. Keep the <u>opened</u> conjugate reagent bottle at 2 - 8°C.
- DO NOT freeze.
- Keep the device sealed until used.
- Keep away from direct sunlight, moisture and heat.
- Refer to **SOP-WP6 -QUAL-05** for handling and storage of RDTs.

3.4 Materials and samples

3.4.1 Materials provided with the kit

- rK28(EASE-Medtrend) test package which contains:
    - Test device
    - Conjugate reagent
    - Whole blood treatment solution
    - Whole blood treatment vial

3.4.2 Additional materials required

- Micropipette (5-20µl)
- Timer
- Latex examination gloves
- Discarding jar (biohazard waste container)

3.4.3 Sample

- Whole blood, EDTA: 5 µl

  (Refer to the SOP-WP2-LAB-44 for the collection of blood)

  Comment: Use sample immediately.

3.5 Procedure

- Check the expiry date of the test. If expired, use a new lot which has not yet expired.
- Tear open the aluminium package and take out all the material.
- Take the test device and place it horizontally on a flat surface
- Write the patient's ID and the date on the label part of the test device
- In a blood treatment vial mix 5µl of whole blood solution and 5µl of whole blood. Wait for 1 minute.
- Hold the conjugate bottle vertically and transfer 2 drops (100µl) of conjugate into reagent port (R) (see figure 1)
- Wait for the conjugate to pass the sample port (S)
- Transfer 5µl of sample onto the sample port (S) (see figure 1)
- Read the results 5 minutes, 10 minutes and 15 minutes after the sample application. DO NOT interpret the result after 15 minutes.

Figure 1:

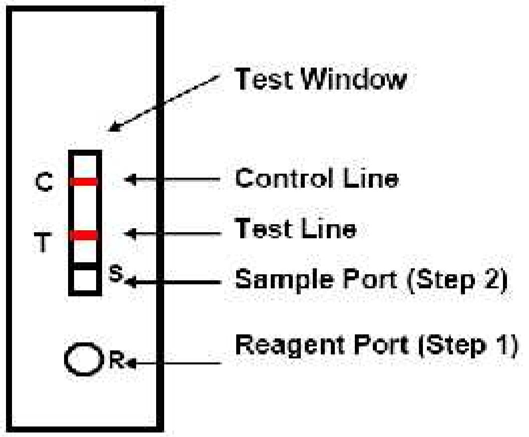

3.6 Interpretation of results

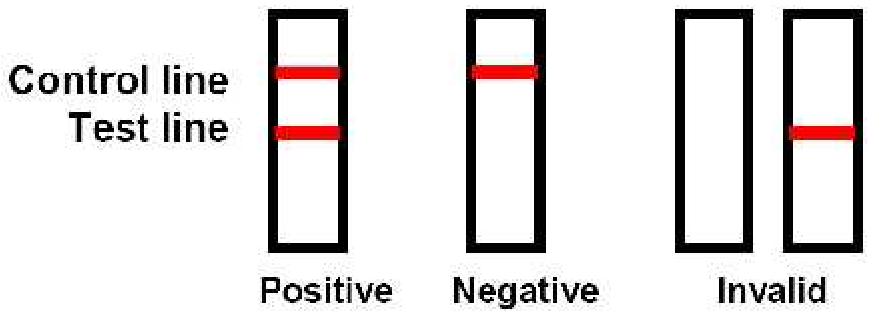

Positive reaction:

- Magenta coloured band appears both at the test and control lines.
- Indicates the presence of antibodies to *Leishmania spp.*
- A faint line in the test region is also considered as a positive reaction.

Negative reaction:

- Magenta coloured band appears only at the control line.
- Indicates the absence of detectable antibodies to *Leishmania spp.*

Invalid:

- Control line fails to appear.
- Repeat the test once using a new device.
- If the test result is still invalid, report as "invalid" in the CRF.

Background clearance

Record in the CRF if there is background visible after completion of the procedure:

Yes	Background visible
No	No background visible

If there is background visible, repeat the test once with a new test device.
If the second test result still has background, report "background present" in the CRF, but interpret the test as "Negative" or "Positive".

Intensity of the reaction

If the reaction is POSITIVE, use the following scaling system to grade the intensity of the test line:

Faint	Test line visible, but very faint
Weak	Test line visible, but weaker than the control line
Medium	Test line intensity equals control line intensity
Strong	Test line more intense than control line

3.7 Documentation of the results

- Nepal and Sudan use a lab register to record the results at 5, 10 and 15 minutes. Only the final result at 15 minutes is then transcribed at a later stage in the CRF.
- Record in the lab register if the test was done or not, and provide a reason if it was not done.
- Record if the control line is present (= valid result): "Yes" or "No".
- Record if background is still present after completion of the procedure: "Yes" or "No".

- If the control line was not present for the first test (= invalid results) or if there was a strong background (= test difficult to interpret), and you have done a repeat test, tick the box “Repeat test”.
- Record if the control line is present for the repeat test: “Yes” or “No”.
- If the control line of the repeat test is not present, record the result as “Invalid”.
- Record if the background is visible for the repeat test: “Yes” or “No”.
- If there is still a background visible, interpret the test as “Negative” or “Positive”.
- Record the result of the test: “Negative”,” Positive” or “Invalid”.
- If the test is positive, record the intensity of the test line: “Faint, weak, medium or strong”.
- Put a paper with the name of the test, the date and Nidiag patient number, next to the test device. Take a photograph of the RDT and write the number of the photograph in the lab register.

3.8 Waste management

- Discard the used test and the tips in a biohazard waste container.

4. References

Patthabhi S et al. PloS NTD 2010 ; 4(9) : e822.

5. Records and archives

Appendices and forms to complete	
**Number**	**Title**
1	Lab register
2	CRF

6. Document History

Revision	
SOP-WP2-LAB-06-V01-19Sep2012	Initial version
SOP-WP2-LAB-06-V02-14Jan2013	- Adaptation of “3.6 Interpretation of results” with adaptation of “Invalid results”, addition of “Background clearance” and “Intensity of the reaction”<br>- Adaptation of “3.7 Documentation of result”
SOP-WP2-LAB-06-V03-11Feb2013	- Correction of storage temperature<br>- Addition of reference to SOP-WP6-QUAL-05.
SOP-WP2-LAB-06-V04-20Aug2013	- Adaptation of storage conditions of the conjugate reagent<br>- Adaptation of “3.7 Documentation of results” with adaptation of time of interpretation and recording of results

Name and function	Date	Signature
Author		
Barbara Barbé	20/08/2013	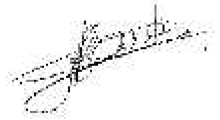
Review by		

Approved by		
Ninon Horié	28/08/2013	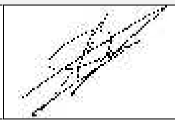

	**SOP Title:** Performing the rk39 IT LEISH (Bio-Rad)
	**Study title:** Evaluation of Rapid Diagnostic Tests (RDT) in association with clinical and laboratory predictors for the diagnosis of Neglected Tropical Diseases (NTD) in patients presenting with persistent fever (≥1 week) in Cambodia, Nepal, Democratic Republic of the Congo and Sudan

1. Scope and application

rK39 IT Leish (BIORAD) is a rapid test used for the detection of antibodies against *Leishmania*. It is an immune-chromatographic test in which the recombinant antigen rK39 derived from the members of the *Leishmania donovani* complex is coated in the test line of the dipstick. In case of the presence of *Leishmania donovani* antibodies in the sample, antibodies captured by the conjugate react with specific coated rK39 antigen. The reactions are demonstrated by the appearance of dark purple bands on the test line (L) and control line(C).

This SOP is applicable for patients with persistent fever enrolled in the fever syndrome of the NIDIAG study in **Nepal** and **Sudan**.

2. Responsibilities

**Function**	**Activities**
Laboratory technician	Performance of rk39 IT Leish (BIORAD), blinded to the results of the other VL diagnostic procedures (DAT, examination of LN or BM aspirate)

3. Procedures

3.1 Materials and samples

3.1.1 Materials

- rK39 IT LEISH (BIORAD) test package which contains
    - Test device (IT device) with dipstick, conjugate well, wash well
    - Well cover
    - Test buffer
    - Pipette (printed mark for 10µl) **(Do NOT use!)**
- Micropipette of 5-20 µl
- Yellow tips
- Timer
- Latex examination gloves
- Discarding jar

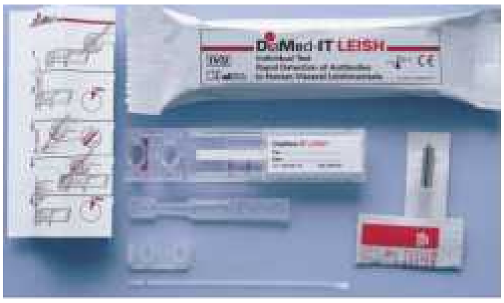

3.1.2 Samples

- Whole blood, EDTA: 10 µl
  (Refer to the SOP-WP2-LAB-44 for the collection of blood specimens)
  Comment: Use sample immediately

3.2 Procedures

- Check the expiry date of the test. If expired, use a new lot which has not yet expired.
- Tear open the aluminum package and take out all the material.
- Comment: Use the test within 15 minutes after opening
- Take the test device and place it horizontally on a flat surface
- Write the patient's ID and the date on the label part of the test device
- Tear open the ampoule of buffer, add 1 drop of buffer to the first well (conjugate well), marked with a colored line) and 4 drops to the second well (wash well).
- Comment: Always hold the buffer vial vertical while dispensing the drops.
- Allow to stand for 1 minute
- Mix-up gently the tube of whole blood
- Add 10µl of whole blood to the conjugate well with a micropipette.
- Stir gently with the upper end of the pipette and allow to stand for 1 minute. Discard the pipette into the discarding jar.
- Pull the IT device apart. Hold the device with the wells between thumb and forefinger and with the other hand, pull out the dipstick holder.
- Place the wells on a flat surface; insert the legs of the dipstick holder into the holes besides the conjugate well so that the dipstick reaches the bottom of the conjugate well.
- Allow to stand for 10 minutes in order to soak up the blood/conjugate mixture completely.
- Transfer the dipstick to the second well (wash well) and allow to stand for 10 minutes to make the field completely cleared of blood.
- Remove the dipstick from the wash well and click it back into the clear plastic piece. Close the wells with the well cover, break them off, and break the two legs off from the clear plastic piece. Discard them into discarding jar.
- Read the reaction and interpret the results

Summary of procedure:

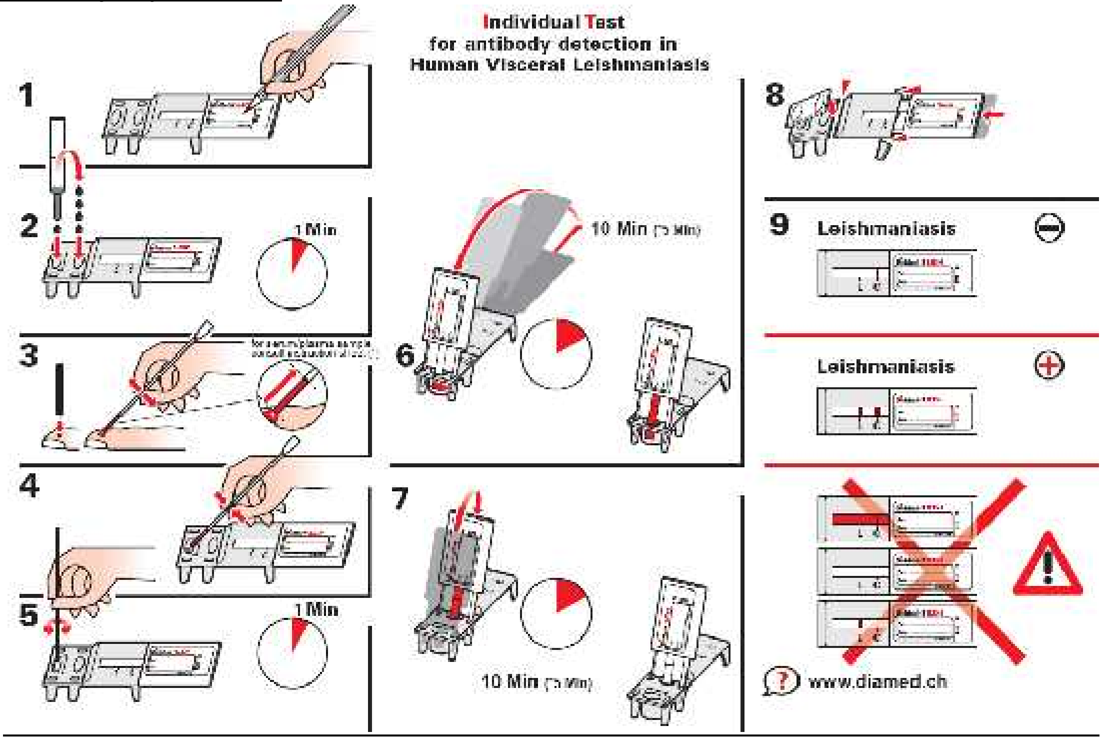

N.B. : For point 3 and 4, use an automatic pipette instead of the plastic pipette provided!

3.3 Interpretation

Invalid results

- **Results are valid** if a purple control band (C) is clearly visible
- **Results are not valid** if the control band is not visible even if the test band (L)is present (1,2)

1

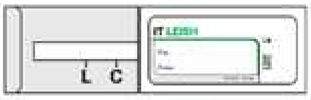

2

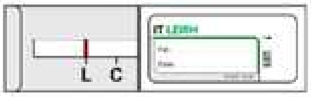

Action taken if invalid result:

If the test result is not valid, repeat the test once with a new rK39 IT LEISH (BIORAD) test device.
If the test result is still invalid, report as "invalid" in the CRF.

Interpretation of the reaction

Negative reaction:

- Dark purple band absent in the test line (L)
- Indicates the absence of detectable antibodies to Leishmaniaspp

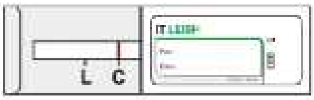

Positive reaction:

- Dark purple band in the test line(L)
- Indicates the presence of antibodies to Leishmania spp
- A faint line in the test band region is also considered as a positive reaction

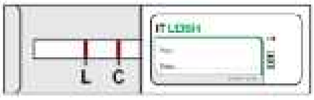

Examples:

Positive reaction

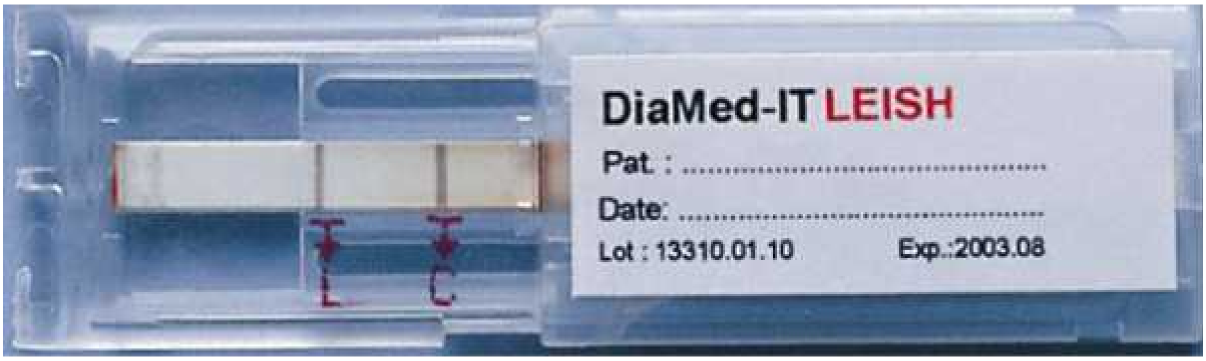

Negative reaction

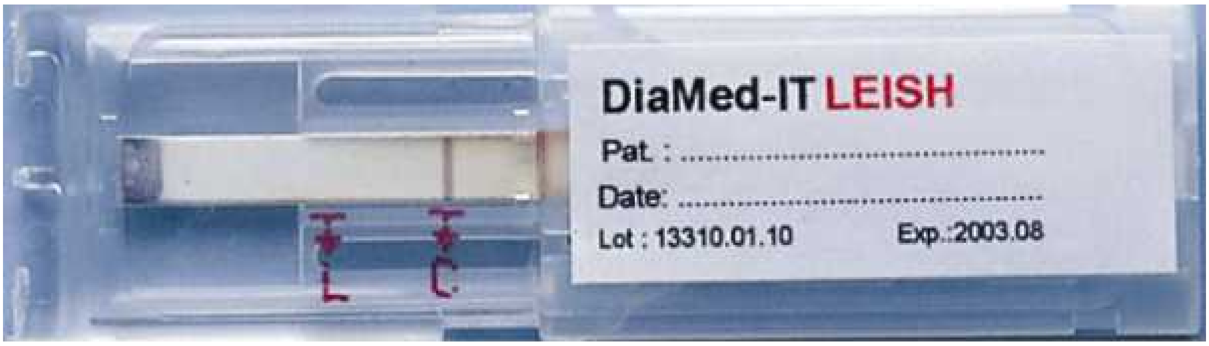

Background clearance

Record in the CRF if there is background visible after completion of the procedure:

Yes	Background visible (example 1,2)
No	No background visible (example 3)

1

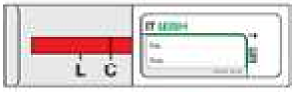

2

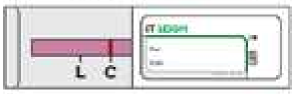

3

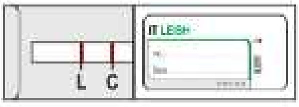

Action taken if strong background is present:

If there is background visible, repeat the test once with a new test device.

If the second test result still has background, report "background present" in the CRF, but interpret the test as "Negative" or "Positive".

Intensity of the reaction

If the reaction is POSITIVE, use the following scaling system to grade the intensity of the test line:

Faint	Test line visible, but very faint
Weak	Test line visible, but weaker than the control line
Medium	Test line intensity equals control line intensity
Strong	Test line more intense than control line

3.4 Documentation of result

- Nepal and Sudan use a lab register to record results. The results are then transcribed at a later stage in the CRF.
- Record if the test was done or not, and provide a reason if it was not done.
- Record if the control line is present (= valid result): "Yes" or "No".
- Record if background is still present after completion of the procedure: "Yes" or "No".
- If the control line was not present for the first test (= invalid results) or if there was a strong background (= test difficult to interpret), and you have done a repeat test, tick the box "Repeat test".
- Record if the control line is present for the repeat test: "Yes" or "No".
- If the control line of the repeat test is not present, record the result as "Invalid".
- Record if the background is visible for the repeat test: "Yes" or "No".
- If there is still a background visible, interpret the test as "Negative" or "Positive".
- Record the result of the test: "Negative"," Positive" or "Invalid".
- If the test is positive, record the intensity of the test line: "Faint, weak, medium or strong".
- Put a paper with the name of the test, the date and Nidiag patient number, next to the test device. Take a photograph of the RDT and write the number of the photograph in the lab register.

3.5 Precaution

- Do not use the kit beyond expiry date.
- If the package is damaged, use another test device.
- Use the test device immediately after it is opened.
- Do not reuse the test device.

3.6 Safety

- Handle all samples as potentially infectious.
- Practice safety precautions for handling and disposal of the infectious materials.

3.7 Storage

- Store the test kits at below 30°C.
- Do not freeze.
- Keep the device sealed until used
- Keep away from direct sunlight, moisture and heat.
- Maintain all the test kits at the same temperature and conditions.
- Refer to **SOP-WP6 -QUAL-05** for handling and storage of RDTs.

4. Records and archives

**Appendices and forms to complete**	
**Number**	**Title**
1	Lab register
2	CRF

5. Document History

**Revision**	
SOP-WP2-LAB-05-V01-19Sep2012	Initial version
SOP-WP2-LAB-05-V02-14Jan2013	- Adjustment of pipetting method<br>- Adaptation of “3.3 Interpretation” with adaptation of “Invalid results”, addition of “Background clearance” and “Intensity of the reaction”<br>- Addition of “3.4 Documentation of result”
SOP-WP2-LAB-05-V03-11Feb2013	- Adjustment storage temperature<br>- Reference to SOP-WP6-QUAL-05

**Name and function**	**Date**	**Signature**
Author		
Basudha Khanal	24/05/2012	
Barbara Barbé (revision 1 and 2)	11/02/2013	
Review by		
Philippe Gillet	11/02/2013	
Approved by		
Emilie Alirol	11/02/2013	

	**SOP Title:** Performing the Typhidot Rapid IgM (Reszon Diagnostics)
	**Study title:** Evaluation of Rapid Diagnostic Tests (RDT) in association with clinical and laboratory predictors for the diagnosis of Neglected Tropical Diseases (NTD) in patients presenting with persistent fever (≥1 week) in Cambodia, Nepal, Democratic Republic of the Congo and Sudan

1. Scope and application

Typhidot Rapid IgM is used as in vitro diagnostic test of typhoid fever. It is an immunochromatographic assay used for the detection of specific IgM antibodies against *Salmonella* Typhi in human blood/serum. Antibodies and reagents for capture of anti-*S.* Typhi IgM are immobilized onto cellulose nitrate membrane as test lines. When the test sample is added to the sample pad, it migrates upwards together with dye conjugated to *S.* Typhi antigens. Specific antibodies if present in the sample form an antibody-antigen complex with the conjugated antigens which are captured at the test window zone by the immobilized antibodies and reagents, giving a pink-purplish coloured band at the test line/s and at the control line. This SOP is applicable for the diagnosis of typhoid fever in the patients enrolled in the fever syndrome of NIDIAG study.

2. Responsibilities

Function	Activities
Technician	Performance of the Typhidot Rapid IgM test by complying with this procedure

3. Procedures

3.1 Materials and samples

3.1.1 Materials

- TYPHIDOT Rapid IgM test device
- Chase buffer
- Micropipette of 20-50 µl
- Microtips (2-100µl)
- Timer
- Latex examination gloves
- Discarding jar

3.1.2 Sample

- Whole blood, EDTA: 35 µl

(Refer to the SOP –WP6 –LAB 44 for the collection of blood specimens)
<u>Comment</u>: Use the sample immediately.

3.2 Procedure (see figure 1)

1. Bring the test kit and the chase buffer to room temperature.
2. Check the expiry date of the test. If expired, use a new lot which has not yet expired.
3. Open the pouch by cutting the seal.
4. Label the test device with the patients ID and the date.
5. Invert the EDTA tube gently for a couple of times.

6. Add 35µl of whole blood into the square sample well.
7. Immediately add 1 drop of buffer to the same well (square sample well).
   Comment: Always hold the buffer vial vertical while dispensing the drops.
8. Wait until the wet sample front reaches the area A.
   Comment: If the front does not reach area A within 5 minutes, but it has already reached the area between A and B, continue to the next step.
   If this is not the case after 5 minutes, repeat the test using a new cassette.
9. Add 3 drops of chase buffer, drop-by-drop in the oval buffer well.
   Comment: Let the buffer sip through between the drops.
10. Pull the clear tab at one site of the device (the side of the square sample well), until resistance is felt.
11. Add 1 drop of chase buffer to the square sample well.
12. Start timing: 15-20 minutes
13. Read the result after 15-20 minutes.
14. Interpret the result as indicated in the figure 2.

Figure 1: Diagrammatic Representation of Assay Procedure

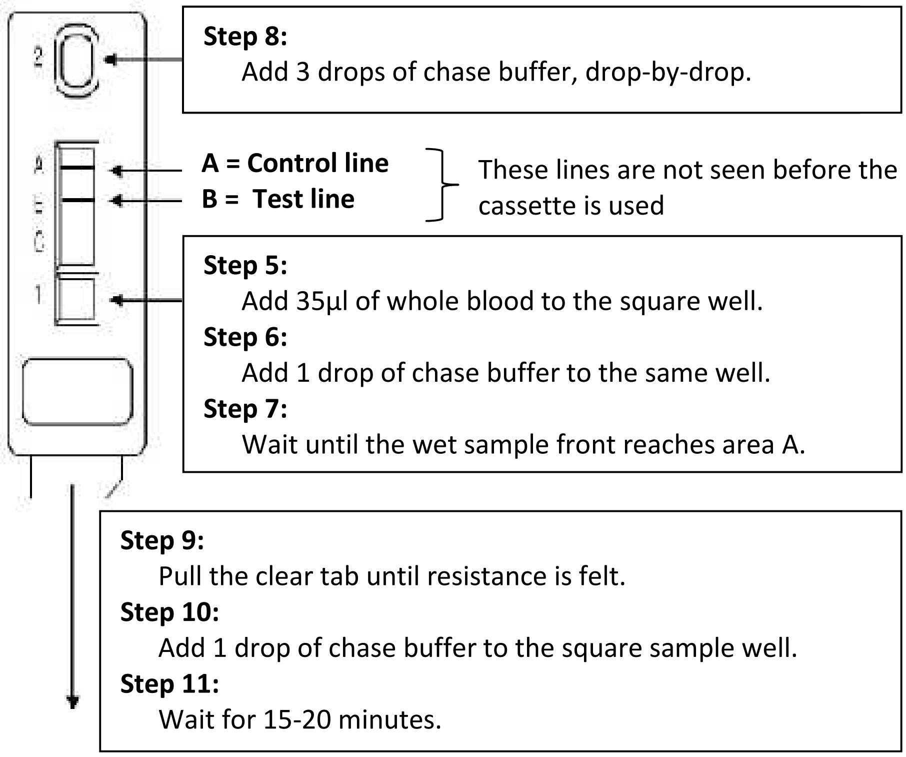

Interpretation (see figure 2)

- Any intensity of the band is considered positive

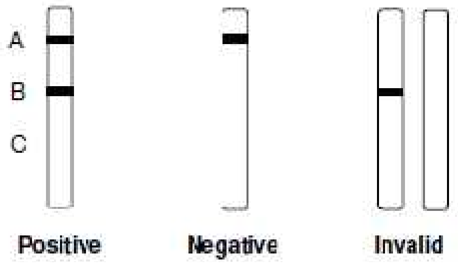

Figure 2: Interpretation of result

Positive result:

Pink-purplish coloured band at the control line (A) and test line (B).

Negative result:

Only pink-purplish coloured band at the control line (A) is visible.

Invalid result:

- No pink-purplish coloured band at control line(A)
- Pink-purplish coloured band present at test line (B) but not at control line (A)
- No pink-purplish coloured band at the test line (A) and control line(C)
- In case of invalid result, repeat once using a new test device.
  If the test result is still invalid, report as “invalid” in the CRF.

Background clearance

Record in the CRF if there is background visible after completion of the procedure:

Yes	Background visible
No	No background visible

If there is background visible, repeat the test once with a new test device.
If the second test result still has background, report “background present” in the CRF, but interpret the test as “Negative” or “Positive”.

Intensity of the reaction

If the reaction is POSITIVE, use the following scaling system to grade the intensity of the test line:

Faint	Test line visible, but very faint
Weak	Test line visible, but weaker than the control line
Medium	Test line intensity equals control line intensity
Strong	Test line more intense than control line

3.3 Documentation of results

- Most study sites (Nepal, Cambodia and Sudan) use a lab register to record results. The results are then transcribed at a later stage in the CRF. In DRC, the result may be recorded directly in the CRF.
- Record if the test was done or not, and provide a reason if it was not done.
- Record if the control line is present (= valid result): “Yes” or “No”.
- Record if background is still present after completion of the procedure: “Yes” or “No”.
- If the control line was not present for the first test (= invalid results) or if there was a strong background (= test difficult to interpret), and you have done a repeat test, tick the box “Repeat test”.
- Record if the control line is present for the repeat test: “Yes” or “No”.
- If the control line of the repeat test is not present, record the result as “Invalid”.
- Record if the background is visible for the repeat test: “Yes” or “No”.
- If there is still a background visible, interpret the test as “Negative” or “Positive” .
- Record the result of the test: “Negative”,” Positive” or “Invalid”.
- If the test is positive, record the intensity of the test line: “Faint, weak, medium or strong”.

- Put a paper with the name of the test, the date and Nidiag patient number, next to the test device. Take a photograph of the RDT and write the number of the photograph in the CRF or lab register.

3.4 Precautions

- Do not use the kit beyond expiry date.
- If the package is damaged, use another test device.
- Use the test device immediately after it is opened.
- Do not reuse the test device.

3.5 Safety

- Handle all samples as potentially infectious.
- Practice safety precautions for handling and disposal of the infectious materials.

3.6 Storage

- Store the test kits at 4-30°C, as per manufacturer's instructions.
- Keep the device sealed until used
- Keep away from direct sunlight, moisture and heat.
- Maintain all the test kits at the same temperature and conditions.
- Do not freeze the kits.
- Refer to **SOP-WP6 -QUAL-05** for handling and storage of RDTs.

4. References

1. Ivanoff B, Levine MM, Lambert PH (1994). Vaccination against typhoid fever: present status. Bull World Health Organ 72: 957-71.

2. Ismail A, Ong KH, Zainoodin SAK (1991). Demonstration of an antigenic protein specific for *Salmonella typhi.* Biochem Biophy Res Commun 181: 301-5.

3. Levine MM, Grados O, Gilman RH, Woodward WE, Solis-Plaza R,Waldman W (1978). Diagnostic value of the Widal test in areas endemic for typhoid fever. Am J Trop Med Hyg 27: 785-800.

5. Records and archives

**Appendices and forms to complete**	
**Number**	**Title**
1	Lab register
2	CRF

6. Document History

**Revision**	
SOP-WP2-LAB-10-V01-27Aug2012	Initial version
SOP-WP2-LAB-10-V02-14Jan2013	- Adaptation of "3.2 Procedure" with adaptation of the procedure from IG/IgM to IgM test.<br>- Adjustment of "Interpretation", "Invalid results" "and "test not interpretable"<br>- Addition of "Background clearance" and "Intensity of the reaction"<br>- Addition of "3.3 Documentation of result"

SOP-WP2-LAB-10-V03-11Feb2013	- Reference to SOP-WP6-QUAL-05

**Name and function**	**Date**	**Signature**
Authors		
Basudha Khanal	06/08/2012	
Barbara Barbé (revision 1 and 2)	11/02/2013	
Review by		
Philippe Gillet	11/02/2013	
Approved by		
Emilie Alirol	11/02/2013	

<table>
<tr><td rowspan="2"></td><td>SOP Title: Performing the S. Typhi IgM/IgG (SD Bioline)</td></tr>
<tr><td>Study title: Evaluation of Rapid Diagnostic Tests (RDT) in association with clinical and laboratory predictors for the diagnosis of Neglected Tropical Diseases (NTD) in patients presenting with persistent fever (≥1 week) in Cambodia, Nepal, Democratic Republic of the Congo and Sudan</td></tr>
</table>

1. Scope and application

Salmonella Typhi IgM/IgG(SD Bioline) is used as in vitro diagnostic test of typhoid fever. It is a rapid, immunochromatographic assay used for the simultaneous detection and differentiation of specific IgM and IgG antibodies against *Salmonella* Typhi in human blood. The test strip has three precoated lines, G (test line for *Salmonella* Typhi IgG), M (test line for *Salmonella* Typhi IgM) and C (Control line).When sample is added to the sample pad , anti-*Salmonella* Typhi IgG and IgM if present in the sample react with *Salmonella* Typhi proteins of the colloid gold conjugates and form a complex of antibodies a conjugates. This mixture then migrates along the length of the strip and is captured by the antibodies in the coated lines generating the purple band at the test lines G and/or M and C. This SOP is applicable for the diagnosis of typhoid fever in the patients enrolled in the fever syndrome of NIDIAG study.

2. Responsibilities

Function	Activities
Technician	Performance of the Rapid diagnostic test Salmonella typhi IgG/IgM(SD Bioline) by complying with this procedure

3. Procedures

3.1 Materials and samples

3.1.1 Materials

- Salmonella Typhi IgG/IgM(SD Bioline) Test strip
- Buffer
- Disposable test tube
- Microtips (0.5-10µl)
- Micropipette 1-5 µl
- Timer
- Latex examination gloves
- Discarding jar (biohazard waste container)

3.1.2 Samples

- Whole blood, EDTA: 1 µl
- (Refer to the SOP –WP2-LAB-44 for the collection of specimens including blood)
- Comment: Use the sample immediately. If not possible, store at 2-8°C for maximum 3 days.

3.2 Procedure

- Bring the kit to the room temperature.
- Check the expiry date of the test. If expired, use a new lot which has not yet expired.

- Take a tube and label with patient's ID and the date.
- Add 4 drops (120µl) of assay buffer to the disposable tube.
    - Comment: Always hold the buffer vial vertical while dispensing the drops.
- Invert the EDTA tube gently for a couple of times.
- Transfer 1µl of the sample to the assay buffer with a micropipette. Stir the assay buffer gently for adequate mixing with the sample.
- Remove test strip from the foil pouch.
- Open the desiccant pouch and check the desiccant for any colour change (orange to green). If green desiccant beads are present, discard the test device and use another device for testing.
- Hold the strip vertically, insert into the tube containing diluted specimen
- Allow to stand for 15 minutes.
- Read the test band on the strip and interpret the result as indicated below.
    - If the test band is faint then read the result again at 30 minutes.

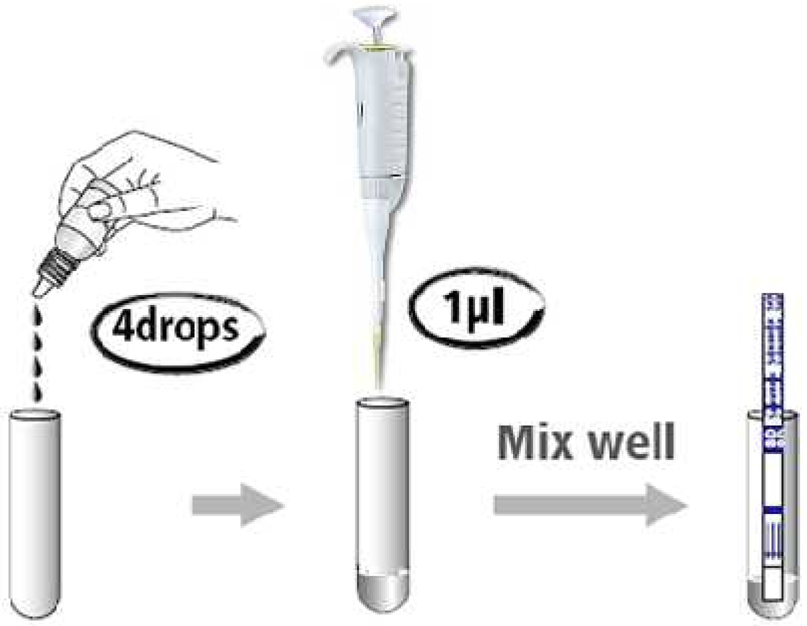

3.3 Interpretation

3.3.1. Positive results:

- **IgM positive**
    - Purple coloured control line (C) and IgM line (M) are visible at the test strip as indicated in figure

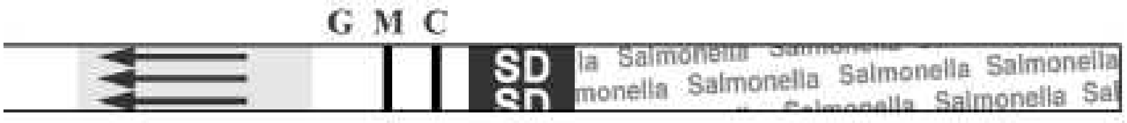

- **IgM and IgG positive**
    - Purple colroured control lines (C), IgM line (M) and IgG line (G) are visible at the test strip as indicated in figure

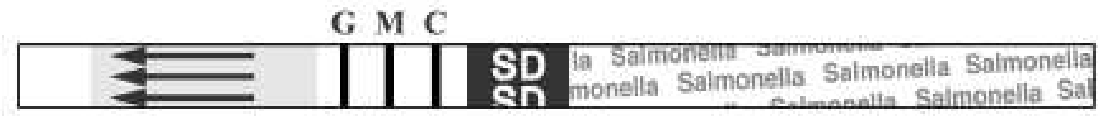

- **IgG positive**
  - o Purple coloured control line (C) and IgG line (G) are visible at the test strip.

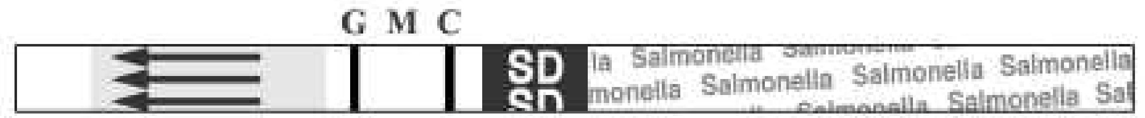

3.3.2. Negative result:

- o Purple coloured control line(C) is visible at the test strip.

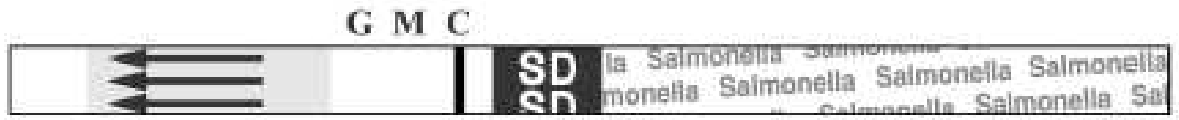

3.3.3. Invalid results:

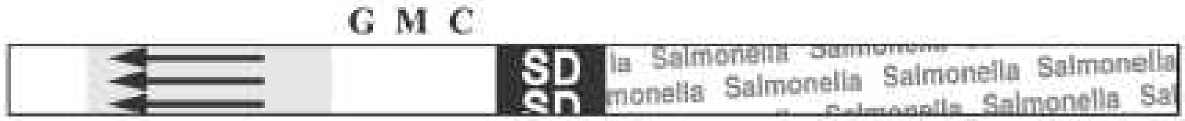

- o No purple coloured control line is visible.

If the test result is not valid, repeat the test once with a new test strip.

If the test result is still invalid, report as “invalid” in the CRF.

3.3.4. Background clearance

Record in the CRF if there is background visible after completion of the procedure:

Yes Background visible

No No background visible

If there is background visible, repeat the test once with a new test device.
If the second test result still has background, report “background present” in the CRF, but interpret the test as “Negative” or “Positive”.

3.3.5. Intensity of the reaction

If the reaction is POSITIVE, use the following scaling system to grade the intensity of each test line:

Faint	Test line visible, but very faint
Weak	Test line visible, but weaker than the control line
Medium	Test line intensity equals control line intensity
Strong	Test line more intense than control line

Do this for the test line of IgM and IgG.

3.4 Documentation of result

- Most study sites (Nepal, Cambodia and Sudan) use a lab register to record results. The results are then transcribed at a later stage in the CRF. In DRC, the result may be recorded directly in the CRF.
- Record if the test was done or not, and provide a reason if it was not done.
- Record if the control line is present (= valid result): “Yes” or “No”.
- Record if background is still present after completion of the procedure: “Yes” or “No”.
- If the control line was not present for the first test (= invalid results) or if there was a strong background (= test difficult to interpret), and you have done a repeat test, tick the box “Repeat test”.
- Record if the control line is present for the repeat test: “Yes” or “No”.
- If the control line of the repeat test is not present, record the result as “Invalid”.
- Record if the background is visible for the repeat test: “Yes” or “No”.
- If there is still a background visible, interpret the test as “Negative” or “Positive .
- Record the result of the test: “Negative”,” Positive” or “Invalid”.
- If the test is positive, record the intensity of the test lines (IgG and IgM): “Faint, weak, medium or strong”.
- Put a paper with the name of the test, the date and Nidiag patient number, next to the test strip. Take a photograph of the RDT and write the number of the photograph in the CRF or lab register.

3.5 Precaution

- Do not use the kit beyond expiry date.
- If the package is damaged, use another test strip.
- Use the test device immediately after it is opened.
- Do not reuse the test strip.
- Handle the assay buffer carefully as it contains sodium azide as preservative.

3.6 Safety

- Handle all samples as potentially infectious.
- Practice safety precautions for handling and disposal of the infectious materials.

3.7 Storage

- Store the test kits at below 30°C.
- Do not freeze.
- Keep the device sealed until used
- Keep away from direct sunlight, moisture and heat.
- Maintain all the test kits at the same temperature and conditions.
- Refer to **SOP-WP6 -QUAL-05** for handling and storage of RDTs.

4. Records and archives

**Appendices and forms to complete**	
**Number**	**Title**
1	Lab register
2	CRF

5. Document History

Revision	
SOP-WP2-LAB-11-V01-27Aug2012	Initial version
SOP-WP2-LAB-11-V02-14Jan2013	- Adaptation of “3.2 Procedure” with adaptation of “Interpretation”, “Invalid results”, addition of “Background clearance” and “Intensity of the reaction”<br>- Addition of “3.3 Documentation of result”
SOP-WP2-LAB-11-V03-11Feb2013	- Adjustment storage temperature<br>- Reference to SOP-WP6-QUAL-05

Name and function	Date	Signature
Authors		
Basudha Khanal	15/05/2012	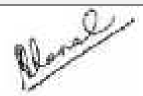
Barbara Barbé	11/02/2013	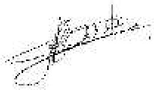
Review by		
Philippe Gillet	11/02/2013	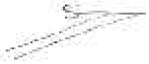
Approved by		
Emilie Alirol	11/02/2013	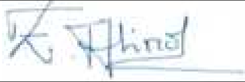

	**SOP Title:** Performing the Test-it Typhoid IgM (Life Assay)
	**Study title:** Evaluation of Rapid Diagnostic Tests (RDT) in association with clinical and laboratory predictors for the diagnosis of Neglected Tropical Diseases (NTD) in patients presenting with persistent fever (≥1 week) in Cambodia, Nepal, Democratic Republic of the Congo and Sudan

1. Scope and application

Test it Typhoid IgM is a lateral flow immunochromatographic assay used for the rapid serodiagnosis of typhoid fever. A lipopolysaccharide antigen (LPS) obtained from a culture of *Salmonella enterica* serotype Typhi is immobilised in a discrete line on a porous nitrocellulose membrane located in the test zone (T). Anti-human IgM antibodies labelled with red colloidal gold particles are used as mobile detection reagent. When the test sample is added to the sample well (S), it migrates upwards together with the mobile detection reagent. *Salmonella* Typhi specific IgM antibodies, if present in the sample will bind to the immobilised antigen and will produce a red line at the test zone (T). This SOP is applicable for the diagnostic evaluation of typhoid fever in the patients enrolled in the persistent fever syndrome of NIDIAG study in Cambodia, Nepal, DRC and Sudan.

Note: This SOP is **only** applicable for the lots of Test-it Typhoid IgM (Life Assay) tests that indicate in the package insert that **a whole blood volume of 5 µl** needs to be used. Verify the package insert of the kits before testing!

2. Responsibilities

Function	Activities
Laboratory Technician	- Performing the RDT Test It Typhoid IgM (Life Assay) blinded to the results of reference tests<br>- Reporting the results in the laboratory register and in the Lab Report Form of the CRF

3. Procedures

3.1 Precautions

- Do not use the kit beyond expiry date.
- If the package is damaged, use another test device.
- Use the test device immediately after it is opened.
- Do not reuse the test device.

3.2 Safety

- Handle all samples as potentially infectious. Wear gloves during the procedure.
- Practice safety precautions for handling and disposal of the infectious materials.

3.3 Storage

- Store the test kits at +4 to 28°C, as per manufacturer's instructions.
- Keep the device sealed until used
- Keep away from direct sunlight, moisture and heat.
- Maintain all the test kits at the same temperature and conditions.
- Refer to **SOP-WP6 -QUAL-05** for handling and storage of RDTs.

3.4 Materials and samples

3.4.1 Materials provided with the kit

- Test it Typhoid IgM test device (Life Assay)
- Running buffer
- Plastic pipette (Do NOT use!)

3.4.2 Additional materials required

- Micropipette of 5-20 µl
- Yellow tips
- Timer
- Latex examination gloves
- Discarding jar

3.4.3 Samples

- Whole blood, EDTA: 5 µl
  (Refer to the SOP for the collection of blood)
- Comment: Use sample immediately.

3.5 Procedure

- Bring the test kit and the running buffer to room temperature.
- Check the expiry date of the test. If expired, use a new lot which has not yet expired.
- Open the package and place the device with the test window facing upwards.
- Check the colour of the desiccant.
- Proceed for testing if the colour is still orange.
- Discard the test and use a new test device if the colour of the desiccant has changed to green.
- Label the test device with the patients ID and the date.
- Invert the EDTA tube gently for a couple of times.
- Draw up 5 µl of whole blood with a micropipette.
- Add the blood to the round sample port (S).
- Add 4 drops of running buffer to the same sample post.
  Comment: Always hold the buffer vial vertical while dispensing the drops.
- Read the result at 15 minutes. Do not read the result after 30 minutes.
- Interpret the result as indicated below.

3.6 Interpretation of the results:

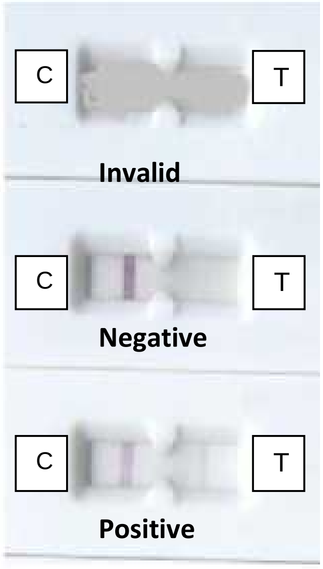

Invalid: Pink control line is absent ⇒ Repeat once using a new test.

If the repeat test is still invalid, report as "Invalid" in the CRF.

Negative result: Pink line at the control zone (C)

= **Negative** for *Salmonella* Typhi IgM antibodies.

Positive result: Pink line at the control zone (C) and Test zone (T)

= **Positive** for *Salmonella* Typhi IgM antibodies.

Background clearance

Record in the CRF if there is background visible after completion of the procedure:

Yes	Background visible
No	No background visible

If there is background visible, repeat the test once with a new test device.
If the second test result still has background, report "background present" in the CRF, but interpret the test as "Negative" or "Positive".

Intensity of the reaction

If the reaction is POSITIVE, use the following scaling system to grade the intensity of the test line:

Faint	Test line visible, but very faint
Weak	Test line visible, but weaker than the control line
Medium	Test line intensity equals control line intensity
Strong	Test line more intense than control line

3.7 Documentation of the result

- Most study sites (Nepal, Cambodia and Sudan) use a lab register to record results. The results are then transcribed at a later stage in the CRF. In DRC, the result may be recorded directly in the CRF.
- Record if the test was done or not, and provide a reason if it was not done.
- Record if the control line is present (= valid result): "Yes" or "No".
- Record if background is still present after completion of the procedure: "Yes" or "No".
- If the control line was not present for the first test (= invalid results) or if there was a strong background (= test difficult to interpret), and you have done a repeat test, tick the box "Repeat test".
- Record if the control line is present for the repeat test: "Yes" or "No".
- If the control line of the repeat test is not present, record the result as "Invalid".

- Record if the background is visible for the repeat test: “Yes” or “No”.
- If there is still a background visible, interpret the test as “Negative” or “Positive”.
- Record the result of the test: “Negative”,” Positive” or “Invalid”.
- If the test is positive, record the intensity of the test line: “Faint, weak, medium or strong”.
- Put a paper with the name of the test, the date and Nidiag patient number, next to the test device. Take a photograph of the RDT and write the number of the photograph in the CRF or lab register.

3.8 Waste management

- Discard the used test and the plastic pipette in a biohazard waste container.

4. Records and archives

Appendices and forms to complete	
**Number**	**Title**
1	Lab register
2	CRF

5. References

Pastoor R,Hattab M,Abdoel TH,Smits H.A simple ,rapid and affordable point of care test for the serodiagnosis of typhoid fever.Diagn Microbial Infect Dis 61;2008:129-34.

6. Document History

Revision	
SOP-WP2-LAB-12-V01-27Aug2012	Initial version
SOP-WP2-LAB-12-V02-14Jan2013	- Adjustment of pipetting method<br>- Adaptation of “3.6 Interpretation” with adaptation of “Invalid results”, addition of “Background clearance” and “Intensity of the reaction”<br>- Adjustment of “4. Documentation of the result”
SOP-WP2-LAB-12-V03-11Feb2013	- Reference to SOP-WP6-QUAL-05
SOP-WP2-LAB-12-V04-08Jan2014	- Adaptation of sample volume from 10 µl to 5 µl whole blood

Name and function	Date	Signature
Author		
Barbara Barbé	08/01/2014	
Reviewed by		
Approved by		
Ninon Horié	10/01/2014	

	**SOP Title:** Performing the Test-it Leptospirosis IgM (Life Assay)
	**Study title:** Evaluation of Rapid Diagnostic Tests (RDT) in association with clinical and laboratory predictors for the diagnosis of Neglected Tropical Diseases (NTD) in patients presenting with persistent fever (≥1 week) in Cambodia, Nepal, Democratic Republic of the Congo and Sudan

1. Scope and application

Test it Leptospira IgM is a lateral flow immunochromatographic assay used for the rapid serodiagnosis of leptospirosis, detecting *Leptospira* specific IgM antibodies. A lipopolysaccharide antigen (LPS) prepared from a culture of *Leptospira* is immobilised in a discrete line on a porous nitrocellulose membrane located in the test zone (T). Detection reagent consists of anti-human IgM antibodies labelled with red colloidal gold particles. When the test sample is added to the sample well (S), it migrates upwards together with the detection reagent. *Leptospira* specific IgM antibodies, if present in the sample will bind to the immobilised LPS antigen and will produce a red line at the test zone (T).

This SOP is applicable for the diagnostic evaluation of leptospirosis in the patients enrolled in the persistent fever syndrome of NIDIAG study in Cambodia, Nepal, DRC and Sudan.

2. Responsibilities

Function	Activities
Laboratory Technician	Performing the RDT Test It Leptospira IgM (Life Assay) blinded to the results of reference tests<br>Reporting the results in the laboratory register and in the Lab Report Form of the CRF

3. Procedures

3.1 Precautions

- Do not use the kit beyond expiry date.
- If the package is damaged, use another test device.
- Use the test device immediately after it is opened.
- Do not reuse the test device.

3.2 Safety

- Handle all samples as potentially infectious. Wear gloves during the procedure.
- Practice safety precautions for handling and disposal of the infectious materials.

3.3 Storage

- Store the test kits at +4 to 28°C, as per manufacturer's instructions.
- Keep the device sealed until used
- Keep away from direct sunlight, moisture and heat.
- Maintain all the test kits at the same temperature and conditions.
- Refer to **SOP-WP6 -QUAL-05** for handling and storage of RDTs.

3.4 Materials and samples

3.4.1 Materials provided with the kit

- Test it Leptospira IgM test device (Life Assay)
- Running buffer
- Plastic pipette (Do NOT use!)

3.4.2 Additional materials required

- Micropipette of 5-20 µl
- Yellow tips
- Timer
- Latex examination gloves
- Discarding jar

3.4.3 Sample

- Whole blood, EDTA: 10 µl
  (Refer to the SOP for the collection of blood)

Comment: use sample immediately.

3.5 Procedure

- Bring the test kit and the running buffer to room temperature.
- Check the expiry date of the test. If expired, use a new lot which has not yet expired.
- Open the package and place the device with the test window facing upwards.
- Check the colour of the desiccant:
- Proceed for testing if the colour is still orange.
- Discard the test if the colour of the desiccant has changed to green.
- Label the test device with the patients ID and the date.
- Invert the EDTA tube gently for a couple of times.
- Draw up 10µl of whole blood with a micropipette.
- Add the blood to the sample well (S).
- Add 4 drops of running buffer to the sample well.
- Comment: Always hold the buffer vial vertical while dispensing the drops.
- Read the result at 15 minutes. Do not read the result after 30 minutes.
- Interpret the result as indicated below.

3.6 Interpretation of the results

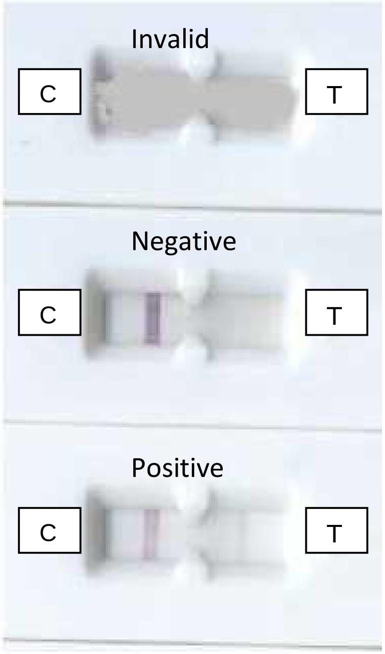

Invalid: Pink ontrol line is absent ⇒ Repeat once using new test

If the repeat test is still invalid, report as “Invalid” in the CRF.

Negative result: Pink line at the control zone (C)

= **Negative** for *Leptospira* IgM antibodies

Positive result: Pink line at the control zone (C) and Test zone (T)

= **Positive** for *Leptospira* IgM antibodies.

Background clearance

Record in the CRF if there is background visible after completion of the procedure:

Yes	Background visible
No	No background visible

If there is background visible, repeat the test once with a new test device.
If the second test result still has background, report “background present” in the CRF, but interpret the test as “Negative” or “Positive”.

Intensity of the reaction

If the reaction is POSITIVE, use the following scaling system to grade the intensity of the test line:

Faint	Test line visible, but very faint
Weak	Test line visible, but weaker than the control line
Medium	Test line intensity equals control line intensity
Strong	Test line more intense than control line

3.7 Documentation of the result

- Most study sites (Nepal, Cambodia and Sudan) use a lab register to record results. The results are then transcribed at a later stage in the CRF. In DRC, the result may be recorded directly in the CRF.
- Record if the test was done or not, and provide a reason if it was not done.
- Record if the control line is present (= valid result): “Yes” or “No”.
- Record if background is still present after completion of the procedure: “Yes” or “No”.
- If the control line was not present for the first test (= invalid results) or if there was a strong background (= test difficult to interpret), and you have done a repeat test, tick the box “Repeat test”.
- Record if the control line is present for the repeat test: “Yes” or “No”.
- If the control line of the repeat test is not present, record the result as “Invalid”.
- Record if the background is visible for the repeat test: “Yes” or “No”.
- If there is still a background visible, interpret the test as “Negative” or “Positive”.
- Record the result of the test: “Negative”,” Positive” or “Invalid”.
- If the test is positive, record the intensity of the test line: “Faint, weak, medium or strong”.
- Put a paper with the name of the test, the date and Nidiag patient number, next to the test device. Take a photograph of the RDT and write the number of the photograph in the CRF or lab register.

3.8 Waste management

- Discard the used test and the plastic pipette in a biohazard waste container.

4. References

1. Smits HL et al. Lateral flow assay for rapid serodiagnosis of human leptospirosis .Clin Diagn Lab Immunol.2001; 8:166-169.
2. Levett PN. Leptospirosis .Clin Microbiol Rev.2001;14:296-326

5. Records and archives

Appendices and forms to complete	
**Number**	**Title**
1	Lab register
2	CRF

6. Document History

Revision	
SOP-WP2-LAB-13-V01-27Aug2012	Initial version
SOP-WP2-LAB-13-V02-14Jan2013	- Adjustment of pipetting method<br>- Adaptation of "3.6 Interpretation" with adaptation of "Invalid results", addition of "Background clearance" and "Intensity of the reaction"<br>- Addition of "3.7. Documentation of the result"
SOP-WP2-LAB-13-V03-11Feb2013	- Adjustment storage temperature<br>- Reference to SOP-WP6-QUAL-05

Name and function	Date	Signature
Authors		
Basudha Khanal	06/08/2012	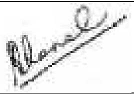
Barbara Barbé (revision 1 and 2)	11/02/2013	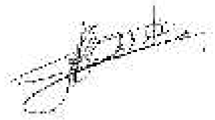
Reviewed by		
Philippe Gillet	11/02/2013	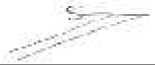
Approved by		
Emilie Alirol	11/02/2013	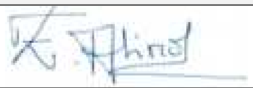

	**SOP Title:** Performing the Leptospira IgG/IgM (SD Bioline)
	**Study title:** Evaluation of Rapid Diagnostic Tests (RDT) in association with clinical and laboratory predictors for the diagnosis of Neglected Tropical Diseases (NTD) in patients presenting with persistent fever (≥1 week) in Cambodia, Nepal, Democratic Republic of the Congo and Sudan

1. Scope and application

Leptospira IgG/IgM (SD Bioline) is a rapid solid phase immunochromatographic assay used for the qualitative and differential detection of specific IgG and/or IgM antibodies to *Leptospira interrogans* in human serum or plasma. The test strip has three precoated lines, G (test line for *L. interrogans* IgG), M (test line for *L. interrogans* IgM) and (C) control line.

When sample is added to the sample well, IgG and IgM antibodies to *L. interrogans* if present, react with the antigen Leptospira lysate and the conjugate (mouse antileptospira –gold conjugate). This mixture then migrates along the length of the strip and is captured by the monoclonal anti-human IgG coated in the G and/or monoclonal anti-human IgM coated in the M generating pink-purple colored line/s. Absence of IgG and/or IgM antibodies to *L. interrogans* does not produce color at the test lines. Control line is for the procedural control which should always appear after addition of the sample indicating the proper performance of the test strip and the reagents. This SOP is applicable for the diagnostic evaluation of leptospirosis in the patients enrolled in the persistent fever syndrome of NIDIAG study in Cambodia, Nepal, DRC and Sudan.

2. Responsibilities

Function	Activities
Laboratory Technician	Performance of the RDT Test It Leptospira IgG/IgM (SD Bioline) blinded to the results of reference tests and reporting the results in the laboratory register and in the Lab Report Form of the CRF

3. Procedures

3.1 Precautions

- Do not use the kit beyond expiry date.
- Use the test device immediately after it is opened.
- If the package is damaged, use another test device.
- Do not reuse the test device.
- Handle the assay diluent carefully as it contains sodium azide as preservative.

3.2 Safety

- Handle all samples as potentially infectious.
- Practice safety precautions for handling and disposal of infectious materials.

3.3 Storage

- Store the test kits below 30°C.
- Do not freeze.
- Keep the device sealed until use.

- Keep away from direct sunlight; moisture and heat.
- Maintain all the test kits at the same temperature and conditions.
- Refer to **SOP-WP6 -QUAL-05** for handling and storage of RDTs.

3.4 Materials and samples

3.4.1 Materials included in the kit

- Leptospira IgG/ IgM test device (SD Bioline)
- 1 bottle of assay diluent
- 5µl capillary pipette (Do NOT use!)

3.4.2 Additional materials required

- Timer
- Latex examination gloves
- Micropipette of 5-20 µl
- Yellow tips
- Discarding jar (biohazard waste container)

3.4.3 Samples

- Serum : 5 µl
  (Refer to the SOP–WP2-LAB-44 for the collection of specimen)
- Comment: Use the sample immediately. If not possible, refrigerate at 2-8°C for maximum 2 weeks. If longer storage is necessary, freeze at -20°C.
- Bring the samples to room temperature before use.

3.5 Procedure

- Bring the test kit and the assay diluent to room temperature.
- Check the expiry date of the test. If expired, use a new lot which has not yet expired.
- Remove the test device from the foil pouch and place it on flat dry surface.
- Open the desiccant pouch and check the desiccant for any colour change (orange to green). If green desiccant beads are present, discard the test device and use another device for testing.
- Label the test device with the patients ID and the date.
- Invert the EDTA tube gently for a couple of times.
- Draw up 5µl of serum with the micropipette.
- Add the sample to the sample well (S).
- Add 4 drops of assay diluent to the round shaped assay diluent well.
- Comment: Always hold the buffer vial vertical while dispensing the drops.
- Wait for 20 minutes. Do NOT read the result afterwards.
- Read and interpret the result as indicated in the figure.

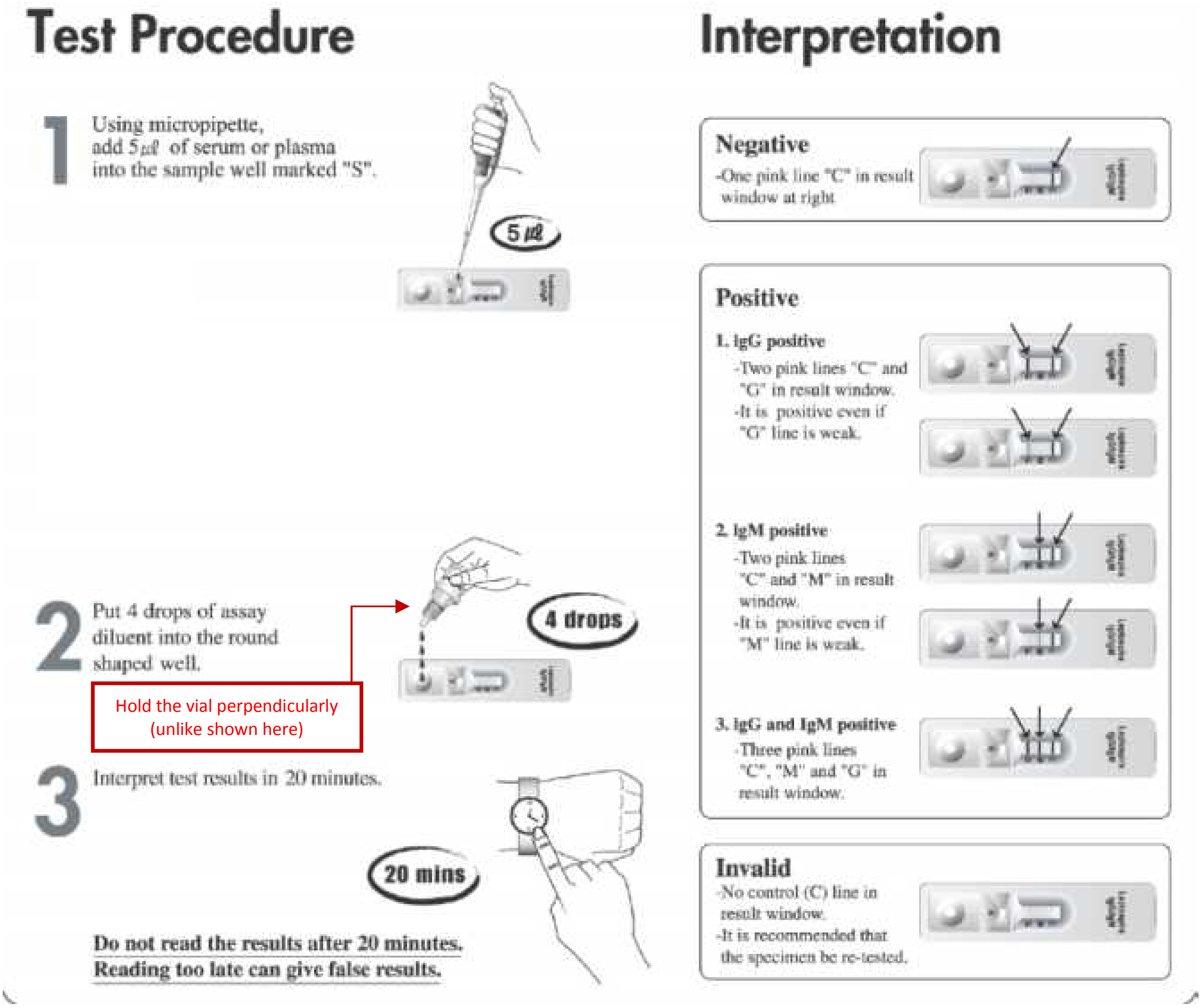

Interpretation of the results:

Positive result

IgM Positive

Pink-purple control line (C) and IgM line (M) visible on the test device. This is positive for IgM antibody to *L. interrogans.*

IgG Positive

Pink-purple control line (C) and IgG line (G) visible on the test device. This is positive for IgG antibody to *L. interrogans.*

IgM and IgGPositive

Pink-purple control line (C), IgM line (M) and IgG line (G) visible on the test device. This is positive for IgM and IgG antibodies to *L. interrogans.*

Negative result

ONLY a pink-purple control line (C) is visible at the test device. It is negative for IgG and IgM antibodies to *L. interrogans*.

Invalid result

Pink-purple control line is absent ⇒ Repeat once using new test device.

If the repeat test is still invalid, report as “Invalid” in the CRF.

Background clearance

Record in the CRF if there is background visible after completion of the procedure:

Yes	Background visible
No	No background visible

If there is background visible, repeat the test once with a new test device.
If the second test result still has background, report “background present” in the CRF, but interpret the test as “Negative” or “Positive”.

Intensity of the reaction

If the reaction is POSITIVE, use the following scaling system to grade the intensity of each test line:

Faint	Test line visible, but very faint
Weak	Test line visible, but weaker than the control line
Medium	Test line intensity equals control line intensity
Strong	Test line more intense than control line

Do this for the test line of IgM and IgG.

3.6 Documentation of result

- Most study sites (Nepal, Cambodia and Sudan) use a lab register to record results. The results are then transcribed at a later stage in the CRF. In DRC, the result may be recorded directly in the CRF.
- Record if the test was done or not, and provide a reason if it was not done.
- Record if the control line is present (= valid result): “Yes” or “No”.
- Record if background is still present after completion of the procedure: “Yes” or “No”.
- If the control line was not present for the first test (= invalid results) or if there was a strong background (= test difficult to interpret), and you have done a repeat test, tick the box “Repeat test”.
- Record if the control line is present for the repeat test: “Yes” or “No”.
- If the control line of the repeat test is not present, record the result as “Invalid”.
- Record if the background is visible for the repeat test: “Yes” or “No”.
- If there is still a background visible, interpret the test as “Negative” or “Positive” .
- Record the result of the test: “Negative”,” Positive” or “Invalid”.
- If the test is positive, record the intensity of the test lines (IgG and IgM): “Faint, weak, medium or strong”.
- Put a paper with the name of the test, the date and Nidiag patient number, next to the test device. Take a photograph of the RDT and write the number of the photograph in the CRF or the lab register.

3.7 Waste management

- Discard the used test and the plastic pipette in a biohazard waste container.

4. Records and archives

**Appendices and forms to complete**	
**Number**	**Title**
1	Lab register
2	CRF

5. References

1. Smits HL,Eapen CK,Sugathan S,Kuriakose M,Gasem MH,Yersin C,Sasaki D,Pujianto B,Vestering M,Abdoel TH,Gussenhoven GC.Lateral flow assay for rapid serodiagnosis of human leptospirosis.Clin Diagn Lab Immunol.2001; 8:166-169.
2. Bajani MD,Ashford DA,Bragg SL,Woods SW,Aye T,Spiegel RA,Plikaytis BD,Perkins BA,Phelan M,Levett PN,Weyant RS.Evaluation of four commercially available rapid serologic tests for diagnosis of leptospirosis.J Clin Microbiol 2003;41(2):803-9.

6. Document History

**Revision**	
SOP-WP2-LAB-14-V01-<br>12Nov2012	Initial version
SOP-WP2-LAB-14-V02-<br>14Jan2013	- Adaptation of “3.5 Procedure” with adaptation of “Interpretation”, “Invalid results”, addition of “Background clearance” and “Intensity of the reaction”<br>- Adaptation of “3.6 Documentation of result”
SOP-WP2-LAB-14-V03-<br>11Feb2013	- Adjustment storage temperature<br>- Reference to SOP-WP6-QUAL-05

**Name and function**	**Date**	**Signature**
Authors		
Basudha Khanal	15/10/2012	
Barbara Barbé (revision 1 and 2)	11/02/2013	
Reviewed by		
Philippe Gillet	11/02/2013	
Approved by		
Emilie Alirol	11/02/2013	

	**SOP Title:** Blood : RDT HIV Determine
	**Project/Study:** Evaluation of Rapid Diagnostic Tests (RDT) in association with clinical and laboratory predictors for the diagnosis of Neglected Tropical Diseases (NTD) in patients presenting with persistent fever (≥1 week) in Cambodia, Nepal, Democratic Republic of the Congo and Sudan

1. Scope and application

This document provides the instructions to perform the immuno-chromatographic rapid test Determine HIV-1/2. This test detects antibodies against VIH-1 and VIH-2 in serum, plasma or whole blood and constitutes a valuable tool to diagnose HIV.
Remark : A positive test result always requires confirmation.

2. Responsibilities

Function	Activities
Laboratory technician	• Blood collection<br>• Performing the test<br>• Interpretation of the test result<br>• Registration of the test result

3. Procedures

3.1 Precautions

- All blood samples are potentially infectious. Please respect universal precautions. USE DISPOSABLE GLOVES DURING THE WHOLE PROCEDURE!
- Do not use the kit beyond expiry date.
- If the package is damaged, use another kit.
- Use the test strip immediately after it is opened.
- Do not reuse the test strip.

3.2 Materials and samples

3.2.1. Material provided in the kit and storage

- Tests Determine HIV-1/2 contain recombinant HIV-1/2 antigens and synthetic peptides.
  Storage : between 2 and 30°C.
  Remark : The buffer is not included in the kit. Please order the buffer if using the test with whole blood.
- Brochure

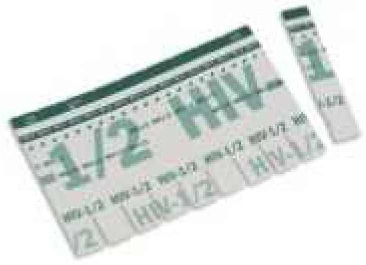

3.2.2. Required supplementary material

- Chase buffer, 2,5 ml (to be used with whole blood)
- Storage : between 2 and 30°C.
- Non sterile gloves, disposable
- Marker
- Micropipettes of 50 µl
- Pipet tips
- Timer
- « bio-hazard » container

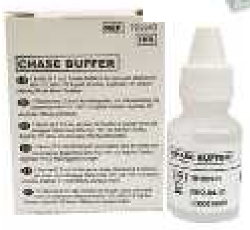

3.2.3. Sample to be examined

- Whole blood (collected with EDTA): 50 µl
- Storage:
- Whole blood: Execute the test immediately or store at 2-8°C up to 7 days. DO NOT FREEZE whole blood samples. Repeated freezing and thawing can affect the test results.

Remark : Let the samples adjust to room temperature before performing the test.

3.3 Test execution

3.3.1. Internal quality control

A control line « C » is included in the system to validate the test. The result is invalid if the control line does not appear by the end of the test.

3.3.2. Procedure

1. Let all samples and reagents adjust to room temperature.
2. Put on disposable gloves.
3. Check the expiry date. Never use an expired test.
4. Open the package and remove the carton with the 10 tests.
5. Check the expiry date of the tests. Detach the number of necessary tests from the carton containing the 10 tests, starting from the right to preserve the lot number and the expiry date on the left of the carton.
6. Immediately close the package.
7. Identify the test with the patient number and note the date.
8. Remove the plastic cover of every test
9. Using a micropipette, distribute 50 µl of the sample on the sample area (arrows).
10. For **whole blood**:
    - 10.1 Wait one minute
    - 10.2 Add one drop of chasing buffer on the sample area
      - Comment: Always hold the buffer vial vertical while dispensing the drops.
11. Start the timer (15 minutes).
12. Read the result after 15 minutes (maximum 60 minutes) (See « 3.4 test interpretation »).
13. Record the test result in the CRF

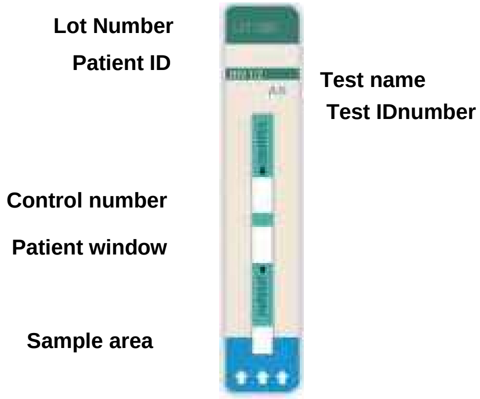

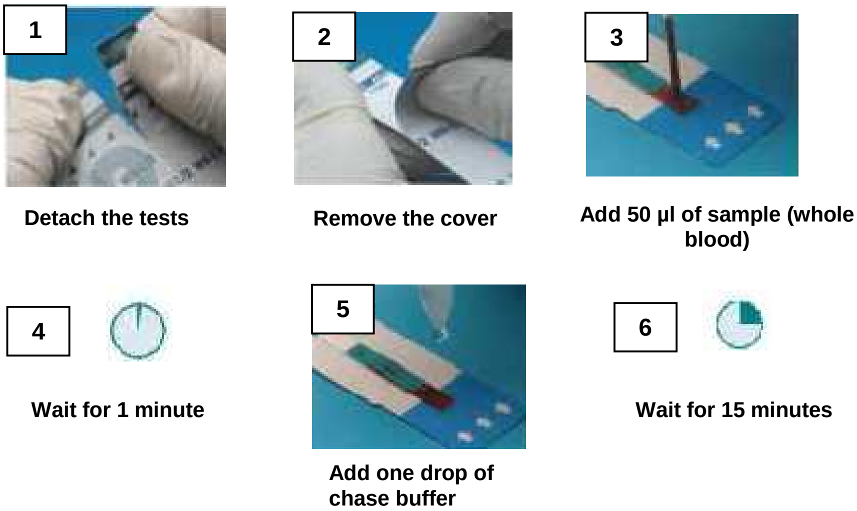

3.4 Interpretation of the test

DO NOT INTERPRETE the test after 60 minutes !

- **Non-reactive** – 1 red line
  One single red line in the control window (annotated « Control »).
- **Reactive** – 2 red lines

Two red lines : one in the control window (annotated « Control ») and one in the patient window (annotated « Patient »).
The intensity of the colour and the order of appearance of the lines is not important. Any red colour in the test zone is interpreted as a reactive test result.

- **Invalid** – no control line
  The absence of a red line in the control window (annotated « Control ») indicated that the test has deteriorated or that the procedure has not been correctly followed. REPEAT THE TEST !

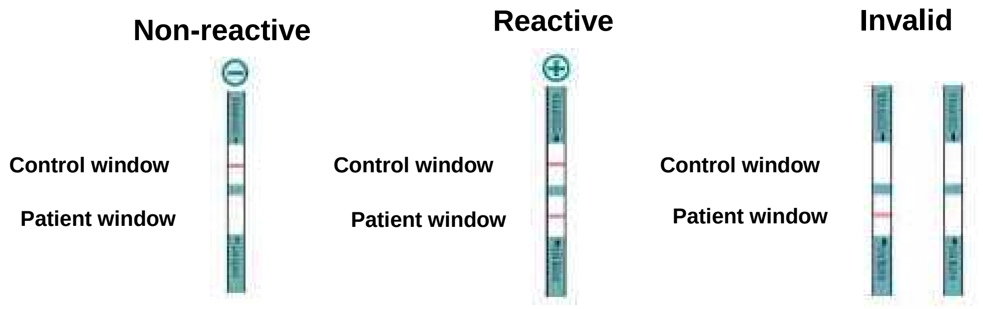

Result	Action to be taken
Non-reactive	Report as « Non-reactive ».
Reactive	Report as « Reactive ».<br>Confirm the reactive result with the recommended tests by your country's guidelines.
Invalid result	Repeat the test. If the control line of the second test is still not visible, report the result as « Invalid ».

3.5 Waste management, cleaning

- Discard the used tests in a « bio-hazard » container.

3.6 Result recording

- Most study sites (Nepal, Cambodia and Sudan) use a lab register to record results. The results are then transcribed at a later stage in the CRF. In DRC, the result may be recorded directly in the CRF.
- Record if the test was done or not, and provide a reason if it was not done.
- Record the result of the test: "Reactive"," Non reactive" or "Invalid".

4. Records and archives

Appendixes & forms to be completed	
**Number**	**Title**
1	Lab register
2	CRF

5. Document History

Revision	
SOP-WP2-LAB-32-V01-12Jul2012	Initial version
SOP-WP2-LAB-32-V01.1-03Dec2012	Translation in English<br>Addition of “3.5 Result recording”

Name and function	Date	Signature
Auteur		
Johan van Griensven	03/12/2012	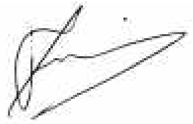
Revised by		
Barbara Barbé	14/01/2013	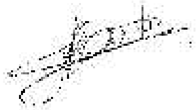
Approved by		
Emilie Alirol	22/01/2013	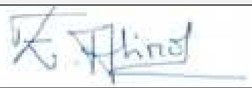

	**SOP Title:** Completing CRFs: WP2_CRF_Fever-v2.0_30JUL2013<br>WP2_CRF_Fever-v2.1_04OCT2013_SUDAN
	Project/study: Evaluation of Rapid Diagnostic Tests (RDT) in association with clinical and laboratory predictors for the diagnosis of Neglected Tropical Diseases (NTD) in patients presenting with persistent fever (≥1 week) in Cambodia, Nepal, Democratic Republic of the Congo and Sudan

1. Scope and application

The Case Report Form (CRF) contains all the data collected during the study. There is one CRF per patient. The aim of this SOP is to describe how the CRFs "WP2_CRF_Fever-v2.0_30JUL2013" and "WP2_CRF_Fever-v2.1_04OCT2013_SUDAN" should be completed and corrected. It applies to all personnel involved with on-site completion and verification of the CRFs in the NIDIAG Fever study in Cambodia, Nepal and Sudan.

2. Responsibilities

Function	Activities
Site investigator (or delegated staff e.g. nurses, laboratory technicians)	- Fill in the CRF according to the SOP<br>- Ensure the legibility, completeness, correctness of the data and consistency with the source documents (patient medical record, laboratory and clinical test results)<br>- Store the CRF in an appropriate place<br>- Correct errors reported by the study Monitor or Site Quality Manager
Site Quality Manager and External Monitor	- Verify that the data are accurate, complete and up-to-date<br>- Report mistakes and discrepancies to the investigator<br>- Ensure errors detected during monitoring are corrected by the investigator

3. Procedures

3.1 Subject privacy

- The CRF is an anonymous document. The patient's name, telephone or address must NOT appear on the CRF. The only identifiers present on the CRF are the patient number, the initials, the sex, the date of birth, the age and the place of residence.
- ONLY file anonymized copies of source documents (e.g. medical record, lab results) in the CRF, if required.
- DO NOT file the informed consent from (ICF) in the CRF.

3.2 Completing the CRF

3.2.1. General points

- Use a ballpoint pen (blue or black ink) to fill in the CRF.
- All entries should be in CAPITAL LETTERS.
- Write clearly. All entries should be legible to others.

- The CRF should be kept up-to-date while the patient is in the study. Enter data either immediately (during interview, in real time) or soon thereafter.
- Only enter results in the fields provided. Fill in the appropriate box or line for every field on each CRF page (unless indicated otherwise).
- Complete the header information on each page in a consistent way.
- Make sure the data collected in the CRF are consistent with the source documents.
- Ensure that you record numerical values in the units provided (*e.g.* kg, µl, ml, …)

3.2.2. Specific points for WP2_CRF_Fever-v2.0_30JUL2013

- Complete Patient Initials according to the following format: First name – Middle name – Last name. Put a dash when one name is missing.

  Note: In Sudan, a format of 4 letters will be used: First name – First middle name – Second middle name – Last name.
- Do not leave questions unanswered:
  - If an answer is not known, mark "NK" (Not Known).

    Note: If a date is unknown, enter "99" for the fields that are unknown (*e.g.* 99/99/2012)

    Note: If weeks/days are unknown, enter "0" in the respective data field
  - If a procedure is not done, enter "ND" (Not Done).

    Note: If a sample has not been collected, indicate all related tests with "ND" (Not Done) by adding a round bracket "}" in front of all the tests that apply.
  - If a question is not applicable, mark "NA" (Not Applicable).
- Some questions or sections have been shaded and crossed with a diagonal line. Do not consider these sections for data collection at your respective site.
- Always use the format DD/MM/YYYY (unless indicated otherwise).

  Note: For Nepal, the Nepali date (B.S.) can be recorded in the CRF.
- Make sure all visits are correctly dated!
- Urine dipstick - p.21:
  - Always tick "Leucocyte esterase" or "Leukocytes" depending on the parameter that is detected by the urine dipstick that is in use in your site.
  - If the urine dipstick gives a negative result for the parameter Erythrocytes/Hemoglobin, tick both boxes "Erythrocytes" and "Hemoglobin" in the left column and tick the box "neg" in the right column.
  - If the urine dipstick gives a positive result for the parameter Erythrocytes/Hemoglobin, tick the appropriate parameter in the left column and tick the correct result in the right column.
- Other body sites – p.27:

  If a culture is done on more than 1 body site/fluid, separate the different body sites/fluids in the right column with a slash "/".

Example:

**OTHER BODY SITES**<br>(throat, fluid, soft tissue, joint, liver, abscess, etc.)	If indicated, other body sites/fluids can be sampled.<br>Record the requested information below next to the tests performed.
Culture for *Mycobacterium*<br>(Sample N°: xxxxx-xx-BS__)<br>Result on p.32	Inoculation: ☒ Done ☐ Not done<br>Body site/fluid used: *abscess aspiration / ascitic fluid / pleural fluid*
Bacteriological culture<br>(Sample N°: xxxxx-xx-BS__)<br>Result on p.32	Inoculation: ☒ Done ☐ Not done<br>Body site/fluid used: *abscess aspiration / ascitic fluid / pleural fluid*

- Culture results – Culture on other body sites/fluids – p.32:
    - Separate the culture results according to body site/fluid used. Note the body site/fluid in the left column. Use 1 row per body site.
    - Record all the results from all the cultures that were performed on the same body site/fluid in the right column.
    - If more than 1 culture was performed on the same body site/fluid, there are 3 options:
        1. None of the cultures presented growth, no species were isolated.
           Tick "No growth" in the right column and note all the cultures that were performed and that presented no growth.
        2. Some of the cultures presented growth, the other cultures not. Species were isolated from some cultures but not from all cultures.
           Tick "Growth" in the right column and note the species that were isolated, and from which culture the species were isolated. Note also which cultures presented no growth.
        3. All the cultures presented growth, and one or more species were isolated.
           Tick "Growth" in the right column, and note the species that were isolated and from which cultures these species were isolated.
    - If more than 3 body sites/fluids were inoculated, make a copy of page 32 and insert it in the CRF. If for example two pages were filled in total, number the first page 32.1 of 32.2 and the second page 32.2 of 32.2.

Example:

CULTURE ON OTHER BODY SITES/FLUIDS<br>(refer to p.27)	
Body site/fluid used:<br>*Abscess aspiration*<br>(Sample N°: xxxxx-xx-BS__)	☒ No growth ☐ Growth<br>Species identification: ______________________________<br>*No growth (Mycobacterium and bacteriological culture)*
Body site/fluid used:<br>*Ascitic fluid*<br>(Sample N°: xxxxx-xx-BS__)	☐ No growth ☒ Growth<br>Species identification: *Mycobacterium tuberculosis (Mycobacterium culture).*<br>*No growth (Bacteriological culture)*
Body site/fluid used:<br>*Pleural fluid*<br>(Sample N°: xxxxx-xx-BS__)	☐ No growth ☒ Growth<br>Species identification: *Mycobacterium tuberculosis (Mycobacterium culture), Staphylococcus aureus (bacteriological culture)*

- Use the "Subsequent clinical assessment" form p.39-40 to record each scheduled and unscheduled visit.
  These visits can occur:
  - during hospitalization and during follow-up of outpatients after the baseline clinical assessment (visit 1)
  - after site discharge of a hospitalized patient and after the last outpatient visit
  - after the follow-up visit (1 month post-site discharge/last outpatient visit).
- Add extra copies of the forms "Subsequent clinical assessment" p.39-40 and "Medication Form" p.48 whenever needed. Each time you insert a "Subsequent clinical assessment" form or a "Medication Form", use the page number format explained below and put the clinical assessment pages in chronological order.
  e.g. when you conducted 3 subsequent clinical assessments in total, number the first page 39.1 of 39.3, the second page 39.2 of 39.3 and the third page 39.3 of 39.3. Apply the same format to page 40 and page 48.

  Note: It is possible that (unscheduled) subsequent clinical assessments occur after site discharge/last outpatient visit/1 month follow-up assessment of the patient. Place all forms in chronological order (some forms might be placed after site discharge/last outpatient visit).
- Fill out the form "Site discharge/last outpatient visit assessment" p.41-42 at:

  - the day of site discharge for the hospitalized patients
  - the day of the last outpatient visit for the patients who are not hospitalized

  It is up to the site investigator to decide during the assessment if the visit will be the last one, so that he/she can fill in the correct form (either "subsequent clinical assessment form" or "site discharge/last outpatient form").

  __Note__: If the patient unexpectedly returns for one or more outpatient visit(s) after the "site discharge/last outpatient form" has been completed, just fill out another "subsequent clinical assessment form(s)".

- Let the part on "Index testing" be completed by a designated person that is NOT the treating physician/site investigator (usually a laboratory technician) in order to keep these results blinded from the treating physician/site investigator.
- Fill the "Medication form" on page 47-48 as follows:
  - Record all ongoing treatments and all treatments started during or after inclusion of the patient in the study.
  - For treatments that started in the past, fill in the correct starting date. Record only the dosage that is currently given (even if the dosage was different in the past).
  - When a treatment changes during follow-up of the patient (*e.g.* change of dosage), fill the 'new' treatment in a new line on the "Medication form" (*e.g.* record the new dosage) Fill in the new start date and make sure that also a stop date has been assigned to the 'previous' treatment.
- __Sign and date__ the CRF on page 46 upon full completion of the CRF, after a __final review__ has been performed by the site investigator. By doing so, you take responsibility for the correctness and accuracy of the data recorded in the CRF.
- Sample collection and culture inoculation data (volume/date/time/initials etc.) are NOT collected in the CRF, but on separate site-specific forms (Annex 1: Sample Collection Form / Annex 2: Culture Inoculation Form). Fill out 1 of each form per patient and store them in the Laboratory File.

3.3 Correcting the CRF

- For any corrections that need to be made to data already recorded, ensure that the following are observed:
  - Draw through the error with a single line (i.e. ~~IZN~~)
  - Do not obscure the original entry. It should remain legible.
  - Do not overwrite an entry (for example changing 3 to 8)
  - Write the corrected entry next to the data being corrected (i.e. ~~IZN~~ RIF)
  - Initial and date each correction or amendment (i.e. ~~IZN~~ RIF HLJ 25/07/2012)
  - Do not use correction fluid
  - Do not scribble

- The instructions for completion and correcting of the CRF are added on page 2 of the CRF. Depending on the study progress or study, amendments to these instructions or other instructions might be added.

3.4 Storage and access

- Keep the CRFs in a locked and safe place, with restricted access to study staff.
- Store the part of the CRF on "Index testing" in the laboratory in a locked and safe place, with restricted access (not accessible by the treating physician nor the study investigator to ensure blinding of the index test results).
- DO NOT store the informed consent forms nor the patient identification lists together with the CRFs.
- Retain the CRFs according to local legislation and for at least 2 years after the end of the study.

4. Definitions and abbreviations

- CRF= Case Report Form: a printed document designed to record all the protocol required data.
- Investigator (or Site investigator): A person who is responsible for the conduct of a clinical trial at a trial site.
- Site quality manager: A person who is responsible for ensuring quality systems are applied at each step of the NIDIAG studies on a day-to-day basis. He/She oversees all research activities and makes sure that studies are conducted in accordance with the protocol, SOPs, GCP/GCLP and national regulations.
- Monitor: The monitor is a person who is responsible for verifying that the study is conducted, recorded and reported in accordance with the study protocol, the Good Clinical Practice (GCP), and the Standard Operating Procedures (SOPs).
- Source documents: These are original documents. They consist of data relevant to the current research such as medical records, administrative files, laboratory reports, consultation reports, pharmacy dispensation registers, etc...

5. Records and archives

**Appendices & Forms for completion**	
**Number**	**Title**
WP2_CRF_Fever-v2.0_30JUL2013	Case Report Form
Annex 1	Site specific "Sample Collection Form"
Annex 2	Site specific "Culture Inoculation Form"

6. Document History

**Revision**	
SOP-WP6-DATA-01-V1-01Feb2012	Initial version
SOP-WP6-DATA-01-V2-30Jul2013	Adaptation and specification of the general initial version to the specific filling out of WP2_CRF_Fever-v2.0_30JUL2013

SOP-WP6-DATA-01-V3-22Aug2013	- Addition of patient initials in Sudan<br>- Addition of how to fill in the Medication form
SOP-WP6-DATA-01-V4-09Oct2013	Addition of comments on:<br>- Urine dipstick<br>- Cultures of other body sites/fluids

**Name and function**	**Date**	**Signature**
Author		
Barbara Barbé	09/10/2013	
Reviewed by		
Harry van Loen	10/10/2013	
François Chappuis	10/10/2013	
Approved by		
Ninon Horié	11/10/2013	

SOP-WP6-DATA-01-V3-30Jul2013-Annex 1_CAMBODIA

SAMPLE COLLECTION FORM CAMBODIA

NIDIAG FEVER STUDY

**Patient N°**	☐☐☐☐☐ – ☐☐

Sample	Code	Sample taken	Volume taken	Date (DD/MM/YYYY)	Time (HH : MM)	Sampled by (Initials)	Comments
**Blood on EDTA**	**BE1**	☐ YES ☐ NO	ml	/ /	:		
**Blood (Dry tube)**	**BD1**	☐ YES ☐ NO	ml	/ /	:		
**Urine**	**UR1**	☐ YES ☐ NO	ml	/ /	:		
**Lymph node aspirate**	**LN1**	☐ YES ☐ NO	µl	/ /	:		
**Bone marrow aspirate**	**BM1**	☐ YES ☐ NO	µl	/ /	:		
**Sputum (1)**	**SM1**	☐ YES ☐ NO	ml	/ /	:		
**Sputum (2)**	**SM2**	☐ YES ☐ NO	ml	/ /	:		
**Sputum (3)**	**SM3**	☐ YES ☐ NO	ml	/ /	:		
**Cerebrospinal fluid**	**CS1**	☐ YES ☐ NO	ml	/ /	:		
**Liver abscess aspirate**	**LA1**	☐ YES ☐ NO	ml	/ /	:		
**Other body site/fluid :** ____________________	**BS__**	☐ YES ☐ NO		/ /	:		
**Other body site/fluid :** ____________________	**BS__**	☐ YES ☐ NO		/ /	:		

Please file this form in the Laboratory File.

SOP-WP6-DATA-01-V3-30Jul2013-Annex 1_NEPAL

SAMPLE COLLECTION FORM NEPAL
NIDIAG FEVER STUDY

**Patient N°**	☐☐☐☐☐ – ☐☐

**Sample**	**Code**	**Sample taken**	**Volume taken**	**Date (DD/MM/YYYY)**	**Time (HH : MM)**	**Sampled by (Initials)**	**Comments**
**Blood on EDTA**	**BE1**	☐ YES ☐ NO	ml	/ /	:		
**Blood (Dry tube)**	**BD1**	☐ YES ☐ NO	ml	/ /	:		
**Urine**	**UR1**	☐ YES ☐ NO	ml	/ /	:		
**Lymph node aspirate**	**LN1**	☐ YES ☐ NO	µl	/ /	:		
**Bone marrow aspirate**	**BM1**	☐ YES ☐ NO	µl	/ /	:		
**Sputum (1)**	**SM1**	☐ YES ☐ NO	ml	/ /	:		
**Sputum (2)**	**SM2**	☐ YES ☐ NO	ml	/ /	:		
**Sputum (3)**	**SM3**	☐ YES ☐ NO	ml	/ /	:		
**Cerebrospinal fluid**	**CS1**	☐ YES ☐ NO	ml	/ /	:		
**Liver abscess aspirate**	**LA1**	☐ YES ☐ NO	ml	/ /	:		
**Other body site/fluid :** ____________________	**BS__**	☐ YES ☐ NO		/ /	:		
**Other body site/fluid :** ____________________	**BS__**	☐ YES ☐ NO		/ /	:		

Please file this form in the Laboratory File.

SAMPLE COLLECTION FORM SUDAN
NIDIAG FEVER STUDY

**Patient N°**	☐☐☐☐☐ – ☐☐

Sample	Code	Sample taken	Volume taken	Date (DD/MM/YYYY)	Time (HH : MM)	Sampled by (Initials)	Comments
**Blood on EDTA**	**BE1**	☐ YES ☐ NO	ml	/ /	:		
**Blood (Dry tube)**	**BD1**	☐ YES ☐ NO	ml	/ /	:		
**Urine**	**UR1**	☐ YES ☐ NO	ml	/ /	:		
**Lymph node aspirate**	**LN1**	☐ YES ☐ NO	µl	/ /	:		
**Bone marrow aspirate**	**BM1**	☐ YES ☐ NO	µl	/ /	:		
**Sputum (1)**	**SM1**	☐ YES ☐ NO	ml	/ /	:		
**Sputum (2)**	**SM2**	☐ YES ☐ NO	ml	/ /	:		
**Sputum (3)**	**SM3**	☐ YES ☐ NO	ml	/ /	:		
**Cerebrospinal fluid**	**CS1**	☐ YES ☐ NO	ml	/ /	:		
**Liver abscess aspirate**	**LA1**	☐ YES ☐ NO	ml	/ /	:		
**Other body site/fluid :** ____________________	**BS__**	☐ YES ☐ NO		/ /	:		
**Other body site/fluid :** ____________________	**BS__**	☐ YES ☐ NO		/ /	:		

Please file this form in the Laboratory File.

CULTURE INOCULATION FORM CAMBODIA
NIDIAG FEVER STUDY

**Patient N°**	☐☐☐☐☐ – ☐☐

Sample	Code	Sample taken	Volume taken	Date (DD/MM/YYYY)	Time (HH : MM)	Sampled by (Initials)	Comments
**Blood culture 1**	**HE1**	☐ YES ☐ NO	ml	/ /	:		
**Blood culture 2**	**HE2**	☐ YES ☐ NO	ml	/ /	:		
**Urine culture**	**UC1**	☐ YES ☐ NO	µl	/ /	:		
**Lymph node aspirate culture for *Mycobacterium tuberculosis***	**NC2**	☐ YES ☐ NO	µl	/ /	:		
**Bone marrow aspirate culture for *Salmonella/Brucella***	**BC2**	☐ YES ☐ NO	µl	/ /	:		
**Sputum culture for *Mycobacterium tuberculosis***	**MC1**	☐ YES ☐ NO	ml	/ /	:		
**Bacteriological culture on CSF**	**CC1**	☐ YES ☐ NO	ml	/ /	:		
**Culture on other body site/fluid : ________________________**	**BS__**	☐ YES ☐ NO		/ /	:		
**Culture on other body site/fluid : ________________________**	**BS__**	☐ YES ☐ NO		/ /	:		

Please file this form in the Laboratory File.

SOP-WP6-DATA-01-V3-30Jul2013-Annex 2_NEPAL

CULTURE INOCULATION FORM NEPAL
NIDIAG FEVER STUDY

Patient N°	☐☐☐☐☐ – ☐☐

Sample	Code	Sample taken	Volume taken	Date (DD/MM/YYYY)	Time (HH : MM)	Sampled by (Initials)	Comments
**Blood culture 1**	**HE1**	☐ YES ☐ NO	ml	/ /	:		
**Blood culture 2**	**HE2**	☐ YES ☐ NO	ml	/ /	:		
**Urine culture**	**UC1**	☐ YES ☐ NO	µl	/ /	:		
**Lymph node aspirate culture for *Mycobacterium tuberculosis***	**NC2**	☐ YES ☐ NO	µl	/ /	:		
**Bone marrow aspirate culture for *Leishmania***	**BC1**	☐ YES ☐ NO	µl	/ /	:		
**Bone marrow aspirate culture for *Salmonella/Brucella***	**BC2**	☐ YES ☐ NO	µl	/ /	:		
**Sputum culture for *Mycobacterium tuberculosis***	**MC1**	☐ YES ☐ NO	ml	/ /	:		
**Bacteriological culture on CSF**	**CC1**	☐ YES ☐ NO	ml	/ /	:		
**Culture on other body site/fluid :** ______________________	**BS__**	☐ YES ☐ NO		/ /	:		
**Culture on other body site/fluid :** ______________________	**BS__**	☐ YES ☐ NO		/ /	:		

Please file this form in the Laboratory File.

CULTURE INOCULATION FORM SUDAN
NIDIAG FEVER STUDY

**Patient N°**	☐☐☐☐☐ – ☐☐

Sample	Code	Sample taken	Volume taken	Date (DD/MM/YYYY)	Time (HH : MM)	Sampled by (Initials)	Comments
**Blood culture 1**	**HE1**	☐ YES ☐ NO	ml	/ /	:		
**Blood culture 2**	**HE2**	☐ YES ☐ NO	ml	/ /	:		
**Lymph node aspirate culture for *Leishmania***	**NC1**	☐ YES ☐ NO	µl	/ /	:		
**Bone marrow aspirate culture for *Leishmania***	**BC1**	☐ YES ☐ NO	µl	/ /	:		
**Bone marrow aspirate culture for *Salmonella/Brucella***	**BC2**	☐ YES ☐ NO	µl	/ /	:		
**Sputum culture for *Mycobacterium tuberculosis***	**MC1**	☐ YES ☐ NO	ml	/ /	:		
**Bacteriological culture on CSF**	**CC1**	☐ YES ☐ NO	ml	/ /	:		
**Culture on other body site/fluid : ________________________**	**BS__**	☐ YES ☐ NO		/ /	:		
**Culture on other body site/fluid : ________________________**	**BS__**	☐ YES ☐ NO		/ /	:		

Please file this form in the Laboratory File.

SOP-WP6-DATA-02-V2-04Mar2014

	**SOP Title:** Procedure for Data Management (data entry):<br>- CRF Flow and Tracking<br>- Data Entry<br>- Data Cleaning<br>- Querying & DCF management<br>- Database Lock<br>- Backup of study data and security measures
	Project/study: NIDIAG FEVER & NIDIAG DIGESTIVE studies

1. Scope and application

The purpose of this SOP is to describe the different aspects related to data management.
The SOP applies to the NIDIAG Fever and NIDIAG Digestive studies and in particular to the study personnel involved in:

- CRF Flow and Tracking
- Data Entry
- Data Cleaning and Data Management

2. Responsibilities

Function	Activities
Clinical and lab staff involved in CRF completion (PI, nurses, lab technicians…)	- Fill in the CRF according to the SOP WP6 DATA 01 and the General Instructions in the CRF<br>- Ensure the legibility, completeness, correctness of the data and consistency with the source documents (patient medical record, laboratory and clinical test results)<br>- Store the CRF in appropriate place<br>- Correct errors reported by the study Monitor or Site Quality Manager
Site Quality Manager and External Monitor	- Verify that the data are accurate, complete and up-to-date<br>- Report mistakes and discrepancies to the investigator<br>- Ensure errors detected during monitoring are corrected by the investigator
Local Data Management	- Ensure that all CRFs are complete before starting Data Entry<br>- Perform Data Entry according to the Data Entry guidelines<br>- Manage a system of queries with the objective to complete and correct the study data.<br>- Ensure a regular backup of study data
Coordinating Data Management (at ITM))	- Coordination and focal point for data management for the three NIDIAG studies: NEURO, FEVER and DIGESTIVE study

3. Procedures

3.1 CRF Flow and Tracking

- **Clinical and Lab Staff**
    - Make sure that data are accurate and that the CRFs are complete and signed (if applicable) before transferring them to the Data Management staff.
    - Keep a copy of the transferred CRFs at site.
    - Arrange the CRFs per patient.

- **Data Management Staff**
    - Keep track of all
        - CRFs which are received,
        - CRFs which are missing,
        - CRFs which are entered,
        - CRFs not yet entered (see also 3.2)
    - Arrange CRFs per patient

3.2 Data Entry

- For each patient, ensure that the CRF is complete and signed (if applicable) before starting data entry.
- Follow the data entry guidelines (see appendix 1).
- For each CRF, make sure you always complete the first entry before starting with the second entry.
- After the first entry, write your initials and date of entry on the first page of the CRF. The same applies for second data entry: write your initials and date of entry on the first page of the CRF. As such you can better keep track that data entry has been done, by whom and when.
- Complete also the file 'CRF Tracking'. This file keeps track on the status of the CRF (if received or missing) and on the status of data entry (first entry, second entry completed) (see appendix 2).
- Ensure for each CRF that all data have been entered and that all queries have been resolved.
- 'Finalize' the second entry not before the moment of database lock (see also 3.5).

3.3 Data Cleaning

- Follow the data entry guidelines (see appendix 1).
- Click 'Check and save' in the database after entering the data of each page or TAB. The system will then prompt a message to save the page. In case that data are still missing or out of range or inconsistent, an 'error' message will be fired to indicate that these issues have to be checked or resolved.
- Use 'Show Compare' in the database to resolve the differences between first entry and second entry.
- Make sure that the corrections in the database are done before selecting 'finalize'.
  After « finalizing» the second entry, data cannot be corrected anymore!

3.4 Querying and DCF management

- Use the DCFs for querying the clinical or laboratory staff (→ DCF= Data Clarification Forms; see appendices 3 and 4).
- Assign a new DCF number for each new DCF.

- Complete also the file ‘DCF Tracking’. This file lists information on the flow and status of the queries of each DCF (Raised; responded, resolved; re-raised; see also appendix 5). If applicable, then correct the database based on the response.
- Keep the DCFs in a dedicated place at Data Management: if digital DCFs, keep them in the folder ‘DCF’; if paper DCFs, keep them in a binder ‘DCF’. Sort them into the following sections: 1. ‘DCFs raised’. ; 2. ‘DCFs responded’; 3. ‘DCFs resolved’. (put the re-raised in the folder ‘raised’)

3.5 Database Lock

- When the database is complete and all queries are resolved, you should still select ‘finalize’ for each second entry. This moment of ‘finalizing’ the database is known as ‘Database Lock’. After the Database Lock, data cannot be changed anymore and the database is then ready to be transmitted to the investigator or statistician.
- Database Lock should be approved by some key study members. At least the coordinating Data Management and the coordinating Study Investigator are involved in the approval.

3.6 Backup of study data and security measures

- Make a daily backup of the study data.
- Keep the backup in a folder ‘Backup’ at a server or study computer(s) and also at an external memory device (e.g. USB stick, DVD, …).
- Complete the form ‘Data Backup Log’ after each backup (see appendix 6).
- Keep the study computers and external memory device(s) at a safe place. Only authorized study personnel might have access to the study computers and external memory device(s).
- Use the study computers and external memory device(s) only for professional reasons, in particular for the NIDIAG project.
- Provide the backup to the Coordinating Data Management at ITM each Friday, unless decided otherwise.

4. Definitions

- CRF= Case Report Form: a printed document designed to record all the protocol required data.
- Investigator (or Site investigator): A person who is responsible for the conduct of a clinical trial at a trial site.
- Site quality manager: A person who is responsible for ensuring quality systems are applied at each step of the NIDIAG studies on a day-to-day basis. He / She oversees all research activities and makes sure that studies are conducted in accordance with the protocol, SOPs, GCP/GCLP and national regulations.
- Monitor: The monitor is a person who is responsible for verifying that the study is conducted, recorded and reported in accordance with the study protocol, the Good Clinical Practice (GCP), and the Standard Operating Procedures (SOPs).
- Source documents: These are original documents. They consist of data relevant to the current research such as medical records, administrative files, laboratory reports, consultation reports, pharmacy dispensation registers, etc...

5. Records and archives

**Appendices & Forms for completion**	
**Number**	**Title**
1	Data entry guidelines
2	CRF Tracking File
3	Data Clarification Form - multiple queries at each DCF form
4	Data Clarification Form - one query at each DCF form

5	DCF Tracking File
6	Data Backup Log

6. Document History

**Revision**	
SOP WP6-DATA-02, Version 1.0, 02 DEC 2013	NIDIAG study
SOP WP6-DATA-02, Version 2.0, 04 MAR 2014	FEVER and DIGESTIVE studies

**Name and function**	**Date**	**Signature**
Author		
Harry van Loen	04/03/2014	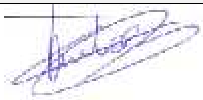
Reviewed by		
Jan Jacobs	06/03/2014	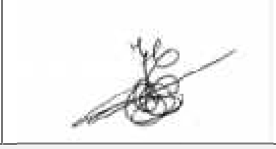
Approved by		
Ninon Horié	06/03/2014	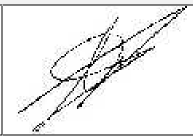

Read these instructions before each working day during the first weeks!
Keep a copy of these instructions near you while you enter data!

DATA ENTRY GUIDELINES
NIDIAG FEVER STUDY DATABASE

Author: H. van Loen

Contents

Contents………. ……….......1
1. Logging In – Opening the database………..………………………………………………………..………………….............2
2. Searching for a 'CRF' (existing patient file)……….………………………… ……………………………………..3
3. Creating a new 'CRF' (new patient file)……………………………… …………………………………….......3
4. General Instructions………………………………………………………… ………………………………….......5
5. Specific Instructions ………………………………………………………………………………………………………6
   5.1 For all Forms/TABs………………………………………………………………………………………………..6
      5.1.1 Check and Save………………………………………………………………………………….....6
      5.1.2 Dates……………………………………………………………………………………………………7
   5.2 Page 1-3………7
   5.3 Page 4 Inclusion/Exclusion…………………………… ……………………………………………………7
   5.4 Page 10-11………7
   5.5 Page 39-40………8
   5.6 Page 41-46………8
   5.7 Page 47-48………9
6. First and Second Entry ……………………………………………………………………………………………9
7. Selecting 'Finalize'……………………………………………… …………………………………………………10
8. Closing the database…………………………………………………………………………………………..11
9. Backup of the data……………………………………………………………………………………………….11

1. Logging In – Opening the database

- Open the NIDIAG application file in: C:/NIDIAG/STUDY FEVER/Database   as shown below:

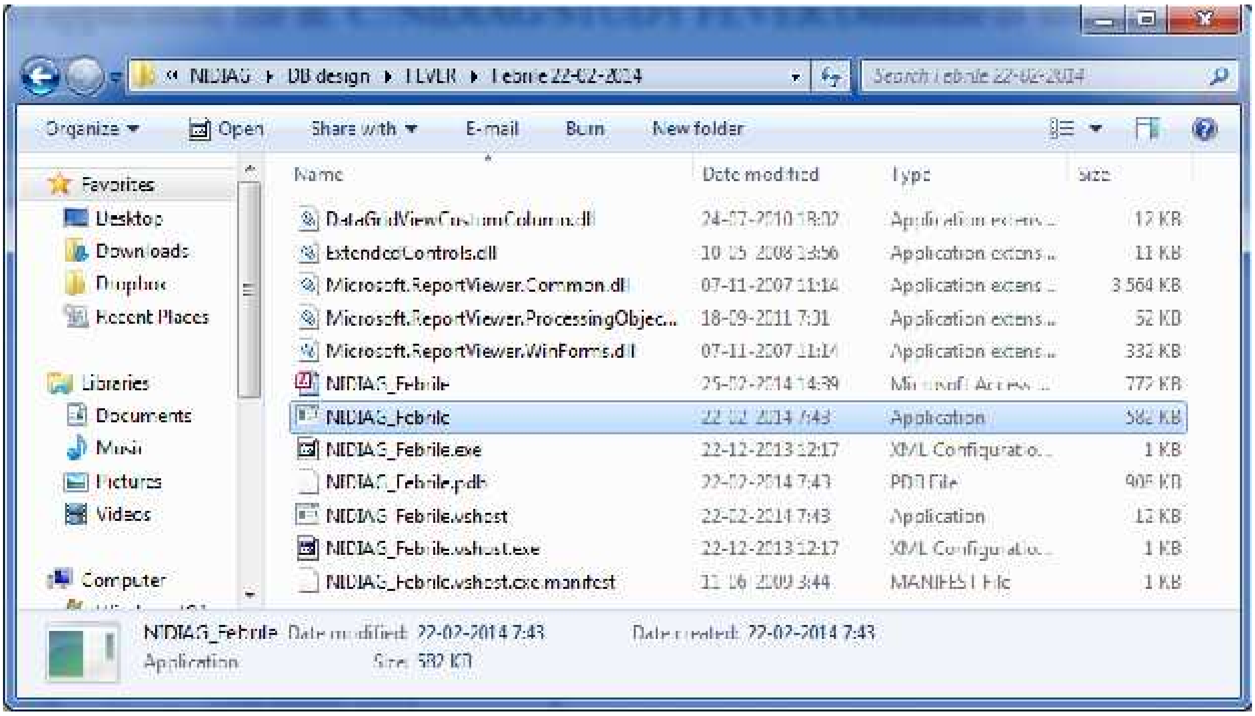

The Login will be shown to enter your User ID and Password which were assigned to you
If you have forgotten your password, then please take contact with the coordinating data manager at ITM with an email at hvanloen@itg.be.

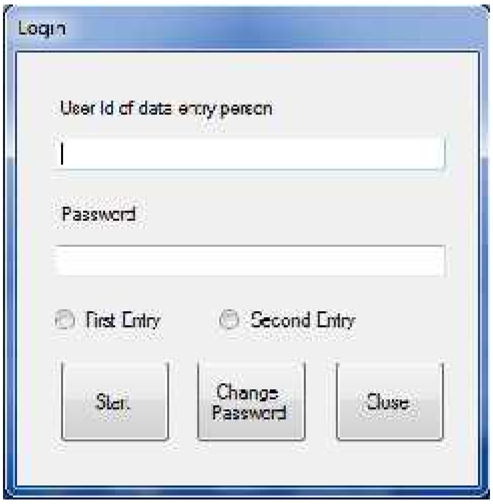

Note : If you login for the first time, then fill in your User ID and Password which was assigned to you, select first entry and then select « Change Password » to change it into a personal password. Write this password in your personal files!

- Select ‘First Entry’ (or ‘Second Entry’ if applicable)
- Select ‘Start’

The Database will open at the first TAB ‘Page 1-3':

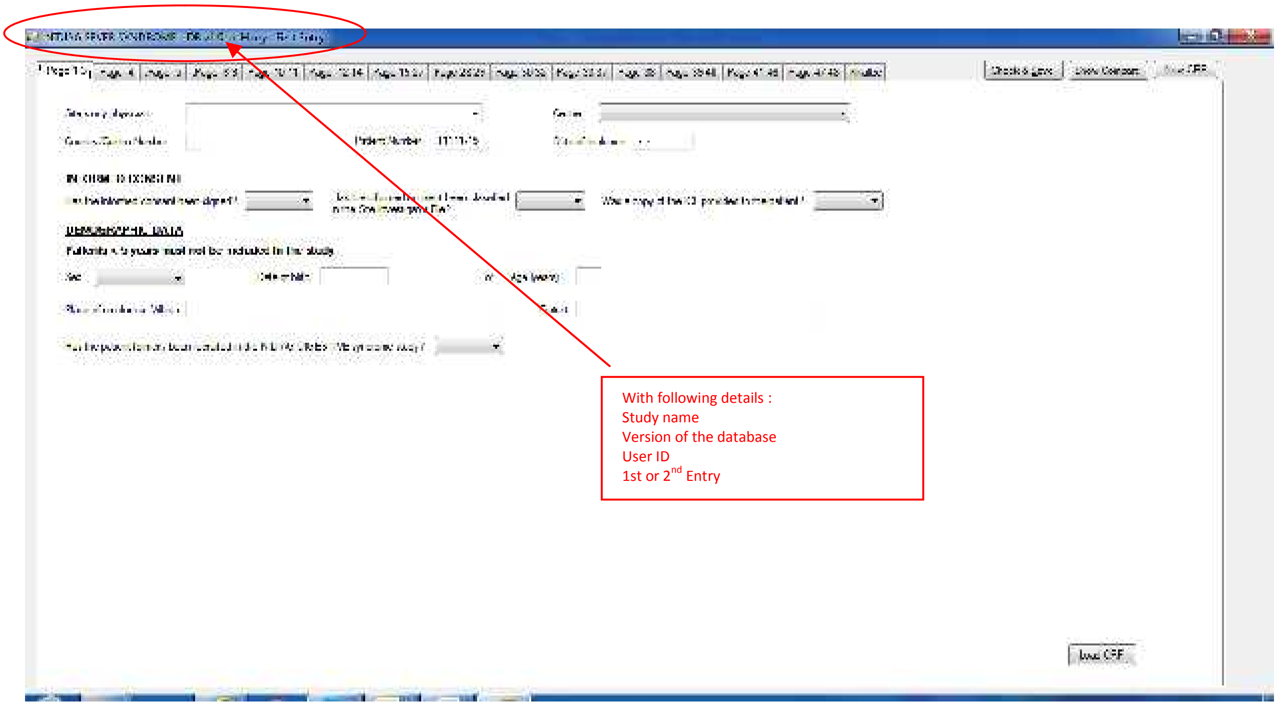

2. Searching for a ‘CRF’ (existing patient file)

- When you have opened the database, then navigate to the first TAB (page 1-3), in particular the question ‘Patient Number’.

- Write the number which you are searching for and then select ‘Load CRF’→ The following message appears: **‘Loading successful for the patient ID ….’**. After confirming ‘ok’ all patient data for the chosen patient ID are shown.

3. Creating a new ‘CRF’ (new patient file)

- When a patient file is not found (see above section 2), then this means that no data for this patient have been introduced yet in the database.

Following message appears: **‘No such record found’** (see next page).

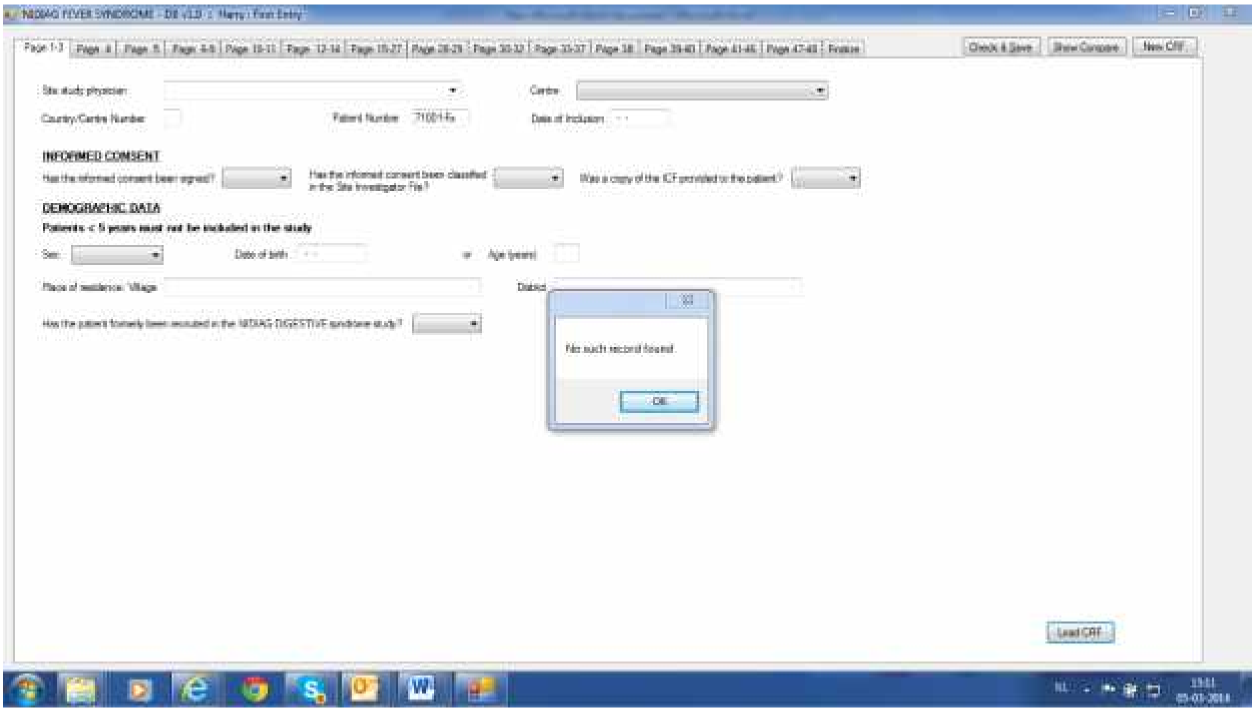

- It is then good practice to check always first the ‘Patient number’ you have entered against the Patient number on the paper CRF. Correct if necessary.

- When it is confirmed that the patient number is not found in the database, a new CRF has to be created.
- Navigate to the last TAB (New CRF), and select ‘New CRF’.

- The system will show the CRF pages for the ‘new’ patient.
- Note that all forms are still ‘blank’ = empty.

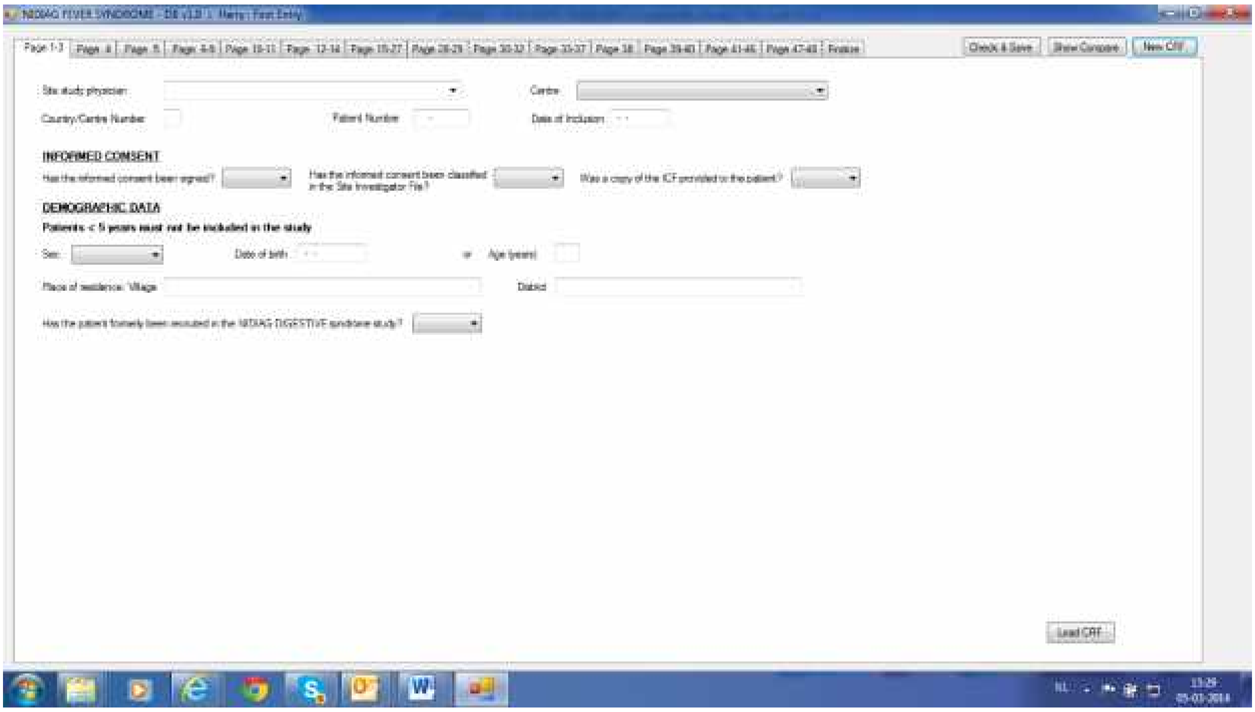

- It is important to complete the first TAB (Page 1-3) in the database before entering the other patient's data!!

4. General Instructions

- The paper documents you use with all the written or selected data (for example by the clinical or laboratory staff) are called **'Case Report Form' (CRF)**.

- The 'TABs' in the database, from left to right, reflect the paper CRF (in fact, the database mirrors the paper CRF).

- Click on each TAB to open the different pages.

- Enter the data from top to bottom or left to right.

- Check what is needed in the database and look for this information in the CRF (and not reverse).

- Complete all questions from the same TAB before continuing with next TAB.

- For unknown data, enter :
  - NK (format text; Not Known)
  - 99-99-9999 (format dates)
  - 999 (format number)
  - NA (for drop down lists where NK is not shown)

!Important:

- **For Date format: enter as dd-mm-yyyyy** (and not as dd/mm/yyyy ; if necessary change the computers settings into 'short date', see also 'control panel' >> 'region and language settings' >> additional settings).

- **Complete all text in CAPITAL LETTERS.**

- **For number format: use a 'zero' before if applicable:** e.g. '01' instead of '1' when there are 2 digits available ; 065 instead of 65 when there are 3 digits…

After the first entry, write your initials and date of entry on the first page of the CRF. The same applies for second data entry: write your initials and date of entry on the first page of the CRF. As such, you can better keep track that data entry has been done, by whom and when.

5. Specific Instructions

5.1 For all Forms/TABs

5.1.1. Check and save

- After completing each TAB, select ‘Check and save’.
- In case all is well, then the following message appears **‘Do you want to save this page?’**
- Continue with ‘Yes’ (or select ‘No’ if you want to correct data)

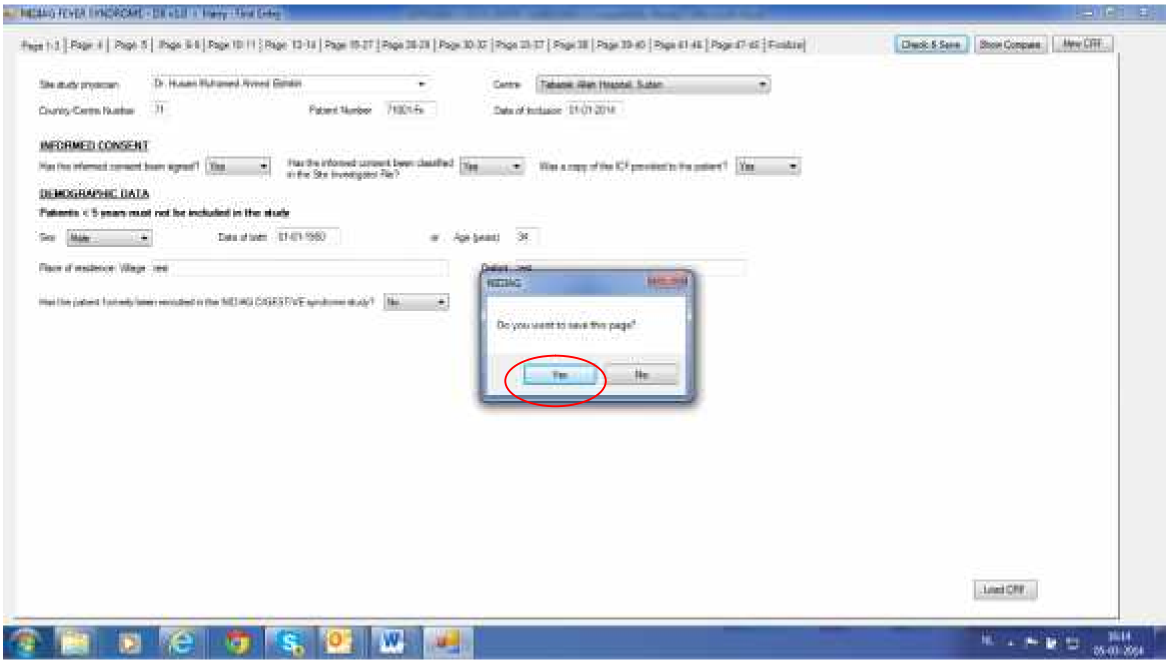

- Be careful: red warning or error messages indicate that some data are missing or inconsistent!

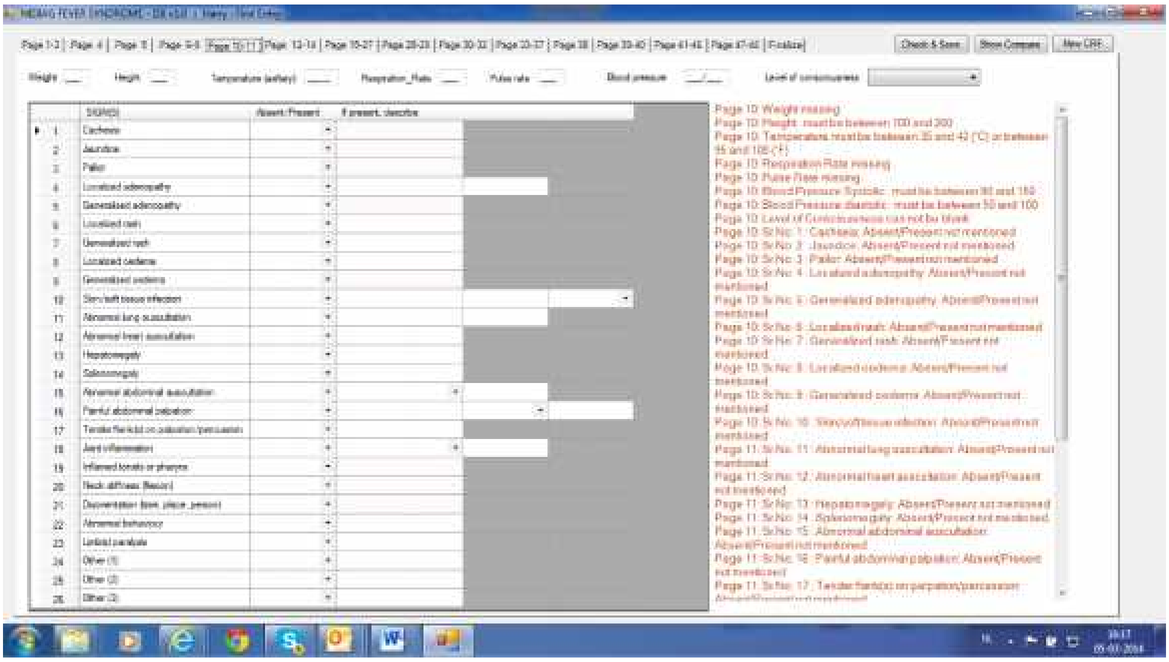

5.1.2. Dates (in general)

- For messages as follows:

  …Date must be before today…
  …Future dates are not allowed…
  …Date must be between 17-Jan-2013 and 31-Dec-2015

  Check out well the particular date!

5.2 Page 1- 3

- Complete these pages always as first from the moment a 'new' patient CRF is created!!
- Ensure that the patient is not yet in the CRF (avoid duplicates of patient data!) Check the number of the patient between the database and the CRF.
- If a warning appears similar to the following:
  Page 3: Date of inclusion must be between 17-Jan-2013 and 31-Dec-2015
  Then check out the date for the question 'Date of inclusion'!

5.3 Page 4: Inclusion/Exclusion

- If a warning appears similar to the following:
  Page 4: Al least one inclusion criteria must be met
  Page 4: At least one exclusion criteria met. Patient can not be enrolled in the study.
  Then check out the answers from inclusion criteria/exclusion criteria.

- If a warning appears similar to the following:
  Page 4: Inclusion criteria not met. Patient cannot be enrolled in the study. Please check!
  Then check out the answer(s) for 'Selection for this study'.

- If a warning appears similar to the following:
  Mismatch in Patient_Number from Page-1.
  Then check out well the Patient number recorded at page 4 against the Patient number at page 1-3.

5.4 Page 10- 11

- Questions Weight, Height, Temperature, Respiration rate, Pulse rate, Blood pressure: **Put a zero in front of the number (if needed): e.g.**

  Weight: 65 → 065
  Height: 99 → 099
  Respiration rate: 60 → 060
  Pulse rate: 75 → 075
  Blood pressure: 90/50 → 090/050

  Temperature: **Put a zero in front of the number (if needed) AND put a zero after the decimal point** ex. 38. → 038.0 (°C) ; 107. → 107.0 (°F)

5.5 Page 39- 40

- The various subsequent clinical assessments (visits) will be recorded here.
- Make sure that in the CRF all subsequent clinical assessments are sorted in chronological order before entering this TAB!

- The table 'subsequent clinical assessments' lists all data from CRF page 39.
- Enter row by row all clinical assessments data from various visits (if applicable). The first row will have the oldest visit date, the last row will have the most recent visit date. Please note that the table also lists the question 'Diagnosis unknown' and 'Hospitalisation' (CRF page 40).

- The table 'Working Diagnosis' lists all data related to Working diagnosis (CRF page 40).
- Make sure to group all data per visit! The visit date defines the visit.

- The table 'Clinical signs/symptoms lists all data related to Clinical symptoms/signs (CRF page 39).
- Make sure to group all data per visit! The visit date defines the visit.

5.6 Page 41- 46

- The 'Site Discharge/Last Outpatient Visit Assessment' visit and the 'Follow-Up Assessment' visit will be recorded here.

- The table 'Site Discharge/Last Outpatient Visit Assessment/ Follow-Up Assessment' lists all data from CRF page 41 till 45.

- Enter on the first row 'Discharge' data referring to Site Discharge/Last Outpatient Visit Assessment.
- Enter on the second row 'Post discharge' data referring to Follow-Up Assessment.
- Please note that the table also lists the question 'Diagnosis unknown', 'Hospitalisation' and 'Patient referred to other hospital' (CRF pages 42 and 45).

- The table 'Clinical signs/symptoms lists all data related to Clinical symptoms/signs (CRF page 41 and 43).
- Make sure to group all data per visit! The visit date defines the visit.

- The table 'Working Diagnosis' lists all data related to Working diagnosis (CRF page 42 and 45, which is the FINAL diagnosis).
- Make sure to group all data per visit! The visit date defines the visit.

- The table 'Other investigations' lists all data from 'Other investigations' (CRF page 44).

5.7 Page 47- 48

- If a warning appears similar to the following:
  Stop DATE can not be before Start DATE
  Then check out well Start and Stop Date

6. First Entry/ Second Entry

- For First Entry → select ‘First Entry’ and then ‘Start’.

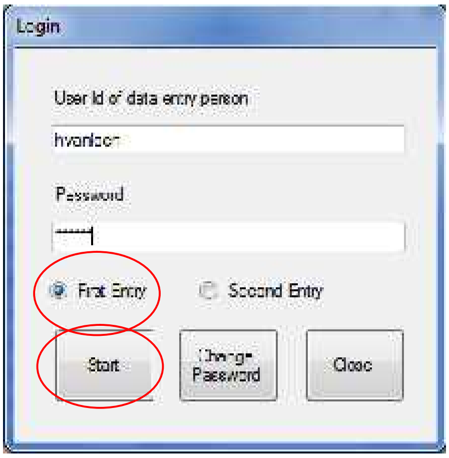

Note : Before starting with second entry you should first ‘finalizing’ the first entry

- For Second Entry → select ‘Second Entry’ and then ‘Start’.

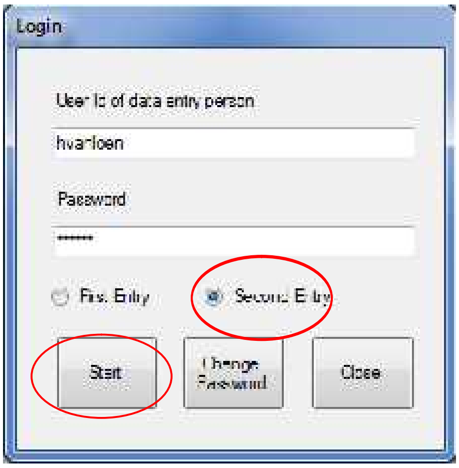

The second data entry clerk has the responsibility to correct the data if needed. He/she must always verify well the data of entry 1 and entry 2 against the data of the CRF!

- The differences between first and second entry are indicated during the second entry by clicking **‘Check and save’**. Another way to show the differences is by clicking **‘Show compare’** during the second entry and at each TAB**.** As such, the results which are different are listed.

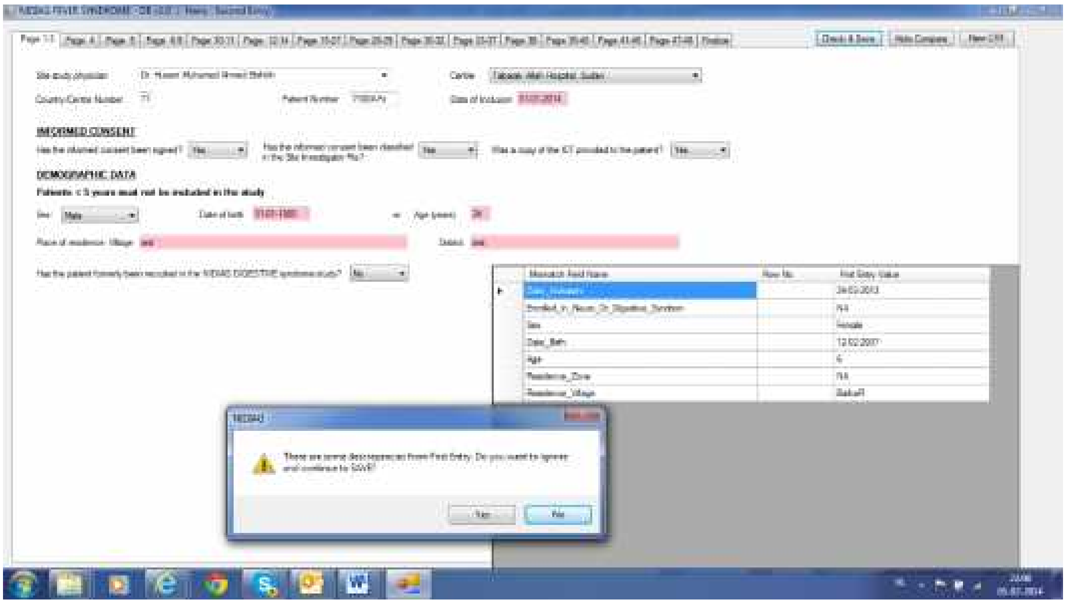

- It is the second data entry clerk (with support of the data manager if needed) who decides if first entry or second entry or another result should be recorded.

7. Selecting ‘Finalize’

- After completing all data for first entry select ‘Finalize’. As long as first entry is not ‘finalized’ you cannot start with second entry.
- The second entry gives still the opportunity to correct data and to complete missing data.

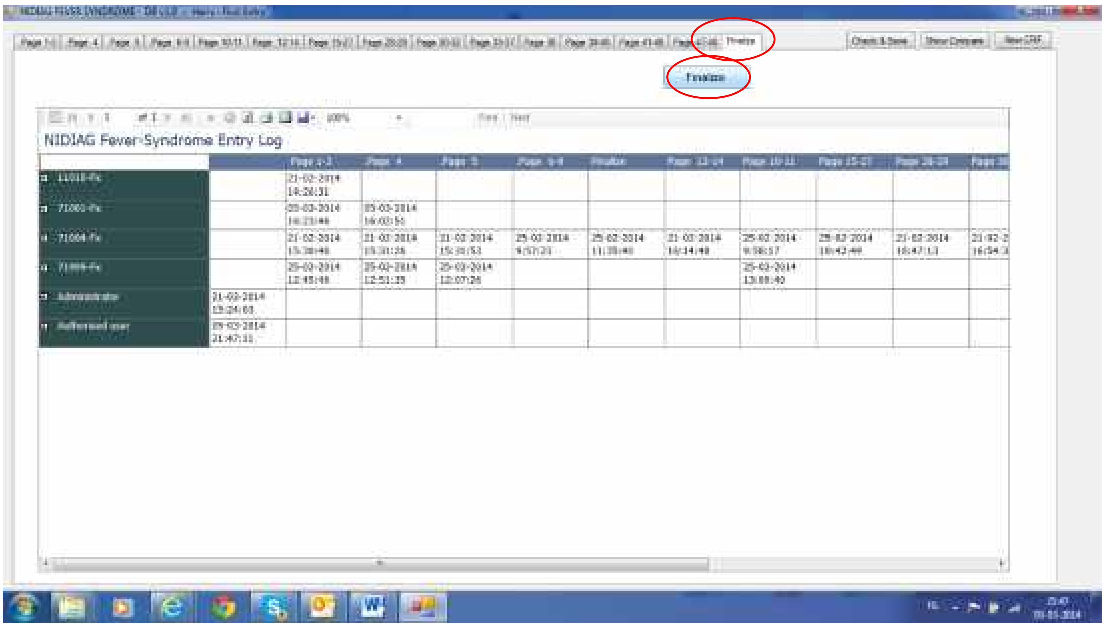

- Select ‘Finalize’ also after completing all data for second entry. After finalizing second entry, it is not possible anymore to change the data for first or second entry!

It is for this reason that the moment of selecting ‘Finalize’ is intended as the final phase, just before ‘database lock’.

8. Closing the database

- Close the database by selecting the red right upper corner.

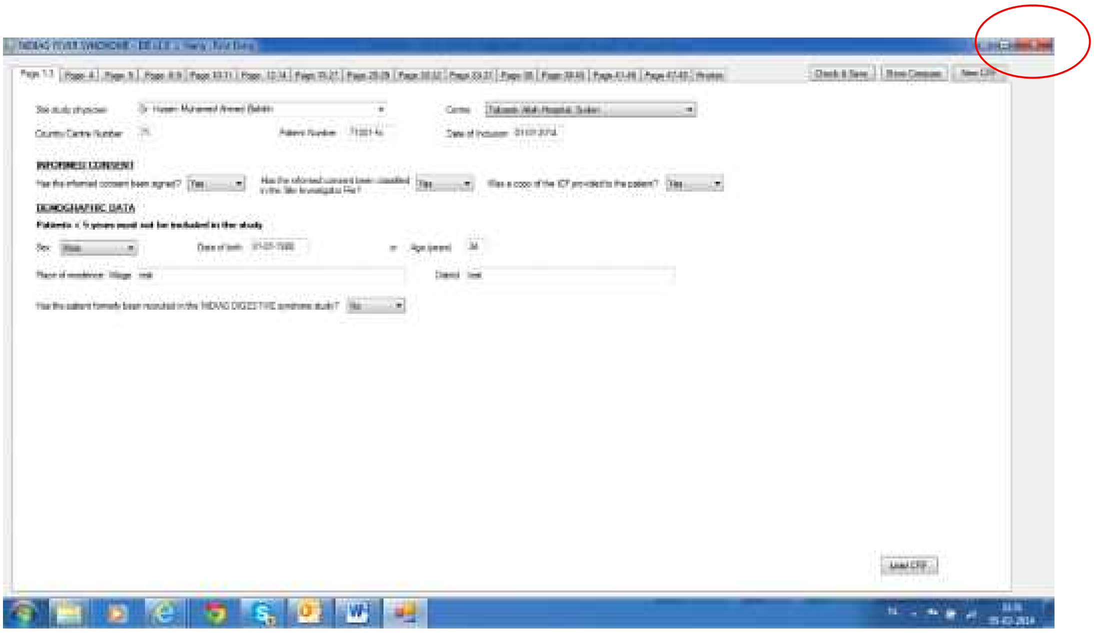

9. Backup of the data

- **Make a daily backup of the study data.**
- Use following format: NIDIAG_FEVER_DB_v1.0_date.
- Keep the backups, from different dates, at a server or study computers (in the folder 'Backup') and also at an external memory device (e.g. USB stick, DVD, …).
- Complete this form after each backup. Keep the study computers and external memory device(s) at a safe place. Only authorized study personnel might have access to the study computers and external memory device(s).
- Use the study computers and external memory device(s) only for professional reasons, in particular for the NIDIAG project.
- Provide the backup to the Coordinating Data Management at ITM each Friday, unless decided otherwise.

SOP-WP6-DATA-02-V2-04Mar2014-Annex2

Participant N°<br><br>_________ - _____	CRF version:<br><br>1.0, 2.0, …	CRF Completed ?<br><br>Yes/No	Date receival CRF<br><br>_ _/ _ _/ _ _ _ _	CRF page/data missing	Initials	Date 1st entry done<br>_ _/ _ _/ _ _ _ _	Initials	Date 2nd entry done<br>_ _/ _ _/ _ _ _ _	Initials	Queries not resolved yet<br><br>Yes/No	Remarks

SOP-WP6-DATA-02-V2-04Mar2014-Annex3

DATA CLARIFICATION FORM

DCF N° ____

From (name) ___________

To (name) ____________

Patient n° ______ - ___ CRF version : _._

Name CRF section _____________________________

Visit ___ Visit date ___/___/_____

Data to clarify

**Page number**	**Query**	**Response (by clinical or Lab staff)**	**Resolved (Yes/ No)**	**Re-raised (Yes/No)**	**DCF N°**

________________________________ ________________________________

Query raised by (initials and date) Responded by (initials and date)

____

Database updated (initials and date)

SOP-WP6-DATA-02-V2-04Mar2014-Annex4

DATA CLARIFICATION FORM

DCF N° __ __ __ __

**Participant N° :**	**Visit + Visit date :**	**CRF section:**	**Data to clarify:**	**CRF page:**	**CRF version:**

**Query :**

Raised by: __ __ __ __ __ __ __ __ __ __ __ __ __ __ (Name) Date :__ __/__ __ __/__ __ __ __ (DD/MMM/YYYY)

**Response:**

Response by: __ __ __ __ __ __ __ __ __ __ __ __ __ __ (Name) Date __ __/__ __ __/__ __ __ __ (DD/MMM/YYYY)

Note: Status only to be completed by Monitors or Data Management

**Status Query:**	Database updated :
O Resolved on Date __ __/__ __ __/__ __ __ __ (DD/MMM/YYYY)<br>O Re-raised with DCF N° __ __ __ __	O Yes on Date __ __/__ __ __/__ __ __ __ (DD/MMM/YYYY)

SOP-WP6-DATA-02-V2-04Mar2014-Annex5

DCF N°:<br><br>_ _ _ _	Participant N°:<br><br>_ _ _ _ _-_ _	Date DCF<br>Raised by DM:<br>_ _/ _ _/ _ _ _ _	Date DCF<br>responded by site<br>_ _/ _ _/ _ _ _ _	Date DCF<br>resolved by DMC:<br>_ _/ _ _/ _ _ _ _	DCF re-raised:<br><br>Yes/No	Re-raised with DCF N°:<br><br>_ _ _ _

Evaluation of Rapid Diagnostic Tests in association with clinical and laboratory predictors for the diagnosis of Neglected Tropical Diseases

DATA BACKUP

Procedure:

- **Make a daily backup of the study data.**
- Use following format: NIDIAG_FEVER_DB_v1.0_date.
- Keep the backup, from different dates, at a server or study computers (in the folder 'Backup') and also at an external memory device (e.g. USB stick, DVD, …).
- Complete this form after each backup. Keep the study computers and external memory device(s) at a safe place. Only authorized study personnel might have access to the study computers and external memory device(s).
- Use the study computers and external memory device(s) only for professional reasons, in particular for the NIDIAG project.
- Provide the backup to the Coordinating Data Management at ITM each Friday, unless decided otherwise.

Month:…………………………….……………………………

Date	Backup Done?		If No, why not?	Staff Initials
	Yes	No		
1				
2				
3				
4				
5				
6				
7				
8				
9				
10				
11				
12				
13				
14				
15				
16				
17				
18				
19				

	**SOP Title:** Obtaining informed consent
	Project/study: This SOP applies to all NIDIAG studies

1. Scope and application

Before a patient decides to take part in a NIDIAG study, he/she should be informed of the study aim and objectives, the required clinical examinations, and the benefits and risks related to his/her participation. If the patient confirms his/her willingness to participate in the study, he/she should sign and date the Informed Consent Form (ICF).
The objective of this standard operating procedure (SOP) is to outline the procedures and responsibilities for obtaining the freely given informed consent from participants in NIDIAG studies.

2. Responsibilities

Function	Activities
Site investigator	- Ensuring that the patient receives the full information that allows him/her to take an informed decision, and that he/she has fully understood it.<br>- Obtaining and documenting the freely given informed consent of the patient (and/or parent/guardian/legal representative) before he/she undergoes any study-related procedures (including screening procedures) which are not part of routine clinical care.<br>- Storing ICF in appropriate place<br>- Documenting consent withdrawals during the study
Site Quality Manager and External Monitor	- Verifying that the ICF is completed, dated and signed by the investigator and the patient (and/or parent/guardian/ legal representative)<br>- Verifying consent withdrawals have been properly documented

3. Procedures

3.1 Timing

- Obtain informed consent from all patients (or parent/guardian/legal representative) prior to any study specific tests or evaluations, including screening procedures, which fall outside the routine medical care available locally for the condition under study.

3.2 Information and discussion

3.2.1 Information

- Give the patient (and/or parent/guardian/legal representative) adequate, full and comprehensive information about the study, according to the information contained in the Informed Consent Form (ICF) and according to the principles of the Helsinki Declaration, WHO and ICH Guidelines and any applicable national regulations.
- In particular, explain the following:

- The study purposes;
- The study procedures, and in particular, all additional diagnostic procedures to be conducted according to the study protocol;
- The duration of the participation for the participant, the number of study visits and the duration of the follow-up period according to the study protocol;
- The risks or inconveniences to the participant, including the risks related to diagnostic procedures that are not part of the routine diagnostic workup (ex. extra blood sampling, etc…);
- The expected benefits for the patient, if any, and the expected benefits for the community;
- The alternative procedure/s that are available, and their benefits and risks;
- The reimbursement for travel expenses, as described in the protocol;
- That the subject (and/or their parent/guardian/legal representative) may refuse to participate or withdraw from the study, at any time (also during the trial), without penalty or loss of benefits;
- Mechanisms of indenisation in case of harm derived from the participation in the study;
- The fact that any trial records identifying the subject will be kept confidential and that the subject's identity will remain confidential also when the results of the trial are being published;
- The fact that the subject (and/or their parent/guardian/legal representative) will be informed if new information becomes available that might influence their willingness to continue participation in the study;
- The person/s to contact for more information on the study;

3.2.2 Language

- Give information in simple, lay language which is understandable to the patient (and/or parent/guardian/legal representative). Avoid complicated medical terms.
- Use the patient's mother tongue whenever possible.
- Allow sufficient time for the patient (and/or parent/guardian/legal representative) to read the ICF and to ask questions about the study. Address all his/her questions.
- Make sure the patient does not feel obliged to participate in the study.

3.2.3 Illiteracy

- If the patient (and/or parent/guardian/legal representative) is illiterate, an impartial independent witness must be present throughout the entire informed consent discussion.
- Assess on a case-by-case basis, the need for an independent witness, even for patients who have received some degree of formal education.

3.3 Legally incompetent patients

3.3.1 Chronic situations

- For legally incompetent patients (physically or mentally incapable of giving consent, or minors), the consent of a legally authorized representative will be obtained. It is up to the Investigator to verify that the person is legally empowered according to national laws and regulations.
- Whenever possible, for instance in case of adolescents, also the assent from the patient will be obtained, to ensure that he/she is not forced to research participation.

3.3.2 Emergency situations

- In case the patient is not legally incompetent but he/she is admitted in a critical medical condition which impairs his/her ability to decide, the informed consent procedure should not delay care.

- Therefore, under emergency conditions, the consent of a legally authorized representative will be initially obtained. It is up to the Investigator to verify that the person is legally empowered according to national laws and regulations.
- Once the emergency is over and the patient feels better, however, the consent of the patient should be obtained. If the patient does not accept to consent, any information already collected that concerns him/her will be excluded from the study data.

3.4 Signature and documentation

- After explanation and discussion, the patient (and/or parent/guardian/legal representative) should confirm his/her consent in written, by dating and signing the ICF.
- The investigator who conducted the interview or the qualified person delegated by the investigator for the informed consent procedure who conduct the interview, should also sign and date the ICF.
- If the patient is illiterate, a thumb-print can replace the signature, provided that an impartial witness has been present during the entire informed consent discussion. The impartial witness must also date and sign the ICF. By signing the ICF, the witness attests that the information in the ICF and any other written information were accurately explained to, and apparently understood by the patient and that informed consent was freely given.
- The ICF should be signed in two original copies. One copy will be given to the patient (or parent/guardian/legal representative) and the other will be kept in the Investigator Master File or at another secured location, only accessible to the medical study team.

3.5 After the signature

- It is the investigator's responsibility to ensure that any new information which could affect the patient's willingness to be in the study is timely provided to the patient (and/or parent/guardian/legal representative).
- If during the study, a patient (and/or parent/guardian/legal representative) decides to withdraw his/her consent, he / she is free to do so. Indicate the date/time and the reason for withdrawal in the CRF and other applicable study documents, such as the recruitment log.

4. Storage and access to the ICF

- The signed ICFs must be kept in the Investigator Master File (IMF) and locked in a safe place. The access should be strictly restricted to the study medical staff.
- Do not file the ICF in the Case Report Form (CRF).
- The following persons are allowed to consult the ICF:
    - the site principal investigator
    - the site investigators, clinical officers and nurses
    - the Site Quality Manager
    - the External Monitors
    - the representatives of the Ethic Committees, if required by national regulation
    - the representatives of the regulatory authorities
- The ICF must be kept together with other study documents for at least 10 years after the end of the study.

5. Definitions and abbreviations

5.1 Definitions

- Informed Consent: Informed Consent is the process by which a study participant voluntarily confirms his/her willingness to participate in a study. Only participants who have fully

understood all aspects of their participation in the study can make the appropriate judgement and give their consent to participate in the study

- Confidentiality: Maintenance of the privacy of trial subjects including their personal identity and all personal medical information.
- Investigator: Each medical person who is involved in the study conduct, and responsible for the trial and for the rights, health and welfare of the subjects in the trial.
- Impartial witness: a person who is independent form the study, who cannot be unfairly influenced by people involved with the study, who attends the informed consent process if the patient cannot read, and who reads the ICF and any other written information supplied to the patient.

5.2 Abbreviations

- CRF: Case Report Form
- ICF: Informed Consent Form
- IMF: Investigators Master File
- SOP: Standard Operating Procedure

6. Records and archives

**Appendices & Forms for completion**	
**Number**	**Title**
1	Informed Consent Form

7. Document History

Indicate previous versions of the SOP and the changes made.

**Revision**	
NA	*NA*

**Name and function**	**Date**	**Signature**
Author		
Veerle Lejon	01/02/2012	
Reviewed by		
Raffaela Ravinetto	01/02/2012	
Approved by		
Emilie Alirol	01/02/2012	

	**SOP Title:** Patient & sample numbering and labelling
	Project/study: This SOP applies to all NIDIAG studies

1. Scope and application

In order to protect research participant's confidentiality, all research records should remain anonymous. All personal information (i.e. names, telephone number, address, etc…) should be removed from the CRF, labels and other study documents, and should be replaced by a unique patient study number. This unique number is attributed upon inclusion of a research participant in a study and allows for the tracking of his/her medical information and biological specimens. This SOP describes how unique patient study numbers are created and how patient's specimens are numbered and labelled.

2. Responsibilities

**Function**	**Activities**
Site investigator	- Attributing a unique study number to each patient included in the study in accordance with this SOP<br>- Establishing a patient identification list detailing the correspondence between patient's study number and patient's name<br>- Ensuring the identification list is kept in a secure place and that access to it is restricted to the site investigator's team<br>- Ensuring that the patient study number is consistent throughout all study documents and all study samples
Site investigator or<br>Lab technician	- Attributing a unique study specimen number to each specimen collected during the study in accordance with this SOP<br>- Establishing a study specimen log detailing the study specimen number, the patient study number, the type of biological sample, and the date and time of collection
Quality Manager (except in DRC)	- Verifying that this SOP is complied with<br>- Verifying that the patient identification list and the study specimen log are correct, up-to-date, and securely stored<br>- Verifying that the patient's study number is consistent throughout all study documents and all study samples<br>- Verifying that study samples are identified and labelled in accordance with the study protocol, the patient identification list and this SOP

3. Patients Identification List

- List all patients potentially eligible to be included in the study in the "Patient Identification List" (see figure 1). There is one "Patient Identification List" per syndrome and per centre.
- Indicate the following information:
    1) Patient's name, age and sex

2) Whether the patient was included in the study or not
3) The reason for non-inclusion if applicable (ex: refused to participate, younger than 5 years old, etc...)
4) The patient study number (only patients included in the study get a study number, see below)
5) The date of inclusion in the study

Patient identification list							
Country & study centre (name, number): Nepal, Dankuta Hospital (61)							
Row nr	Patient's name (Last, First)	Age (years)	Sex (M/F)	Included (yes /no)	Reason for non inclusion	Patient Study n°	Inclusion date (dd/mmm/yyyy)
1	Dupont Francis	41	M	yes	na	61001-Fx	14/07/2012
2	Piccard Marie	4	F	no	The patient is below 5	na	na
3							
4							
5							

Figure 1: Example of patient identification list for the Fever Syndrome

4. Patient study number

- Give a unique patient study number to each patient included in one of the NIDIAG study.
- The patient study number consists of 4 fields:
  1) Country number (1 digit, see list table 1)
  2) Centre's number (1 digit, see list in table 1)
  3) Patient order number (3 digits)
  4) NIDIAG syndrome code (2 letters see list in table 2)

Ex: for the first patient included in the Neurological Syndrome clinical study in Mosango, the patient study number is 21001-Nx.

The patient study number is attributed to the patient after inclusion in the study, i.e. after checking inclusion/exclusion criteria and after the patient has signed the informed consent

4.1 Field 1 and 2: Country Number & Centre's number

The first digit of the patient study number indicates the country where the patient was included. The second digit of the patient study number indicates the study site where the patient was included.

Table 1: List of country and centre numbering (2 first digits of patient study number)

Country number	Centre number	Country	Study site
1	1	Cambodia	Sihanouk Hospital Center of HOPE
2	1	DR Congo	Hôpital rural de Mosango
3	1	Indonesia	Tulehu Hospital
3	2	Indonesia	Waai Health Center
4	8	Ivory Coast	Hôpital Méthodiste de Dabou
5	1	Mali	INRSP Reference Lab of Parasitology, Bacteriology and Virology
5	2	Mali	Niono Health Center
6	1	Nepal	Dhankuta Hospital
6	3	Nepal	BPKIHS
7	1	Sudan	Tabarak Allah Hospital

4.2 Field 3: Patient order number

In each centre, every patient included in the study (irrespective of the syndrome) gets an order number, consisting of 3 digits.

The first patient included gets number 001, the second 002 etc..

4.3 Field 4: Syndrome

The last field of the patient number consists of letters indicating the syndrome for which the patient is included.

Table 2: List of syndromes and their letter

Letter	Syndrome
Nx	Neurological
Fx	Fever
Dx	Digestive
FN or NF	Fever and Neurological
FD or DF	Fever and Digestive

4.4 Examples

1) The first patient included in Hôpital rural de Mosango in Congo gets number 21001. He is included in the Neurological syndrome clinical study, his complete patient study number becomes: 21001-Nx.
2) The second patient included in Hôpital rural de Mosango in Congo gets number 21002. He is included in the Fever syndrome clinical study, his complete patient study number is: 21002-Fx.
3) The third patient included in Hôpital rural de Mosango in Congo gets number 21003. He is included in the Neurological and then in the Fever syndrome clinical studies, therefore he gets patient study number: 21003-NF. If the patient would be included first in the Fever syndrome and then in the Neurological syndrome he would get patient study number: 21003-FN.

5. Study specimen number

All samples collected from a study patient during the study period should be identified, at all times, by a unique study specimen number. This applies to samples used for the index tests, for the reference tests, and for long-term storage.

The study specimen number consists of:

1) the patient study number
2) the specimen type abbreviation (2 letters, see table 3 below)
3) the specimen number out of the total number of specimens collected (important when more than one tube is collected).

Table 3: List of sample types and their abbreviation

Specimen type	Specimen type abbreviation
**Blood for HEmoculture**	HE
**Blood (Dry tube)**	BD
**Blood on EDTA**	BE
**Blood on Heparin**	BH
**Plasma on EDTA**	PE
**Plasma on Heparine**	PH
**SErum**	SE

**URine**	UR
**Urine Culture**	UC
**CereboSpinal fluid**	CS
**Cerebrospinal fluid Culture**	CC
**STool**	ST
**Stool Culture**	SC
**Lymph Node aspirate**	LN
**Lymph Node aspirate Culture**	NC
**Bone Marrow aspirate**	BM
**Bone marrow aspirate Culture**	BC
**Liver Abscess aspirate**	LA
**Liver Abscess aspirate Culture**	LC
**SputuM**	SM
**SputuM Culture**	MC
**Other Body Site culture**	BS
**Organisms (bacteria) isolated from Blood**	OB
**Organisms (bacteria) isolated from Cerebrospinal fluid**	OC

5.1 Examples

1) The first patient included in Hôpital rural de Mosango in Congo gets number 21001. He is included in the Neurological syndrome, his complete patient study number is: 21001-Nx.

- Blood is collected, in accordance with the study protocol:
  - for hemoculture and is numbered 21001-Nx-HE1
  - On a dry tube and is numbered 21001-Nx-BD1. If 2 tubes of serum are prepared from this blood tube, then they are numbered 21001-Nx-SE1 and 21001-Nx-SE2.
  - On Heparine and is numbered 21001-Nx-BH1. If 2 tubes of plasma are prepared from this blood tube, then they are numbered 21001-Nx-PH1 and 21001-Nx-PH2.
- Urine is also taken, and the specimen number will be 21001-Nx-UR1
- Finally four CSF samples are collected. The 4 tubes are numbered as follows:
- 21001-Nx-CS1, 21001-Nx-CS2, 21001-Nx-CS3, and 21001-Nx-CS4.

2) Specimen number 61014-Fx-PH1 indicates that this is Plasma on heparine (first tube to be collected) from the 14th patient included in Nepal at the Dhankuta Hospital, who participates in the fever syndrome.

5.2 Study Specimens Log

All specimens collected from a study patient during the study period should be recorded in a “Study Specimens Log” (see figure 2). There is one Study Specimen Log per Syndrome per centre.

The following information should be indicated:
1) Patient’s Study Number
2) Type of sample
3) Date of collection
4) Time of collection
5) Study Specimen label (only applicable when using excel sheets)

6) Person who performed the collection
7) Date of shipment
8) Organization to whom the sample is shipped
9) Person to whom the sample is shipped
10) Date of receipt of sample
11) Comments (if applicable)

Study specimen log											
Country & study centre (name, number): RD Congo, Mosango (21)											
Row nr	Patient study n°	Sample type ( )	Date collection (dd/mmm/yyyy)	Time collection (hh:mm)	Study specimen label	Collected by	Date of shipment (dd/mm/yyyy)	Sent to Organization	Sent to person	Date of receipt	Comments
1	21001-Nx	HE1	12/Mar/2012	10:24	21001-Nx-HE1-12/Mar/2012	nurse x	16/03/2012	INRB	Lunguya	17/03/2012	
2	21001-Nx	BD1	12/Mar/2012	10:24	21001-Nx-BD1-12/Mar/2012	nurse x	na	na	na	na	
3	21001-Nx	BH1	12/Mar/2012	10:25	21001-Nx-BH1-12/Mar/2012	nurse x	na	na	na	na	
4	21001-Nx	UR1	12/Mar/2012	10:45	21001-Nx-UR1-12/Mar/2012	nurse x	na	na	na	na	
5	21001-Nx	PH1	12/Mar/2012	11:00	21001-Nx-PH1-12/Mar/2012	tech y	na	na	na	na	
6	21001-Nx	PH2	12/Mar/2012	11:00	21001-Nx-PH2-12/Mar/2012	tech y	16/03/2012	ITM	Jan Jacobs	17/03/2012	
7	21001-Nx	CS1	12/Mar/2012	12:05	21001-Nx-CS1-12/Mar/2012	Dr X	na	na	na	na	
8	21001-Nx	CS2	12/Mar/2012	12:05	21001-Nx-CS2-12/Mar/2012	Dr X	na	na	na	na	
9	21001-Nx	CS3	12/Mar/2012	12:05	21001-Nx-CS3-12/Mar/2012	Dr X	16/03/2012	ITM	Jan Jacobs		lost
10	21001-Nx	CS4	12/Mar/2012	12:05	21001-Nx-CS4-12/Mar/2012	Dr X	16/03/2012	ITM	Jan Jacobs	17/03/2012	
11	21001-Nx	SE1	12/Mar/2012	15:56	21001-Nx-SE1-12/Mar/2012	tech y	na	na	na	na	
12	21001-Nx	SE2	12/Mar/2012	15:56	21001-Nx-SE2-12/Mar/2012	tech y	16/03/2012	ITM	Jan Jacobs	17/03/2012	tube damaged
13											
14											

Figure 2: An example of Study Specimen Log. This example matches the one given above under 5.1. The serum and plasma samples have been processed from the blood collected on dry tube or heparine as indicated under “comments”. For shipped date of shipment, receiving organization and person and confirmation of receipt are indicated. The other samples have been analysed on site, therefore, “not applicable (na)” is filled in.

6. Study specimen labelling

The study specimen label consists of 2 fields:

1) Specimen identification number (such as: 21001-Nx-CS1 and 61014-Fx-PH1)
2) Collection Date Format: DD-MMM-YYYY for example: 13-FEB-2012

MMM is the English abbreviation of the month, listed in table 4.

Table 4: List of the abbreviations to be used for the months

**Month**	Abbreviation
January	JAN
February	FEB
March	MAR
April	APR
May	MAY
June	JUN
July	JUL
August	AUG
September	SEP
October	OCT
November	NOV
December	DEC

Study sites are responsible for printing study specimen labels. The writing should be clear, in <u>dark permanent ink</u> and block

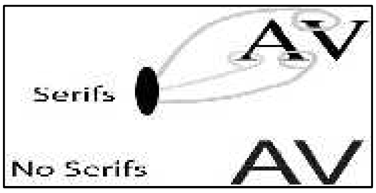

capital letters (do not use inkjet printers since this ink will be dissolved when it comes in contact with water).

The size of the letter type on the label should be at least 9-points.

The letter type of the label is preferentially a “sans serif” letter type (see figure) such as Arial

Align text left

6.1 Examples of labels

1) A label for blood EDTA sample n°1 collected from patient 115 in Tabarak Allah (Sudan) during the fever syndrome study. Date of collection 23 August 2012:

71115-Fx-BE1<br>23-AUG-2012

2) A label for urine sample n°1 collected from patient 89 in Mosango (DR Congo) during the neurological syndrome study. Date of collection 2 June 2013:

21089-Nx-UR1<br>02-JUN-2012

6.2 Correct label placement

- Large tubes (longer than label):
  1) Hold sample tube horizontally with cap in left hand.
  2) Affix patient label to be read from left to right, starting below tube cap (directly over manufacturer label).
  3) Label as high as possible on tube (To allow for maximum length of uncovered tube at bottom, facilitating placement of tubes in racks etc.)

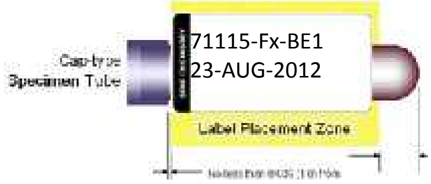

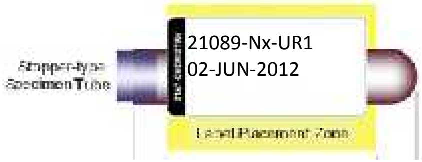

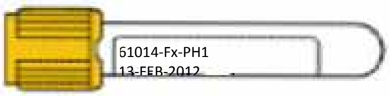

- Small tubes (shorter than label, for example cryovials):
  1) Hold sample tube vertically.
  2) Affix label, making sure that text is not overlapped.
  3) If using paper labels on tubes that will be cooled or frozen: cover the label completely with transparent tape (the tape should overlap the complete label) to avoid that the label comes off the tube.

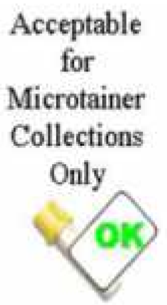

7. Records and Archives

Appendices & Forms for completion	
**Number**	**Title**
1	Patient Identification List
2	Study Specimens Log

8. Document History

Indicate previous versions of the SOP and the changes made

**Revision**	
SOP-WP6-DOC-02-V1.0-25Jun2012	Initial version
SOP-WP6-DOC-02-V1.1-09Jul2012	Translation in French
SOP-WP6-DOC-02-V2.0-21Dec2012	Addition of specimen abbreviations used in the fever syndrome
SOP-WP6-DOC-02-V2.1-18Sep2012	Addition of annexes to the French version
SOP-WP6-DOC-02-V3.0-22April2013	Deletion of Yassa Bonga and Koshi Zonal Hospital sites<br>Modification of Annex 2
SOP-WP6-DOC-02-V3.1-22Apr2013	Addition of specimen abbreviations used in the fever syndrome to the French version<br>Correction of spelling and grammar errors

**Name and function**	**Date**	**Signature**
Author		
Barbara Barbé	21/12/2012	
Revised by		
Philippe Gillet	28/12/2012	
Approved by		
Emilie Alirol	02/01/2013	

Patient identification list

Country & study centre (name, number):

Row nr	Patient's name (Last, First)	Age *(years)*	Sex (M/F)	Included (yes/no)	Reason for non inclusion	Patient Study n°	Inclusion date *(dd/mmm/yyyy)*
1							
2							
3							
4							
5							
6							
7							
8							
9							
10							
11							
12							
13							
14							
15							
16							
17							
18							

Study specimen log

Country & study centre (name, number):

Row nr	Patient study n°	Sample type (_ _ _)	Date collection (dd/mmm/yyyy)	Time collection (hh:mm)	Study specimen label	Collected by	Date of shipment (dd/mm/yyyy)	Sent to Organization	Sent to person	Date of receipt	Comments
1					--						
2					--						
3					--						
4					--						
5					--						
6					--						
7					--						
8					--						
9					--						
10					--						
11					--						
12					--						
13					--						
14					--						
15					--						
16					--						
17					--						
18					--						

	**SOP Title:** Management of study documents
	Project/study: This SOP applies to all NIDIAG clinical studies (WP2).

1. Scope and application

This procedure gives a list of essential documents to be collected by the study teams for NIDIAG clinical studies. It also describes how study-related documents should be handled, stored and archived.

2. Responsibilities

Function	Activities
Principal Investigator at each site	- Collect all essential documents pertaining to the work conducted at the study site and file them in the Site Investigator File (SIF)<br>- Ensure the IF is kept up to date<br>- Ensure all essential documents are stored under appropriate conditions and for the required period of time<br>- Make sure access to essential documents is restricted to authorized staff<br>- Make sure that copies of all essential documents originated at the site are sent to the country coordination (except documents baring patients identifiers)
Laboratory head at each site	- Collect all essential documents pertaining to the laboratory work conducted at the study site and file them in the Laboratory File (LF)<br>- Ensure the LF is kept up to date<br>- Ensure all essential documents are stored under appropriate conditions and for the required period of time<br>- Make sure access to essential documents is restricted to authorized staff<br>- Make sure that copies of all essential documents originated at the site laboratory are sent to the country coordination (except documents baring patients identifiers)
Country Coordinating Investigator	- Collect all essential documents pertaining to the work conducted in the country and file them in the Investigator Master File (IMF)<br>- Ensure the IMF is kept up to date<br>- Ensure all essential documents are stored under appropriate conditions and for the required period of time<br>- Make sure access to essential documents is restricted to authorized staff<br>- Make sure that copies of all essential documents originated at the country clinical sites and at the country coordination are shared with the WP2 Task Leader, and that the site receive all the documents which are relevant for their work
WP2 Task Leader	- Collect all essential documents pertaining to the work conducted under the specific task (i.e. Digestive Syndrome, Fever Syndrome or Neurological Syndrome) and file them in the Sponsor Master File (SMF). This responsibility can be delegated, and the delegation must be documented.<br>- Ensure the SMF is kept up to date<br>- Ensure all essential documents are stored under appropriate conditions and for the required period of time<br>- Make sure access to essential documents is restricted to authorized staff

	- Make sure that copies of all essential documents originated within the country coordinating investigators
Site Quality Manager (QM) or equivalent function, and External Monitor	- Verify this SOP is complied with by all intended users and in particular<br>a. verify the IMF, SIF and LF are complete and up-to-date<br>b. verify that access is restricted to authorized individuals<br>- Report all major or systematic deviations from this SOP to the WP2 and WP6 leaders

3. Procedures

3.1 Collection and handling of study essential documents:

A list of all documents to be collected and filed can be found at the end of this SOP. Study documents should be filed in 4 different binders:

- Laboratory File (LF): The Laboratory File includes all essential documents related to the laboratory work at the site level. There is one Laboratory File per study site. The laboratory personnel is responsible for keeping it updated and for ensuring it is stored adequately.
- Site Investigator File (SIF): The Site File includes all essential documents and forms related to the conduct of the study at the site level. There is one Site File per study site. The site investigators are responsible for keeping it updated and for ensuring it is stored adequately.
- Country Investigator's Master File (IMF): The Investigator's Master File includes all essential documents related to the conduct of the study at the country level. There is one Investigator Master File per country. The Country Coordinating Investigator is responsible for keeping it updated and for ensuring it is stored adequately.
- Sponsor Master File (SMF): The Sponsor's Master File includes all essential documents related to the conduct of the study as per ICH GCP guidelines. There is one Sponsor Master File per clinical syndrome. The Task Leader, or the delegated function at his/her institution, is responsible for keeping it updated and for ensuring it is stored adequately.

Refer to Table 1 to know which document needs to be filed in which binder.

File the documents in the order given in Table 1.

3.2 Storage and access to study documents

All documents must be stored in a secured place.

The Laboratory File should be stored in the laboratory. The Site File should be stored under responsibility of the Principal Investigator. The Investigator's Master File should be stored under the responsibility of the Country Coordinating Investigator. The Sponsor File should be stored under the responsibility of the Task Leader.

Access to study documents should be restricted to the staff involved in NIDIAG.

- The access to the Site Investigator File should be restricted to the staff involved in patient's management (study nurses, site investigators, etc…)
- The access to the Laboratory File should be restricted to the lab personnel (Lab technicians, lab head, etc…) and supervisors, including the Country Coordinating Investigator and the quality manager or equivalent function
- The access to the Investigator's Master File should be restricted to the Country Coordinating Investigator and his/her team.
- The access to the Sponsor Master File should be restricted to the Task Leader and his/her team.

The study personnel should prevent accidental destruction of study documents. This involves protection against fire and flood.

Upon request and under confidentiality agreement, site Quality Managers, study monitors, external auditors, inspectors (national regulatory authorities) and Ethics Committee should be given direct access to all study documents.

3.3 Maintenance

- All study documents should be kept up to date. The site principal investigator and the Country Coordinating Investigator should, among others, ensure that the latest versions of the protocol, Informed Consent Form (ICF), Case Report Form (CRF) and Standard Operating Procedures are used. The site investigators are responsible for keeping all documents mentioned in Annex 1 up-to-date and complete.
- The laboratory head should regularly check, among others, that laboratory normal values included in the Laboratory File correspond to the ones used for the analyses, that the study specimen log and records of retained samples are updated in accordance with study recruitment, and that shipping records of biological samples to reference labs are filed. The laboratory head is also responsible for the maintenance of all laboratory documents listed in Annex 1.
- The country coordinating investigator or his/her delegate are responsible for the maintenance of all documents of the Investigators Master File listed in Annex 1. The Country Coordinating Investigator should make sure that changes in essential documents are communicated in a timely fashion to the site principal investigator and to the WP2 Task Leader whenever relevant.
- The Task Leader or his/her delegate should ensure that the content of the Sponsor Master File is regularly updated and matches the current status of the study in each partner country.

All changes occurring during the study should be documented in the SIF, LF (ex: new staff's CV should be collected, new contracts should be established and the organizational diagram of study staff and signature sheet should be updated).

3.4 Archiving

The site principal investigator should keep the Site Investigator File until the Country Coordinating Investigator collects it at the end of the study.

The lab head should keep the Laboratory File until the Country Coordinating Investigator collects them at the end of the study.

The Country Coordinating Investigator should keep the Investigator's Master File and all other study documents for a period of time corresponding to the country's regulatory requirements and for 5 years after the end of the study.

The Task Leader should keep the Sponsor's Master File and all other study documents for a period of time corresponding to the country's regulatory requirements and for at least 5 years after the end of the study.

4. Definitions and abbreviations

Essential Documents: Documents which individually and collectively permit the evaluation of the conduct of a study and quality of the data produced. These documents are important for the successful management of the study. They are also crucial to demonstrate compliance with GCP/GCLP, protocol, SOPs and to enable quality control. A list of essential documents is provided in Annex 1.

Source Documents: These are original documents. They consist of data relevant to the current research such as medical records, administrative files, laboratory reports, consultation reports, pharmacy dispensation registers, etc...

CRF: Case Report Form

ICF: Informed Consent Form

IMF: Investigator Master File

LF: Laboratory File

SIF: Site Investigator File

SMF: Sponsor Master File

SOP: Standard Operating Procedure

5. Records and archives

**Appendices & Forms for completion**	
**Number**	**Title**
NA	NA

6. Document History

**Revision**	

**Name and function**	**Date**	**Signature**
Author		
Emilie Alirol	10/05/2012	
Reviewed by		
Raffaella Ravinetto	14/05/2012	
Approved by		
Rosanna Peeling	24/05/2012	

Table 1: List of essential documents to be filed in the SIF, LF

Title of document	SIF	LF	IMF	SMF
**1. Study Documents**				
Signed study protocol and amendments if any	x	x	x	x
Sample Case Report Form (CRF)			x	x
Sample Informed Consent Form (ICF) as well as any written information given to study participants	X		x	x
Study SOPs	x	x	x	x
Nidiag ethical charter	X		X	X
**2. Regulatory Documents**				
All correspondence with ITM IRB and EC of Antwerp University Teaching Hospital (UZA) including final approval of the study protocol and its amendments	x			x
All correspondence with national IRB/EC including final approval of the protocol and its amendments	x		x	x
Approval from the national regulatory authorities (where required)	x		x	x
Interim reports to ECs/IRBs			x	x
Import authorizations for RDTs (if required)			x	x
Insurance statement			x	x
**3. Study Staff and Contracts**				
Delegation log (updated with dates and signatures) of all study staff at the study site, including the lab	x		x	
Delegation log (updated with dates and signatures) of all study staff at the Sponsor level				x
Site contact list	X		x	x
Sponsor contact list (per each protocol)	X		X	X
CVs of all personnel involved in the study (investigators, sub-investigators, lab technicians, etc…)	x		x	x
Signature sheet of all authorized staff at study site, including the lab	x		x	x
GCP/GCLP training certificate of all personnel involved in the study, including the lab	x		x	x
Copy of NIDIAG grant agreement			x	x
Signed agreement between study personnel and partner institution			x	x
4. **Management of RDTs**				
Shipping records of RDTs (including documents for custom clearance)	x		x	x
RDT accountability at study site		x	x	
5. **Biological Samples**				
Country-specific normal values / range for laboratory procedures		x	x	x
Documents pertaining to Quality Control of Laboratory Procedures		x	x	
Shipping records for biological samples (to national and international reference labs)		x	x	
Study specimen logs, including Record of retained biological samples		x	x	
Country-specific sample flow diagram		x	x	x
RDTs results form		x[2]	x[1]	
Completed Temperature Logs		x		

**6. Monitoring**				
Monitoring Plan			x	x
Study Initiation Visit Report or letter of follow up	x		x	x
Monitoring Visit Reports or letter of follow-up				x
Close-out Monitoring Report or letter of follow up				x
**7. Study participants**				
Patient Identification List	x		x	
All signed Informed Consent and Assent forms	x			
Source Documents (including medical files and lab investigations results)	x(2)	x(2)	x(1)	
Signed, dates and completed CRFs	x(1)		x(2)	
**8. Correspondence**				
All essential correspondence between TMG and/or country coordinating investigator and/or the site(s)	x	X	x	x

(1) Copies (2) Originals
SIF: Site Investigator File; LF: Lab File; IMF: Investigator Master File; SMF: Sponsor Master File

SOP-WP6-QUAL-01-V1-02Nov2011

nidiag Better Diagnosis for Neglected Infectious Diseases	**SOP Title:** SOP on SOP
	Study title: NA (This SOP applies to all NIDIAG studies)

1. Scope and application

This procedure provides a guideline on how to write a Standard Operating Procedure (SOP), including how to format the document. The purpose of a SOP is to provide detailed instructions on how to carry out a task so that any team member can carry out the task correctly every time. The purpose or objective of a SOP should restate and expand a well-written title. A well-written SOP will facilitate training. The best SOP is one that accurately transfers the relevant information and facilitates

compliance with reading and using the SOP. This SOP for SOPs is aimed at WP leaders, task leaders and all those who will be involved in SOP writing. It applies to all SOPs developed within the NIDIAG consortium.

2. Responsibilities

Function	Activities
SOP author	Draft SOP in consultation with the intended users<br>Correct SOP according to WP6 feedback<br>Make SOP available to intended users<br>Amend SOP if required
WP6 representative	Review SOP initial draft and consecutive amendments<br>Release and formally approve SOP versions<br>Make SOP available to the consortium through NIDIAG website
Site Quality Manager and External Monitor	Ensure compliance of SOP by all intended users<br>Ensure the SOP version used is the most recent one approved by WP6<br>Report non compliance to PI and WP6

3. Procedures

3.1 Writing a Standard Operating Procedure (SOP)

- Write one SOP per study-related activity. Ex: Performance of lumbar puncture, Handling, transport and storage of CSF samples, Microscopic detection of trypanosomes etc… Do not mix too many activities in one SOP.
- Make sure you are familiar with the procedure to be described in the SOP. If you are not, ask somebody who performs the procedure regularly to show it to you. Have this person read your first draft before you send it to WP6 for review
- Describe in details how the procedure is being carried out
- List the steps in a chronological order as in the example below

<u>Making a cup of tea:</u>

1. Collect a cup and saucer
2. Place teabag into cup

3. Boil water in kettle

4. Add water to cup and teabag

5. Allow tea to infuse

6. Remove teabag

7. Add milk and sugar (if desired)

- Use a simple, active language e.g. ‘weigh 10 mg’ rather than ’10 mg should be weighed’
- Indicate in the “Responsibilities” section who is doing what. Do not use people’s name, use functions / job title e.g. laboratory technician, physician…
- Include all necessary information to perform the procedure, not more.
- Use the fewest possible words, if different steps are involved in the activity, use bullet points
- If possible add visual displays (VD) such as diagrams, flow charts, pictures or table
- Have a specific reader in mind. Know the type of person who will be reading the procedure and tailor the writing according to the end user.
- Avoid “do this or alternatively do that”
- Avoid “where appropriate”
- Make sure all technical terms and acronyms are defined under the “Definition” section

3.2 SOP Format

- Use the SOP template provided by WP6. The template is available on the NIDIAG website (http://www.nidiag.org/)
- Each SOP should have a unique identifier which includes:
  - the number of the Work package under which the SOP is being developed
  - an acronym referring to the type of the procedure (LAB: for laboratory SOP, DOC: for SOP related to documentation management, CLIN: for clinical SOP, DATA: for SOP related to data management, QUAL: for SOP related to Quality Assurance (QA) and Quality Control (QC))
  - the number of the SOP

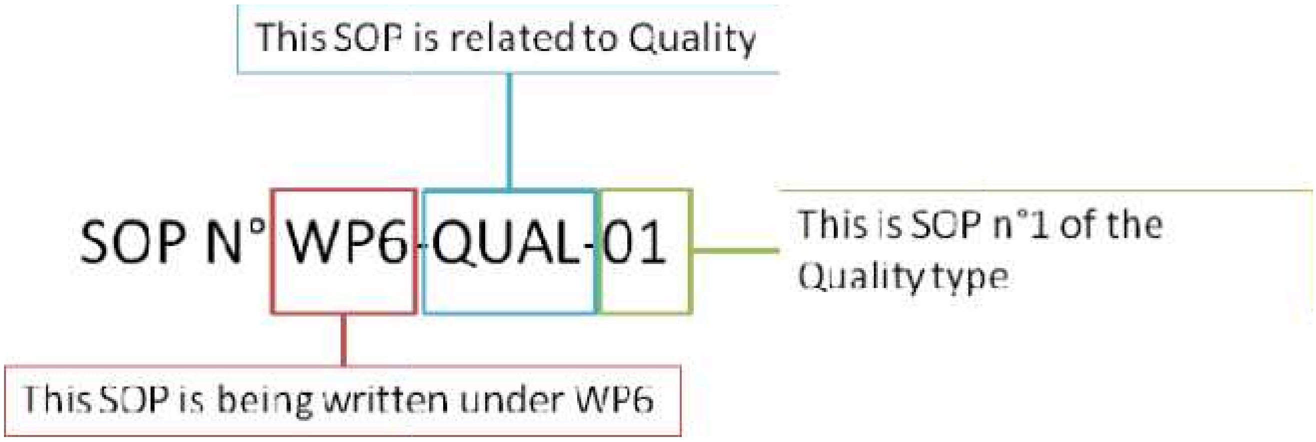

- If the procedure is a lengthy one, then the description of the procedure can be split up and placed under smaller headings. e.g. '3.1 Materials, 3.2 Preparation of reagents, 3.3 Operation and maintenance, …'
- On each page of the SOP indicate:
  - The SOP number, the version number and version date
  - The page number and the total number of pages

3.3 SOPs review and version control

- Each SOP should be reviewed by a WP6 authorized representative. The first draft should be circulated as version 0. Comments and corrections from WP6 should be incorporated in this draft to create version 1. WP6 is responsible for the final approval (final OK) of the document.
- The SOP should be signed by the SOP author and by the person who reviews and approves it.
- Corrections and modifications of the initial version should also be submitted for review to WP6. Version 2 should be created only after WP6 feedback and approval has been obtained.
- Each consecutive version and reason for/description of each modification made to the SOP should appear in the "Document History" section at the end of the SOP.

3.4 SOPs language

SOPs will be written in English. However, SOPs intended to French-speaking users will be translated to French.

4. Definitions

Principal Investigator (PI): Person who is responsible for the overall conduct of the study at a given site. The principal investigator is the responsible leader of the team involved in NIDIAG.

Standard Operating Procedures (SOPs): SOPs are issued to specifically instruct employees / team members in areas of responsibility, Work Instructions, appropriate specifications and required records. SOPs outline procedures, which must be followed to claim compliance with GCP and GCLP principles or other Statutory rules and regulations. Procedures can take the form of a narrative, a flow chart, a process map, computer screen printouts or combination of all or any other suitable form, however must be written in appropriate, effective grammatical style. (e.g. plain English).

Form: A form is a document which is to be printed at the time of use and filled out for the purpose of becoming a record (e.g. Library Log Form), or for the purpose of becoming Visual Display tool.

Template: A template is a form to be used as a model for creating other documentation

Visual Display (VD): A VD is a form requiring no additional data to be added, (i.e. no written record) which provides visual information to instruct in the process, e.g. "Out of Order" tag stuck on a machine. The information can be in the form of pictures or photographs; flowchart; operating instructions; or a notice. The Visual Display form is usually located in a permanent position, however maybe in use for a specific period of time, e.g. for a single batch. Pages from a single Visual Display form must be located together in a specified location.

5. Records and archives

Appendices & Forms for completion	
**Number**	**Title**
NA	*NA*

6. Document History

Revision	
-	-

Name and function	Date	Signature
Author		
Emilie Alirol	24/10/2011	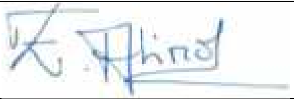
Reviewed by		
Raffaella Ravinetto<br>Rosanna Peeling	27/10/2011	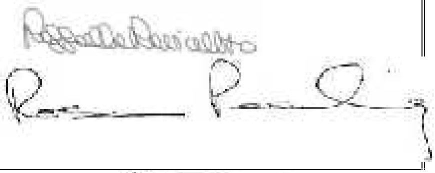
Approved by		
Veerle Lejon	07.11.2011	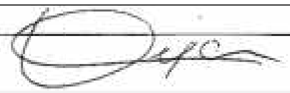

	**SOP Title:** External monitoring
	Project/study: this SOP applies to all NIDIAG clinical studies (WP2).

1. Scope and application

Monitoring visits are conducted throughout NIDIAG clinical studies to ensure that:

1) the rights and well-being of research subjects are protected
2) the study is conducted in accordance with GCP and regulatory requirements and with the study protocol
3) study data are accurate, complete and verifiable from source documents.

This procedure describes how external monitoring visits should be carried out, documented and followed up, in compliance with WHO and ICH Good Clinical Practices Principles.

2. Responsibilities

Function	Activities
External Monitor	- Plan monitoring visits in accordance with the monitoring plan, as agreed with the WP6 leader<br>- Verify that the trial is conducted in compliance with international and national regulatory/ethical requirements, with the study protocol and amendments, and with the NIDIAG SOPs<br>- Report any deviations in the monitoring visit report<br>- Send the monitoring visit report to the concerned WP6 leader
Quality manager (QM) or equivalent function	▪ Support the external monitor in the fulfillment of his/her tasks<br>▪ Attend monitoring visits
Site PI	▪ Support the external monitor in the fulfillment of his/her tasks<br>▪ Attend monitoring visits and ensure all key-study staff are present<br>▪ Ensure that the external monitor has full access to the study documents and facilities<br>▪ Ensure that the corrective actions listed in the monitoring visit report and/or follow-up letter are implemented
Country coordinating investigator or his/her delegate<br><br>WP2 leader or his/her delegate	▪ Verify that the corrective actions listed in the monitoring visit report and/or follow-up letter are implemented (joint follow-up will take place within the clinical trial-specific Trial Management Groups)
WP6 leader or his/her delegate	▪ Be the link between the Trial Management Group and the monitor<br>▪ Establish monitoring plan for each study site

	▪ Ensure that the Monitor receives any new key-information relevant to the study status and conduct<br>▪ Review the monitoring visit reports and/or the follow-up letter.<br>▪ Send timely a copy of the monitoring visit report and/or follow-up letter to WP2 leader or his/her delegate, the Country coordinating investigator, and to the concerned members of the Trial Management Group<br>▪ Send a copy of the monitoring visit report and/or follow-up letter to the site PI

3. Procedures

3.1 Monitoring schedule and visit planning

The WP6 leader, in consultation with the Country Coordinating Investigator, the WP2 leader and other concerned members of the Trial Management Group, will determine the appropriate calendar of monitoring visits for each clinical study l (e.g. country monitoring plan).

The monitor is responsible for planning monitoring visits in accordance with the monitoring plan.

Prior to visit, the monitor should:

- Review the study protocol, CRF, monitoring tools (Monitoring Visit Report and/or Follow-up Letters, NIDIAG SOPs…) and previous reports, as well as any other key documents provided by the WP6 leader.
- Inform the WP6 leader and the site PI at least 2 weeks ahead of the monitoring visit, and ask them to ensure that all key-staff are present for the visit.
- Send a provisional visit agenda, agreed with the WP6 leader, to the site PI.
- Discuss with the WP6 leader about the latest developments of the study and agree on communication streamlines, so that the WP6 leader can contact the Monitor if needed for the follow up of the visit's findings.
- Enquire about the number of patients included in the study site since last visit. Make sure that enough time is allowed during the visit to complete all monitoring activities.

3.2 Study initiation visit

It is performed after receipt of all study materials, after approval of the EC and CA, and prior to the start of recruitment. At least the following activities must be carried out:

Ethical and GCP training: the Monitor should meet the site PI and study staff, to verify that they have an appropriate understanding of ethical and GCP requirements. If needed, he/she should conduct a GCP training or refreshment session.

Protocol and CRF review: the Monitor will review the protocol and the CRF with all the Investigators and co-workers.

Site Investigator's file and Laboratory File: the Monitor should verify the completeness of the Site Investigator's file and Laboratory File (ref. SOP No WP6-DOC-03 on Management of Study Documents). If logistically feasible and if time allows it, the Monitor should also check the completeness of the Investigator Master File.

Visit of site's facilities: the Monitor should visit the site's facilities, including the laboratory, to confirm that they are adequate for the conduct of study activities. He/she should also check that there are appropriate spaces and facilities for storage of the study materials and for archiving the site investigator's file and source documents.

Reporting: the Monitor will document all the above in a Monitoring Visit Report, which will be sent to the WP6 leader/delegate. It is the responsibility of the WP6 leader to timely distribute it to the country coordinating investigator/delegate, to the WP2 leader/delegate, to other concerned members of the Trial Management Group and to the site Principal Investigator for follow up on findings and corrective actions.

Exceptions: the WP6 leader may decide to skip the study initiation visit, if all the points have been carried out and documented on previous visits, and corrective actions have been implemented.

3.3 Routine Monitoring visits

The first monitoring visit takes place as early as possible, and no later than one month after recruitment started, so that any major mistakes or systematic problems may be identified and corrected in the early phases. At least the following activities should be carried out:

Follow up of previous visit: the Monitor will follow-up any pendings from the previous visit/s.

New staff: if new staff have been appointed, the Monitor will check the CV, delegation log and training records. If needed, he/she will carry out a GCP training/refreshment session.

Progress of the trial: the Monitor will discuss the recruitment status (planned vs. achieved) with the Site Principal Investigator. He/she will also check if the patients' referral process is working satisfactorily.

Informed consent: the Monitor will check informed consent has been obtained and properly documented for each patient prior to undergoing any Study-specific procedures (ref SOP No WP6-DOC-01 on Informed Consent).

Compliance with the protocol: the Monitor will verify the overall compliance with the protocol and amendments. Any serious and/or any systematic deviations will be brought to the attention of the country coordinating investigator and WP2 leader. Corrective action must be planned.

CRF: the Monitor will provide guidance and support on the correct procedures for filling the CRF and (when appropriate) for performing data entry.

Source data verification The Monitor will review the patients' files, to verify that the information entered in the CRF match the original observations in the hospital files and in the laboratory print-outs, and that it is consistent and accurate. The SDV is carried out on a sample basis (e.g., on 15% of all CRF) and percentage must be increased if the findings are not satisfactory.

Site Investigator's file and Laboratory file: the Monitor will verify completeness of the Investigator's study file and of the Laboratory file (ref. SOP No WP6-DOC-03 on Management of Study Documents)

Rapid diagnostic tests: the Monitor will check that the storage, transportation and dispensing conditions are appropriate, and will check that the expiry dates have not been exceeded. He/she will review the drug accountability forms.

Reporting: the Monitor will document all the above in a Monitoring Visit Report, which will be sent to the WP6 leader/delegate. It is the responsibility of the WP6 leader to timely distribute it to the country coordinating investigator/delegate, to the WP2 leader/delegate, to other concerned members of the Trial Management Group, and to the site Principal Investigator for follow up on findings and corrective actions (the WP6 leader may choose to send to the PI either a follow-up letter summarizing the main findings and corrective actions needed, or a copy of the Visit Report itself).

3.4 Close-out visit

It is performed after the last visit has been completed, all data have been entered in the database and all queries resolved. At least the following activities should be carried out:

Follow up of previous visit: the Monitor will follow-up all pendings from the previous visits. A plan of action will be agreed for corrective actions that cannot be implemented during the visit.

Final trial assessment: the final number of patients screened, recruited, drop-out and completed, must be verified and attached to the close-out monitoring visit report.

Unused study rapid diagnostic tests: the Monitor will check the accountability forms and will perform a final inventory, to check that there are no discrepancies. The tests that are registered in the country can be given to the Hospital Pharmacy. For non-registered products, arrangements will be defined by the WP6 leader.

Site Investigator's file: the Monitor will verify completeness of the Site Investigator's file, and will check that it is moved to the place of the country coordinating investigator, to be available for audits or inspections (ref. SOP No WP6-DOC-03 on Management of Study Documents).

Reporting: the Monitor will document all the above in a Close-Out Visit Report, which will be sent to the WP6 leader/delegate. It is the responsibility of the WP6 leader to timely distribute it to the country coordinating investigator/delegate, to the WP2 leader/delegate, to other concerned members of the Trial Management Group and to the site Principal Investigator for follow up on findings and corrective actions (the WP6 may choose to send to the Principal Investigator either a follow-up letter summarizing the main findings and corrective actions needed, or a copy of the Visit Report itself).

Exceptions: the WP6 leader may decide to skip the close-out visit, provided that all the above points have been carried out and documented on previous visits (e.g., by the laboratory supervisor) and that corrective actions have been implemented.

3.5 Note on laboratory supervision

Most clinical monitors do not have a specific laboratory expertise. Because of the characteristics of the Nidiag project, external clinical monitoring must be complemented by regular supervision visits carried out by the lab experts.

4. Definitions and abbreviations

Audit A systematic examination, carried out independently of those directly involved in the clinical trial, to determine whether the conduct of the trial complies with the protocol and whether the data reported are consistent with the records on site.

Case-Report Form A document used to record data on each trial subject during the trial, as defined by the protocol. The data should be collected by procedures which

guarantee preservation, retention and retrieval of information and allow easy access for verification, audit and inspection.

Compliance Adherence to all the research related requirements, Good Clinical Practice requirements, and the applicable regulatory requirements.

Good Clinical Practice A standard for clinical studies which encompasses the design, conduct, monitoring, termination, audit, analyses, reporting and documentation of the studies and which ensures that the studies are scientifically and ethically sound and that the clinical properties of the pharmaceutical product (diagnostic, therapeutic or prophylactic) under investigation are properly documented.

Informed consent A subject's voluntary confirmation of willingness to participate in a particular trial, and the documentation thereof. This consent should only be sought after all appropriate information has been given about the trial including an explanation of its status as research, its objectives, potential benefits, risks and inconveniences, alternative treatment that may be available, and of the subject’s rights and responsibilities in accordance with the current revision of the Declaration of Helsinki.

Inspection An officially-conducted examination by relevant authorities at the site of investigation and/or at the site of the sponsor in order to verify adherence to Good Clinical Practice.

Investigator Each medical person who is at some stage involved in the study conduct, and responsible for the trial and for the rights, health and welfare of the subjects in the trial. The investigator should have qualifications and competence in accordance with local laws and regulations as evidenced by up-to-date curriculum vitae and other credentials.

Monitor A person appointed by, and responsible to, the sponsor for the monitoring and reporting of progress of the trial and for verification of data.

Patient/subject file A collection of data consisting of all relevant information on the patient or subject (such as hospital file, consultation records or special subject file) that permits the authenticity of the information presented in Case-Report Forms to be verified and, where necessary, completed or corrected.

Principal investigator The investigator serving as coordinator within each study site.

Protocol A document which states the background, rationale and objectives of the trial and describes its design, methodology and organization, including statistical considerations, and the conditions under which it is to be performed and managed. The protocol should be dated and signed by the investigator, the institution involved and the sponsor.

Protocol amendment A written description of a change to or a formal clarification of the protocol.

Source data All records or certified copies of original observations, clinical findings or other activities in a clinical trial necessary for the reconstruction and evaluation of the trial. Such material includes laboratory notes, memoranda, calculations and documents, as well as all records of data from automated instruments or exact, verified copies in the form of photocopies, microfiches etc. Raw data can also include photographic negatives, microfilm or magnetic media (e.g. computer diskettes).

Source data verification The procedures carried out to ensure that the data contained in the final study report match original observations. These procedures may apply to raw data, data in Case-Report Forms (in hard copy or electronic form), computer print-outs and statistical analyses and tables.

Trial Management Group The entire group of individuals and functions that are jointly responsible for the appropriate set up, conduct and daily management of the clinical trial in WP2.

AE	Adverse Event
CA	Competent Authorities
CRF	Case-Report Form
CV	Curriculum Vitae
EC	Ethics Committee
GCP	Good Clinical Practice
ICH	International Conference for Harmonisation
SDV	Source Data Verification
SOP	Standard Operating Procedure
TMG	Trial Management Group
WHO	World Health Organisation

5. Records and archives

Write in this section the records that need to be filled when following the procedure, and where these records will be stored and eventually archived. This can include a lab book for example, or a checklist,

**Appendices & Forms for completion**	
**Number**	**Title**
1	Monitoring visit report

6. Document History

**Revision**	
V2	Compared to the first version of this SOP (dated 7 Jul 2012), the WP6 now becomes the central contact for the external monitor, in compliance with the ICH GCP (the WP6 represents the sponsor).

**Name and function**	**Date**	**Signature**
Author		
Raffaella Ravinetto	05/12/2012	
Reviewed by		
Emilie Alirol	05/12/2012	
Approved by		
Rosanna Peeling	05/12/2012	

REPORT OF MONITORING VISIT N°			
Syndrome:			
Study protocol n°:			
Country Coordinating Investigator:			
Study Site :			
Country :			
Monitor :		Visit date :	Previous Visit:
Visit type	☐ Initiation	☐ Routine	☐ Close-out

STAFF					
	Name	Available at this visit	CV in the SIF?	GCP training?	Signed delegation log?
Principal Investigator		☐yes ☐no	☐yes ☐no	☐yes ☐no	☐yes ☐no
Sub-Investigator		☐yes ☐no	☐yes ☐no	☐yes ☐no	☐yes ☐no
Sub-Investigator		☐yes ☐no	☐yes ☐no	☐yes ☐no	☐yes ☐no
Sub-Investigator		☐yes ☐no	☐yes ☐no	☐yes ☐no	☐yes ☐no
Study nurses		☐yes ☐no	☐yes ☐no	☐yes ☐no	☐yes ☐no
Study nurses		☐yes ☐no	☐yes ☐no	☐yes ☐no	☐yes ☐no
Study nurses		☐yes ☐no	☐yes ☐no	☐yes ☐no	☐yes ☐no
Head of Lab		☐yes ☐no	☐yes ☐no	☐yes ☐no	☐yes ☐no
Lab technician		☐yes ☐no	☐yes ☐no	☐yes ☐no	☐yes ☐no
Lab technician		☐yes ☐no	☐yes ☐no	☐yes ☐no	☐yes ☐no
Lab technician		☐yes ☐no	☐yes ☐no	☐yes ☐no	☐yes ☐no
Quality Manager		☐yes ☐no	☐yes ☐no	☐yes ☐no	☐yes ☐no
Data management staff		☐yes ☐no	☐yes ☐no	☐yes ☐no	☐yes ☐no
Other staff					
		☐yes ☐no	☐yes ☐no	☐yes ☐no	☐yes ☐no
		☐yes ☐no	☐yes ☐no	☐yes ☐no	☐yes ☐no
		☐yes ☐no	☐yes ☐no	☐yes ☐no	☐yes ☐no

STAFF INVOLVED IN THE TRIAL	
Is the study team adequately trained and motivated to conduct the study?	☐yes ☐no ☐NA
Is there sufficient time allocated to the study team to conduct the study efficiently?	☐yes ☐no ☐NA
Was there any change in staff since the previous visit?	☐yes ☐no ☐NA

Please document change;	
Has a signed and dated CV been filed in the SIF and collected for the IMF?	☐yes ☐no ☐NA
Has the site delegation log been updated?	☐yes ☐no ☐NA
Did the new staff member follow a documented GCP training?	☐yes ☐no ☐NA
Has this person been sufficiently trained in all study documents?	☐yes ☐no ☐NA
Additional Comments	

GENERAL DISCUSSION	
HAS THE FOLLOWING BEEN DISCUSSED WITH THE PI AND RELEVANT STUDY STAFF?	
Protocol/amendment?	☐yes ☐no ☐NA
ICF and informed consent process?	☐yes ☐no ☐NA
Investigator and study staff responsibilities?	☐yes ☐no ☐NA
Handling and storage of CRF and Source Documents?	☐yes ☐no ☐NA
Data collection?	☐yes ☐no ☐NA
Participant's follow-up?	☐yes ☐no ☐NA
Biological samples handling, transport and storage?	☐yes ☐no ☐NA
Accountability of RDTs and other study materials?	☐yes ☐no ☐NA
Additional Comments	

MASTER FILES	
SITE INVESTIGATOR FILE (SIF)	
Are all essential documents filed in the SIF?	☐yes ☐no ☐NA
Was the SIF reviewed with the PI and relevant study staff?	☐yes ☐no ☐NA
Were copies of all site specific documents collected for the IMF?	☐yes ☐no ☐NA
Is the SIF kept in an appropriate place?	☐yes ☐no ☐NA
Is it possible to lock the place?	☐yes ☐no ☐NA
Is the access restricted to authorized staff?	☐yes ☐no ☐NA
LABORATORY FILE (LF)	
Are all essential documents filed in the SIF?	☐yes ☐no ☐NA
Was the LF reviewed with the lab head and relevant study staff?	☐yes ☐no ☐NA
Were copies of all site specific documents collected for the IMF?	☐yes ☐no ☐NA
Is the LF kept in an appropriate place?	☐yes ☐no ☐NA
Is it possible to lock the place?	☐yes ☐no ☐NA
Is the access restricted to authorized staff?	☐yes ☐no ☐NA
COUNTRY INVESTIGATOR MASTER FILE (IMF)	
Are all essential documents filed in the SIF?	☐yes ☐no ☐NA
Was the LF reviewed with the lab head and relevant study staff?	☐yes ☐no ☐NA
Is the IMF kept in an appropriate place?	☐yes ☐no ☐NA
Is it possible to lock the place?	☐yes ☐no ☐NA
Is the access restricted to authorized staff?	☐yes ☐no ☐NA

Additional Comments	

RECRUITMENT (FOR ROUTINE AND CLOSE-OUT MONITORING VISITS ONLY)	
RECRUITMENT NUMBERS	
Is the Patient Identification List sent to the country coordinating investigator on a regular basis?	☐yes ☐no ☐NA
Are the Screening and recruitment log sent to the country coordinating investigator and to the sponsor (WP2 leader) on a regular basis?	☐yes ☐no ☐NA
Number of patients included*	
Number of patients withdrawn/dropped out*	
Is reaching the recruitment target by the end of the recruitment period attainable?	☐yes ☐no ☐NA
If no; what actions will be taken to increase recruitment?	
Additional Comments	
OVERALL CIRCUIT OF PATIENTS	
How is screening organized?	
How is recruitment organized?	
Is patient's referral working satisfactorily?	☐yes ☐no ☐NA
If not, explain:	
Is patient's follow-up working satisfactorily?	☐yes ☐no ☐NA
If not, explain:	
Do study participants have access to proper care?	☐yes ☐no ☐NA
If not, explain:	
Do screened but non-recruited patients have free access to all diagnostic procedures?	☐yes ☐no ☐NA
If not, explain:	
Additional Comments	

PROTOCOL, ICF & CRF	
INFORMED CONSENT FORMS (ICF)	
Does the study team use the latest version of the ICF?	☐yes ☐no ☐NA
Is the informed consent procedure conducted properly?	☐yes ☐no ☐NA
If no, was the site staff retrained in the ICF procedure?	☐yes ☐no ☐NA
If yes, who attended this training?	
Were ICF checked?	☐yes ☐no ☐NA

If yes, from patient n° until patient n°

Where all ICF correctly completed? ☐yes ☐no ☐NA

If no, please list deviations:

patient n° Deviation:

patient n° Deviation:

Patient(s) n°
Deviation:

patient(s) n°
Deviation:

patient(s) n°
Deviation:

patient(s) n°
Deviation:

patient(s) n°
Deviation:

patient(s) n°
Deviation:

PROTOCOL

Was the overall compliance of the research conduct with the protocol and amendments verified? ☐yes ☐no ☐NA

Have protocol deviations been detected? ☐yes ☐no ☐NA

If yes, please list deviations:

Patient(s) n°
Deviation:

patient(s) n°
Deviation:

patient(s) n°
Deviation:

patient(s) n°
Deviation:

patient(s) n°
Deviation:

patient(s) n°
Deviation:

patient(s) n°
Deviation:

patient(s) n°
Deviation:

Are all protocol deviations documented? ☐yes ☐no ☐NA

Are there systematic errors to be reported? ☐yes ☐no ☐NA

If yes, please add which errors and what action was taken

Additional Comments

CRF (FOR ROUTINE AND CLOSE-OUT MONITORING VISITS ONLY)

Was SDV performed during this visit? ☐yes ☐no ☐NA

If not, specify:
…………………………………………………………………

Had the Quality Manager performed 100% SDV prior to this visit?	☐yes ☐no ☐NA
If not, specify: …………………………………………………………………	
If yes, CRF fully checked at this visit:	
From patient n° until patient n° ; from patient n° until patient n° ; from patient n° until patient n°	
Were CRF legible, accurate and complete?	☐yes ☐no ☐NA
Indicate estimated error rate: %	
Are there any corrections pending?	☐yes ☐no ☐NA
If yes, specify:	
For Close-Out Monitoring Visit Only:	
Have all outstanding queries and issues been resolved?	☐yes ☐no ☐NA
Have copies of the final CRFs and data queries been filed?	☐yes ☐no ☐NA
Were other forms legible, accurate and complete?	
Patient Identification List	☐yes ☐no ☐NA
Study Specimen Log	☐yes ☐no ☐NA
Additional Comments	

STUDY TESTS AND MATERIALS	
Where are the study diagnostic tests kept?	
Who is responsible for the storage and distribution of the tests?	
Are transportation, storage and dispensing conditions of tests appropriate?	☐yes ☐no ☐NA
Is the amount of tests left still sufficient?	☐yes ☐no ☐NA
Was any tests expired?	☐yes ☐no ☐NA
If yes, please indicate which test and action taken:	
Are the accountability logs consistent with the number of recruited patients and the remaining stock?	☐yes ☐no ☐NA
Is the history of shipment reception of tests accurately documented?	☐yes ☐no ☐NA
Is there temperature control log in the facility?	☐yes ☐no ☐NA
Did the temperature exceed the allowed temperatures?	☐yes ☐no ☐NA
if yes, what action was taken?	
Is there any concern about the quality of the study diagnostic tests?	☐yes ☐ no ☐ NA
if yes, please indicate which test and action taken:	
Additional Comments	

LABORATORY
EXPERT SUPERVISION
WHEN WAS THE LAB LAST VISITED BY A NIDIAG LAB EXPERT? ……………..

WAS THE VISIT REPORT AVAILABLE? ……………………………………….<br>WERE THE EXPERT RECOMMENDATIONS IMPLEMENTED?<br>IF NOT, EXPLAIN WHY AND DESCRIBE FURTHER PLANS :………………………………	
FACILITY	
Is the design and location of the laboratory facility appropriate to the study needs?	☐yes ☐no ☐NA
Does the laboratory allow for separation of areas meant for study specimens reception, analysis and storage?	☐yes ☐no ☐NA
Does the laboratory have back up facility in the event of power failure?	☐yes ☐no ☐NA
Does the laboratory ensure safety to the work and the worker?	☐yes ☐no ☐NA
Is access to data on paper, magnetic and other storage device restricted only to authorized personnel?	☐yes ☐no ☐NA
Additional Comments	
EQUIPMENT AND MATERIAL	
Is the laboratory furnished with all necessary and appropriate items of equipment?	☐yes ☐no ☐NA
Are the laboratory personnel trained in the operation of the equipment?	☐yes ☐no ☐NA
Are the equipments cleaned and maintained well by the personnel?	☐yes ☐no ☐NA
Are the equipments calibrated periodically and records of calibration documented?	☐yes ☐no ☐NA
Are systems in place to detect faults in the equipment? e.g. temperature log for freezers, refrigerators and incubators, biological indicators for autoclave	☐yes ☐no ☐NA
Are the materials / reagents used fit for purpose?	☐yes ☐no ☐NA
Are the reagents labelled appropriately with the identity, concentration, date of preparation, date of opening and expiry, storage conditions?	☐yes ☐no ☐NA
Are the reagents and materials stored under appropriate conditions?	☐yes ☐no ☐NA
Additional Comments	
STUDY SPECIMENS	
Are study specimens stored in a safe manner (under lock and key custody)?	☐yes ☐no ☐NA
Are the conditions for storage of samples sufficient?	☐yes ☐no ☐NA
Did the storage temperature exceed the allowed temperatures?	☐yes ☐no ☐NA
if yes, which samples were affected, and what action was taken?	
Are specimens received in the laboratory coded with a unique number to allow tracking of the sample from receipt to reporting?	☐yes ☐no ☐NA
Are the SOPs followed in the performance of the sample analysis?	☐yes ☐no ☐NA
Are analytical procedures validated and performance checked periodically with suitable controls?	☐yes ☐no ☐NA
Are deviations in the performance of analytical procedures documented and reported to the lab director?	☐yes ☐no ☐NA

Are reference ranges available for each analytical procedure performed?	☐yes ☐no ☐NA
Does specimen transport follow proper organization, packaging, shipping to ensure integrity, timely and safe transfer?	☐yes ☐no ☐NA
Additional Comments	

DATA	
Are data generated recorded directly, promptly and accurately?	☐yes ☐no ☐NA
Are data recorded in a lab notebook before being entered in the database?	☐yes ☐no ☐NA
If yes, please specify (a) if this concern a part of data or all data, (b) who enters the data into the study database and (c) who verifies the accuracy of transcription:	
Is there a system for back up of electronic data as a routine?	☐yes ☐no ☐NA
Is access to the data restricted only to authorized personnel?	☐yes ☐no ☐NA
Additional Comments	

QUALITY CONTROL	
Is the internal quality control (IQC) performed on a daily basis?	☐yes ☐no ☐NA
Has the IQC procedure been reviewed by all lab technicians?	☐yes ☐no ☐NA
Is an external quality control (EQC) performed on a regular basis?	☐yes ☐no ☐NA
If yes, are records of the EQC participation archived?	☐yes ☐no ☐NA
Additional Comments	

**COMMENTS**

Monitor :	Reviewer :
Print name :	Print name :
Signature :	Signature :
Date : dd mmm yyyy	Date : dd mmm yyyy

PENDINGS – FOLLOW-UP ACTIONS REQUIRED			
Pending (Please repeat all lines until date done is completed.)	Responsible	Date Due	Date done
Staff			
Master Files			
Recruitment			
Protocol, ICF, CRFs			
Study Tests and materials			
Laboratory			
General			

Monitor :	Reviewer :
Print name :	Print name :
Signature :	Signature :
Date :	Date :
dd mmm yyyy	dd mmm yyyy

	**SOP Title:** Internal Quality Control Activities
	Project/study: This SOP applies to all NIDIAG studies.

1. Scope and application

All NIDIAG studies have to be conducted in compliance with the NIDIAG Ethical Charter, the national and international applicable regulations, the WHO Good Clinical Laboratory Practices (GCLP) and the ICH and WHO Good Clinical Practices (GCP) Guidelines. The GCP/GCLP monitoring system to be implemented throughout NIDIAG includes 2 complementary components: 1) An internal quality control (IQC) component and 2) an external quality control (EQC) component which includes lab monitoring. The IQC component involves site Quality Managers (QM) who are responsible for performing regular quality checks at the study site.
Since the GCP and GCLP supervision may entail different skills and expertise, the site country coordinating investigator can decide to allocate the task of QM to one or two persons within his/her team, depending on available skills and expertise.
This procedure describes the responsibilities of the site QM and how his/her work articulates with the work of the external monitoring component (SOP-WP6-QUAL-02) and of lab monitoring.
This SOP doesn't apply to DRC.

2. Responsibilities

Function	Activities
Site Quality manager (QM)	- Ensure the study site team is aware of all aspects related to GCP/GCLP compliance<br>- Ensure the study site team is trained on the study protocol and relevant NIDIAG SOPs<br>- Conduct regular quality control checks for all study-related activities<br>- Document the quality control checks in the QM Report and discuss the GCP/GCLP deviations identified with the PI and the study team<br>- Report any major deviation –in addition to the site PI- to the WP6 leader, who will coordinate communication with the External Monitor and/or the lab monitor, as needed<br>- Support the PI, the External Monitor and the lab supervisor in the organization and conduct of monitoring visits (SOP-WP6-QUAL-02).<br>- Attend monitoring visits<br>- Assist Site Principal Investigator in the implementation of corrective actions following IQC and EQC
External Monitor<br>Lab Monitor	- Provide advice and support to QM on the performance of quality control checks between monitoring visits<br>- Review any major deviation reported by the QM and if required, discuss these deviations with WP6 leader
Site Principal Investigator (PI)	- Ensure that the QM has full access to the study documents and facilities<br>- Ensure that the QM is allocated enough time to carry out the requested activities, without any detriment for his/her other routine tasks<br>- Ensure that the QM receives any new key-information relevant to the study status and conduct Implement corrective actions to address the deviations identified by the QM
Country Coordinating Investigator	- Assign the task of QM to one or two member(s)

WP6 leader or his/her delegate	▪ Be the link between the Trial Management Group and the QM<br>▪ Provide guidance to QM for the planning of QC activities at each study site<br>▪ Review the QM reports and timely send a copy to the Country Coordinating Investigator, the WP4 representative and the concerned members of the Trial Management Group<br>▪ Be in touch with the QM and PI regularly, to advise them on problems concerning the quality and/or the frequency of QC

3. Procedures

3.1 Training of the study site team

All staff involved in NIDIAG should receive training on the study protocol, the SOPs that are relevant to his/her work, Good Clinical Practice (GCP) and Good Clinical Laboratory Practice (GCLP). The QM will, together with the site PI and the external monitor/lab monitor, provide such training for all staff who have not attended NIDIAG GCP/GCLP workshop, before recruitment starts. The model training material is available through the NIDIAG website (www.nidiag.org) or through WP6 leader.

The QM together with the PI will provide training for new staff in case of handovers, or retraining to staff in need of it.

3.2 Description of Internal Quality Control Activities

3.2.1. Verifying Informed Consent Forms (ICF)

Verify compliance with NIDIAG SOP-WP6-DOC-01. In particular, the QM should try to be present during the informed consent discussion for the first 10 patients enrolled in the study and then after, at least once every 25 patients provided of course the individual patients agree. He/she should verify that the process of obtaining Informed Consent complies with the principles of the Helsinki Declaration, WHO and ICH Guidelines for Good Clinical Practice.

Verify that **all** patients included in the study have provided written informed consent:

- Verify that names, date and time of ICF signature are appropriate.
- Check whether or not a legal representative /an independent witness was required for each one of the cases according to GCP and SOP-WP6-DOC-01
- For legally incompetent subjects, check whether assent was required and if yes, if it was obtained
- For patients included under an emergency situation, check that consent was initially obtained from the patient's relatives and then reiterated by the patient himself/herself
- Verify that consent has been obtained prior to any study related procedure
- Verify with the investigator that each participant has been given a signed copy of the ICF
- Verify that all consent forms are securely stored in the Site Investigator's File

Document the ICF review in the Quality Manager report.

3.2.2. Reviewing Case Report Forms (CRF) and Lab Data Collection Forms

Check that the "Patient Identification list" is completed, accurate and coherent with the center's register (check patient's name, initials, date of birth/age, registration number, date of admission).

Check that patients' numbers have been allocated in accordance with SOP-WP6-DOC-02.

Note down the number of screened patients and the number of enrolled patients in the QM report.

For all patients, check that CRF have been completed in accordance with SOP-WP6-DATA-01. Check that CRF entries are legible and that only authorized staff has filled in the CRFs and laboratory data collection forms.

Check all patient's CRF against source documents, i.e. the patient medical file, the laboratory notebook(s), the study doctor's notes, the study center's register and any other source document available at each site.

In particular check the following:

- The results of the reference tests performed on site are consistent throughout the CRF, the lab notebook(s) and other lab report forms
- The results of the reference tests performed in reference labs are consistent throughout the CRF and the reference lab report forms
- The results of the index tests are consistent throughout the CRF, the lab notebook(s) and any other source document available at each site (if applicable).
- The medication prescribed are consistent throughout the CRF, the patient medical file and the study doctor's notes
- The timing of clinical examinations, sample collection and laboratory investigations are consistent throughout all documents

Regarding Index test results the source is the lab register / lab notebook in Cambodia, Nepal and Sudan.

In case of discrepancies between the CRF and the source documents, report them in the QM report. Remind the PI to ensure missing data is completed and that incorrect entries are corrected by the responsible site investigator.

Check that the format of corrections made in CRF and other data collection forms complies with SOP-WP6-DATA-01.

3.2.3. Reviewing the Site Investigator File/Lab File

The Site Investigator File includes all essential documents to be collected and filed by the study team throughout the NIDIAG project.

The lab file includes all essential lab documents to be collected and filed by the lab team.

Support the PI and the lab manager to check that the SIF and the lab file are complete and kept up-to-date in accordance with SOP-WP6-DOC-03.

Verify that the site investigator sends copies of relevant documents to the Country Coordinating Investigator.

3.2.4. Biological Samples

All laboratory activities should be verified by a QM with appropriate laboratory skills.

Some of the samples collected during NIDIAG are processed and analyzed on site. Some others are sent to reference laboratories (in the country or abroad), while others are stored in view of future research.

Check that the sample flow is running smoothly and in accordance with the corresponding SOP.

Verify that the handling, processing, management and disposal of hazardous specimens meet the necessary safety precautions.

Check that the "Study Specimen Log" or equivalent document(s) is completed, accurate and coherent with the center's register and the CRF (check patient's number, date of specimen collection, type of specimen collected, whether the specimen was shipped or not, etc…).

Check that the samples are correctly numbered and labeled in accordance with SOP-WP6-DOC-02. Verify that samples are not labeled with patient's personal identifiers (names, address, and telephone number).

Check that samples are appropriately stored, and in particular that:
- access is restricted to authorized staff
- study specimens are stored separately from specimens collected for other lab routine activities
- the storage temperature is appropriate according to relevant SOPs and checked regularly by responsible staff

Check that analyses performed on site are conducted in accordance with the protocol and applicable NIDIAG SOPs. Verify that:
- analyses are performed only by appropriately trained staff
- low-quality samples (i.e. insufficient volume collected, contaminated samples, etc…) are rejected upon receipt
- deviations from SOPs are documented in the lab notebook(s)
- analyses yielding abnormal test results or out-of-range /invalid controls are repeated
- results are validated by lab director before being transmitted to the site PI

- blinding is respected for index tests
- results are transmitted in a timely fashion to the site PI

Check that the shipment of samples to reference laboratories (inside or outside the country) is appropriate: verify that the timing of shipment complies with the applicable NIDIAG SOP, that the temperature during transport is adequate and that the necessary safety precautions are taken for hazardous samples. Also, verify that the date and time of specimen shipment, the duration of transport and the date and time of specimen receipt at the reference lab is documented in the “Study Specimen Log”.

The laboratory activities throughout the digestive study should actively be monitored by a QM who should regularly re-read a certain amount of around 5-10% of all processed samples. 10% of all stool samples should be preserved in SAF solution to allow for later quality control. Specifically, the QM for the digestive study could carry out the following examinations:

- on SAF-preserved samples: Mini-FLOTAC, Formalin-ether concentration technique (SAF-preserved samples can be analysed within several weeks after sampling).
- on acid-fast stains: quality control by re-reading 10% of all samples (acid-fast stained slides can be analysed within several months after sampling).
- If feasible, a photo should be taken of RDT results at the time when the result is read and documented (RDT-specific, e.g. after 15 minutes). These photos and the recorded results should be checked for consistency by the QM.
- For most other laboratory tests (direct faecal smear, Kato-Katz, Baermann technique, Koga agar plate), no preservation is performed but the ‘fresh’ samples from the same day should be re-read by the QM.
- Bacteriological stool cultures are not performed in the field laboratory, but in specialized institutions (e.g. the Institut Pasteur in Côte d’Ivoire, the Institut National de Recherche en Santé Publique in Mali). The QM should check whether the institutions’ standard quality assurance guidelines are followed throughout the processing of the NIDIAG digestive study samples.

3.2.5. Index RDTs storage and accountability

Verify that the index RDTs evaluated in NIDIAG studies are stored in appropriate manner. In particular, check that:

- Access to RDTs stored is restricted to authorized staff
- The storage temperature is checked regularly by responsible staff. RDT kits should not be used if they are damaged.

Also verify that:

- RDTs are used before the expiry dates
- RDTs are used only for patients included in the study
- An adequate system for RDT accountability is in place

3.3 Frequency of Quality Control activities

Quality control activities should be performed regularly throughout recruitment, on at least every 25 patients enrolled in the study (see Table 1).

Quality control activities should be performed prior to routine monitoring visits. The PI is responsible for ensuring that the study team implements corrective actions formulated by the QM and address deviations BEFORE the external monitor visit.

It is anticipated that internal quality control activities, if conducted regularly as indicated below, should take approximately 3 days to complete.

Table 1: Suggested frequency of internal quality control activities

Internal QC activity	Frequency of QC activity
Verification of ICF	ALL patients, every 10 patients enrolled
Verification of Patient ID list	ALL patients, at least every 25 patients enrolled

Review of CRF	ALL patients, at least every 25 patients enrolled
Review of SIF, IMF and LF	At least once in a month and every time there is major change (i.e. change in staff, amendment of study protocol, receipt of new RDTs batches)
Verification of biological samples	ALL patients, at least every 25 patients
Verification of index test storage and accountability	At least once in a month and upon receipt of a new RDT package

3.4 Reporting

Each time quality control activities are conducted, a QM report should be completed and shared and discussed with the site PI and the WP6 leader and/or his delegates.

All critical and major deviations should be immediately reported (in addition to the site PI) to the WP6 leader or delegate, who will circulate them to the relevant persons in the TMG, to help formulating corrective actions.

QM are encouraged to group internal QC activities over a period of 2-3 days and to adapt the frequency of the quality checks based on recruitment pace and problems encountered. As long as possible, QM should submit complete reports to the WP6 representative, although some activities may not be conducted with the same frequency.

4. Definitions and Abbreviations

4.1 Definitions

Laboratory File (LF): The Laboratory File includes all essential documents related to the laboratory work at the site level. There is one Laboratory File per study site. The laboratory personnel is responsible for keeping it updated and for ensuring it is stored adequately.

Site Investigator File (SIF): The Site File includes all essential documents and forms related to the conduct of the study at the site level. There is one Site File per study site. The site investigators are responsible for keeping it updated and for ensuring it is stored adequately.

Country Investigator's Master File (IMF): The Investigator's Master File includes all essential documents related to the conduct of the study at the country level. There is one Investigator Master File per country. The Country Coordinating Investigator is responsible for keeping it updated and for ensuring it is stored adequately.

Critical deviation: Conditions, practices or processes that adversely affect the rights, safety or wellbeing of the subjects and/or the quality and integrity of data. These must be documented and reflected in the yearly and final study reports. Corrective actions must be immediately implemented.

Major deviation: Conditions, practices or processes that might adversely affect the rights, safety or wellbeing of the subjects and/or the quality and integrity of data. These must be documented and, if needed, reflected in the yearly and final study reports. Corrective actions must be implemented.

Quality Assurance (QA): All those planned and systematic actions that are established to ensure that the study is performed and the data are generated, documented (recorded), and reported in compliance with Good Clinical Practice (GCP) and the applicable regulatory requirement(s).

Quality Control (QC): The operational techniques and activities undertaken within the quality assurance system to verify that the requirements for quality of the trial-related activities have been fulfilled.

Source Document: These are original documents. They consist of data relevant to the current research such as medical records, administrative files, laboratory reports, consultation reports, pharmacy dispensation registers, etc...

4.2 Abbreviations

CRF: Case Report Form

EQC: External Quality Control

GCLP: Good Clinical Laboratory Practice

Revision	
V2	Compared to the first version of this SOP (dated 6 Aug 2012), the WP6 now becomes the central contact for the quality manager, and the external lab monitor is regularly mentioned when applicable.<br>Also, the extent and frequency of quality control activities was further described
V3	Project/study concerned was changed from “Fever study” to “All studies” since Neuro and Digestive syndromes are also concerned by this SOP.
V4	Additional digestive study-specific QM responsibilities mentioned in “3.2.4 Biological Samples”.

GCP: Good Clinical Practice

ICF: Informed Consent Form

IMF: Country Investigator File

IQC: Internal Quality Control

LF: Laboratory File

PI: Principal Investigator

RDT: Rapid Diagnostic Test

QM: Quality Manager

SIF: Site Investigator File

5. Records and archives

Appendices & Forms for completion	
**Number**	**Title**
SOP-WP6-QUAL03-annex1	Quality Manager Report Template

6. Document History

Name and function	Date	Signature
Author		
Emilie Alirol	01/02/2013	
Reviewed by		
Raffaella Ravinetto	01/02/2013	
Rosanna Peeling	01/02/2013	
Approved by		
Ninon Horié	07/07/2014	

QUALITY MANAGER REPORT N°		
Syndrome:		
Study protocol n°:		
Study Site :		
Country :		
Site PI:		
Country Coordinating Investigator:		
Quality Manager :		
External Monitor :		
Date of Report:	(dd/mm/yyyy)	
Number of patients (at time of reporting):	Screened:	Included:
Patients checked this time	From patient n°	Until patient n°
In case there are two QM at this site, please describe how the tasks are shared:		

**INFORMED CONSENT**	
The Informed Consent process complies with SOP WP6 DOC 01	☐yes ☐no ☐NA
All patients included in the study have provided written informed consent	☐yes ☐no ☐NA
Consent has been obtained prior to the conduct of study-related procedures	☐yes ☐no ☐NA
The site PI (or his delegate) has signed and dated the consent form	☐yes ☐no ☐NA
When appropriate, consent was obtained from legally-authorized representatives and/or impartial witness	☐yes ☐no ☐NA
When appropriate, assent was obtained from the child	☐yes ☐no ☐NA
For patients admitted under emergency situations, consent was obtained both from the relatives (initial) and from the patient himself/herself (reiteration)	☐yes ☐no ☐NA
All patients included in the study have received a copy of the ICF	☐yes ☐no ☐NA
The ICF are securely stored in the site investigator file	☐yes ☐no ☐NA
If deviations in the Informed Consent process are noted, please list them below	
Patient(s) n°	Deviation:
Patient(s) n°	Deviation:
Patient(s) n°	Deviation:
Patient(s) n°	Deviation:
Patient(s) n°	Deviation:
Additional Comments and corrective actions proposed (including retraining of staff if appropriate):	

CASE REPORT FORMS AND LABORATORY DATA COLLECTION TOOLS	
The CRFs have been completed in accordance with SOP-WP6-DATA-01	☐yes ☐no ☐NA
CRFs and Laboratory data collection tools are legible, accurate and complete	☐yes ☐no ☐NA
CRFs and Laboratory data collection tools have been completed by authorized staff only	☐yes ☐no ☐NA
CRFs and Laboratory data collection tools are consistent with the source documents	☐yes ☐no ☐NA
Corrections to CRF and Laboratory data collection tools comply with SOP-WP6-DATA-01	☐yes ☐no ☐NA
CRFs and Laboratory data collection tools are stored in appropriate place	☐yes ☐no ☐NA
If deviations in the Data Collection process are noted, please list them below	
Patient(s) n°	Deviation:
Patient(s) n°	Deviation:
Patient(s) n°	Deviation:
Patient(s) n°	Deviation:
Patient(s) n°	Deviation:
Additional Comments and corrective actions proposed (including retraining of staff if appropriate):	

BIOLOGICAL SPECIMENS	
The collection, processing, handling and shipment of biological specimens are in accordance with applicable NIDIAG SOPs	☐yes ☐no ☐NA
The specimen collection logs is completed and kept up-to-date	☐yes ☐no ☐NA
The samples are labeled with a unique specimen number in accordance with SOP-WP6-DOC-02	☐yes ☐no ☐NA
The process ensures patient's confidentiality	☐yes ☐no ☐NA
Study specimens are stored in a secure manner (locked + restricted access)	☐yes ☐no ☐NA
Storage temperature is adequate and checked regularly	☐yes ☐no ☐NA
All analyses are conducted in accordance with applicable NIDIAG SOPs	☐yes ☐no ☐NA
Results of analyses are promptly recorded and transmitted to the site PI	☐yes ☐no ☐NA
Analyses yielding abnormal test results or out-of-range controls are documented and repeated	☐yes ☐no ☐NA
Hazardous specimens are handled and disposed appropriately	☐yes ☐no ☐NA
Study specimens are shipped in accordance with NIDIAG SOPs and with national and internal regulations	☐yes ☐no ☐NA
If deviations in the specimen management process are noted (missed tube, haemolysis, inappropriate specimen volume, thawing), please list them below	
Patient(s) n°	Deviation:

Patient(s) n°	Deviation:
Patient(s) n°	Deviation:
Patient(s) n°	Deviation:
Patient(s) n°	Deviation:
Additional Comments and corrective actions proposed (including retraining of staff if appropriate):	

**RAPID DIAGNOSTIC TESTS**	
The RDTs are securely stored (restricted access + lock space)	☐yes ☐no ☐NA
The storage temperature is in accordance with manufacturer's recommendations	☐yes ☐no ☐NA
The RDTs are used only for the patients included in NIDIAG study	☐yes ☐no ☐NA
The RDTs are used (and will be used) before the expiry date	☐yes ☐no ☐NA
The accountability system for RDTs is correct and kept up-to-date	☐yes ☐no ☐NA
Additional Comments and corrective actions proposed (including retraining of staff if appropriate):	
**Any other relevant information**	

Report written by the QM(s):

Name (s):

Signature and date:

Report approved by the site PI:

Name:

Signature(s) and date:

Report sent to the WP6 representative (………….. …………………….): on ……. [DATE]

NA: Not Assessed

	**SOP Title:** Laboratory supervision visit
	Project/study: NIDIAG: this SOP applies to all NIDIAG clinical studies (WP2).

1. Scope and application

All NIDIAG studies have to be conducted in compliance with the NIDIAG Ethical Charter, the national and international applicable regulations, the WHO Good Clinical Laboratory Practices (GCLP) and the ICH and WHO Good Clinical Practices (GCP) Guidelines. The GCP/GCLP monitoring system to be implemented throughout NIDIAG includes 2 complementary components: 1) An internal quality control (IQC) component and 2) an external quality control (EQC) component.
This procedure describes how laboratory supervision visits should be carried out, documented and followed up, in compliance with the GCLP principles.

2. Responsibilities

Function	Activities
Laboratory supervisor	- Verifies that the trial is conducted in compliance with the GCLP requirements, with the study protocol and amendments, and with the NIDIAG SOPs<br>- Reports any deviation in the laboratory visit report<br>- Sends the laboratory visit report at a minimum to the WP2 leader, the country coordinator investigator, the site principal investigator and the WP6 monitoring coordinator*<br>- Address specific questions in-between two visits
External Monitor	- Reports any deviations regarding the laboratory in the monitoring visit report
Quality manager (QM) or equivalent function	▪ Supports the laboratory monitor in the fulfillment of his/her tasks<br>▪ Attends the laboratory monitoring visits
Site Principal Investigator (PI)	▪ Supports the laboratory monitor in the fulfillment of his/her tasks<br>▪ Attends the laboratory monitoring visits and ensures all key-study staff are present<br>▪ Ensures that the laboratory monitor has full access to the study documents and facilities<br>▪ Ensures that the corrective actions listed in the laboratory visit report and/or follow-up letter are implemented
Country coordinating investigator	▪ Ensures that the laboratory monitor receives any new key-information relevant to the study status and conduct<br>▪ Verifies that the corrective actions listed in the laboratory visit report and/or follow-up letter are implemented
WP6 monitoring coordinator*	▪ Makes sure that the laboratory monitor receives the reports from the external monitor

*The WP6 monitoring coordinator is Raffaella Ravinetto for Neurological syndrome, Ninon Horié for Fever syndrome and Peiling Yap for Digestive syndrome.

3. Laboratory supervision visits to be carried out

3.1 Laboratory supervision visit planning

The WP6 coordinator, in consultation with the Country Coordinating Investigator and the WP2 leader, will determine the appropriate calendar for laboratory monitoring visits for each clinical study. The laboratory supervision visits take place at the field site itself and at the referral labs and central administration, if applicable.

Prior to visit, the laboratory supervisor should:

- Review the study protocol, CRF, study SOPs and previous reports from external monitor/laboratory supervisor, as well as any other key documents provided by the country coordinating Investigator or by the WP2 leader.
- Inform the country coordinating investigator and the site PI at least 1 month ahead of the laboratory supervision visit, and ask them to ensure that all key-staff are present for the visit.
- Send a provisional visit agenda to the country coordinating investigator.

3.2 Laboratory supervision visits to be carried out

3.2.1 Laboratory assessment visit

The laboratory assessment is carried out before start of recruitment. It is carried out by the WP2 leader or a qualified delegated person, together with the country coordinating investigator, and ideally with either the laboratory supervisor (if already identified) or a delegate with knowledge on routine laboratory procedures and GCLP aspects. A report with the outcomes and conclusions from the assessment is prepared and distributed among the study coordination team.

During the laboratory assessment visit, the following aspects are checked (checklists can be used as an aid):

- The general conditions of the laboratory (facilities, electricity supply, which kind of laboratory testing is performed, type and quality of reagents and equipment, stock system, laboratory recording, etc.)
- The availability and knowledge of the lab staff (sufficient dedicated number of lab staff available, lab staff competent or not, back-up available or not)
- The quality management system for the lab (quality controls available, participation in EQA, documentation, etc.)

The following decisions and actions should be taken <u>before</u> study initiation visit:

1. Decide which (index) testing should be performed on site and which should be performed in referral labs and make sure that all (index) tests are validated before use.
2. Develop an analytical plan or alternatively, make sure that this type of information is available through other means (SOPs, lab file), *e.g.* sample and result flow chart, SOP on handling, storing and management of study samples; documentation of normal ranges, SOP on data management, …
3. Make sure a high quality Case Report File (CRF) is developed in collaboration with WP6 data manager and statisticians.
4. Make sure that all necessary equipment, test kits and reagents for all laboratory tests to be performed are available.
5. Make sure SOPs are available for all laboratory procedures and related activities (*e.g.* quality assurance, data management). If needed, prepare site specific SOPs.
6. Make sure an efficient ordering system is in place for a timely and routinely supply of test kits and reagents (either locally or centrally at the study coordinator site[1])

If any of the above requirements are not fulfilled, the study should <u>NOT</u> be initiated.

3.2.2 Study initiation visit

The study initiation visit is performed after receipt of all study materials, after approval of the EC, and prior to the start of recruitment. Ideally the visit is carried out together with the external monitor and/or WP2 leader. At least the following activities must be carried out:

- *GCLP training*[2]:

In discussion with the WP6 monitoring coordinator and the monitor of the site, a formal GCLP training is given for all staff who have not yet attended NIDIAG GCP/GCLP workshop, by either the external monitor and/or the laboratory supervisor in collaboration with the QM, before start of recruitment.

- *Protocol review*: the laboratory aspects of the protocol are reviewed with all the study staff, the analytical plan and related documents are discussed (*e.g.* sample and result flow chart).
- *Laboratory training:* If required training is provided on all the SOPs related to laboratory activities (laboratory procedures, handling/storing/management of study samples, source data and data management, stock system, quality assurance, etc.)
- *CRF training:* Training on the laboratory part of the Case Report File (CRF) is provided.
- *Laboratory File*: The laboratory file is verified for completeness (ref. SOP No WP6-DOC-03 on Management of Study Documents).
- *Laboratory facility*: the laboratory supervisor should confirm that the lab is adequate for the conduct of study activities (storage space for lab materials and source documents, temperature monitoring).
- *Piloting*: If all findings are satisfactory, a piloting study (with 10-20 patients) can be conducted at the end of the initiation visit, under supervision of the laboratory supervisor and/or the external monitor/WP2 leader. During this piloting study, all activities are carried out as if real patients were recruited. Corrective actions are applied where needed.
- *Start of recruitment:* If the piloting phase was satisfactory, the recruitment can start.

A laboratory supervision report of the initiation visit and the piloting phase is prepared, documenting all the above, which is sent to the WP2 leader/delegate, the country coordinating investigator/delegate, and to the site Principal Investigator for follow-up on findings and corrective actions.

Exceptions: the WP2 leader may decide to skip the study initiation visit, if all the points have been carried out and documented on previous visits, and corrective actions have been implemented.

3.2.3 Routine laboratory supervision visits

Routine laboratory supervision visits take place as planned with the WP6 monitoring coordinator and the country coordinating investigator (ideally every 3 months at the beginning of the study, up to every 6-8 months at a further stage of the study) These visits can coincide with the visits of the GCP monitor and/or the WP2 leader.

At least the following aspects should be checked:

- *Follow-up of previous visit*: Follow-up of any pendings from the previous visit/s.
- *Lab facility:* Check if there were any problems concerning the lab facility during the course of the study. These should be recorded in a notebook (*e.g*. problems with electricity)
- *Stock management and ordering of laboratory products*
  - Check that the laboratory products are stored in a suitable environment (dry, cool, protected from flooding/rodents, at the correct temperature - air conditioning, fridge - etc.)
  - Check that proper temperature monitoring is in place for storage room, fridges, freezers, incubators and laboratory. (Refer to SOP-WP6-QUAL-06 How to install and use a "Min/Max" thermometer). Temperature monitoring forms should be filed in the laboratory file.
  - Check that a good stock management system is in place for the laboratory products to be used for the Nidiag study: stock cards and monthly inventory of stock (Refer to SOP-WP6-QUAL-07-V01-24Sep2012 - Stock management). Monthly inventories should be stored in laboratory file.
  - Check that the "First in, first out" principle is used: use first the products that will expire first.
  - Check that there is a track record of what has been ordered (information is available depending on where ordering takes place: either at study coordination level or at country coordination level/field site).
    - Which items have been ordered, what was the quantity, at what time was the order done
    - Lot number and expiry dates of ordered items
    - If shipment of laboratory items: when did the shipment take place, proof of reception of the items, data on temperature monitoring (and if needed humidity monitoring) during shipment of items via a temperature logger
  - If index tests are ordered centrally (at study coordination level):
    - Check that a track record is available of what has been ordered (see previous point)
    - Check that 1 retention kit per lot of index tests is kept at the study coordination level
    - Check that version control of instructions for use (IFU) is being applied:
      - which lot has which IFU version number and date of issue
      - for every new version of IFU, adapt the related SOP if needed
- *Biological specimens*
  - Check that all biological specimens are handled as described in the applicable SOPs (*e.g.* biosafety). Check if the sample flow is running smoothly.
  - Check that all biological specimens are correctly labeled (Refer to SOP-WP6-DOC-02-V2.2-21December2012 – Numbering system).
  - Check that the required volume of samples is collected as described in the applicable SOPs (e.g. *SOP-WP2-LAB-44 – blood collection)*, and that this information is correctly recorded (in CRF / specific sampling forms).
  - Check that the required volume of left-over samples are collected in proper containers (*e.g.* 2 ml cryo-vials) according to applicable SOPs and that the stored

samples are correctly recorded in the study specimen log stored in the laboratory file.

- Check that the Study Specimen Log or equivalent document(s) is completed, accurate and coherent with the center's register and the CRF (check patient's number, date of specimen collection, type of specimen collected, whether the specimen was shipped or not, etc…).
- Storage of biological specimens
  - Check that all biological specimens are properly stored until transport or until the end of the study (*e.g.* -70°C for left-over patient samples, incubator for blood cultures)
  - Check that a freezer inventory is available for the -70°C freezer, together with daily temperature monitoring records (laboratory file)
  - Check that the bacterial isolates are properly stored (Refer to SOP-WP6-LAB-01) until transport or until the end of the study.
- Transport of biological specimens
  - Check that a packing list is filled out for each transport, and a copy of the packing list is stored in the laboratory file.
  - Check that the transport of biological specimens within the country (*e.g.* from study site to referral lab) or between countries (*e.g.* from study site to ITM Antwerp) is done according to applicable SOPs: triple packaging (biosafety), correct temperature (*e.g.* ice packs, temperature logger)
  - Check that the date and time of specimen shipment, the duration of transport and the date and time of specimen receipt at the reference lab is documented in the Study Specimen Log.

- *Laboratory testing*
  - Check that laboratory request forms are in place (which samples to be taken, which tests to be done, etc.) and are according to study protocol and applicable SOPs.
  - Check that the correct materials and reagents are used for the laboratory testing. None of the products should be expired.
  - Check that country specific normal values of all relevant laboratory testing are available in the laboratory file and the Site Investigator file.
  - Check that the tests are interpreted according to applicable SOPs.
  - Check that test results are correctly recorded in laboratory notebooks and/or CRF.
  - Check that all source data (test results) are correctly stored in lockable cabinets and can be traced for data source verification by the external monitor.
  - Check that the results are reported to the PI in a correct and timely manner.

- *Index testing*
  - Check that the index tests are correctly handled and stored (Refer to SOP-WP6-QUAL-05 Handling and storage of RDTs)
  - Check that none of the index tests in use are expired.
  - Check that the index tests are only used for patients that are recruited in the study.
  - Check that the index test log is properly filled.

- Check that the index tests are performed according to applicable SOPs (correct volume of sample, correct buffer, correct number of buffer drops, correct reading time).
    - Check that micropipettes are used instead of the blood transfer devices included in the RDT kits.
    - Check that the procedures for repeat testing and documentation of results (desiccant color change, presence of control line, presence of background, intensity of test lines) are correctly applied.
    - Check that results are correctly interpreted according to applicable SOPs.
    - Check that pictures are taken of the index tests, and if this data is securely saved on a computer (with back-ups).
    - Check that all source data (index test results) and CRFs are correctly stored (*e.g.* separately from the rest of the CRF if blinding of index tests results is required) and that they can be traced for data verification by the external monitor.

- *Quality control:*
    - Check that internal quality controls are used according to applicable SOPs:
        - Daily/weekly internal controls for biochemistry, hematology, serology etc.
        - ATCC strains and quality parameters for bacteriology (% contamination, % clinically significant organisms (CSO), volume of blood collected in blood culture bottles)
        - Second reading for specific tests (refer to specific SOPs)
    - Perform re-reading of positive microscopy slides (*e.g.* malaria slides, Gram stainings) and a percentage of negative microscopy slides (blinded of original results).
    - Check the report of the external quality assessment if the laboratory is participating in an EQA program (*e.g.* EQA for bacterial diagnostics for Nidiag sites in the first trimester of 2014).
    - Check the report of the quality audit if performed (to be agreed within TMG).
    - Check that all records of the quality controls are filed in the lab file.

- *SOPs*
    - Check that every test and every equipment has an up-to-date SOP.
    - Check that all versions of the SOPs are filed in the lab file.
    - Check that only the latest versions of the SOPs are used in the lab.
    - Check that the SOPs are reviewed and revised regularly.

- *CRF*
    - If applicable, check if the laboratory technician that fills the CRF is trained in Good Documenting Practice (GDP).
    - Check that the laboratory part of the CRFs are properly filled in according to GDP.
    - Check that the laboratory CRFs are securely stored until they are sent back to the PI.
    - For fever study: Check that the index testing part of the CRF is separately stored from the rest of the CRF to be sent directly to the data entry clerk, without passing by the PI (blinding of index test results).
    - Data source verification of the CRFs is done by the external monitor.

- *Equipment*

- Equipment inventory
  - Check that an equipment inventory is in place for the equipment in the lab.
- Maintenance plan
  - Check that a maintenance plan is in place for the equipment or if in the SOPs of the equipment it is mentioned how the equipment should be maintained and at which time points this maintenance should be done.
- Service and repair
  - Check that a logbook is in place to record if an equipment is out of order, together with the actions taken and the outcome.
  - Check that if an equipment is out of order, this is clearly labeled on the machine.
- Calibration
  - If applicable, check if calibration is performed when needed, and if a record of these calibrations is filed in the lab file.

- *Infection control and Waste management*
  - Check that the laboratory is clean, if the benches are disinfected daily, if the required personal protection is used (lab coats, gloves, masks, etc.).
  - Check that a site-specific procedure for waste management is in place and is correctly applied.
  - Check if the procedure for the management of expired products (SOP in preparation for Nidiag) is correctly applied.

- *Laboratory File*
  - Review the laboratory file and check if it is complete (Refer to SOP-WP6-DOC-03 Management of study documents): all SOPs, all quality control forms, all filled packing lists, study specimen log, index test log shipment information, normal values, etc.

A laboratory supervision report of the visits is prepared, documenting all the above, which is sent to the WP6 coordinator, the WP2 leader/delegate, the country coordinating investigator/delegate, and to the site Principal Investigator for follow-up on findings and corrective actions.

4. Definitions and Abbreviations

4.1 Definitions

Laboratory File (LF): The Laboratory File includes all essential documents related to the laboratory work at the site level. There is one Laboratory File per study site. The laboratory personnel is responsible for keeping it updated and for ensuring it is stored adequately.

Site Investigator File (SIF): The Site File includes all essential documents and forms related to the conduct of the study at the site level. There is one Site File per study site. The site investigators are responsible for keeping it updated and for ensuring it is stored adequately.

Quality Assurance (QA): All those planned and systematic actions that are established to ensure that the study is performed and the data are generated, documented (recorded), and reported in compliance with Good Clinical Practice (GCP) and the applicable regulatory requirement(s).

Quality Control (QC): The operational techniques and activities undertaken within the quality assurance system to verify that the requirements for quality of the trial-related activities have been fulfilled.

<u>Source Document</u>: These are original documents. They consist of data relevant to the current research such as medical records, administrative files, laboratory reports, consultation reports, pharmacy dispensation registers, etc...

4.2 Abbreviations

CRF: Case Report Form

EC: Ethics Committee

EQC: External Quality Control

GCP: Good Clinical Practice

GCLP: Good Clinical Laboratory Practice

GDP: Good Documenting Practice

IFU: Instruction For Use

IQC: Internal Quality Control

LF: Laboratory File

PI: Principal Investigator

RDT: Rapid Diagnostic Test

QM: Quality Manager

SIF: Site Investigator File

5. Records and archives

**Appendices & Forms for completion**	
**Number**	**Title**

6. Document History

**Revision**	

**Name and function**	**Date**	**Signature**
Author		
Barbara Barbé	23/02/2014	
Reviewed by		
Raffaella Ravinetto	17/04/2014	
Approved by		
Ninon Horié	06/05/2014	

SOP-WP6-QUAL-05-V2-20Aug2013

	**SOP Title:** Storing and handling RDTs
	Project/Study: NIDIAG: This SOP applies to the NIDIAG Fever study.

1. Scope and application

This SOP describes the storing and handling of RDTs. This SOP focusses on, but is not limited to, the index tests that are evaluated for the NIDIAG fever study.

2. Responsibilities

Function	Responsibilities
Laboratory technician	- Comply to this SOP<br>- Ensure correct storage of the RDTs<br>- Ensure correct stock management of the RDTs<br>- Keep the Index test log form up-to-date<br>- Daily temperature recording of storage room
Laboratory Manager	- Supervision of the activities mentioned above
Quality Manager	- Verify correct storage of RDTs<br>- Verify Index test log form<br>- Verify stock management of the RDTs<br>- Propose corrective actions if inconsistencies to this SOP are noticed

3. Procedures

3.1 Materials

- Forms described in “4. Records and archives”
- Min-Max thermometer
- Lockable cupboard
- Biohazard waste container

3.2 Procedure

3.2.1 Handling of RDTs

3.2.1.1 General regulations

1. Wear gloves.
2. Handle all specimens as potentially infectious.
3. Bring all reagents/components to room temperature before testing.
4. Do not use the test device after expiry date.
5. Do not use the test device if the foil pouch is damaged or if the seal is broken.
6. Keep the pouch sealed until use.
7. Perform the test immediately after opening of the pouch.
8. Check the desiccant that is included in the pouch for color change. Discard the device if the color has changed (= desiccant is saturated). Use a new test device.

9. Label the test device (or the tube in case of a dipstick) with the patient's ID number and date.
10. Do not mix components from different kits/lot numbers.
11. Some buffers (assay diluents) contain sodium azide as preservative. Avoid direct skin contact!
12. Do not replace the buffer (assay diluent) by other fluids.
13. Do not reuse the test device.
14. Discard used test devices, samples and potentially contaminated materials in a biohazard waste container.

3.2.1.2 Specific handling and procedures for Nidiag RDTs

1. Refer to the specific Nidiag SOPs for the handling and the procedures of the specific RDTs used for the Nidiag studies.

3.2.2 Storage of RDTs

3.2.2.1 General storage conditions for RDTs

1. Store the RDT kits in a dry and cool place, protected from direct sunlight.
2. Store the RDT kits according to the temperature ranges specified by the manufacturer.
3. DO NOT freeze RDTs or kit components.
4. Some RDTs can be stored in the fridge. Bring the tests to room temperature before use.

3.2.2.2 Specific storage conditions for index tests

1. Store the index tests in a locked cupboard.
2. Restrict access of the cupboard to authorized Nidiag personnel.
   Comment: Only use the index tests for patients enrolled in the Nidiag studies.
3. Store the index tests according to the temperature ranges in **table 1**. Try to avoid as much as possible temperatures above the upper temperature limit.
4. Perform twice daily temperature recording of the storage room. Use the Temperature Registration Form in **annex 1**. If the index tests are stored in the fridge, use SOP-WP6-QUAL-06-annex1
   Comment: Refer to **SOP-WP6-QUAL-06** for installation of Min-Max thermometer and temperature recording. The probe of the Min-Max thermometer can but does not have to be put in a tube with water when used to record the room temperature.
5. Record the room temperature of the room where the index testing takes place in the CRF, when performing the index tests.

Table 1. Temperature ranges for storage of index tests

Index test	Temperature range
Gambiense-Sero-K-Set (Coris Bioconcept)	4°C - 30°C
HAT FIND dipstick (Standard Diagnostics)	1 - 40°C
CATT R250	CATT antigen:<br>- lyophilized: 2 - 8°C or -20°C<br>- reconstituted: 2 - 8°C (1 week) or 37°C (8 hours)<br>CATT buffer: 2 - 8°C.<br>Positive/negative control lyophilized: 2 - 8°C or -20°C
Typhidot Rapid IgM (Reszon Diagnostics)	2 - 28°C
Test-it Typhoid IgM (Life Assay)	4 - 28°C
Test-it Leptospira IgM (Life Assay)	4 - 28°C
Salmonella typhi IgG/IgM (Standard Diagnostics)	2 - 30°C
Leptospira IgG/IgM (Standard Diagnostics)	1 - 30°C
rK28 ICT (Ease-Medtrend)	- Test cards and whole blood treatment solution: 2 - 30°C<br>- Conjugate reagent:<br>- unopened : 2 - 30°C<br>- after opening: 2 - 8°C
rK39 IT LEISH (BioRad)	2 - 30°C

3.2.3 Stock management and accountability of index tests

3.2.3.1 Index log form

1. Record for each index test which lot numbers were used for which patients in the Index Log Form in **annex 2**.
2. Keep the Index Log Form up-to-date.
3. File the Index Log Form in the Lab file.

3.2.3.2 Stock cards and monthly inventories

1. Use a stock card for each lot number of each index test, according to **SOP-WP6-QUAL-07**.
2. Do a physical count of the index tests at the end of each month and fill out the inventory form, of **SOP-WP6-QUAL-07-annex2** (sheet "consumables"), according to **SOP-WP6-QUAL-07.**

4. Records and archives

Appendices and forms to complete	
**Number**	**Title**
CRF fever	Temperature registration form – Room temperature

SOP-WP6-QUAL-05-V01-08Feb2013-annex1	Index log form
SOP-WP6-QUAL-05-V01-20Aug2013-annex2	Temperature registration form - Fridge
SOP-WP6-QUAL-06-V1.1-04Feb2013-annex1	Stock card
SOP-WP6-QUAL-07-V1.1-04Feb2013-annex1	Stock inventory form
SOP-WP6-QUAL-07-V1.1-04Feb2013-annex2	

5. Documents History

**Revision**	
SOP-WP6-QUAL-05-V01-08Feb2013	Initial version
SOP-WP6-QUAL-05-V02-20Aug2013	Adaptation of table 1: addition of CATT R250 and adaptation of storage temperatures for rk28 index test.

**Name and function**	**Date**	**Signature**
Author		
Barbara Barbé	20/08/2013	
Reviewed by		
Approved by		
Ninon Horié	28/08/2013	

SOP-WP6-QUAL-05-V1-08Feb2013-annex1

Name of hospital/Laboratory:

Daily temperature registration form (Storage room)

Month/Year:____________________________ Storage room ID:__________________________

Location:__________________________________

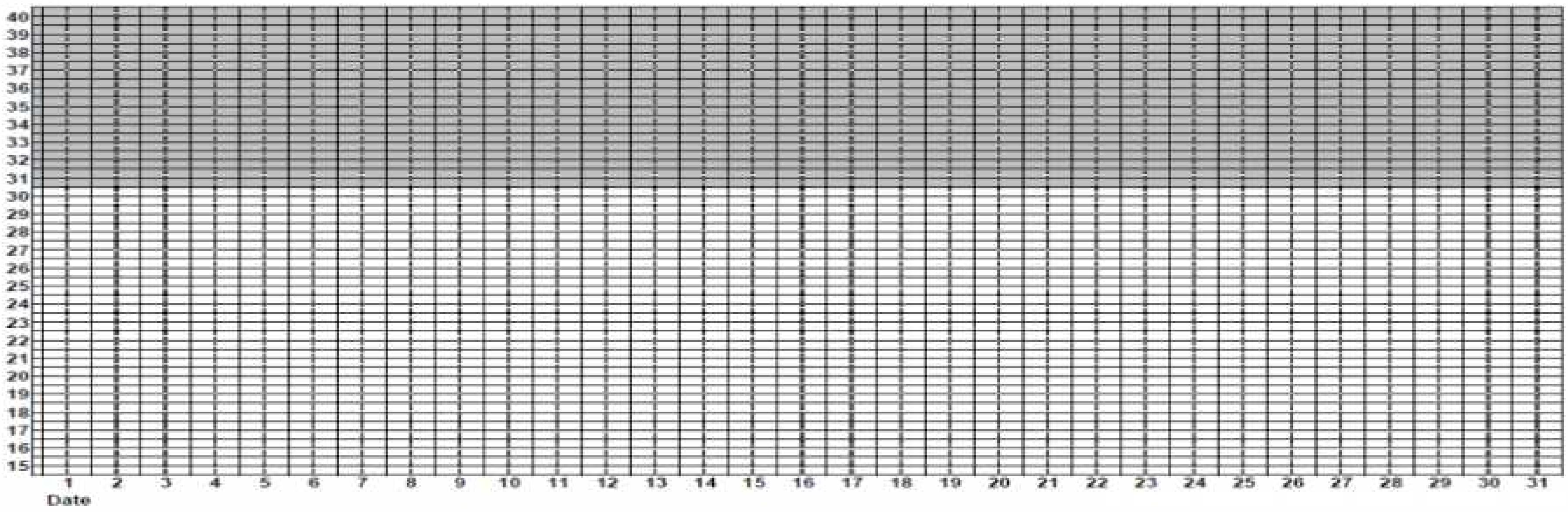

Date	1	2	3	4	5	6	7	8	9	10	11	12	13	14	15	16	17	18	19	20	21	22	23	24	25	26	27	28	29	30	31
MIN T (℃)																															
MAX T (℃)																															
INITIALS LAB TECH																															

Action report:

Date	Action taken	Result	Initials lab tech	Comments

SOP-WP6-QUAL-05-Annex 1

Reviewed by:____________________

Index Test Log

Study Site :

Name of Country PI :

Name of Lab supervisor :

- **Gambiense-Sero-K-Set (Coris Bioconcept)**

Lot n° ___________________ was used from patient n° ☐☐☐☐☐-☐☐ to patient n° ☐☐☐☐☐-☐☐

Lot n° ___________________ was used from patient n° ☐☐☐☐☐-☐☐ to patient n° ☐☐☐☐☐-☐☐

Lot n° ___________________ was used from patient n° ☐☐☐☐☐-☐☐ to patient n° ☐☐☐☐☐-☐☐

☐ NA (Sudan, Nepal and Cambodia)

- **HAT FIND dipstick (Standard Diagnostics)**

Lot n° ___________________ was used from patient n° ☐☐☐☐☐-☐☐ to patient n° ☐☐☐☐☐-☐☐

Lot n° ___________________ was used from patient n° ☐☐☐☐☐-☐☐ to patient n° ☐☐☐☐☐-☐☐

Lot n° ___________________ was used from patient n° ☐☐☐☐☐-☐☐ to patient n° ☐☐☐☐☐-☐☐

☐ NA (Sudan, Nepal and Cambodia)

- **CATT R250**

Lot n° ___________________ was used from patient n° ☐☐☐☐☐-☐☐ to patient n° ☐☐☐☐☐-☐☐

Lot n° ___________________ was used from patient n° ☐☐☐☐☐-☐☐ to patient n° ☐☐☐☐☐-☐☐

Lot n° ___________________ was used from patient n° ☐☐☐☐☐-☐☐ to patient n° ☐☐☐☐☐-☐☐

☐ NA (Sudan, Nepal and Cambodia)

- **TyphiDot Rapid IgM (Reszon Diagnostics)**

Lot n° ___________________ was used from patient n° ☐☐☐☐☐-☐☐ to patient n° ☐☐☐☐☐-☐☐

Lot n° ___________________ was used from patient n° ☐☐☐☐☐-☐☐ to patient n° ☐☐☐☐☐-☐☐

Lot n° ___________________ was used from patient n° ☐☐☐☐☐-☐☐ to patient n° ☐☐☐☐☐-☐☐

- **Test-it Typhoid IgM (Life Assay)**

Lot n° ___________________ was used from patient n° ☐☐☐☐☐-☐☐ to patient n° ☐☐☐☐☐-☐☐

Lot n° ___________________ was used from patient n° ☐☐☐☐☐-☐☐ to patient n° ☐☐☐☐☐-☐☐

Lot n° ___________________ was used from patient n° ☐☐☐☐☐-☐☐ to patient n° ☐☐☐☐☐-☐☐

- **Test-it Leptospira IgM (Life Assay)**

Lot n° ___________________ was used from patient n° ☐☐☐☐☐-☐☐ to patient n° ☐☐☐☐☐-☐☐

Lot n° ___________________ was used from patient n° ☐☐☐☐☐-☐☐ to patient n° ☐☐☐☐☐-☐☐

Lot n° ___________________ was used from patient n° ☐☐☐☐☐-☐☐ to patient n° ☐☐☐☐☐-☐☐

- **Salmonella typhi IgG/IgM (Standard Diagnostics)**

Lot n° ___________________ was used from patient n° ☐☐☐☐☐-☐☐ to patient n° ☐☐☐☐☐-☐☐

Lot n° ___________________ was used from patient n° ☐☐☐☐☐-☐☐ to patient n° ☐☐☐☐☐-☐☐

Lot n° ___________________ was used from patient n° ☐☐☐☐☐-☐☐ to patient n° ☐☐☐☐☐-☐☐

- **Leptospira IgG/IgM (Standard Diagnostics)**

Lot n° ___________________ was used from patient n° ☐☐☐☐☐-☐☐ to patient n° ☐☐☐☐☐-☐☐

Lot n° ___________________ was used from patient n° ☐☐☐☐☐-☐☐ to patient n° ☐☐☐☐☐-☐☐

Lot n° ___________________ was used from patient n° ☐☐☐☐☐-☐☐ to patient n° ☐☐☐☐☐-☐☐

- **rK28 ICT (Ease-Medtrend)**

Lot n° ___________________ was used from patient n° ☐☐☐☐☐-☐☐ to patient n° ☐☐☐☐☐-☐☐

Lot n° ___________________ was used from patient n° ☐☐☐☐☐-☐☐ to patient n° ☐☐☐☐☐-☐☐

Lot n° ___________________ was used from patient n° ☐☐☐☐☐-☐☐ to patient n° ☐☐☐☐☐-☐☐

☐ NA (DRC and Cambodia)

- **rK39 IT LEISH (BioRad)**

Lot n° ___________________ was used from patient n° ☐☐☐☐☐-☐☐ to patient n° ☐☐☐☐☐-☐☐

Lot n° ___________________ was used from patient n° ☐☐☐☐☐-☐☐ to patient n° ☐☐☐☐☐-☐☐

Lot n° ___________________ was used from patient n° ☐☐☐☐☐-☐☐ to patient n° ☐☐☐☐☐-☐☐

☐ NA (DRC and Cambodia)

SOP-WP6-QUAL-06-V1.1-04Feb2013

	**SOP Title:** How to install and use the "Min/Max" thermometer?
	Project/study: This SOP applies to all NIDIAG studies

1. Scope and application

This SOP describes the installation and the use of the Min/Max thermometer, in order to allow daily temperature monitoring by measuring the current temperature, the maximum and minimum temperature of a refrigerator, -20°C freezer, incubator, or water bath, to ensure its performance.
A probe is submerged in a fluid (water for refrigerators, incubators or water baths ; glycerol for freezers) to avoid temperature fluctuations when opening the fridge/freezer's door.

2. Responsibilities

Function	Activities
Laboratory technician/Quality manager/Laboratory manager	- Installation of the Min/Max thermometer<br>- Daily temperature monitoring<br>- Daily temperature recording (SOP-WP6-QUAL-06-V1.1-04Feb2013-annex1-4)

3. Procedures

3.1 Materials

- Min/Max thermometer – 50°C/+70°C
- Water/Glycerol 87%
- Plastic tube, 10 ml
- Silicones/glue

3.2 Procedure

3.2.1. Preparation of the thermometer

1. Install the battery in the thermometer with correct polarity positioning (+ side up).
   (If nothing appears on the display, replace the battery).
2. Fill a plastic tube with water (for refrigerator/incubator) or glycerol (for freezer)

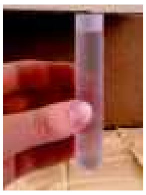

Figure 1: Fill the plastic tube while leaving a bit of space for the probe and

1. Make a hole in the plastic cap to allow the probe to pass. Submerge the probe of the thermometer in the plastic tube and close the cap. Fill up the cap with silicones/glue. Avoid air bulls. Let dry for 24 hours.

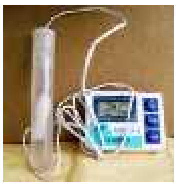

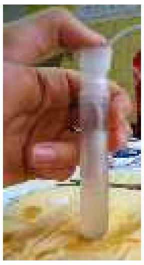

3.2.2 Installation of the thermometer

1. Attach the tube with the sensor inside the fridge/-20°C freezer/incubator/water bath, at the middle of the equipment.
   <u>Comment</u>: The Min/Max thermometer **CANNOT** be used for -80°C freezers!
2. Attach the thermometer on the outside of the equipment, by using the magnets on the back of the thermometer.

3.2.3 Temperature monitoring

1. Check that the temperature is displayed in °C, if not, select "°C" with the red button "°C/°F" at the back of the thermometer.
2. Check that the temperature inside the equipment is displayed ("Fridge" has to be displayed above the temperature measurement). If not, select "Fridge" with the blue button "ROOM/FRIDGE".
   (If "Room" is selected, the room temperature is displayed)

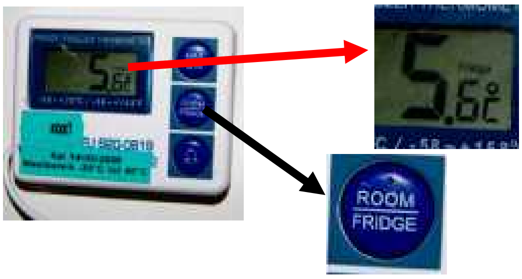

Figure 2: Select "Fridge" with the button "ROOM/ FRIDGE" to display the temperature inside the fridge/freezer/incubator/water bath.

3. Record the current temperature twice daily, once in the morning and once in the evening, on the Daily temperature registration form (SOP-WP6-QUAL-06-V1.1-04Feb2013-annex1-4).
4. Record every evening the maximum and minimum temperatures, by selecting the button "MAX/MIN" on the Daily temperature registration form (SOP-WP6-QUAL-06-V1.1-04Feb2013-annex1-3).

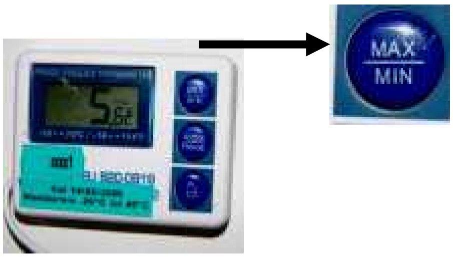

Figure 3: Select the maximum and minimum temperature with the "MAX/MIN" button.

5. After recording of the actual temperature, the maximum and the minimum temperature, reset the min/max values to the current temperature. Press the "MAX/MIN" button for 2 seconds (until the second beep).
6. For -80°C freezers, use the Daily temperature registration form (SOP-WP6-QUAL-06-V1.1-04Feb2013-annex4) to record the temperature that is displayed on the equipment itself twice daily.
   (Min/Max thermometer **CANNOT** be used for – 80°C freezers!)

4. Records and archives

Appendices and forms to complete	
**Number**	**Title**
SOP-WP6-QUAL-06-V1.1-04Feb2013-annex1	Daily temperature registration form - Fridge
SOP-WP6-QUAL-06-V1.1-04Feb2013-annex2	Daily temperature registration form - Incubator
SOP-WP6-QUAL-06-V1.1-04Feb2013-annex3	Daily temperature registration form - Freezer -20°C
SOP-WP6-QUAL-06-V1.1-04Feb2013-annex4	Daily temperature registration form - Freezer -80°C

5. Document History

Revision	
SOP-WP6-QUAL-06-V01-19Sep2012	Initial version
SOP-WP6-QUAL-06-V1.1-04Feb2013	Translation in English

Name and function	Date	Signature
Author		
Barbara Barbé	04/02/2013	
Reviewed by		
Approved by		
Emilie Alirol	05/02/2013	

Name of hospital/Laboratory:

Daily temperature registration form (Fridge)

Month/Year:________________________ Fridge ID:______________________ (Expected range: 2 - 8°C)

Location:____________________________

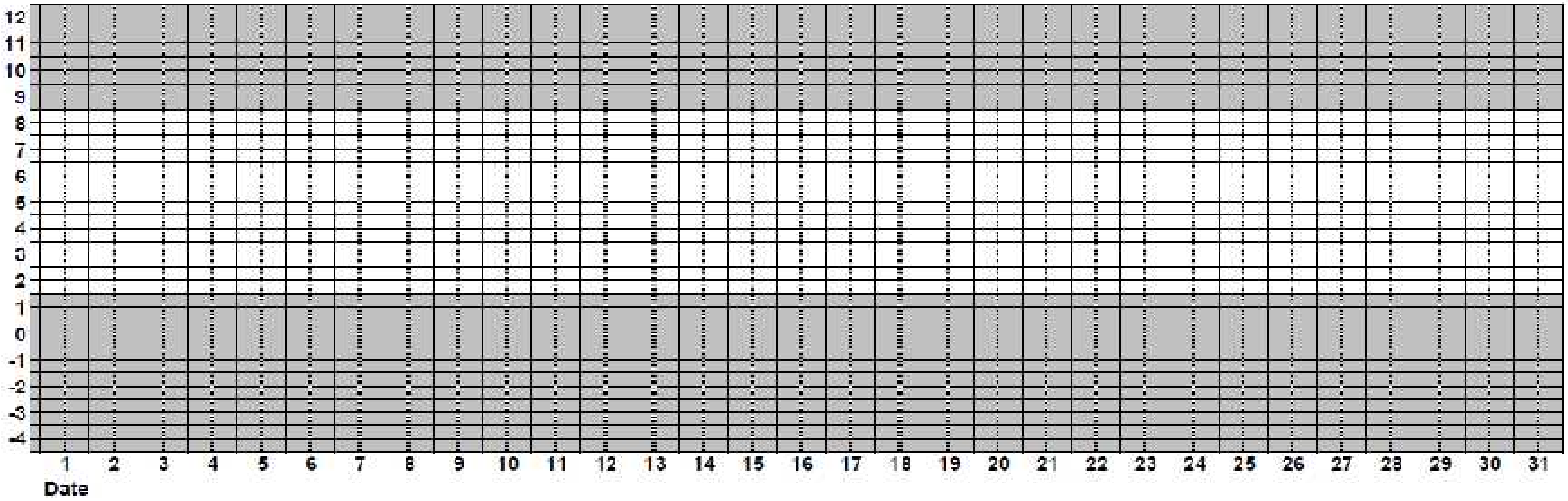

Date	1	2	3	4	5	6	7	8	9	10	11	12	13	14	15	16
MIN T (°C)																
MAX T (°C)																
INITIALS LAB TECH																

Date	17	18	19	20	21	22	23	24	25	26	27	28	29	30	31
MIN T (°C)															
MAX T (°C)															
INITIALS LAB TECH															

Action report:

Date	Action taken	Result	Initials lab tech	Comments

Reviewed by:________________

Name of hospital/Laboratory:

Daily temperature registration form (Incubator)

Month/Year:________________________ Incubator ID:______________________ (Expected range: 34 - 37°C)

Location_____________________________

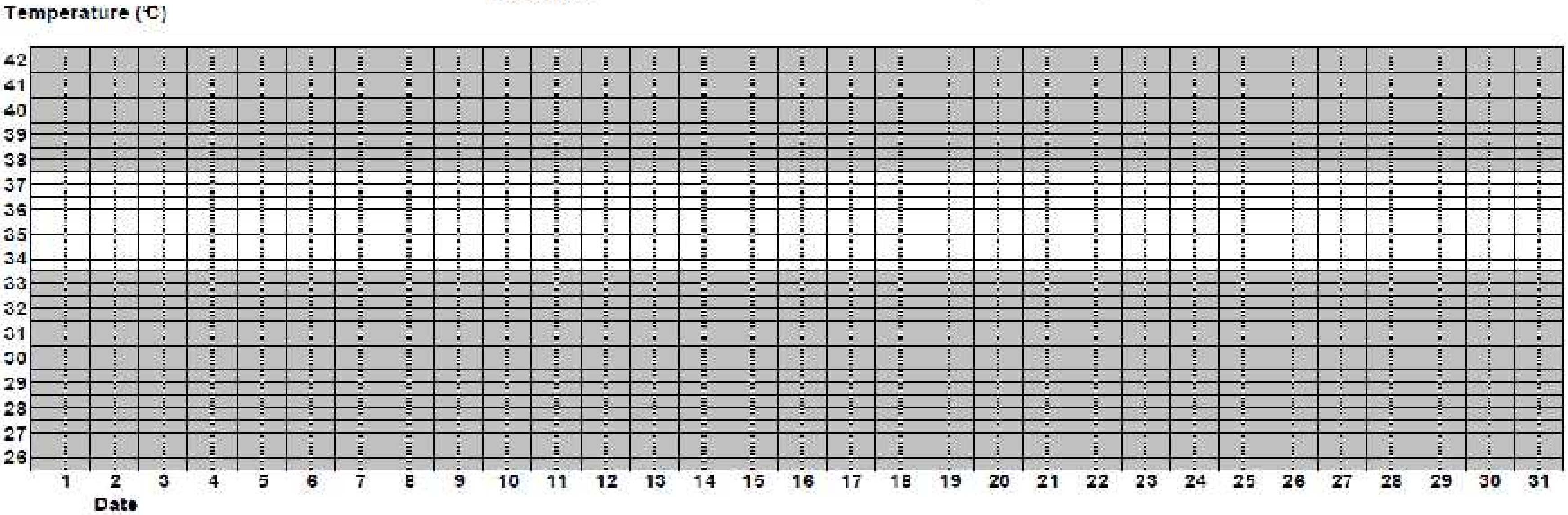

Date	1	2	3	4	5	6	7	8	9	10	11	12	13	14	15	16	17	18	19	20	21	22	23	24	25	26	27	28	29	30	31
MIN T (°C)																															
MAX T (°C)																															
INITIALS LAB TECH																															

Action report:

Date	Action taken	Result	Initials lab tech	Comments

SOP-WP6-QUAL-06-Annex 2 Reviewed by:________________

nidiag

Name of hospital/Laboratory:

Daily temperature registration form (-20℃ freezer)

Month/Year__________________________ Freezer ID:_______________________ (Expected range: < - 18°C)

Location:______________________________

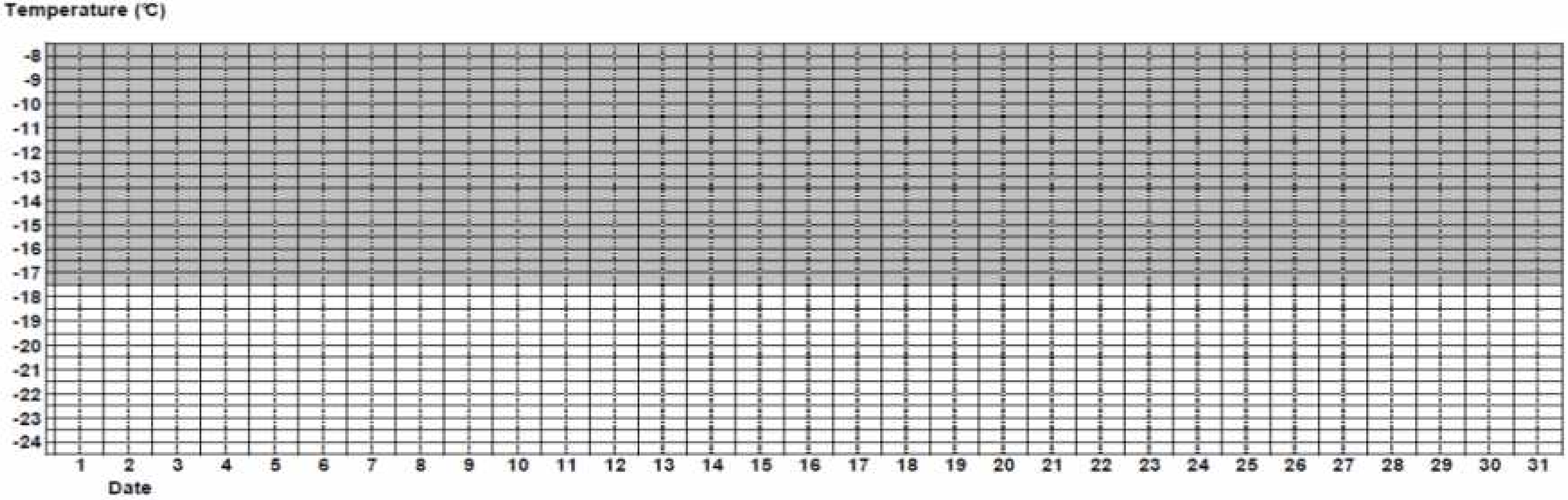

Date	1	2	3	4	5	6	7	8	9	10	11	12	13	14	15	16	17	18	19	20	21	22	23	24	25	26	27	28	29	30	31
MIN T (℃)																															
MAX T (℃)																															
INITIALS LAB TECH																															

Action report:

Date	Action taken	Result	Initials lab tech	Comments

SOP-WP6-QUAL-06-Annex 3 Reviewed by:__________________

Name of hospital/Laboratory:

Daily temperature registration form (-80℃ freezer)

Month/Year__________________________ Freezer ID:______________________ (Expected range: < - 65°C)

Location:_____________________________

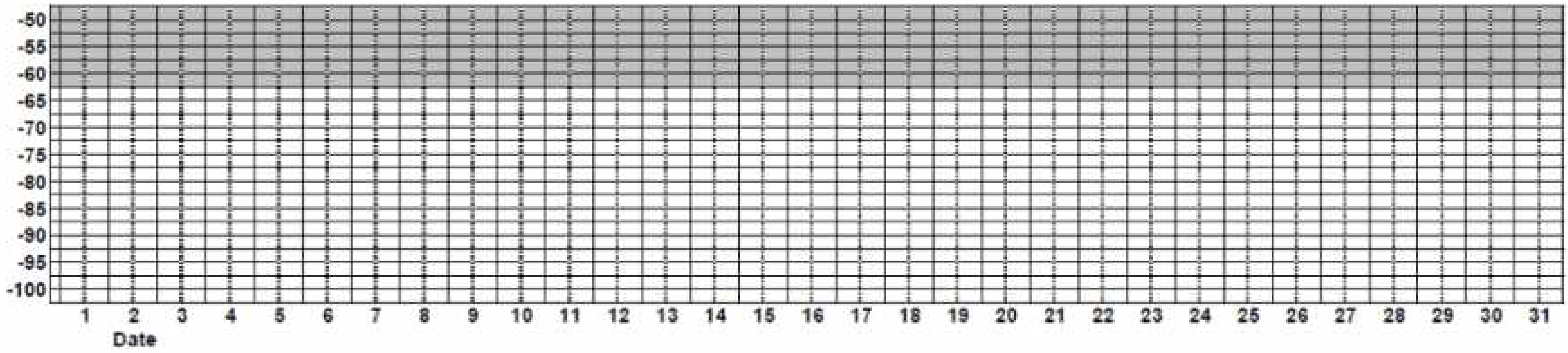

Date	1	2	3	4	5	6	7	8	9	10	11	12	13	14	15	16	17	18	19	20	21	22	23	24	25	26	27	28	29	30	31
MIN T (℃)																															
MAX T (℃)																															
INITIALS LAB TECH																															

Action report:

Date	Action taken	Result	Initials lab tech	Comments

SOP-WP6-QUAL-06-Annex 4 Reviewed by:__________________

SOP-WP6-QUAL-07-V1.1-04Feb2013

	**SOP Title:** Stock management
	Project/Study: This SOP applies to all NIDIAG WP2 clinical studies.

1. Scope and application

This SOP describes the stock management of consumables and materials that are used for NIDIAG, by using stock cards and performing monthly inventories.

2. Responsibilities

Function	Activities
Laboratory technician / Laboratory manager	- Stock tracking of consumables and materials.<br>- Management of machines.<br>- Recording of entries and exits of stock on the stock cards.<br>- Monthly physical inventory of all stock.

3. Procedures

3.1 Materials

- Stock card (cf. SOP-WP6-QUAL-07-V1.1-04Feb2013-annex1)
- Monthly inventory form (manual recording on print-out of document or computer file (cf. SOP-WP6-QUAL-07-V1.1-04Feb2013-annex2)

3.2 Procedure

3.2.1 Stock cards

1. Record all the entries ("IN") of the consumables or materials on the stock cards. **Use 1 stock card per product and per expiry date.**
2. Record all exits ("OUT") of consumables or materials from the stock to the laboratory on the stock cards.

   Comment:

   - Record each time a box is removed from the stock and sent to the laboratory.
   - Record the number of tests, not the number of boxes that are removed.

   E.g. malaria RDTs: packaging is 25 tests per box. The exit of 2 boxes of malaria RDTs is recorded as an exit of 50 tests (units).

3.2.2 Monthly inventory

1. Do a physical count of all reagents and consumables at the end of each month and fill out the inventory form, sheet "consumables" of SOP-WP6-QUAL-07-V1.1-04Feb2013-annex2:

- Record the stock that was present at the beginning of the month (= physical count at the end of last month)
- Record the stock received during the month (= products delivered during that month)
- Record the stock still present at the end of the month (= physical count at the end of this month)

<u>Comment</u>: the stock is recorded per test (not per box), per bottle (not per kit), etc.

2. Calculate the consumption of the consumables of each month by using the following formula:

   Consumption of this month =
   (Initial stock at beginning of the month + Stock received during the month) – Final stock at the end of the month

3. Do a physical count of the materials at least once every 3 months and record the information on the sheet "materials" of the same document (SOP-WP6-QUAL-07-V1.1-04Feb2013-annex2). Do not calculate consumption for materials: materials are re-usable (e.g. micropipette). Record when a material breaks down or is out of use under "comments".

4. Record the machines in use for the NIDIAG on the sheet "machines" of the document (SOP-WP6-QUAL-07-V1.1-04Feb2013-annex2). Record if the machine functions properly, or – if not – what are the problems and which actions have been taken. Regular maintenance activities can be recorded under "comments". Fill out the form every month.

4. Records and archives

Appendices and forms to complete	
**Number**	**Title**
SOP-WP6-QUAL-07-V1.1-04Feb2013-annex1	Stock card
SOP-WP6-QUAL-07-V1.1-04Feb2013-annex2	Excel document for stock inventory

5. Document History

Revision	
SOP-WP6-QUAL-07-V01-24Sep2012	Initial version
SOP-WP6-QUAL-07-V1.1-04Feb2013	Translation in English

Name and function	Date	Signature
Author		
Barbara Barbé	04/02/2013	
Reviewed by		
Approved by		
Emilie Alirol	07/02/2013	

SOP-WP6-QUAL-07-V1.1-04Feb2013-annex1

nidiag

STOCK CARD (SOP-WP6-QUAL-07-V1.1-04Feb2013-annex1)

Description of product	....		
LOT N°	....	Expiry date	....
Packaging (units/package)	....	Comments	....

DATE	IN (units)	OUT (units)	STOCK (units)	Comments	Initials Signature
STOCK TRANSFERRED FROM PREVIOUS CARD					

DATE	IN (units)	OUT (units)	STOCK (units)	Comments	Initials Signature
STOCK TRANSFERRED FROM PREVIOUS CARD					

Stock management of materials

Initials/signature of lab tech:

PERIOD:

	Description of material	Reference number	Initial stock Date: / /	Stock received Date: / /	Final stock Date: / /	Comments
1						
2						
3						
4						
5						
6						
7						
8						
9						
10						
11						
12						
13						
14						
15						
16						
17						
18						
19						
20						
21						
22						

	**SOP Title :** Handling of expired & disqualified products
	Project/Study : This SOP is applicable to all NIDIAG studies

1. Scope and application

This document gives instructions on how to handle expired products and products that are disqualified as "unfit for purpose" (drugs, laboratory reagents, RDT kits, etc.) in the NIDIAG studies.

2. Responsibilities

Function	Activities
Laboratory technician	• Collect all expired or disqualified laboratory products.<br>• Centralize all expired laboratory products in the central stock of the site.
Clinician	• Collect all expired drugs.<br>• Centralize all expired drugs in the central stock of the site.
Site investigator	• Ensure that all expired or disqualified products are packed in a box marked "Out of use".<br>• Transport all expired and disqualified products once a month to the coordination site (if applicable).
Principal investigator	• Receive and store all expired and disqualified products at the central stock of the coordination site as "Out of use" until the end of the study.<br>• Use a logbook to register all expired and disqualified products.<br>• Keep the logbook up to date.

3. Procedures

3.1 Procedure on the site

- Always verify the expiration date before using a product (laboratory reagent, RDT kit, drug, etc.)
- If expired or disqualified, do not use the product! Store the product in a box marked with "OUT OF USE!" in the central stock of the site.
- At the end of the month, transport the box(es) with the expired and disqualified products to the coordination site (if applicable).

3.2 Procedure at the country coordination level

- Use a logbook to register all expired and disqualified products (See example in **Annex 1**).
- Receive the expired and disqualified products. Update the logbook after each reception.
- Store the products at the central stock of the coordination site in a box(es) marked "OUT OF USE!"

- Record the reason for classifying the products as “Out of use” in the logbook under “Reason” (*e.g.* expired product, product unfit for purpose (refer to a report))
- If expired products are used for training or educational purposes, note this in the logbook under “Comments”.
- Destroy the expired and disqualified products according to local waste management procedures at the end of the NIDIAG study.

4. Records and archives

**Appendices and forms to complete**	
**Number**	**Title**
SOP-WP6-QUAL-08-Annex 1	Logbook for expired and disqualified products

5. Document History

**Revision**	
SOP-WP6-QUAL-08-V1.0-05Mar2014	Initial version

**Name and function**	**Date**	**Signature**
Author		
Barbara Barbé	05/03/2014	
Revised by		
Jan Jacobs	05/03/2014	
Approved by		
Ninon Horié	07/03/2014	

Logbook for expired and disqualified products

Product name	Lot number	Expiration date	Unit	Quantity	Date of reception	Reason*	Comment

**e.g.* expired product, product unfit for purpose (refer to a report)
